# Enantioselective Organocatalytic Addition of 1,3-Dicarbonyl Compounds to β-Arylvinyl Triflones

**DOI:** 10.1021/acs.orglett.5c00412

**Published:** 2025-03-19

**Authors:** Michał Kopyt, Jan Dudziński, Michał Barbasiewicz, Piotr Kwiatkowski

**Affiliations:** † University of Warsaw, Faculty of Chemistry, Pasteura 1, 02-093 Warsaw, Poland; ‡ University of Warsaw, Biological and Chemical Research Centre, Żwirki i Wigury 101, 02-089 Warsaw, Poland

## Abstract

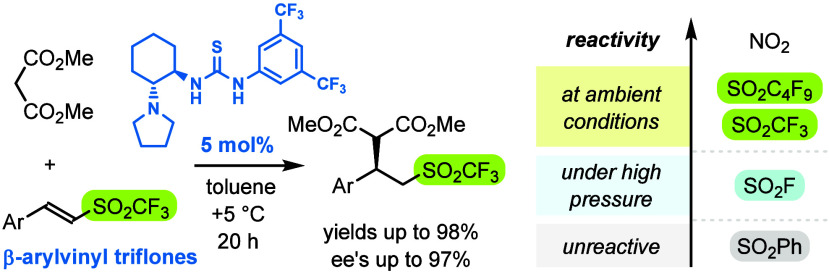

The sulfonyl group is able to polarize adjacent C=C bonds, but strength of the effect considerably varies with sulfonyl substituents (SO_2_X). In this report, we present asymmetric organocatalyzed conjugate addition of 1,3-dicarbonyl compounds to β-arylvinyl triflones (ArCH=CHSO_2_CF_3_). The reaction runs under mild conditions with 5 mol % of tertiary amino-thiourea to afford Michael-type adducts in high yields and enantioselectivities. Comparative experiments reveal that electron-withdrawing properties increase in the series SO_2_F ≪ SO_2_CF_3_ < SO_2_C_4_F_9_, with the latter approaching strength of the nitro group.

Sulfonyl compounds find countless applications in organic synthesis, medicinal chemistry, and material science, spanning from the sulfur fluoride exchange (SuFEx) reaction, Julia olefination, and Oppolzer sultam, to saccharin sweetener, ‘sulfa drugs’, and sulfourea herbicides, to name just a few.^1^ Presence of the sulfonyl group affects bioproperties of the molecules, acting as a moderately lipophilic^2^ electron acceptor and mimicking tetrahedral intermediates of acyl substitution reactions.^1b^ As expected for third-row elements of the periodic table, electronic effect of the sulfonyl group is inductive in nature, in contrast to a predominant resonance character of nitro, carbonyl, and cyano functions. Accordingly, negative charge adjacent to the sulfonyl group is stabilized mainly with n_C_ → σ*_S–X_ hyperconjugation^3^ that translates into changes of p*K*_a_ values of the conjugated acids. For phenylmethanesulfonyl derivatives (PhCH_2_SO_2_X), dissociation constants in DMSO span over *10 orders of magnitude* in a series: X = NMe_2_, p*K*_a_ = 25.2; CH_2_Ph, 23.9; OPh, 19.9; F, 16.9; and CF_3_, 14.6, with the latter approaching acidity of phenylnitromethane (PhCH_2_NO_2_, p*K*_a_ = 12.3).^4^ The sulfonyl substituents influence also adjacent π-electron systems that manifest, for example, in acidity of benzoic acids, for which Hammett constants of the SO_2_CF_3_ group (σ_m_ = 0.76 and σ_p_ = 0.96) surpass values determined for the nitro group (σ_m_ = 0.71, σ_p_ = 0.81).^5^

Surprisingly, relative electrophilicity of α,β-unsaturated sulfonamides, sulfonates, and sulfones remains poorly recognized in the literature, despite their desired biological activity attributed to conjugate (1,4-) addition of nucleophilic residues.^6^ To the best of our knowledge, only report by Roush systematically compared reaction rates of *S*-nucleophiles with unsaturated sulfonyl substrates, tested as model covalent inhibitors of the cysteine proteases.^7^ Experimental data is also available for ethenesulfonyl fluoride (CH_2_=CHSO_2_F, ESF)^8^ and its β-phenyl derivative, demonstrated to be 4.5 orders of magnitude less electrophilic,^9^ and poorly entering addition of 1,3-dicarbonyl substrates.^10^ Pronounced reactivity was also reported for vinyltriflone (CH_2_=CHSO_2_CF_3_) and its longer perfluoroalkyl analogues, designed as fluorous tagging agents for use in fluorous-organic biphasic separation techniques.^11^ Last, but not least, the sulfonyl Michael-type acceptors can be useful substrates for enantioselective metal-catalyzed^12^ and organocatalyzed^13,14^ addition reactions, although such processes in the latter case often require the presence of additional acceptor groups^14^ or use of forceful conditions. Recently, we reported addition of dialkyl malonates to β-arylethenesulfonyl fluorides^15^ (ArCH=CHSO_2_F), catalyzed with chiral tertiary amino-thiourea under pressure of 9 kbar.^16^ Increase of pressure is known to kinetically promote reactions that display negative volume of activation,^17^ and this technique is particularly useful for enantioselective processes, where higher temperatures usually decrease optical purity of the product. Based on the literature data of malonate additions to β-nitrostyrenes,^18^ we expected that substrates more electrophilic than sulfonyl fluorides could react under *ambient conditions*, and β-arylvinyl triflones (ArCH=CHSO_2_CF_3_) seemed to be promising candidates for the task.

Importantly, enantioselective syntheses of triflones^19^ remain scarce, and the only examples of such conjugate additions^20^ concern reports by Deng^21^ and Wennemers.^22^ Deng presented a few examples of Michael reactions with β-alkylvinyl triflones catalyzed by *Cinchona* alkaloid derivatives.^21^ More recently, Wennemers developed efficient tripeptide catalyzed addition of aldehydes to α-arylvinyl triflones with the formation of two nonadjacent stereogenic centers.^22^ In the current report, we present studies of organocatalytic enantioselective addition of 1,3-dicarbonyl substrates to β-arylvinyl triflones and compare their electrophilicity with related Michael-type acceptors.

Our project started from literature search for preparations of β-arylvinyl triflones. Surprisingly, methods of their synthesis suffer from numerous limitations;^23−26^ thus, for initial experimentation, we chose NaOH-promoted condensation of arylaldehydes with methyl triflone (CH_3_SO_2_CF_3_).^27^ Although expected triflones **1a**,**c**,**g**,**i**,**l** (Scheme 1) were formed in the reactions, isolated yields were only moderate (43–79%), likely due to equilibrium character of the process.^28^

**Scheme 1:**
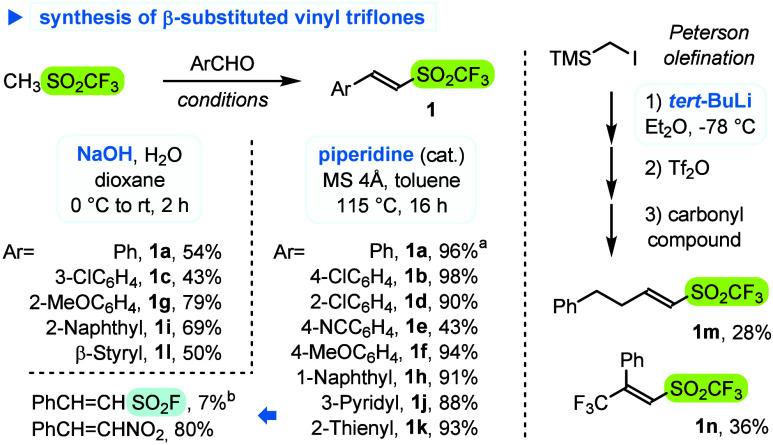
Preparation of Vinyl Triflones **1a**–**n** Triflone **1a** was synthesized at gram scale (4.53 g). Under the piperidine-catalyzed conditions, CH_3_SO_2_F and CH_3_NO_2_ gave condensation products with PhCHO isolated in 7% and 80% of yield, respectively.

Later, the reaction course was improved by application of procedure reported for condensation of nitroalkanes.^29^ Accordingly, a series of aromatic aldehydes was treated with methyl triflone in a sealed tube at 115 °C in toluene in the presence of piperidine catalyst (10 mol %) and 4 Å molecular sieves to afford products **1a,b,d–f,h,j,k**, isolated in much better yields of up to 98%. Finally, more demanding hydrocinnamaldehyde and trifluoroacetophenone were transformed into triflones **1m** and **1n** using variant of the Peterson olefination reaction.^25^

With the synthesized triflones in hand, we tested set of organocatalysts^28^**2a**–**f** (5 mol %) in a model reaction of dimethyl malonate with β-phenylvinyl triflone (**1a**, Scheme 2). To our delight, under ambient conditions, most of the organocatalysts led to the conjugate (1,4-) adduct **3a**, and best results were obtained with pyrrolidine analogue of the Takemoto catalyst (**2d,** 97% of NMR yield, 89% ee). It is worth to stress that the parent Takemoto system (**2a**) was originally developed for addition of malonates to β-nitrostyrenes,^18^ and only minor modifications of its tertiary amino group were required to achieve high enantioselectivities for different classes of the substrates (NMe_2_ for ArCH=CHNO_2_,^18^ piperidine for ArCH=CHSO_2_F,^16^ and pyrrolidine for ArCH=CHSO_2_CF_3_, reported here).

**Scheme 2:**
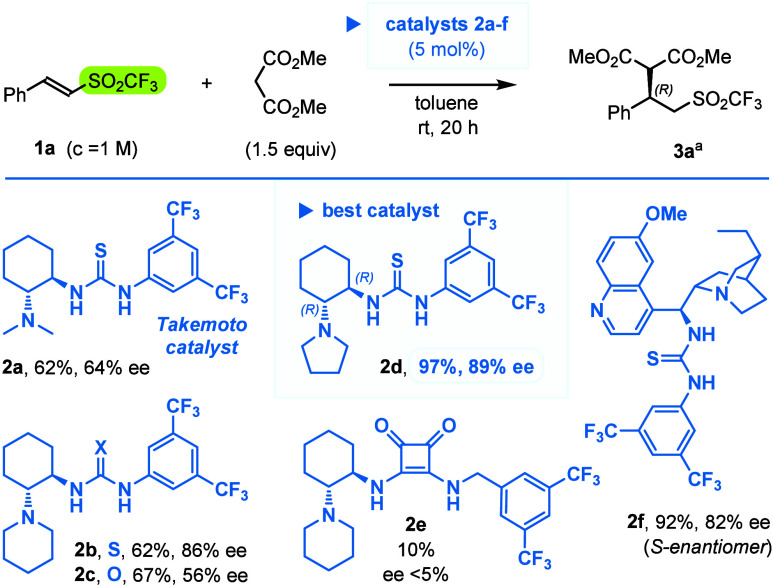
Screening of the Catalysts **2a**–**f** in Model Reaction of Dimethyl Malonate with Triflone **1a** NMR yields and enantiomeric excesses (HPLC) of adduct **3a** are given below the catalysts’ structures.

Thus, the applicability of the catalytic system to various classes of the substrates inspired us to compare their reactivity with dimethyl malonate in the presence of catalyst **2d** (Scheme 3).

**Scheme 3:**
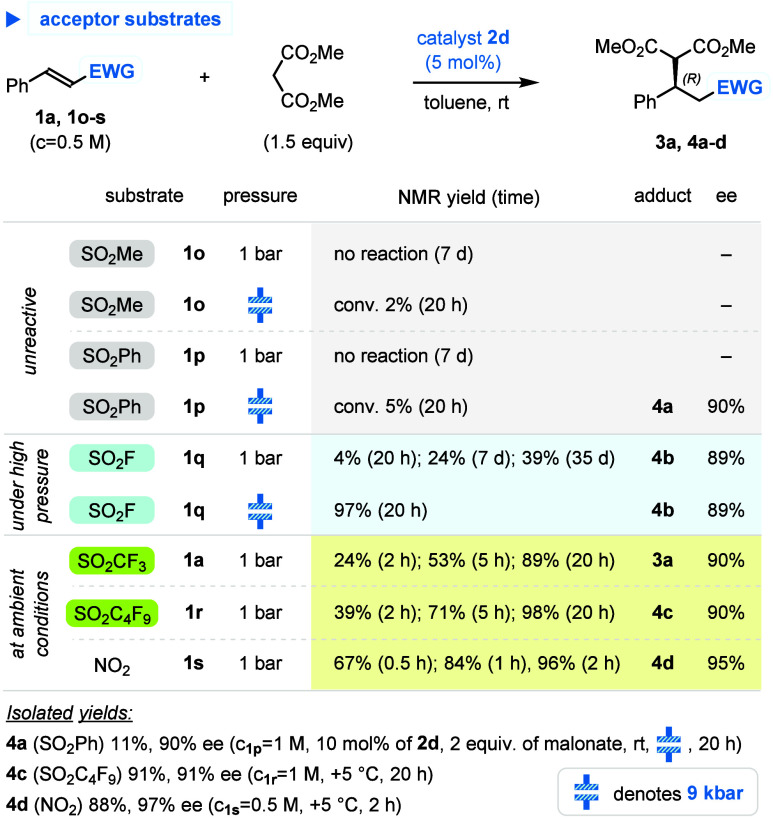
Comparative Studies of Reactivity of Michael Acceptors

Reaction of methyl styryl sulfone (PhCH=CHSO_2_Me, **1o**) was practically ineffective under atmospheric and high-pressure conditions, whereas phenyl styryl sulfone (PhCH=CHSO_2_Ph, **1p**) appeared to be only slightly more reactive. However, in the latter case, a combination of high pressure (9 kbar) and catalyst loading increased to 10 mol % enabled isolation of adduct **4a** in 11% yield and 90% ee. Sulfonyl fluoride (PhCH=CHSO_2_F, **1q**) required a high-pressure activation,^16^ giving after 20 h under 9 kbar adduct **4b** with 97% of NMR yield and 89% ee. Finally, β-phenylvinyl triflone **1a** and nonafluorobutyl sulfone (PhCH=CHSO_2_C_4_F_9_, **1r**)^30^ reacted under *atmospheric pressure* to afford adducts **3a** and **4c** in high NMR yields and 90% ee (with **1r** being ca. twice as reactive as triflone **1a**).^28^ Last, but not least, β-nitrostyrene (**1s**), tested for the sake of comparison, appeared to be a superior acceptor, by combining 96% of yield and 95% ee of adduct **4d** formed after only 2 h. The data revealed that under the **2d**-catalyzed conditions electrophilicity of α,β-unsaturated triflones and nonaflones is much higher than that of sulfonyl fluorides^16^ and approaches reactivity of β-nitrostyrenes. Importantly, absolute configuration of all these products (cf. Scheme 4) turned out to be the same for catalysts derived from (1*R,*2*R*)-1,2-diaminocyclohexane (*R,R*-DACH).^16,18,31^

**Scheme 4:**
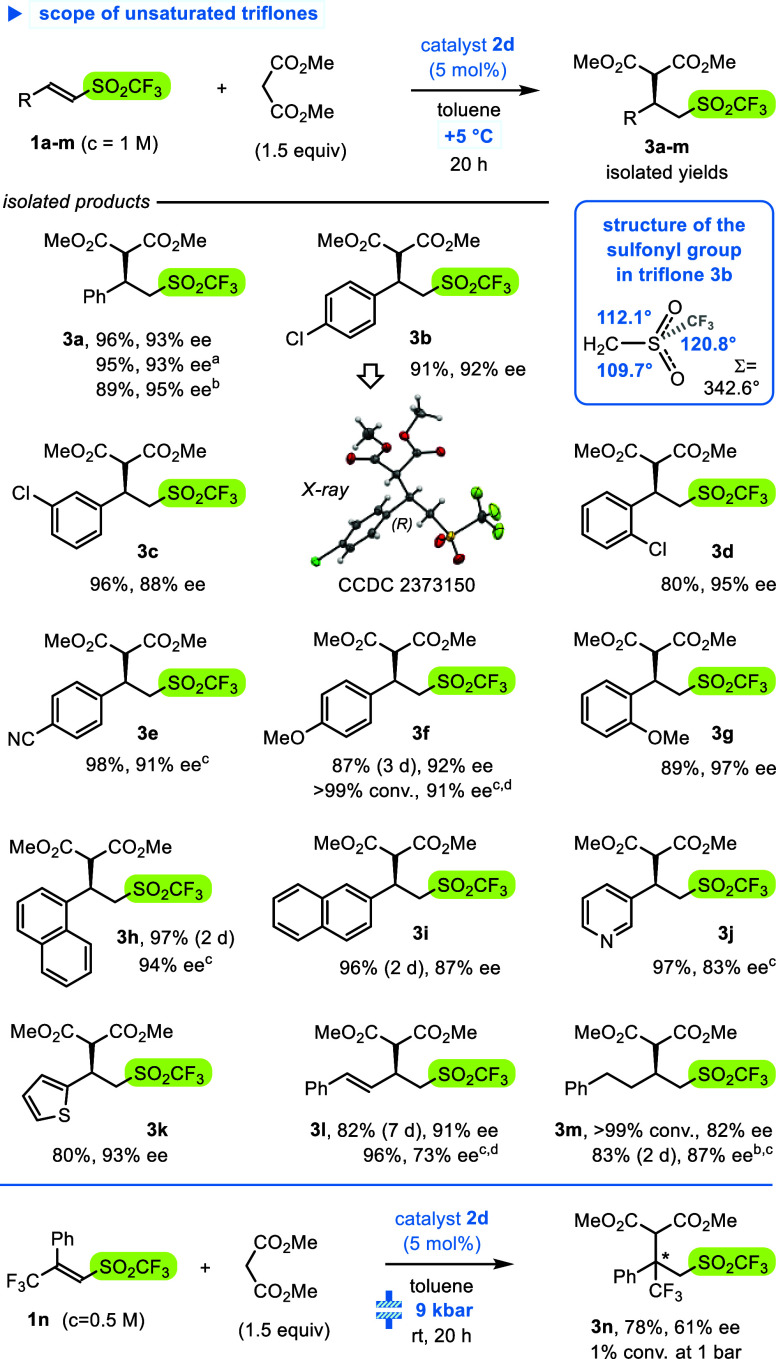
Scope of Unsaturated Triflones **1** in **2d-**Catalyzed Reactions with Dimethyl Malonate The structure and absolute configuration of adduct **3b** were established using X-ray crystallography. Modifications of the reaction conditions: With 2 mol % of **2d** for 48 h. At −15 °C. At *c*_**1**_ = 0.5 M. At 9 kbar, rt, 2 h.

After careful optimization of model reaction with triflone **1a** (including solvents, concentrations, temperatures, reaction times, etc.),^28^ an experiment carried out in toluene solution at +5 °C and substrate concentration *c*_**1a**_ = 1 M afforded dimethyl malonate adduct **3a** isolated in 96% of yield and improved enantioselectivity of 93% ee (Scheme 4). Based on this, we focused on scope and limitations of the procedure by screening triflones **1b**–**m** (Scheme 4). Presence of acceptor and donor substituents at the aromatic ring of ArCH=CHSO_2_CF_3_ displayed little effect on enantioselectivity, although reactivity was diminished in the latter case. Only *ortho*-substitution with Cl and OMe was beneficial for the reaction course, with enantioselectivity increased to 95–97% ee (adducts **3d** and **3g**). Heteroaromatic rings were tolerated, but for 3-pyridyl derivative, enantioselectivity was lowered to 83% (**3j**). Dienyl homologue PhCH=CHCH=CHSO_2_CF_3_ reacted exclusively in a 1,4-addition fashion,^32^ but longer reaction time (7 d) was necessary to obtain product **3l** in a reasonable yield of 82%. Finally, β-alkylvinyl triflone PhCH_2_CH_2_CH=CHSO_2_CF_3_ reacted well but also with lower enantioselectivity of adduct **3m** (82% ee at +5 °C and 87% ee at −15 °C). We tested also a sterically hindered substrate **1n**, with additional CF_3_ group attached at the β-position.^12d^ Unfortunately, the reaction ran exceedingly slowly, and only under high-pressure (9 kbar) adduct **3n**, bearing a quaternary stereogenic center, was formed in 78% yield and 61% ee (Scheme 4, bottom).

In the following step, a series of 1,3-dicarbonyl nucleophiles were combined with triflone **1a** under standard conditions with the catalyst **2d** (Scheme 5). Interestingly, 1,3-diesters and 1,3-diketones reacted well, except 2,2,6,6-tetramethylheptano-3,5-dione, bearing bulky *tert*-butyl groups, for which high pressure (9 kbar) was required to afford product **5e** in high yield. In turn, β-ketoesters formed two diastereoisomers of adducts, while selectivity substantially varied with the enolate precursor. Cyclohexanone and cyclopentanone derivatives **5f,g** were formed as one predominant diastereoisomer (>10:1) and with high enantioselectivities. In turn, adducts of indanone **5h** and open-chain acylacetates were produced as 1.6:1 to 1:1 dr mixtures (**6a**–**e**, Table 1). Interestingly, for acyclic β-ketoesters, both diastereisomers were formed with high enantioselectivities and with the same absolute configuration at the benzylic carbon atom. The observation inspired us for further transformation of produced adducts **6** via hydrolysis and decarboxylation. Accordingly, reaction of **1a** with ethyl acetylacetate catalyzed with 5 mol % of **2d** at +5 °C for 20 h in toluene solution gave adduct **6a** isolated in 90% yield, as a mixture of diastereoisomers (1.25:1). Then, hydrolysis and decarboxylation with a HCl/water/AcOH mixture at 95 °C led to the formal acetone adduct **7a**, isolated in 90% yield and 90% ee (the process run as a one-pot from **1a** gave comparable yield of 84%). Other β-ketoesters bearing *n*-propyl, isopropyl, phenyl, and 2-thienyl groups reacted analogously to afford products **7b**–**e** in the two-step sequences with final enantioselectivities in range of 81–92%. Moreover, for aromatic substrates, the enantioselectivity was improved by a decrease in the reaction temperature to −15 °C (**7d,e**, ee 88–90%). It is worth to mention that the sequential two-step approach significantly exceeds efficiency of direct addition of acetone using covalent organocatalysis,^33^ which in our hands gave **7a**, in only 41% of NMR yield and 63% ee.^28^

**Scheme 5:**
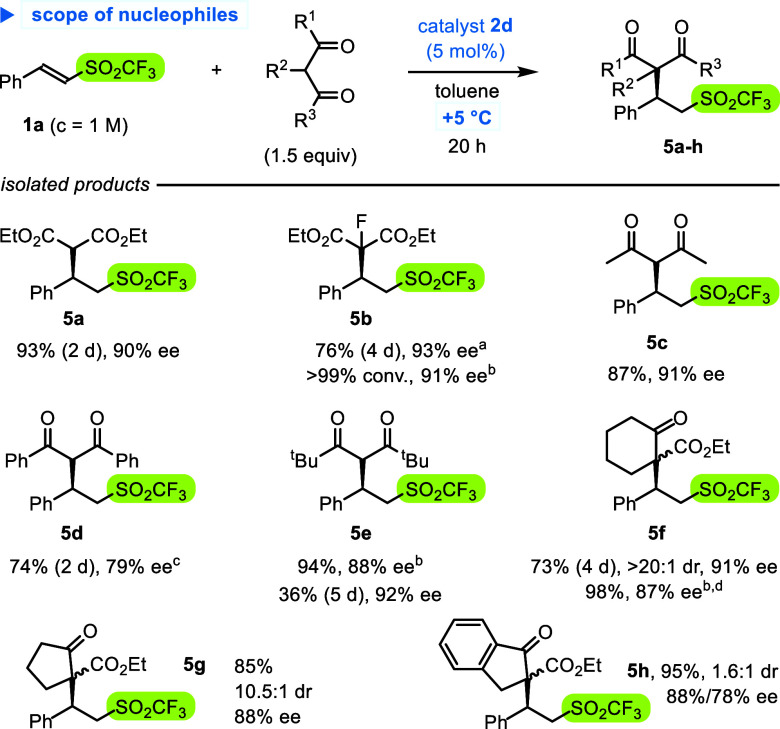
Scope of 1,3-Dicarbonyl Compounds in **2d-**Catalyzed Reactions with Triflone **1a** Modifications of the reaction conditions: 1 equiv of NaHCO_3_. *c*_**1**_ = 0.5 M, 9 kbar, rt, 2 h. ee after the next step (see Scheme 6). NMR yield.

**Table 1:**
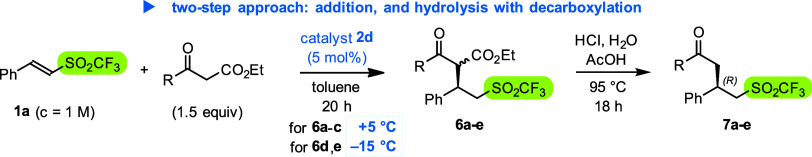
Synthesis of Formal Adducts of Methyl Ketones (**7a**–**e**)

		Products **6a**–**e**		Products **7a**–**e**		
Entry	R =	No.	Yield (%) (dr)	No.	Yield (%)	ee (%)
1	Me	**6a**	90% (1.25:1); 96% of conv.	**7a**	90% (one-pot 84%)	90%
2	*n*-Pr	**6b**	95% (1:1)	**7b**	92%	92%
3	*i*-Pr	**6c**	96% (1:1)	**7c**	94%	91%
4	Ph	**6d**	94% (1.4:1) (92%/92% ee)	**7d**	96%	90% (83%)^a^
5	2-Thienyl	**6e**	97% (1.5:1)	**7e**	96%	88% (81%)^a^

a Enantiomeric excesses of adducts synthesized at +5 °C.

In the last part of the project, we attempted transformations of the enantioenriched triflone adducts (Scheme 6). Surprisingly, adduct **3a** subjected to an excess of DBU formed dimethyl 2-phenylcyclopropane-1,1-dicarboxylate (**8**, 75%) close to a racemic mixture, in a striking contrast to the reaction course with analogous SO_2_F substrate.^16^ The unexpected behavior likely arose from stronger electron-withdrawing properties of the SO_2_CF_3_ group, and resulting ability of adduct **3a** to undergo equilibration via retro-Michael reaction (adduct dissociation). In effect, the substrate racemized faster than it cyclized, as confirmed when **3a** was subjected to catalytic amount of DBU (initially 92% ee; 2 h, 70% ee; 20 h, 24% ee).^28,34^ Cyclization of adduct **3a** was also attempted in the presence of iodine as an oxidant, where carbanion is immediately trapped, prior to the retro-Michael reaction. In this case, degradation of the optical purity was not observed, and *trans*-cyclopropane **9** with preserved triflone group was isolated in 79% yield, and 92% ee. The triflone adduct **3a** was also subjected to hydrolysis-decarboxylation conditions giving acid **10**, and then, the corresponding ethyl ester **11** (92% ee). Finally, diketone adducts **5c** and **5d** were heated with hydroxylamine hydrochloride in EtOH to give isoxazoles **12** and **13** with 91% and 79% ee, respectively.

**Scheme 6:**
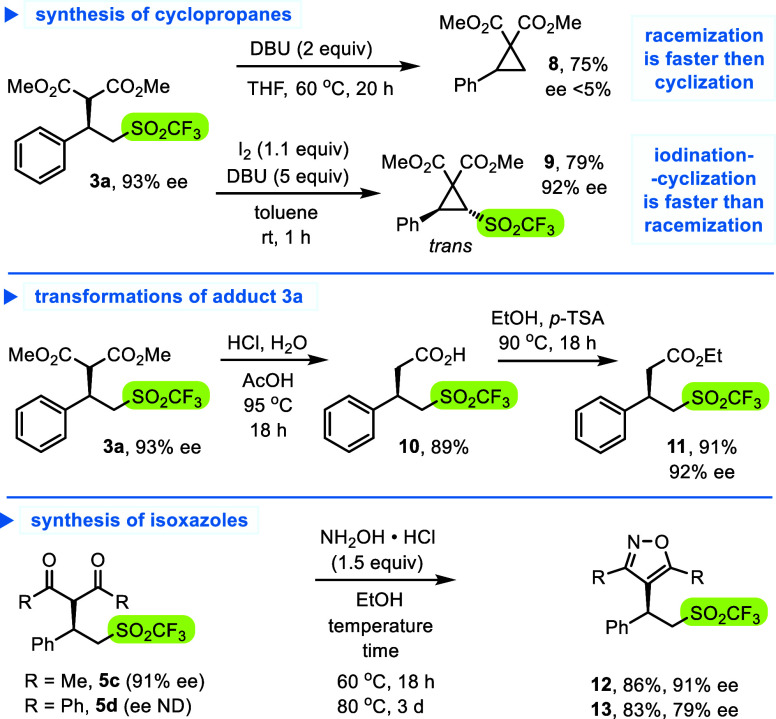
Studies of Cyclization and Other Transformations of Adducts **3a** and **5c,d**

In conclusion, we developed a general and effective organocatalytic addition of 1,3-dicarbonyl compounds to β-substituted vinyl triflones, which ran in high yields and enantioselectivities under mild conditions (+5 °C, 1 bar). Comparative experiments demonstrated that substituents at the sulfonyl group (ArCH=CHSO_2_X) display large effect on the reactivity,^35^ with trifluoromethyl (SO_2_CF_3_) and nona-fluorobutyl (SO_2_C_4_F_9_) sulfones being the most powerful acceptors. The reactions are catalyzed with easily available tertiary-amino thioureas, derived from the Takemoto catalyst, originally developed for addition of malonates to β-nitrostyrenes.^18^ Importantly, adducts to the electrophilic sulfones possess the same absolute configuration, as adducts to β-nitrostyrenes,^31^ and both classes of the substrates differ in reactivity to only limited extent. Therefore, the NO_2_ and SO_2_CF_3_ groups may act as functional isosteres that pave the way for their interchangeable applications in noncovalent organocatalysis.

## Data Availability

The data underlying this study are available in the published article and its Supporting Information.

## Supporting Information Available

Experimental procedures, characterization data, HPLC chromatograms, and NMR spectra reproductions of the synthesized compounds (PDF)

## Dedication

Dedicated to Professor Mieczysław Mąkosza on the occasion of his 90th birthday.
