# Supplementary material for: Enantioselective Organocatalytic Addition of 1,3-Dicarbonyl Compounds to β-Arylvinyl Triflones
Source: Org Lett. 2025 Mar 19;27(12):2879–84. doi: 10.1021/acs.orglett.5c00412 (PMC11959617; doi:10.1021/acs.orglett.5c00412)

Enantioselective Organocatalytic Addition of 1,3-Dicarbonyl Compounds to β-Arylvinyl Triflones

Michał Kopyt,[1,2] Jan Dudziński,[1] Michał Barbasiewicz[1]* and Piotr Kwiatkowski[1,2]*

[1] *University of Warsaw, Faculty of Chemistry, Pasteura 1, 02-093 Warsaw, Poland;*

[2] *University of Warsaw, Biological and Chemical Research Centre, Żwirki i Wigury 101, 02-089 Warsaw, Poland*

Supporting Information

Table of Contents

I. General information 3

II. Preparation of starting materials..4

II.1. Synthesis of methyl triflone..4

II.2. Preliminary condensation reaction with EtOH as a cosolvent 5

II.3. Preliminary condensation reaction with dioxane as a cosolvent 8

II.4. Equilibration of **1a** with aqueous solution of NaOH and EtOH 10

II.5. Preparation of triflones under NaOH-promoted conditions 12

II.6. Analitycal data for triflones **1c**, **1g**, **1i**, **1l** 12

II.7. Preparation of triflones under piperidine-catalyzed conditions 14

II.8. Analitycal data for triflones **1a**, **1b**, **1d**, **1e**, **1f**, **1h**, **1j**, **1k** 14

II.9. Preparation of vinyl triflones under Peterson olefination conditions 18

II.10. Synthesis of nonafluorobutyl sulfone **1r** 20

II.11. 2-Methoxycarbonyl-1-indanone..21

III. Preparation of catalyst **2d** 22

IV. Enantioselective organocatalytic conjugate additions 23

IV.1 Screening of catalysts **2a-j** in model reaction with triflone **1a** 23

IV.2 Optimization studies of model reaction with triflone **1a** 24

IV.3. General procedure for addition of 1,3-dicarbonyl compounds to the vinyl triflones 26

IV.4. Analytical data for adducts **3a-n**..27

IV.5. Analytical data for adducts **4a-d**..41

IV.6. Analytical data for adducts **5a-h**..44

IV.7. Analytical data for adducts **6a-e**..52

V. Other transformations..57

V.1. Hydrolysis-decarboxylation sequence 57

V.2. Direct addition of acetone to triflone **1a**..62

V.3. Cyclization of adduct **3a**..63

V.4. Oxidative cyclization of adduct **3a** 64

V.5. Hydrolysis-decarboxylation of adduct **3a** 66

V.6. Esterification of acid **10** 67

V.7. Synthesis of oxazoles **12** and **13** 68

V.8. DBU-catalyzed racemization of adduct **3a**..70

V.9. Reversible elimination-addition of malonate in compound **3a** in presence of DBU 70

V.10. Competitive experiments of malonate addition to **1a**, **1s** and **1r** 71

VI. Single-crystal XRD analysis data for compound **3b** (CCDC 2373150) 72

VII. Reproductions of ^{1}H, ^{13}C and ^{19}F spectra..78

I. General information

All solvents were commercially-available and used as received unless noted otherwise. Purification of triflone adducts was performed using flash chromatography on silica gel (Silicycle SiliaFlash® P60, 230-400 mesh) with mixtures of hexanes/ethyl acetate, as eluent (typically hexanes to hexanes/ethyl acetate 85:15 gradient elution) unless noted otherwise. Thin-layer chromatography (TLC) was performed on silica gel plates (Merck Kieselgel 60 F_{254}). Visualization of the developed TLC plates was accomplished using UV light or *p*-anisaldehyde stain.

NMR spectra were recorded in $CDCl_3$ or D_2O using Agilent 400 MHz spectrometer. Chemical shifts of ^{1}H NMR and ^{13}C NMR are reported as δ values relative to TMS (δ = 0.00) and $CDCl_3$ (δ = 77.0), respectively. ^{19}F NMR spectra were recorded using hexafluorobenzene, as an internal standard (δ = -161.6 ppm), except for spectra of vinyl triflones, which used trichlorofluoromethane (δ = 0.00). The following abbreviations are used to indicate multiplicity: s - singlet, d - doublet, t - triplet, q - quartet, m - multiplet.

Mass spectra were measured on a Q Exactive unit equipped with Orbitrap mass analyzer using HR ESI technique. Optical rotation was recorded on a Perkin Elmer 241 polarimeter. Enantiomeric ratios were determined using high performance liquid chromatography (HPLC) techniques. HPLC analyses were performed on a Shimadzu LC-20AT chromatograph equipped with the diode-array detector SPD-M20A and Phenomenex Lux® Amylose-1, Amylose-2, Cellulose-1, Cellulose-2, Cellulose-3 or Cellulose-4 (150 mm × 4.6 mm, 3 μm) columns eluted with isopropanol/hexane at 40 °C.

High pressure experiments were performed at room temperature using a direct, single-stage piston-cylinder apparatus with a hydraulic press from Unipress (Warsaw, Poland) equipped with a liquid piston vessel LV/30/16 and laboratory hydraulic press U101. Experiments were conducted in 0.1-0.2 ml, 0.5 ml and 0.9 ml Teflon vials inserted into the high-pressure vessel filled with hexanes as a transmission medium.

II. Preparation of starting materials

II.1. Synthesis of methyl triflone

Methyl triflone was prepared according to the literature: Guangzhou Tinci Materials Technology - Patent CN115557862, **2023**, A

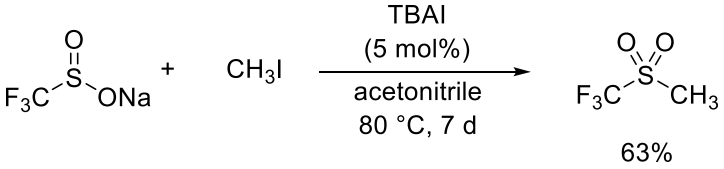

A 100 mL Schlenk tube (with high pressure valve) was charged with sodium trifluoromethanesulfinate (31.22 g, 200.1 mmol, 1.0 equiv), tetrabutylammonium iodide (3.692 g, 5.0 mol%) and flushed with argon. Anhydrous acetonitrile (40 mL) and iodomethane (18.7 mL, 300 mmol, 1.5 equiv) were added. The flask was sealed and the resulting suspension was stirred vigorously (800 rpm) at 80 °C in an oil bath for 7 days (168 h). The suspension was cooled to rt, and then majority of liquid was decanted and concentrated on the rotary evaporator. Precipitate was dissolved in H_2O (30 mL), then brine (20 mL) and concentrated supernatant were added. The mixture was extracted with DCM (3×50 mL). Combined organic layers were washed with saturated aqueous solution of $Na_2S_2O_3$ (50 mL), brine (50 mL) and dried over anhydrous $MgSO_4$. The mixture was filtered, concentrated and distilled under reduced pressure (bp 105-110 °C, p = 450 mbar) to afford methyl triflone (18.73 g, 126.5 mmol, 63%) as a colorless liquid.

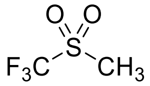

^{1}H NMR (400 MHz, $CDCl_3$): δ 3.13 (q, *J* = 1.0 Hz, 3H).

^{13}C NMR (100 MHz, $CDCl_3$): δ 119.1 (q, *J* = 326 Hz), 36.4 (q, *J* = 1.4 Hz).

^{19}F NMR (376 MHz, $CDCl_3$): δ -80.4 (s).

^{1}H NMR spectrum was consistent with the literature: Chen, Q.-Y.; Yang, G.-Y.; Wu, S.-W. *J. Fluor. Chem.* **1991**, *3*, 291-298.

II.2. Preliminary condensation reaction with EtOH as a cosolvent

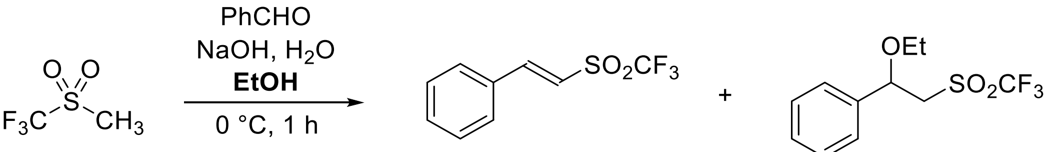

1a, 35% 25%

A 25 mL round-bottom flask was charged with methyl triflone (0.741 g, 5.0 mmol, 1.0 equiv) and flushed with argon. Then, benzaldehyde (0.527 g, 5.0 mmol, 1.0 equiv) and EtOH (1 mL) were added and the flask was cooled in an ice-water bath. Aqueous solution of NaOH (1 mL, 5.0 M, 5.0 mmol, 1.0 equiv) was added dropwise (over ca. 1 min) and the resulting mixture was stirred (600 rpm) at 0 °C. After 1 h the mixture was quenched with aqueous solution of $NaHCO_3$ (14 mL, 5%) and extracted with ethyl acetate (3×15 mL). Combined organic layers were washed with water (15 mL), brine (15 mL) and dried over anhydrous $MgSO_4$. The mixture was filtered, concentrated, and separated with column chromatography (ca. 250 mL of silica gel, eluent: cyclohexane/toluene 10:1 to 1:1) to afford **1a** (0.413 g, 1.75 mmol, 35%) as a white solid, and ethanol adduct (0.356 g, 1.26 mmol, 25%) as a colorless oil.

NMR data of compound **1a** are reported on page S-14.

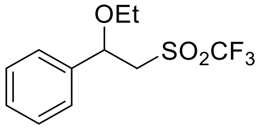

^{1}H NMR (400 MHz, $CDCl_3$): δ 7.47-7.32 (m, 5H), 4.96 (dd, *J* = 10.4, 2.4 Hz, 1H), 3.78 (dd, *J* = 14.9, 10.4 Hz, 1H), 3.45 (qd, *J* = 7.0, 1.2 Hz, 2H), 3.33 (ddd, *J* = 14.9, 2.5, 1.3 Hz, 1H), 1.20 (t, *J* = 7.0 Hz, 3H).

^{13}C NMR (100 MHz, $CDCl_3$): δ 137.9, 129.1, 129.0, 126.3, 119.4 (q, *J* = 327 Hz), 74.9, 64.9, 57.9 (q, *J* = 1.4 Hz), 14.7.

^{19}F NMR (376 MHz, $CDCl_3$): δ -78.3 (s).

MS (EI): (%) 282 (4, [M^+]), 237 (5), 148 (10), 135 (100), 107 (44), 104 (55), 91 (10), 79 (27).

HRMS (EI) m/z: $[M]^+$ Calcd for $C_{11}H_{13}O_3F_3S$: 282.0538, found 282.0543.

^{1}H and ^{19}F NMR spectra of crude reaction mixture after workup (NaOH, H_2O, EtOH conditions)

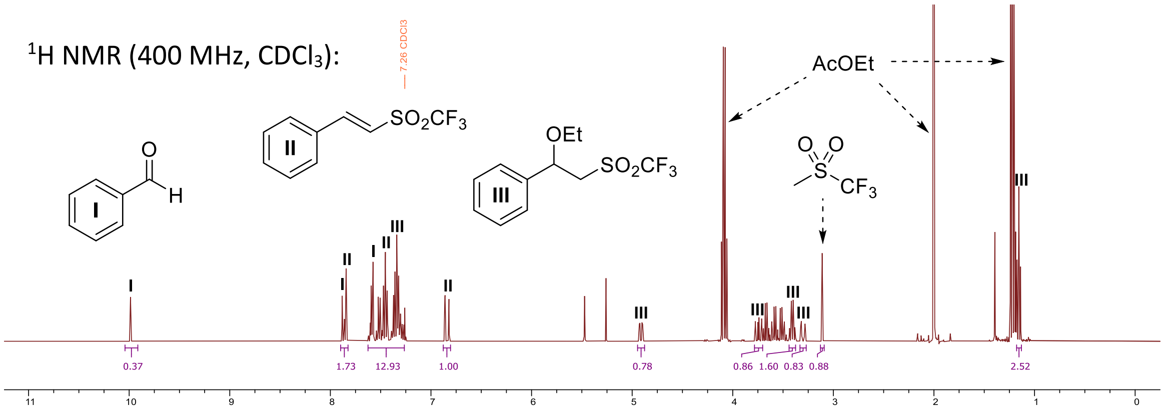

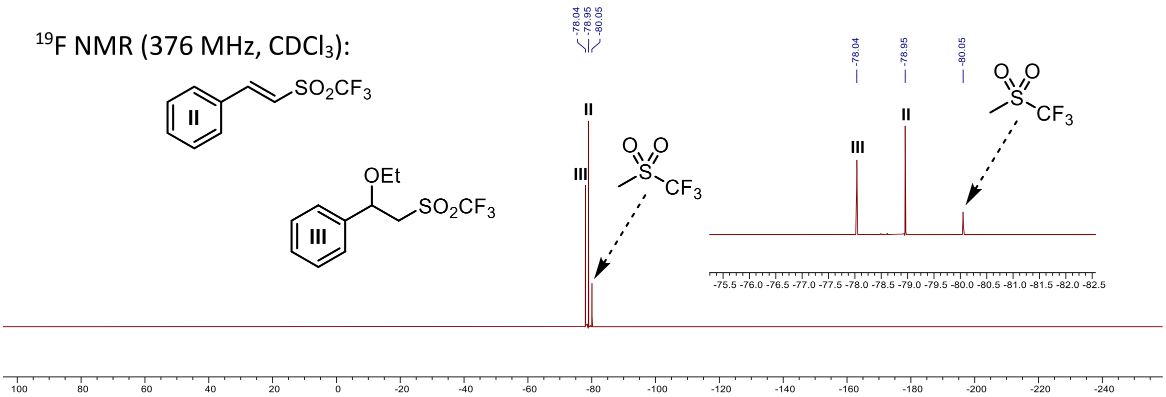

Under the same conditions (NaOH, H_2O, EtOH) mesyl fluoride (CH_3SO_2F) was treated with benzaldehyde. NMR data revealed presence of CH_3SO_2OEt (in organic phase) and $CH_3SO_3^-$ (in aqueous phase).

^1H and ^{19}F NMR spectra of crude organic phase after workup (NaOH, H_2O, EtOH conditions)

^1H NMR (400 MHz, $CDCl_3$):

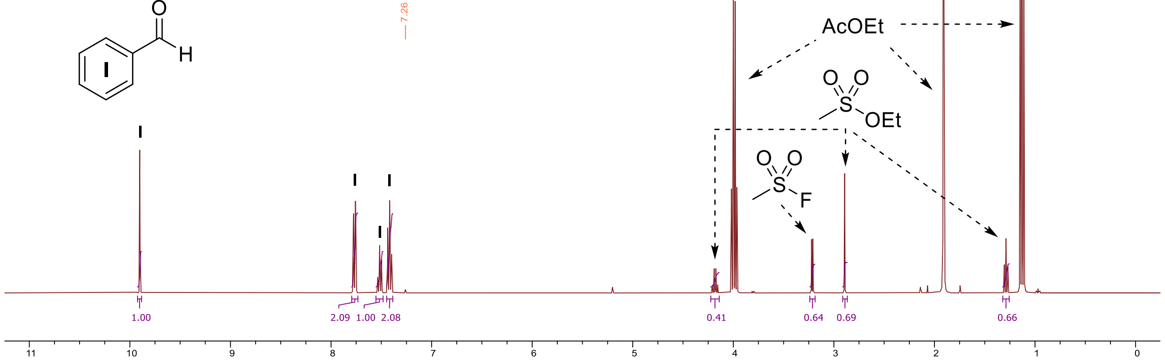

^{19}F NMR (376 MHz, $CDCl_3$):

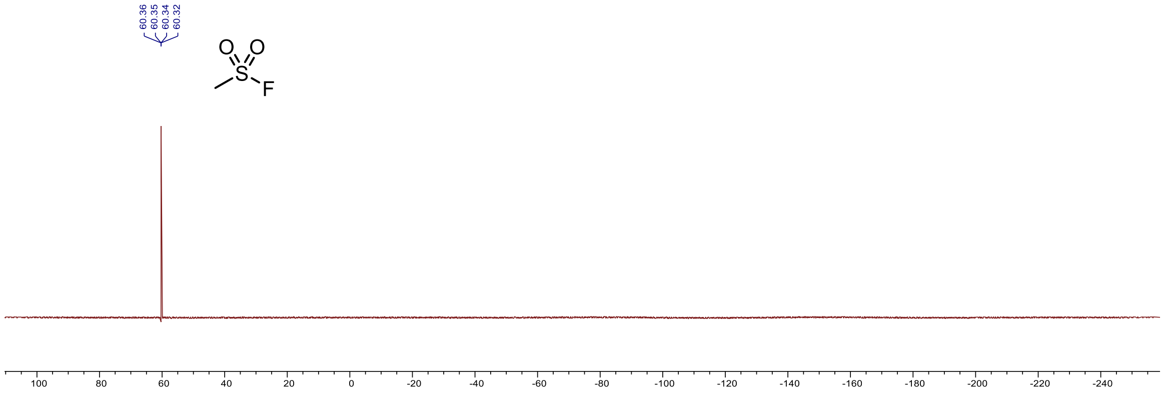

^1H spectrum of crude aqueous phase after workup (NaOH, H_2O, EtOH conditions)

^1H NMR (400 MHz, D_2O):

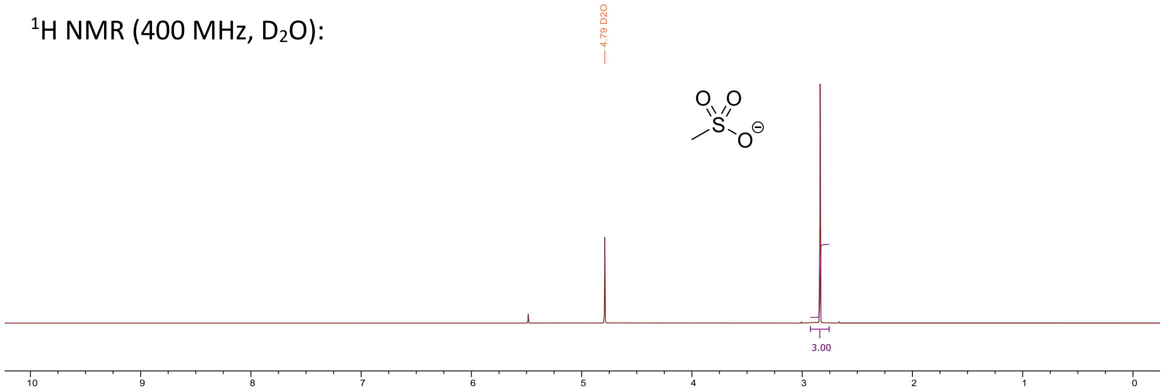

^1H NMR chemical shift (2.84 ppm in D_2O) was consistent with CH_3SO_3Na data from the literature: Noriaki, F.; Seiji, I.; Saburo, N. *Bull. Chem. Soc. Jpn.* **2002**, *4*, 719-723.

II.3. Preliminary condensation reaction with dioxane as a cosolvent

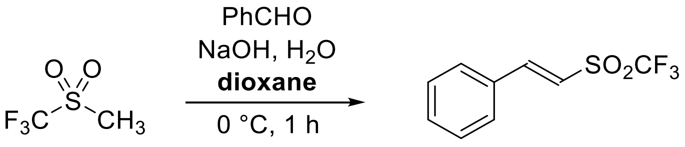

1a, 54%

A 25 mL round-bottom flask was charged with methyl triflone (0.369 g, 2.5 mmol, 1.0 equiv) and flushed with argon. Then, benzaldehyde (0.270 g, 2.5 mmol, 1.0 equiv.) and dioxane (0.5 mL) were added and the flask was cooled in an ice-water bath. Aqueous solution of NaOH (0.5 mL, 5.0 M, 2.5 mmol, 1.0 equiv.) was added dropwise (over ca. 1 min) and the resulting mixture was stirred (1000 rpm) at 0 °C for 1 h. Then, the mixture was warmed to rt and after 1 h it was quenched with aqueous solution of $NaHCO_3$ (14.5 mL, 5%) and extracted with ethyl acetate (3×15 mL). Combined organic layers were washed with water (15 mL), brine (15 mL) and dried over anhydrous $MgSO_4$. The mixture was filtered, concentrated, and separated with column chromatography (ca. 150 mL of silica gel, eluent: cyclohexane/toluene 6:1 to 1:1) to afford **1a** (0.316 g, 1.34 mmol, 54%) as a white solid.

^1H and ^{19}F NMR spectra of crude reaction mixture after workup (NaOH, H_2O, dioxane conditions)

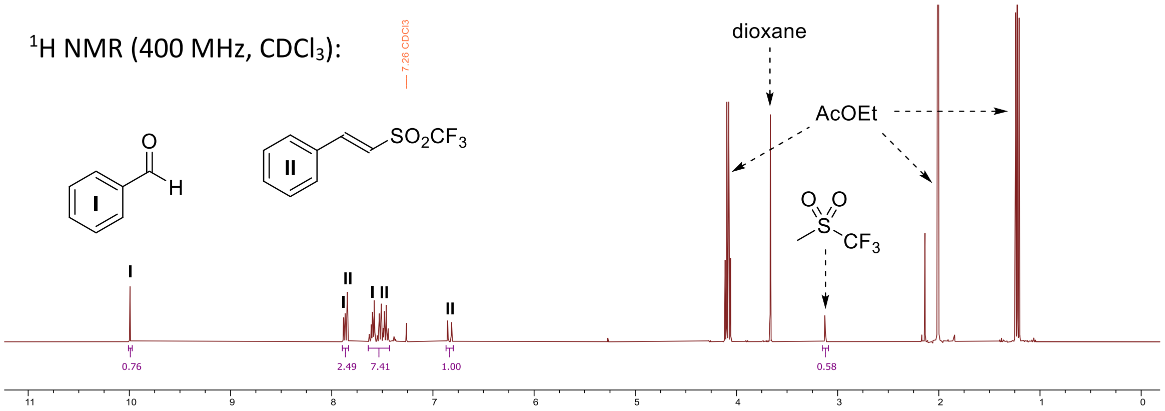

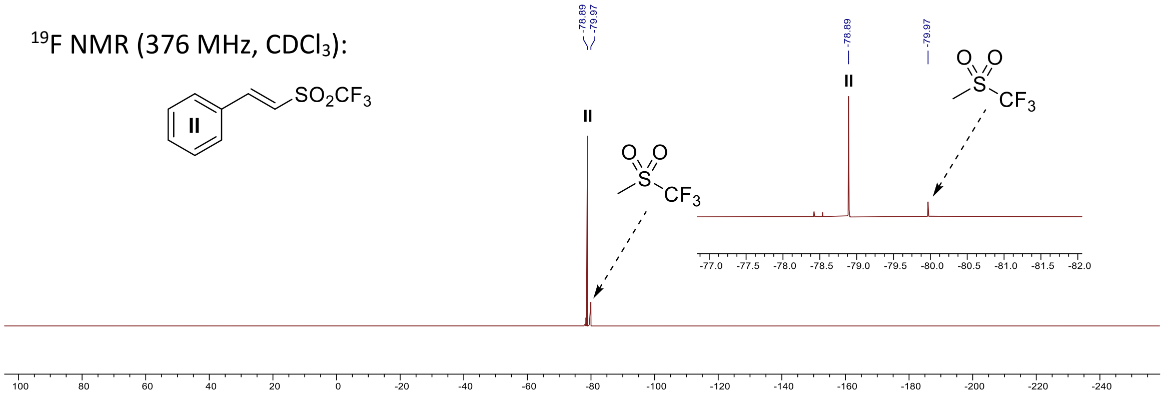

Under the same conditions (NaOH, H_2O, dioxane) mesyl fluoride (CH_3SO_2F) was treated with benzaldehyde. NMR data revealed presence of substrates (in organic phase) and $CH_3SO_3^-$ (in aqueous phase).

^1H and ^{19}F NMR spectra of crude organic phase after workup (NaOH, H_2O, dioxane conditions)

^1H NMR (400 MHz, $CDCl_3$):

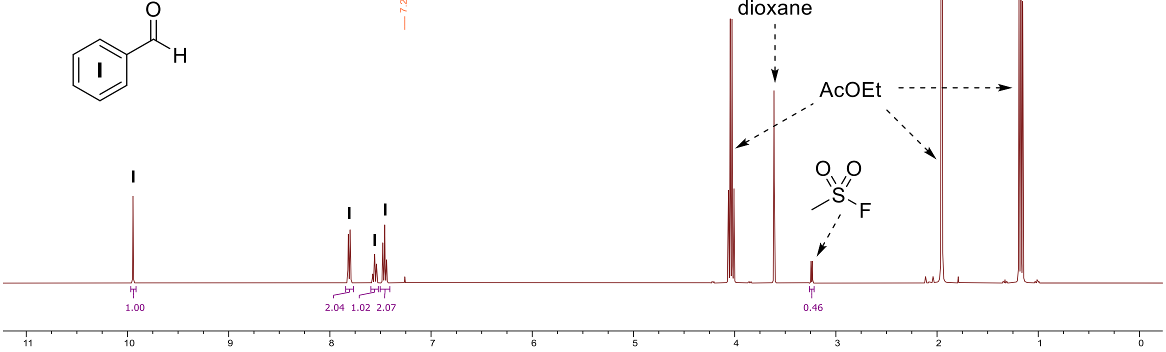

^{19}F NMR (376MHz, $CDCl_3$):

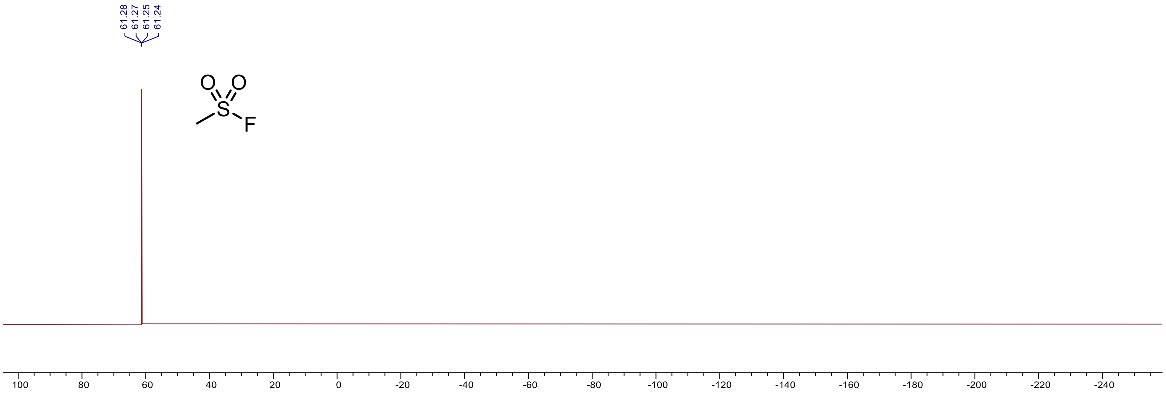

^1H spectrum of crude aqueous phase after workup (NaOH, H_2O, dioxane conditions)

^1H NMR (400 MHz, D_2O):

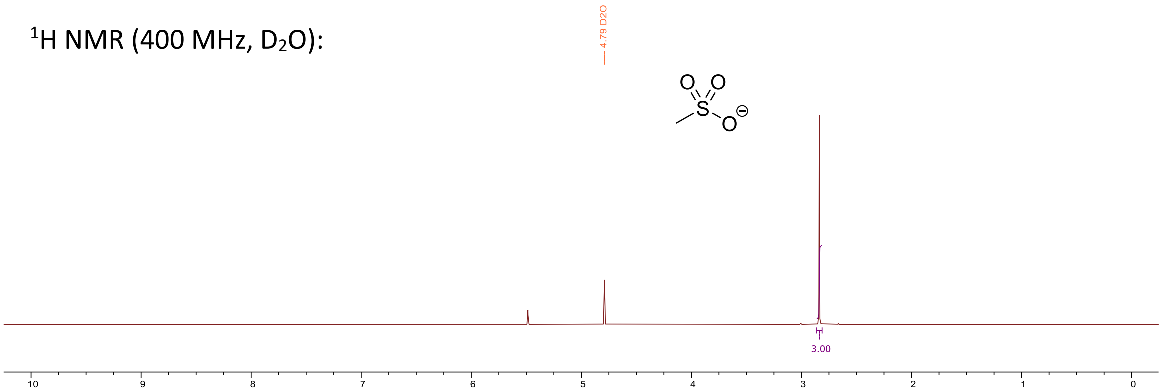

^1H NMR chemical shift (2.84 ppm in D_2O) was consistent with CH_3SO_3Na data in the literature: Noriaki, F.; Seiji, I.; Saburo, N. *Bull. Chem. Soc. Jpn.* **2002**, *4*, 719-723.

II.4. Equilibration of 1a with aqueous solution of NaOH and EtOH

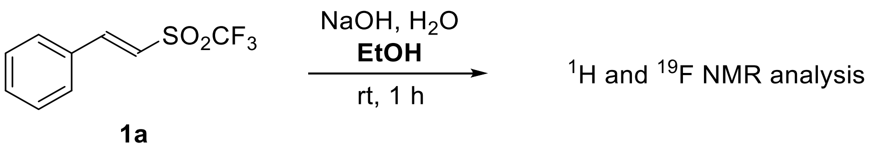

Treatment of **1a** with aqueous solution of NaOH with EtOH and analysis of crude reaction mixture with NMR revealed presence of methyl triflone, benzaldehyde, and ethanol adduct.

^{1}H and ^{19}F NMR spectra of crude reaction mixture after workup (NaOH, H_2O, EtOH conditions)

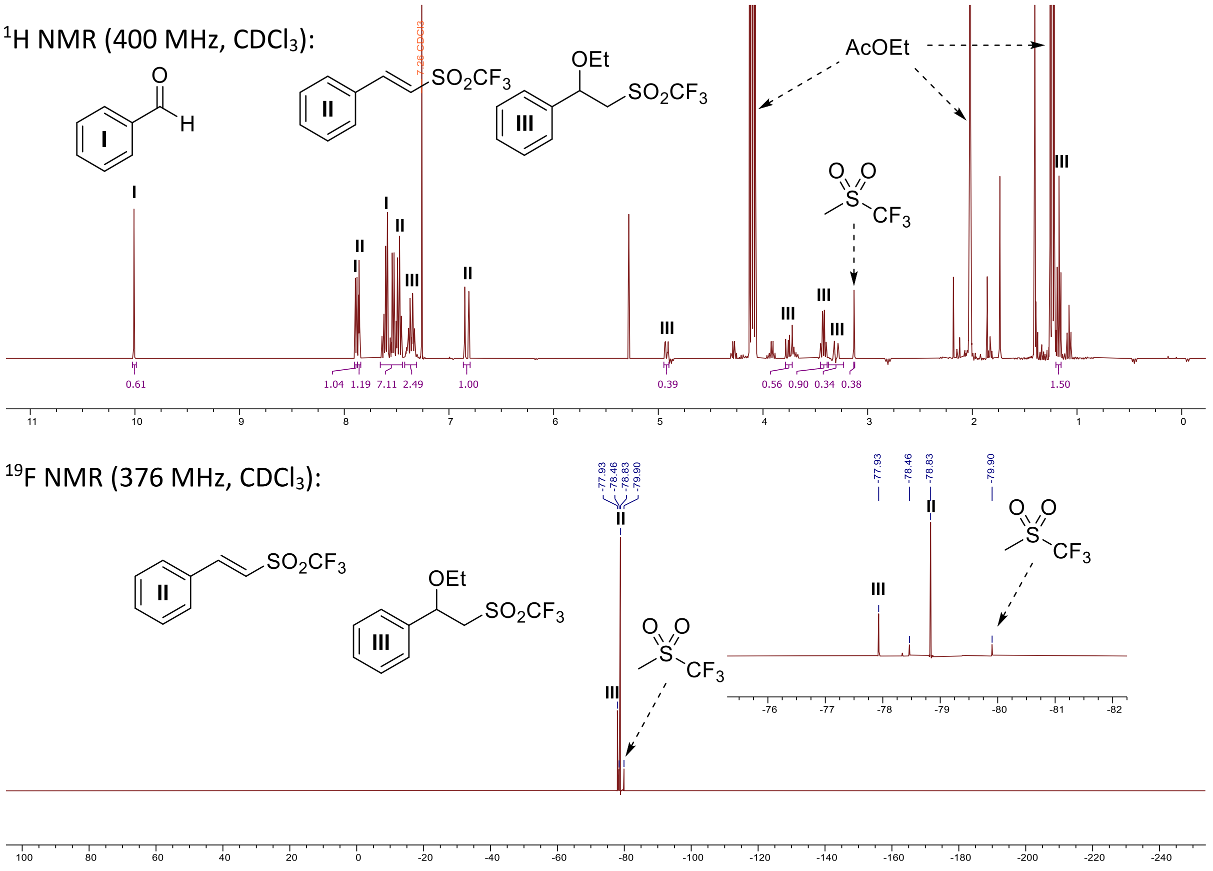

Under the same conditions (NaOH, H_2O, EtOH) <u>(*E*)-2-phenylethenesulfonyl fluoride</u> (PhCH=$CHSO_2F$) was tested. Product of substitution of the sulfonyl group with EtOH was isolated in 83% of yield.

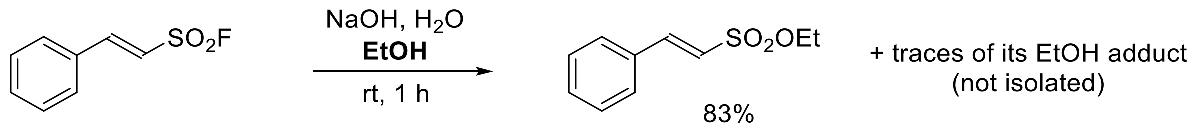

A 25 mL round-bottom flask was charged with (*E*)-2-phenylethenesulfonyl fluoride (0.186 g, 1.0 mmol) and EtOH (1 mL) was added. Then aqueous solution of NaOH (1 mL, 1.0 M, 1.0 mmol, 1.0 equiv) was added dropwise (ca. 1 min) and the resulting mixture was stirred (600 rpm) at rt. After 1 h the mixture was quenched with aqueous solution of $NaHCO_3$ (14 mL, 5%) and extracted with ethyl acetate (3×15 mL). Combined organic layers were washed with water (15 mL), brine (15 mL) and dried over anhydrous $MgSO_4$. The mixture was filtered, concentrated, and separated with column chromatography (ca. 75 mL of silica gel, eluent: cyclohexane/ethyl acetate 10:1 to 6:1) to afford ethyl (*E*)-2-phenylethenesulfonate (0.176 g, 0.83 mmol, 83%) as a white solid.

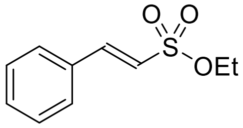

mp = 44.5-46.0 °C

^{1}H NMR (400 MHz, $CDCl_3$): δ 7.60 (d, *J* = 15.6 Hz, 1H), 7.54-7.49 (m, 2H), 7.48-7.38 (m, 3H), 6.75 (d, *J* = 15.6 Hz, 1H), 4.23 (q, *J* = 7.2 Hz, 2H), 1.40 (t, *J* = 7.1 Hz, 3H).

^{13}C NMR (100 MHz, $CDCl_3$): δ 144.6, 131.9, 131.4, 129.1, 128.4, 121.2, 66.8, 14.8.

^{1}H and ^{13}C NMR spectra (Metaferia, B. B.; Fetterolf, B. J.; Shazad-ul-Hussan, S.; Moravec, M.; Smith, J. A.; Ray, S.; Gutierrez-Lugo, M.-T.; Bewley, C. A. *J. Med. Chem.* **2007**, *50*, 6326-6336) and melting point (Hartig, S. *J. Prakt. Chem.* **1966**, *4*, 215-224) were consistent with the literature.

The same reaction tested with dioxane as a cosolvent gave unreacted sulfonyl fluoride (recovered in 45%) and the corresponding sulfonate anion (PhCH=$CHSO_3^-$) detected in aqueous phase by NMR.

NMR (D_2O) spectra of the aqueous phase after the reaction in dioxane

^{1}H NMR (400 MHz, D_2O):

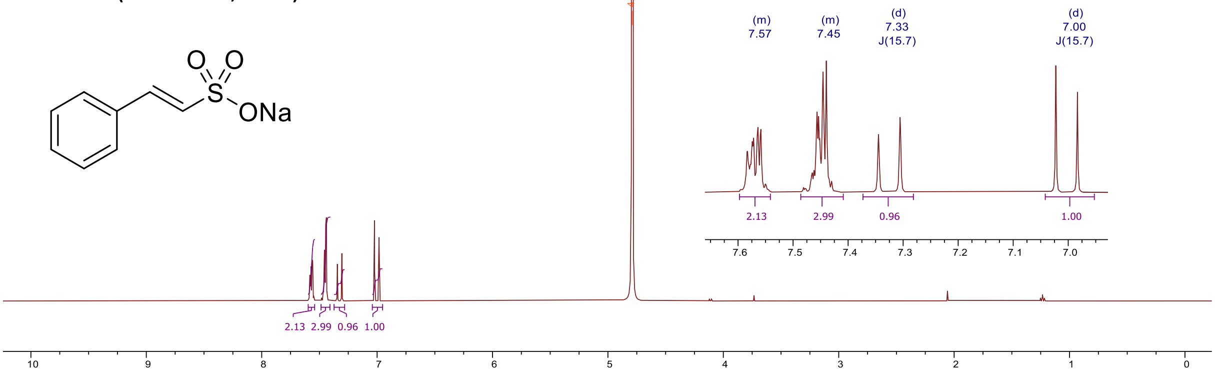

^{1}H NMR spectrum was consistent with the literature: Zhang, H.; Wang, M.; Jiang, X. *Green Chem.* **2020**, *22*, 8238-8242.

II.5. Preparation of triflones under NaOH-promoted conditions

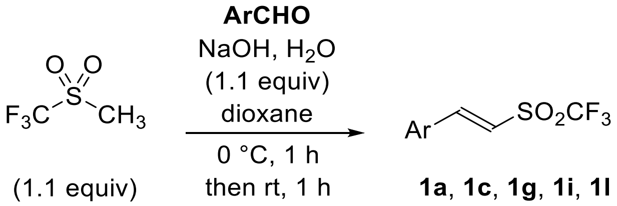

A 25 mL round-bottom flask was charged with methyl triflone (1.629 g, 11 mmol, 1.1 equiv), aldehyde (10 mmol, 1.0 equiv) and flushed with argon. Then, dioxane (1 mL) was added and the flask was cooled in an ice-water bath. Aqueous solution of NaOH (1.1 mL, 10 M, 11 mmol, 1.1 equiv) was added dropwise (over ca. 2 min) and the resulting mixture was stirred (1000 rpm) at 0 °C. After 1 h the mixture was warmed to rt and after next 1 h it was poured onto aqueous solution of HCl (20 mL, ca. 3.5%). Then H_2O (30 mL) was added, and the mixture was extracted with ethyl acetate (3×50 mL). Combined organic layers were washed with brine (50 mL) and dried over anhydrous $MgSO_4$. The mixture was filtered, concentrated, and separated with column chromatography (ca. 300 mL of silica gel).

Under these conditions 3-pyridinecarboxaldehyde gave mostly 3-hydroxymethylpyridine: a product of the Cannizzaro reaction, as confirmed by ^{1}H NMR (chemical shifts of 3-hydroxymethylpyridine were consistent with the literature: S. Yadav, M. Rao Kuram, *Eur. J. Org. Chem.* **2023**, *26*, e202201344).

Spectroscopic data of compound **1a** are reported on page S-14.

II.6. Analitycal data for triflones 1c, 1g, 1i, 1l

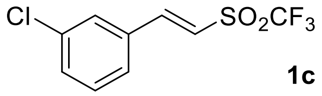

1c

Yield: 43% (1.174 g, 4.34 mmol) from 3-chlorobenzaldehyde (1.404 g, 9.99 mmol), eluent: cyclohexane/toluene 6:1 to 1:1, yellowish solid.

mp = 77.0-79.0 °C.

^{1}H NMR (400 MHz, $CDCl_3$): δ 7.83 (d, *J* = 15.5 Hz, 1H), 7.59 (t, *J* = 1.9 Hz, 1H), 7.54-7.41 (m, 3H), 6.86 (dq, *J* = 15.5, 0.9 Hz, 1H).

^{13}C NMR (100 MHz, $CDCl_3$): δ 152.0, 135.6, 133.0, 132.8, 130.7, 129.0, 127.7, 119.6 (q, *J* = 325 Hz), 118.4.

^{19}F NMR (376 MHz, $CDCl_3$): δ -79.1 (s).

HRMS (ESI) m/z: $[M-H]^-$ Calcd for $C_9H_5ClF_3O_2S$: 268.9645; Found: 268.9654.

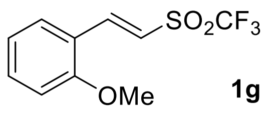

1g

Yield: 79% (2.093 g, 7.86 mmol) from 2-methoxybenzaldehyde (1.357 g, 9.98 mmol), eluent: cyclohexane/toluene 6:1 to 1:3, white solid.

mp = 47.5-48.5 °C.

^{1}H NMR (400 MHz, $CDCl_3$): δ 8.00 (d, *J* = 15.6 Hz, 1H), 7.55-7.44 (m, 2H), 7.10 (dq, *J* = 15.6, 0.9 Hz, 1H), 7.06-6.98 (m, 2H), 3.94 (s, 3H).

^{13}C NMR (100 MHz, $CDCl_3$): δ 159.7, 149.7, 134.7, 132.4, 121.0, 120.0, 119.8 (q, *J* = 325 Hz), 116.9, 111.5, 55.6.

^{19}F NMR (376 MHz, $CDCl_3$): δ -79.4 (s).

HRMS (ESI) m/z: $[M+H]^+$ Calcd for $C_{10}H_{10}F_3O_3S$: 267.0297; Found: 267.0295.

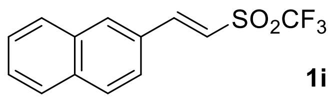

1i

Yield: 69% (1.988 g, 6.94 mmol) from 2-naphthaldehyde (1.566 g, 10.03 mmol), eluent: cyclohexane/toluene 6:1 to 1:1, white solid.

mp = 104.5-106.5 °C.

^{1}H NMR (400 MHz, $CDCl_3$): δ 8.07-8.00 (m, 2H), 7.94-7.85 (m, 3H), 7.66-7.55 (m, 3H), 6.93 (d, *J* = 15.4 Hz, 1H).

^{13}C NMR (100 MHz, $CDCl_3$): δ 153.8, 135.3, 133.2, 132.9, 129.4, 129.1, 128.9, 128.6, 127.9, 127.4, 123.1, 119.7 (q, *J* = 325 Hz), 116.2.

^{19}F NMR (376 MHz, $CDCl_3$): δ -79.2 (s).

^{1}H, ^{13}C and ^{19}F NMR spectra were consistent with the literature: Alkan-Zambada, M.; Hu, X. *Organometallics* **2018**, *21*, 3928-3935.

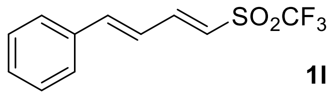

1l

Yield: 50% (0.666 g, 2.54 mmol) from cinnamaldehyde (0.665 g, 5.03 mmol), eluent: cyclohexane/toluene 10:1 to 1:2, white solid.

mp = 78.5-80.5 °C.

^{1}H NMR (400 MHz, $CDCl_3$): δ 7.64 (dd, *J* = 14.8, 11.2 Hz, 1H), 7.57-7.49 (m, 2H), 7.49-7.39 (m, 3H), 7.15 (d, *J* = 15.5 Hz, 1H), 6.93 (dd, *J* = 15.5, 11.2 Hz, 1H), 6.40 (d, *J* = 14.7 Hz, 1H).

^{13}C NMR (100 MHz, $CDCl_3$): δ 153.5, 147.8, 134.4, 130.8, 129.0, 128.1, 122.6, 119.6 (q, *J* = 325 Hz), 117.5.

^{19}F NMR (376 MHz, $CDCl_3$): δ -79.5 (s).

HRMS (ESI) m/z: $[M+H]^+$ Calcd for $C_{11}H_{10}F_3O_2S$: 263.0348; Found: 263.0347.

II.7. Preparation of triflones under piperidine-catalyzed conditions

The reaction was carried out according to the procedure for condensation of nitroalkanes: Fioravanti, S.; Pellacani, L.; Tardella, P. A.; Vergari, M. C. *Org. Lett.* **2008**, *10*, 1449-1451.

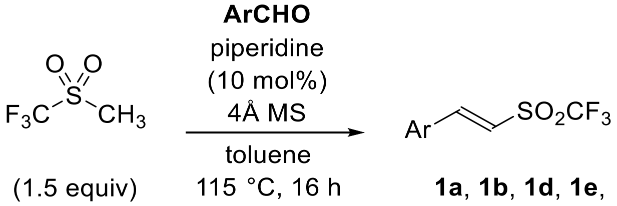

1a, **1b**, **1d**, **1e**, **1f**, **1h**, **1j**, **1k**

A 25 mL Schlenk tube (with high pressure valve) was charged with aldehyde (3.0 mmol), methyl triflone (0.667 g, 4.5 mmol, 1.5 equiv) and flushed with argon. Then, anhydrous toluene (5 mL) and freshly dried 4Å molecular sieves (3 g) were added, followed by the addition of a solution of piperidine (0.026 g, 0.3 mmol, 10 mol%) in anhydrous toluene (1 mL). The flask was sealed, and the mixture was stirred at 115 °C in an oil bath for 16 h. Then, the mixture was cooled to rt and filtered through silica plug (ϕ = 4 cm, d = 2 cm), followed by washing with dichloromethane (200 mL). After evaporation of the solvents analytically pure vinyl triflones were obtained, unless stated otherwise.

II.8. Analitycal data for triflones 1a, 1b, 1d, 1e, 1f, 1h, 1j, 1k

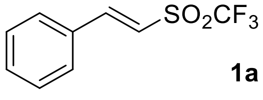

1a

Yield: 96% (4.527 g, 19.16 mmol) from benzaldehyde (2.129 g, 20.06 mmol), using 1.1 equiv. of methyl triflone, 20 g of 4Å MS and 40 mL of toluene; white solid.

mp = 58.0-59.0 °C.

^{1}H NMR (400 MHz, $CDCl_3$): δ 7.89 (d, *J* = 15.5 Hz, 1H), 7.64-7.46 (m, 5H), 6.84 (d, *J* = 15.5 Hz, 1H).

^{13}C NMR (100 MHz, $CDCl_3$): δ 153.8, 133.2, 131.2, 129.53, 129.46, 119.7 (q, *J* = 325 Hz), 116.6.

^{19}F NMR (376 MHz, $CDCl_3$): δ -79.3 (s).

^{1}H, ^{13}C and ^{19}F NMR spectra were consistent with the literature: Alkan-Zambada, M.; Hu, X. *Organometallics* **2018**, *21*, 3928-3935.

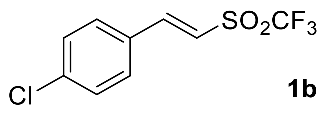

1b

Yield: 98% (0.806 g, 2.98 mmol) from 4-chlorobenzaldehyde (0.426 g, 3.03 mmol), yellowish solid.

mp = 101.0-103.0 °C.

^{1}H NMR (400 MHz, $CDCl_3$): δ 7.84 (d, *J* = 15.5 Hz, 1H), 7.57-7.52 (m, 2H), 7.49-7.44 (m, 2H), 6.82 (d, *J* = 15.5 Hz, 1H).

^{13}C NMR (100 MHz, $CDCl_3$): δ 152.3, 139.5, 130.7, 129.8, 129.5, 119.6 (q, *J* = 325 Hz), 117.1.

^{19}F NMR (376 MHz, $CDCl_3$): δ -79.1 (s).

^{1}H and ^{19}F NMR spectra were consistent with the literature: Alkan-Zambada, M.; Hu, X. *Organometallics* **2018**, *21*, 3928-3935.

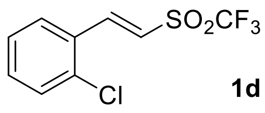

1d

Yield: 90% (0.737 g, 2.73 mmol) from 2-chlorobenzaldehyde (0.425 g, 3.03 mmol) and using molecular sieves 4Å (6 g), white solid.

mp = 51.5-53.5 °C.

^{1}H NMR (400 MHz, $CDCl_3$): δ 8.32 (d, *J* = 15.6 Hz, 1H), 7.65 (dd, *J* = 7.8, 1.6 Hz, 1H), 7.55-7.44 (m, 2H), 7.43-7.34 (m, 1H), 6.91 (d, *J* = 15.6 Hz, 1H).

^{13}C NMR (100 MHz, $CDCl_3$): δ 149.4, 136.2, 133.8, 130.7, 129.2, 128.9, 127.6, 119.5 (q, *J* = 325 Hz), 119.2.

^{19}F NMR (376 MHz, $CDCl_3$): δ -79.0 (s).

HRMS (ESI) m/z: $[M-H]^-$ Calcd for $C_9H_5ClF_3O_2S$: 268.9645; Found: 268.9655.

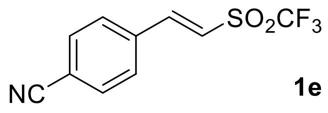

1e

Yield: 43% (0.342 g, 1.31 mmol) from 4-formylbenzonitrile (0.396 g, 3.02 mmol) and using molecular sieves 4Å (6 g), isolated with column chromatography, eluent: cyclohexane, then cyclohexane/ethyl acetate 10:1 to 2:1, white solid.

mp = 166.5-168.5 °C.

^{1}H NMR (400 MHz, $CDCl_3$): δ 7.90 (d, *J* = 15.6 Hz, 1H), 7.82-7.77 (m, 2H), 7.75-7.69 (m, 2H), 6.96 (d, *J* = 15.6 Hz, 1H).

^{13}C NMR (100 MHz, $CDCl_3$): δ 150.9, 134.9, 133.1, 129.7, 120.7, 119.5 (q, *J* = 325 Hz), 117.5, 116.2.

^{19}F NMR (376 MHz, $CDCl_3$): δ -78.8 (s).

^{1}H, ^{13}C and ^{19}F NMR spectra and melting point were consistent with the literature: Engl, S.; Reiser, O. *Eur. J. Org. Chem.* **2020**, 1523-1533.

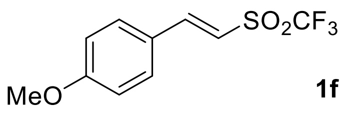

1f

Yield: 94% (0.758 g, 2.85 mmol) from 4-methoxybenzaldehyde (0.411 g, 3.02 mmol) and using molecular sieves 4Å (6 g), white solid.

mp = 81.0-82.5 °C.

^{1}H NMR (400 MHz, $CDCl_3$): δ 7.80 (d, *J* = 15.4 Hz, 1H), 7.55 (d, *J* = 8.9 Hz, 2H), 6.97 (d, *J* = 8.8 Hz, 2H), 6.66 (d, *J* = 15.4 Hz, 1H), 3.86 (s, 3H).

^{13}C NMR (100 MHz, $CDCl_3$): δ 163.7, 153.5, 131.7, 123.7, 119.7 (q, *J* = 325 Hz), 114.8, 112.6, 55.4.

^{19}F NMR (376 MHz, $CDCl_3$): δ -79.5 (s).

HRMS (ESI) m/z: $[M+H]^+$ Calcd for $C_{10}H_{10}F_3O_3S$: 267.0297; Found: 267.0296.

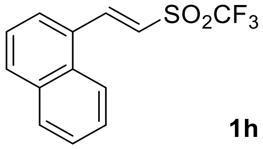

1h

Yield: 91% (0.782 g, 2.73 mmol) from 1-naphthaldehyde (0.469 g, 3.00 mmol), yellowish solid.

mp = 110.5-111.5 °C.

^{1}H NMR (400 MHz, $CDCl_3$): δ 8.74 (d, *J* = 15.3 Hz, 1H), 8.12 (d, *J* = 8.4 Hz, 1H), 8.05 (d, *J* = 8.2 Hz, 1H), 7.93 (dd, *J* = 8.0, 1.5 Hz, 1H), 7.83 (dd, *J* = 7.3, 1.2 Hz, 1H), 7.69-7.51 (m, 3H), 6.97 (d, *J* = 15.3 Hz, 1H).

^{13}C NMR (100 MHz, $CDCl_3$): δ 150.7, 133.5, 131.1, 129.0, 128.0, 127.9, 126.9, 126.8, 125.2, 122.3, 119.7 (q, *J* = 325 Hz), 118.3.

^{19}F NMR (376 MHz, $CDCl_3$): δ -79.1 (s).

^{1}H and ^{19}F NMR spectra were consistent with the literature: Alkan-Zambada, M.; Hu, X. *Organometallics* **2018**, *21*, 3928-3935.

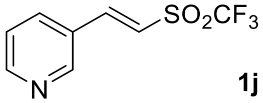

1j

Yield: 88% (0.624 g, 2.63 mmol) from 3-pyridinecarboxaldehyde (0.319 g, 2.98 mmol) and silica plug was additionally washed with ethyl acetate (200 mL), white solid.

mp = 87.5-89.0 °C.

^{1}H NMR (400 MHz, $CDCl_3$): δ 8.84 (d, *J* = 2.3 Hz, 1H), 8.76 (dd, *J* = 4.9, 1.6 Hz, 1H), 7.95-7.88 (m, 2H), 7.45 (dd, *J* = 8.0, 4.8 Hz, 1H), 6.95 (d, *J* = 15.7 Hz, 1H).

^{13}C NMR (100 MHz, $CDCl_3$): δ 153.3, 150.7, 150.3, 135.5, 127.0, 124.0, 119.4 (q, *J* = 325 Hz), 118.8.

^{19}F NMR (376 MHz, $CDCl_3$): δ -78.9 (s).

HRMS (ESI) m/z: $[M+H]^+$ Calcd for $C_8H_7F_3NO_2S$: 238.0144; Found: 238.0143.

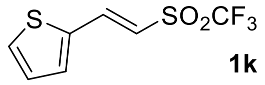

1k

Yield: 93% (0.679 g, 2.80 mmol) from 2-thiophenecarboxaldehyde (0.337 g, 3.0 mmol), brown solid.

mp = 84.5-87.5 °C.

^{1}H NMR (400 MHz, $CDCl_3$): δ 7.97 (d, *J* = 15.1 Hz, 1H), 7.66 (d, *J* = 5.1 Hz, 1H), 7.51 (d, *J* = 3.7 Hz, 1H), 7.18 (dd, *J* = 5.0, 3.8 Hz, 1H), 6.56 (d, *J* = 15.1 Hz, 1H).

^{13}C NMR (100 MHz, $CDCl_3$): δ 145.8, 135.69, 135.65, 133.4, 129.0, 119.6 (q, *J* = 325 Hz), 113.3.

^{19}F NMR (376 MHz, $CDCl_3$): δ -79.3 (s).

Melting point was consistent with the literature: Yagupolskii, L. M.; Panteleimonv, A. G. *J. General Chem. USSR* **1966**, *36*, 433-437 (*Zh. Obshch. Khim.* **1966**, *36*, 416-421).

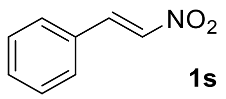

1s

Yield: 80% (0.357 g, 2.39 mmol) from benzaldehyde (0.317 g, 2.99 mmol) and using $MeNO_2$, pale brown solid.

mp = 55.5-56.5 °C.

^{1}H NMR (400 MHz, $CDCl_3$): δ 7.99 (d, *J* = 13.7 Hz, 1H), 7.58 (d, *J* = 13.7 Hz, 1H), 7.56-7.42 (m, 5H).

^{13}C NMR (100 MHz, $CDCl_3$): δ 138.9, 137.0, 132.0, 129.9, 129.3, 129.0.

^{1}H and ^{13}C NMR spectra were consistent with the literature: Zhou, L.; Huang, R.; Lu, S.; Liu, B.; Gao, M.; Xu, B. *Org. Lett.* **2023**, *25*, 1415-1419.

II.9. Preparation of vinyl triflones under Peterson olefination conditions

Synthesis of **1m** was based on procedure described in the literature: Mahadevan, A.; Fuchs, P. L. *Tetrahedron Lett.* **1994**, *35*, 6025-6028.

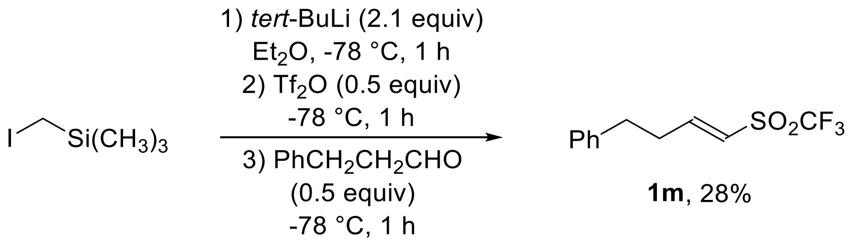

A 100 mL Schlenk flask was charged with (iodomethyl)trimethylsilane (2.149 g, 10.04 mmol, 1.0 equiv) and flushed with argon. Anhydrous Et_2O (30 mL) was added and the flask was placed in acetone bath (-78 °C). Then, *tert*-butyllithium (13.2 mL, 21.1 mmol, 1.6 M solution in pentane, 2.1 equiv) was added dropwise over 5 min. After 1 h triflic anhydride (0.85 mL, 5.05 mmol, 0.5 equiv) was added and after another 1 h solution of hydrocinnamaldehyde (0.667 g, 4.97 mmol, 0.5 equiv) in anhydrous Et_2O (10 mL) was added and the mixture was stirred for another 1 h at -78 °C. Then, cooling bath was removed, after 5 min the mixture was poured onto aqueous solution of $NaHCO_3$ (100 mL, 5%), and extracted with ethyl acetate (3×100 mL). Combined organic layers were washed with water (100 mL), brine (100 mL) and dried over anhydrous $MgSO_4$. The mixture was filtered, concentrated, and separated with column chromatography (ϕ = 3 cm, d = 40 cm, ca. 300 mL of silica gel, eluent: cyclohexane, then cyclohexane/toluene 20:1 to 1:1) to afford **1m** (0.361 g, 1.37 mmol, 28%) as a pale yellow oil.

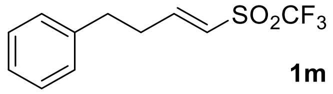

1m

^{1}H NMR (400 MHz, $CDCl_3$): δ 7.40-7.32 (m, 3H), 7.31-7.25 (m, 1H), 7.20 (dd, *J* = 7.2, 1.8 Hz, 2H), 6.33 (d, *J* = 15.2 Hz, 1H), 2.88 (t, *J* = 7.5 Hz, 2H), 2.78-2.70 (m, 2H).

^{13}C NMR (100 MHz, $CDCl_3$): δ 159.2, 139.0, 128.6, 128.2, 126.6, 121.7, 119.5 (q, *J* = 325 Hz), 33.9, 33.2.

^{19}F NMR (376 MHz, $CDCl_3$): δ -79.5 (s).

HRMS (ESI) m/z: $[M-H]^-$ Calcd for $C_{11}H_{10}F_3O_2S$: 263.0348; Found: 263.0357.

Synthesis of **1n** was based on procedure described in the literature: Mahadevan, A.; Fuchs, P. L. *Tetrahedron Lett.* **1994**, *35*, 6025-6028.

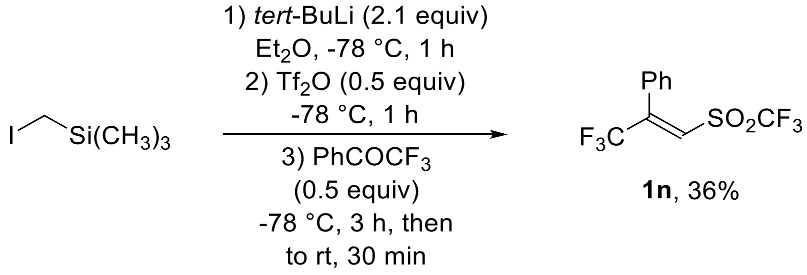

A 100 mL Schlenk flask was charged with (iodomethyl)trimethylsilane (4.291 g, 20.04 mmol, 1.0 equiv) and flushed with argon. Anhydrous Et_2O (30 mL) was added and the flask was placed in acetone bath (-78 °C). Then, *tert*-butyllithium (26.3 mL, 42.0 mmol, 1.6 M solution in pentane, 2.1 equiv) was added dropwise over 10 min. After 1 h triflic anhydride (1.70 mL, 10.1 mmol, 0.5 equiv) was added and after another 1 h solution of trifluoroacetophenone (1.745 g, 10.02 mmol, 0.5 equiv) in anhydrous Et_2O (10 mL) was added and the mixture was stirred for another 3 h at -78 °C. Then, cooling bath was removed and after 30 min the mixture was poured onto aqueous solution of $NaHCO_3$ (100 mL, 5%) and extracted with ethyl acetate (3×100 mL). Combined organic layers were washed with water (100 mL), brine (100 mL) and dried over anhydrous $MgSO_4$. The mixture was filtered, concentrated, and separated with column chromatography (ϕ = 6 cm, d = 25 cm, ca. 350 mL of silica gel, eluent: cyclohexane/toluene 10:1 to 1:2) to afford **1n** (1.091 g, 3.59 mmol, 36%) as a white solid.

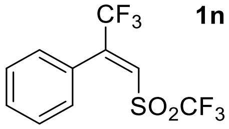

1n

mp = 54.5-56.0 °C.

^{1}H NMR (400 MHz, $CDCl_3$): δ 7.59-7.53 (m, 1H), 7.52-7.46 (m, 2H), 7.39 (d, *J* = 7.1 Hz, 2H), 7.11 (s, 1H).

^{13}C NMR (100 MHz, $CDCl_3$): δ 150.8 (q, *J* = 32.6 Hz), 131.1, 128.7, 128.5, 126.6, 125.49-125.14 (m), 120.9 (q, *J* = 278 Hz), 119.1 (q, *J* = 326 Hz).

^{19}F NMR (376 MHz, $CDCl_3$): δ -68.7, -78.6 (s).

HRMS (ESI) m/z: $[M-H]^-$ Calcd for $C_{10}H_5F_6O_2S$: 302.9909; Found: 302.9919.

Compounds **1o**, **1p** and **1q** were prepared according to the literature procedures:

1o: a) Bosset, C.; Lefebvre, G.; Angibaud, P.; Stansfield, I.; Meerpoel, L.; Berthelot, D.; Guérinot, A.; Cossy, J. *J. Org. Chem.* **2017**, *82*, 4020-4036. b) Benedetti, F.; Fabrissin, S.; Risaliti, A. *Tetrahedron* **1983**, *39*, 3887-3893.

1p: Noble, A.; MacMillan, D. W. C. *J. Am. Chem. Soc.* **2014**, *136*, 11602-11605.

1q: Tryniszewski, M.; Basiak, D.; Barbasiewicz, M. *Org. Lett.* **2022**, *24*, 4270-4274.

II.10. Synthesis of nonafluorobutyl sulfone 1r

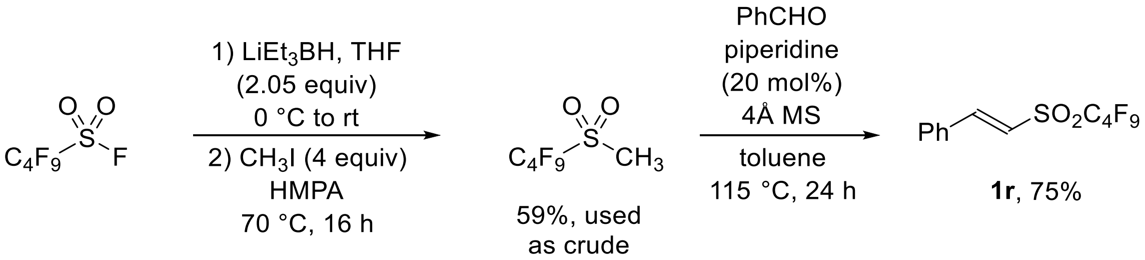

A 50 mL Schlenk tube (with high pressure valve) was charged with nonafluorobutanesulfonyl fluoride (1.810 g, 5.99 mmol, 1.0 equiv) and flushed with argon. Anhydrous THF (8 mL) was added and the flask was placed in an ice-water bath. Then, lithium triethylborohydride (12.3 mL, 12.3 mmol, 1.0 M solution in THF, 2.05 equiv) was added dropwise over 10 min with vigorous stirring (1000 rpm). Then, cooling bath was removed, and after 1 h at rt iodomethane (1.5 mL, 24.1 mmol, 4.0 equiv) and anhydrous HMPA (5 mL) were added. The flask was sealed and the resulting solution was stirred at 70 °C in an oil bath for 16 h. Then, the mixture was cooled to rt and poured onto brine (50 mL). The mixture was then extracted with DCM/*n*-hexane (1:1, 3×50 mL). Combined organic layers were washed with aqueous solution of HCl (50 mL, 3.5 %), aqueous solution of LiCl (50 mL, 1M), H_2O (50 mL), brine (50 mL) and dried over anhydrous $MgSO_4$. The mixture was the filtered through silica plug (ϕ = 6 cm, d = 2 cm), followed by washing with dichloromethane (200 mL). Evaporation of solvents afforded crude methyl nonaflone ($C_4F_9SO_2CH_3$, 1.047 g, 3.51 mmol, 59%) as a pale yellow oil.

A 25 mL Schlenk tube (with high pressure valve) was charged with benzaldehyde (0.320 g, 3.02 mmol, 1.0 equiv) and flushed with argon. Then, solution of methyl nonaflone ($C_4F_9SO_2CH_3$, 0.990 g, 3.32 mmol, 1.1 equiv) in anhydrous toluene (5 mL) was added. Then, freshly dried 4Å molecular sieves (3 g) and solution of piperidine (0.050 g, 0.6 mmol, 20 mol%) in anhydrous toluene (1 mL) were added. The flask was closed tightly with a teflon valve and the resulting solution was stirred at 115 °C in an oil bath for 24 h. Then, the mixture was cooled to rt, and it was filtered through silica plug (ϕ = 4 cm, d = 2 cm), followed by washing with dichloromethane (200 mL). The mixture was concentrated and separated with column chromatography (ϕ = 6 cm, d = ca. 25 cm, ca. 400 mL of silica gel, eluent: cyclohexane, then cyclohexane/ethyl acetate 50:1 to 15:1) to afford 2-phenylvinyl nonaflone (**1r**, 0.877 g, 2.27 mmol, 75%) as a white solid.

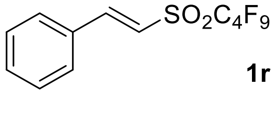

1r

mp = 44.0-45.5 °C.

^{1}H NMR (400 MHz, $CDCl_3$): δ 7.88 (d, *J* = 15.4 Hz, 1H), 7.61 (d, *J* = 7.2 Hz, 2H), 7.60-7.53 (m, 1H), 7.54-7.45 (m, 2H), 6.86 (d, *J* = 15.4 Hz, 1H).

^{13}C NMR (100 MHz, $CDCl_3$): δ 153.9, 133.3, 131.2, 129.6, 129.5, 117.6, 125-100 (complex C_4F_9 signals).

^{19}F NMR (376 MHz, $CDCl_3$): δ -81.2 (t, *J* = 9.5 Hz, 3F), -113.5 (t, *J* = 14.1 Hz, 2F), -121.4 to -121.6 (m, 2F), -126.4 (td, *J* = 14.0, 4.9 Hz, 2F).

After column chromatography **1r** was recrystallized from *n*-hexane in order to remove residual contamination. ^{1}H NMR spectrum and melting point were consistent with the literature: Hanack, M.; Laping, K. *Tetrahedron Lett.* **1977**, *51*, 4493-4494.

II.11. 2-Methoxycarbonyl-1-indanone

The compound was prepared according to the literature: Miura, T.; Moritani, S.; Shiratori, Y.; Murakami, M. *Chem. Commun.* **2022**, *58*, 2710-2713.

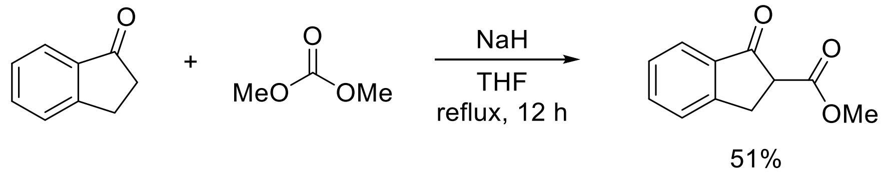

Sodium hydride (0.755 g, 18.9 mmol, 2.5 equiv, 60% dispersion in mineral oil) was added to a flask and mineral oil was washed away with hexane. Under inert atmosphere THF (10 mL), 1-indanone (1.00 g, 7.57 mmol) and dimethyl carbonate (3.40 g, 37.8 mmol, 5 equiv) were added and the reaction mixture was stirred under reflux for 12 h. After cooling down, the mixture was quenched with saturated aqueous solution of NH_4Cl, diluted with water and extracted with ethyl acetate three times. Combined organic layers were dried with Na_2SO_4, filtered, concentrated, purified using column chromatography (silica gel, eluent: hexanes/dichloromethane 70:30 to 30:70), and crystallized from diethyl ether-hexane mixture to afford 2-methoxycarbonyl-1-indanone (0.733 g, 3.85 mmol, 51%) as an orange powder (mp = 57.0-60.5 °C).

^{1}H NMR (400 MHz, $CDCl_3$): δ 7.77 (d, *J* = 7.7 Hz, 1H), 7.62 (t, *J* = 7.5 Hz, 1H), 7.50 (d, *J* = 7.7 Hz, 1H), 7.39 (t, *J* = 7.3 Hz, 1H), 3.78 (s, 3H), 3.73 (dd, *J* = 8.4, 4.0 Hz, 1H), 3.56 (dd, *J* = 17.3, 4.1 Hz, 1H), 3.37 (dd, *J* = 17.3, 8.3 Hz, 1H). Selected enol tautomer (ca. 13%) signals: δ 10.36 (s, 1H), 3.85 (s, 3H).

^{13}C NMR (100 MHz, $CDCl_3$): δ 199.3, 169.4, 153.5, 135.4, 135.1, 127.7, 126.5, 124.5, 53.0, 52.7, 30.1. Selected enol tautomer signals: δ 143.1, 136.7, 129.3, 126.7, 124.6, 120.6, 102.1, 51.1, 32.4.

^{1}H and ^{13}C NMR spectra and melting point were consistent with the literature: Companys, S.; Peixoto, P. A.; Bosset, C.; Chassaing, S.; Miqueu, K.; Sotiropoulos, J.; Pouységu, L.; Quideau, S. *Chem. Eur. J.* **2017**, *23*, 13309-13313.

III. Preparation of catalyst 2d

Monoprotected BOC-*(R,R)*-DACH was prepared according to the literature: Zhang, X.; Emge, T. J.; Hultzsch, K. C. *Angew. Chem. Int. Ed.* **2012**, 51, 394-398; Lee, D. W.; Ha, H.; Lee, W. K. *Synth. Commun.* **2007**, *37*, 737-742.

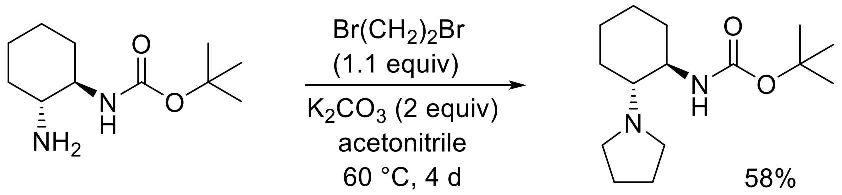

BOC-*(R,R)*-DACH (2.00 g, 9.34 mmol), potassium carbonate (2.60 g, 18.8 mmol, 2 equiv) and 1,4-dibromobutane (2.22 g, 10.28 mmol, 1.1 equiv) were sequentially added to acetonitrile (10 mL) and stirred at 60 °C in an aluminum thermoblock for 4 days. After cooling down to rt, reaction mixture was diluted with DCM (50 mL) filtered through cellite pad and concentrated. Residue was subjected to column chromatography on silica gel (DCM/methanol 100:0 + 0.1% NEt_3 to 90:10 +0.1% NEt_3) to obtain *(R,R)*-N-BOC-2-(1-pyrrolidinyl)-cyclohexylamine (1.43 g, 5.14 mmol, 58%) as a white solid.

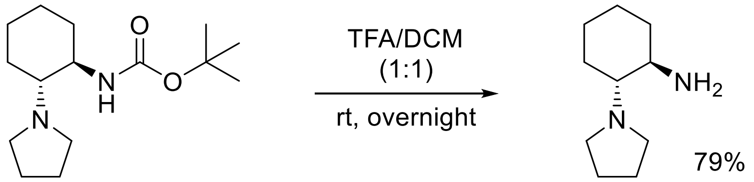

(R,R)-N-BOC-2-(1-pyrrolidinyl)-cyclohexylamine (1.33 g, 4.96 mmol) was dissolved in TFA/DCM mixture (1:1, 4 mL) and left overnight with stirring. Then, the reaction mixture was concentrated, diluted with DCM, basified with saturated aqueous solution of Na_2CO_3, aqueous layer was extracted with DCM three times, combined organic layers were dried with Na_2SO_4 and concentrated to afford *(R,R)*-2-(1-pyrrolidinyl)-cyclohexylamine (660 mg, 3.93 mmol, 79%) as a yellow oil, which was immediately used in next step.

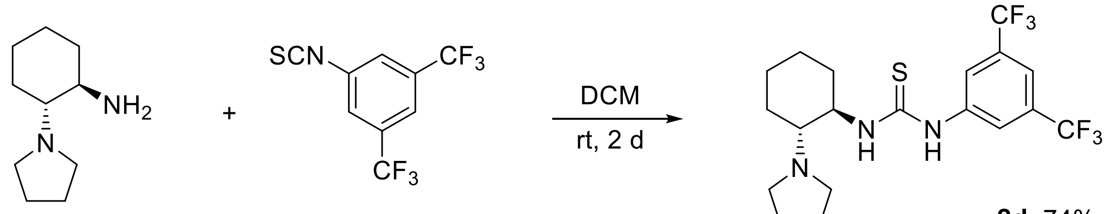

2d, 74%

(R,R)-N-BOC-2-(1-pyrrolidinyl)-cyclohexylamine (660 mg, 3.93 mmol) was dissolved in DCM (2 mL) and 3,5-bis-trifluoromethylphenylisothiocyanate (1.35 g, 4.98 mmol, 1.2 equiv) was added dropwise with stirring. Reaction mixture was stirred for 2 days at rt, then concentrated and purified by column chromatography on silica gel (DCM/methanol 100:0 +0.1% NEt_3 to 95:5 +0.1% NEt_3) to obtain organocatalyst **2d** (1.27 g, 2.89 mmol, 74%) as a white solid. ^{1}H NMR data matched literature (Choudhury, A. R.; Mukherjee, S. *Adv Synth Catal* **2013**, 355 (10), 1989–1995)

^{1}H NMR (400 MHz, $CDCl_3$) δ 8.01 (s, 2H), 7.59 (s, 1H), 3.04 – 2.78 (m, 3H), 2.04 – 1.96 (m, 2H), 1.93 – 1.70 (m, 8H), 1.46 – 1.17 (m, 6H).

^{19}F NMR (376 MHz, $CDCl_3$) δ -62.90 (s).

IV. Enantioselective organocatalytic conjugate additions

IV.1 Screening of catalysts 2a-j in model reaction with triflone 1a

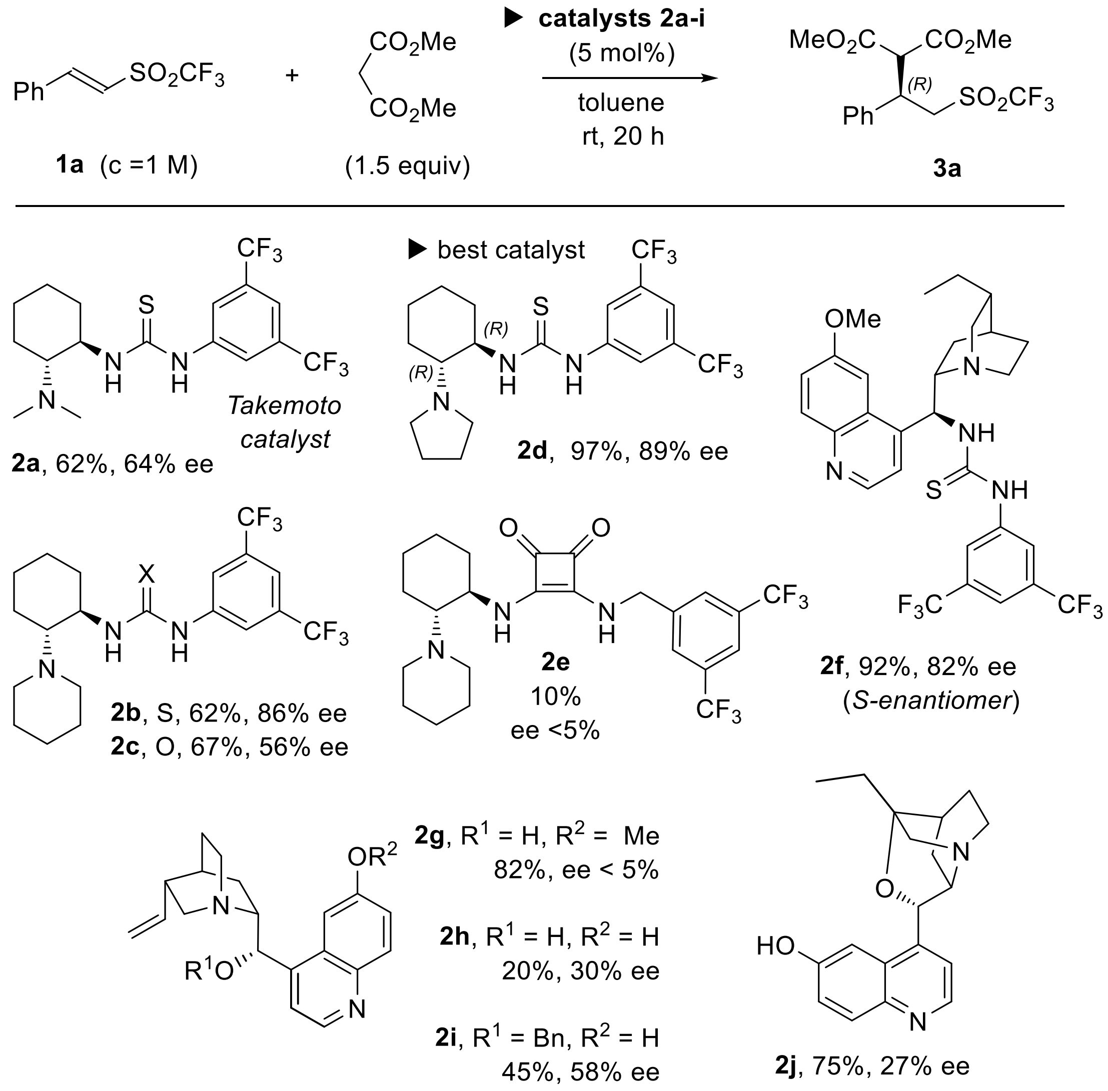

Note: Protonated aminothioureas such as catalyst **2d** may form complexes with certain anions, such as carboxylates, sulfonates and halides (Mandal, T.; Zhao, C. *Angew. Chem. Int. Ed.* **2008**, *47*, 7714-7717). Therefore, it is of particular importance to keep reaction mixture free of the corresponding acids, as they poison aminothiourea catalyst by preventing formation of nucleophile-catalyst complex (Hamza, A.; Schubert, G.; Soós, T.; Pápai, I. *J. Am. Chem. Soc.* **2006**, *128*, 13151-13160). In particular, filtration of malonate esters through a pad of basic alumina can improve their reactivity in the addition reaction.

IV.2 Optimization studies of model reaction with triflone 1a

Solvent screening

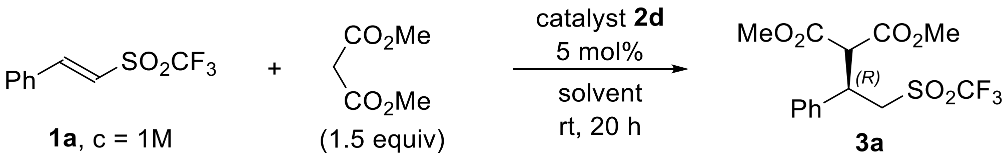

Entry	Solvent	^{19}F NMR yield	Ee
1	toluene	98%	89%
2	DCM	89%	86%
3	$CHCl_3$	83%	87%
4	DCE	83%	85%
5	$PhCF_3$	97%	87%
6	Et_2O	95%	89%
7	MTBE	81%	83%
8	CPME	82%	86%
9	Bu_2O	94%	88%
10	benzene	96%	89%
11	m-xylene	93%	90%
12	mesitylene	95%	90%
13	cyclohexane	99%	85%
14	THF	44%	74%

Further optimization studies (in toluene)

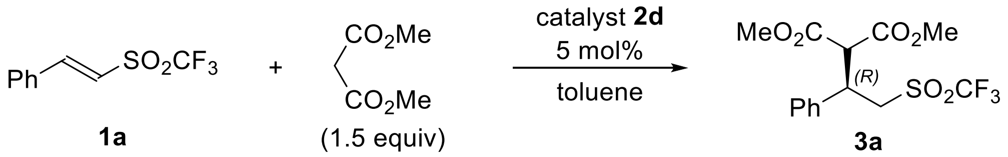

Entry	**2d** (mol%)	Conc. of **1a**	Temp.	Time	Yield[a]	Ee
1	5%	1 M	rt	20 h	97%	89%
2	5%	0.5 M	rt	20 h	89%	90%
3	5%	0.5 M	+5 °C	20 h	76%	93%
4	5%	1 M	+5 °C	20 h	98% (96%)[b]	93%
5	5%	1 M	+5 °C	5 h	60%[c]	93%
6	5%	1 M	-15 °C	20 h	94% (89%)[b]	95%
7	2%	1 M	+5 °C	48 h	99%[d] (95%)[b]	93%
8	10%	1 M	+5 °C	5 h	82%	92%
9	1%	0.5 M	rt	2 h **(9 kbar)**	100% conv.	88%

[a] Yields determined by NMR. [b] Isolated yields. [c] NMR yield after 2 h was 30%. [d] NMR yield after 20 h was 84%.

Toluene is optimal solvent, considering high activity and enantioselectivity achieved in rt at c[**1a**] = 1M. Use of more diluted solution (entry 2), or lower temperature (+5 °C, entry 3) was beneficial for the reaction enantioselectivity. At -15 °C it was improved even further (95% ee, entry 6), but accompanied with a diminished yield of **3a** (89%, 94% ^{19}F NMR). Changes of the catalyst's loading did not affect enantioselectivity, and as optimal conditions we chose reaction time 20 h at +5 °C that ensured adduct **3a** in 96% of isolated yield, and 93% ee (entry 4). Interestingly, the same reaction tested under high-pressure conditions (9 kbar) at rt with 1 mol% of the catalyst **2d** has been completed in only 2 h (entry 9).

IV.3. General procedure for addition of 1,3-dicarbonyl compounds to the vinyl triflones

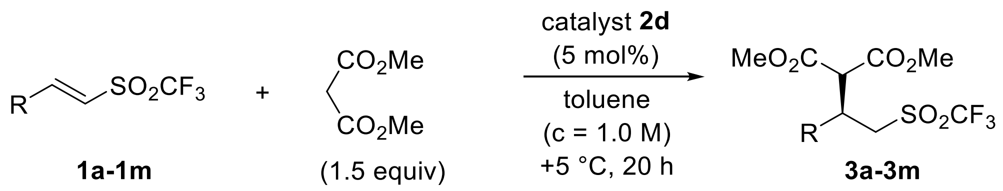

General procedure for dimethyl malonate addition to triflones 1 (Scheme 4 in the manuscript): A screw-top vial was charged with triflone **1** (1.0 mmol, final concentration 1 M), 22 mg of catalyst **2d** (5 mol%) and toluene (ca. 0.55 mL). Then the homogenous solution was cooled to +5 °C (e.g. 15 min in a refrigerator) and 200 mg of dimethyl malonate (1.5 mmol, 1.5 equiv) was added with stirring and reaction mixture was left at +5 °C (refrigerator) for 20 h. After this time, reaction mixture was directly purified using column chromatography on silica gel (eluent: hexanes/ethyl acetate 100:0 to 80:20) to obtain adduct **3**.

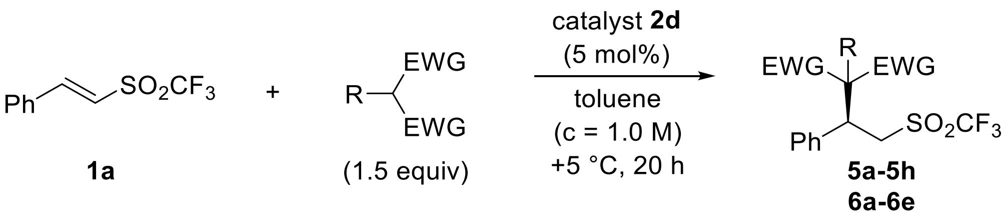

General procedure for reaction of triflone 1a with 1,3-dicarbonyl nucleophiles (Scheme 5 and Table 1 in the manuscript): A screw-top vial was charged with 236 mg of triflone **1a** (1.0 mmol, final concentration 1 M), 22 mg of catalyst **2d** (5 mol%) and toluene (ca. 0.5-0.55 mL). Then the homogenous solution was cooled to +5° C (e.g. 15 min in a refrigerator) and 1.5 mmol of 1,3-dicarbonyl nucleophile (1.5 equiv) was added with stirring and reaction mixture was left at +5° C (refrigerator) for 20 h. After this time, reaction mixture was directly purified using column chromatography on silica gel (eluent: hexanes/ethyl acetate 100:0 to 80:20) to obtain adduct **5** or **6**.

Isolations of triflones **3**, **5** and **6** were typically performed on a scale of 0.3 - 1.2 mmol.

Racemic samples of adducts **3a-n**, **4a-d**, **5a-h**, and **6a-e** were prepared using racemic catalyst **2b** (5 mol%) or DBU (5 mol%) as a catalyst. Racemates were used to determine HPLC method conditions for the separation of enantiomers.

General procedure for high-pressure experiments (9 kbar):
A. **For isolation**: A 0.9 mL Teflon ampoule was charged with catalyst **2d** (10 mg, 5 mol%), triflone **1** (0.45 mmol) and 1,3-dicarbonyl compound (0.68 mmol, 1.5 equiv). Then, Teflon ampoule was filled with toluene up to a volume of 0.9 ml, and after dissolving all reagents, it was closed. Teflon ampoule with homogenous reaction mixture was placed in a high-pressure chamber filled with the inert liquid (hexane) and pressure was slowly increased to 9 kbar (using laboratory hydraulic press U101 from Unipress) at ambient temperature (20-25 °C) by hexane compression. After the pressure has stabilized, the reaction mixture was kept under these conditions for 2 h. After decompression, the reaction mixture was purified by chromatography on a silica gel using hexane fraction from petroleum/AcOEt as an eluent to afford selected triflone adducts.

B. High pressure tests on a small scale: A 1.5 mL vial was charged with 5 mol% of catalyst **2d** (2.2 mg), 0.1 mmol of triflone **1**, toluene and 1.5 equiv of 1,3-dicarbonyl compound (0.15 mmol) to reach a final volume of 0.2 mL. Part of the homogenous reaction mixture was transferred to 0.15 mL Teflon ampoule and compressed in high-pressure apparatus (9 kbar for 2 h). After decompression, the reaction mixture was analyzed by ^{19}F NMR to determine conversion and yield. The enantiomeric excess was determined by HPLC analysis on chiral column after filtration of reaction mixture through a short pad of silica gel with DCM and concentration.

IV.4. Analytical data for adducts 3a-n

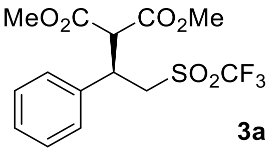

3a

Prepared according to the general procedure from triflone **1a** (287 mg, 1.22 mmol, c = 1M), catalyst **2d** (26 mg, 0.059 mmol, 5 mol%) and dimethyl malonate (240 mg, 1.82 mmol, 1.5 equiv) in toluene at +5 °C. After 20 h, reaction mixture was directly purified using column chromatography on silica gel (eluent: hexanes/ethyl acetate 100:0 to 80:20) to afford **3a** (428 mg, 1.16 mmol, 96%, 93% ee) as a white solid.

Reaction using 2 mol% of **2d**: triflone **1a** (236 mg, 1.00 mmol, c = 1M), catalyst **2d** (9 mg, 0.02 mmol, 2 mol%) and dimethyl malonate (200 mg, 1.51 mmol, 1.5 equiv) in toluene at +5 °C for 2 d provided **3a** (351 mg, 0.95 mmol, 95%, 93% ee) as a white solid.

Reaction at -15 °C: triflone **1a** (71 mg, 0.30 mmol, c = 1M), catalyst **2d** (6.5 mg, 0.015 mmol, 5 mol%) and dimethyl malonate (60 mg, 0.45 mmol, 1.5 equiv) in toluene at -15 °C for 20 h provided **3a** (99 mg, 0.27 mmol, 89%, 94% 19F NMR yield, 95% ee) as a white solid.

^{1}H NMR (400 MHz, $CDCl_3$): δ 7.38-7.26 (m, 5H), 4.13 (ddd, *J* = 10.4, 7.6, 3.5 Hz, 1H), 4.06 (dd, *J* = 14.5, 3.4 Hz, 1H), 3.91-3.86 (m, 1H), 3.83 (d, *J* = 7.7 Hz, 1H), 3.74 (s, 3H), 3.60 (s, 3H).

^{19}F NMR (376 MHz, $CDCl_3$): δ -78.7 (s).

^{13}C NMR (100 MHz, $CDCl_3$): δ 167.8, 167.1, 136.9, 129.0, 128.6, 127.9, 119.2 (q, *J* = 327 Hz), 56.4, 53.0, 52.9, 52.2 (d, *J* = 1.3 Hz), 38.7.

HRMS (ESI) m/z: $[M+H]^+$ Calcd for $C_{14}H_{15}F_3O_6S$: 369.0614; Found 369.0619.

$[\alpha]_D^{25}$ = +10.0 (c 1.04, $CHCl_3$, 93% ee).

Enantiomeric excess was determined by HPLC analysis using a Phenomenex Lux® Amylose-1 column (eluent: hexane/i-PrOH 90:10, flow rate 1.0 mL/min, λ = 209 nm): ent-(R) t_r = 7.00 min and ent-(S) t_r = 9.60 min.

Racemic sample

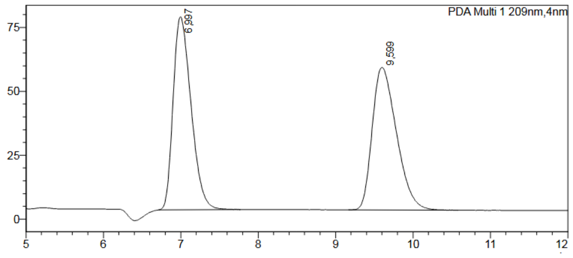

Peak#	Ret. Time	Area	Height	Mark	Area%
1	6,997	1216515	75443	M	50,011
2	9,599	1215977	55789	M	49,989
Total		2432493	131233		100,000

Reaction conducted at +5 °C

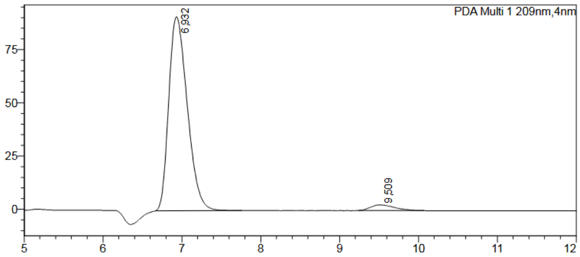

Peak#	Ret. Time	Area	Height	Mark	Area%
1	6,932	1489700	91163	M	96,438
2	9,509	55019	2607	M	3,562
Total		1544719	93771		100,000

Reaction conducted at -15 °C

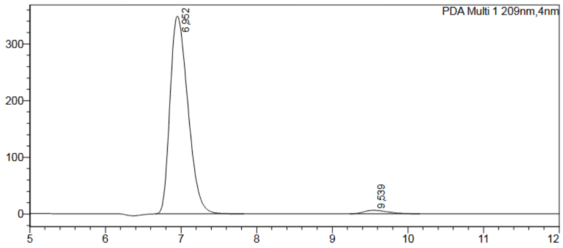

Peak#	Ret. Time	Area	Height	Mark	Area%
1	6,952	5737386	348463	M	97,743
2	9,539	132506	6186	M	2,257
Total		5869892	354649		100,000

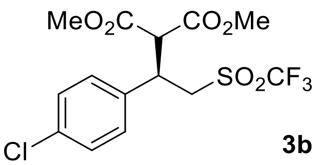

3b

Prepared according to the general procedure from triflone **1b** (273 mg, 1.01 mmol, c = 1M), catalyst **2d** (23 mg, 0.050 mmol, 5 mol%) and dimethyl malonate (200 mg, 1.51 mmol, 1.5 equiv) in toluene at +5 °C. After 20 h, reaction mixture was directly purified using column chromatography on silica gel (eluent: hexanes/ethyl acetate 100:0 to 85:15) to afford **3b** (371 mg, 0.92 mmol, 91%, 92% ee) as a white solid.

A sample was crystallized from hexane/isopropanol 9:1 mixture by slow evaporation (several days) to obtain XRD-quality crystal (**mp** = 63.5-65.0 °C).

^{1}H NMR (400 MHz, $CDCl_3$): δ 7.36-7.31 (m, 2H), 7.26-7.22 (m, 2H), 4.11 (ddd, *J* = 10.6, 7.6, 3.3 Hz, 1H), 4.03 (dd, *J* = 14.5, 3.4 Hz, 1H), 3.83 (dd, *J* = 14.6, 10.3 Hz, 1H), 3.80 (d, *J* = 7.7 Hz, 1H), 3.75 (s, 3H), 3.63 (s, 3H).

^{19}F NMR (376 MHz, $CDCl_3$): δ -78.6 (s).

^{13}C NMR (100 MHz, $CDCl_3$): δ 167.5, 166.9, 135.3, 134.5, 129.4, 129.2, 119.1 (q, *J* = 327 Hz), 56.2, 53.1, 53.0, 52.0, 38.1.

HRMS (ESI) m/z: $[M+H]^+$ Calcd for $C_{14}H_{14}ClF_3O_6S$: 403.0225; Found 403.0222.

$[\alpha]_D^{25}$ = +11.4 (c 1.08, $CHCl_3$, 92% ee).

Enantiomeric excess was determined by HPLC analysis using a Phenomenex Lux® Cellulose-4 column (eluent: hexane/i-PrOH 98:2, flow rate 1.0 mL/min, λ = 209 nm): ent-(R) t_r = 7.06 min and ent-(S) t_r = 7.43 min.

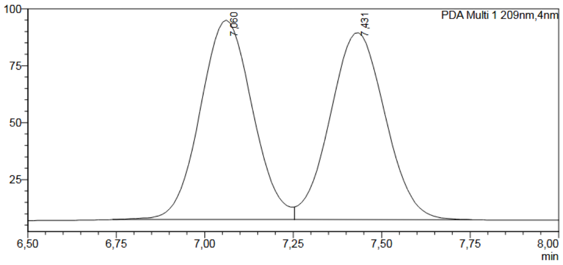

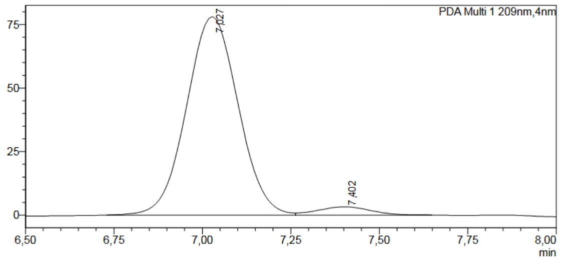

Peak#	Ret. Time	Area	Height	Mark	Area%
1	7,060	900965	87524	M	50,433
2	7,431	885504	82035	V M	49,567
Total		1786470	169559		100,000

Peak#	Ret. Time	Area	Height	Mark	Area%
1	7,027	793179	78065	M	95,938
2	7,402	33581	3268	V M	4,062
Total		826761	81333		100,000

XRD sample, crystallized from hexane/i-PrOH (evap.), 99.5% ee.

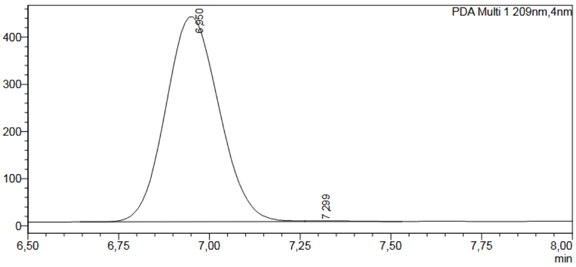

Peak#	Ret. Time	Area	Height	Mark	Area%
1	6,950	4450413	434487	M	99,724
2	7,299	12328	1508	V M	0,276
Total		4462741	435995		100,000

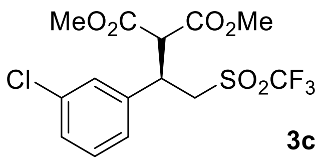

3c

Prepared according to the general procedure from triflone **1c** (137 mg, 0.51 mmol, c = 1M), catalyst **2d** (11 mg, 0.025 mmol, 5 mol%) and dimethyl malonate (99 mg, 0.75 mmol, 1.5 equiv) in toluene at +5 °C. After 20 h, reaction mixture was directly purified using column chromatography on silica gel (eluent: hexanes/ethyl acetate 100:0 to 85:15) to afford **3c** (196 mg, 0.49 mmol, 96%, 88% ee), as a white solid.

Additional experiment at -15 °C using triflone **1c** (27 mg, 0.10 mmol, c = 1M), catalyst **2d** (2.2 mg, 0.005 mmol, 5 mol%) and dimethyl malonate (20 mg, 0.15 mmol, 1.5 equiv) in toluene after 20 h provided **3c** in 98% ^{19}F NMR yield and 93% ee.

^{1}H NMR (400 MHz, $CDCl_3$): δ 7.31-7.28 (m, 3H), 7.19 (ddd, *J* = 5.0, 3.3, 1.5 Hz, 1H), 4.13-4.01 (m, 2H), 3.88-3.79 (m, 2H), 3.75 (s, 3H), 3.65 (s, 3H).

^{19}F NMR (376 MHz, $CDCl_3$): δ -78.6 (s).

^{13}C NMR (100 MHz, $CDCl_3$): δ 167.5, 166.9, 138.9, 134.8, 130.3, 128.8, 128.2, 126.2, 119.2 (q, *J* = 327 Hz), 56.1, 53.1, 53.0, 51.9, 38.3.

HRMS (ESI) m/z: $[M+H]^+$ Calcd for $C_{14}H_{14}ClF_3O_6S$: 403.0225; Found 403.0225.

$[M+Na]^+$ Calcd for $C_{14}H_{14}ClF_3O_6S$ 425.0044; Found 425.0043.

$[\alpha]_D^{25}$ = +12.3 (c 1.03, $CHCl_3$, 88% ee).

Enantiomeric excess was determined by HPLC analysis using a Phenomenex Lux® Cellulose-1 column (eluent: hexane/i-PrOH 95:5, flow rate 1.0 mL/min, λ = 220 nm): ent-(R) t_r = 5.36 min and ent-(S) t_r = 6.25 min.

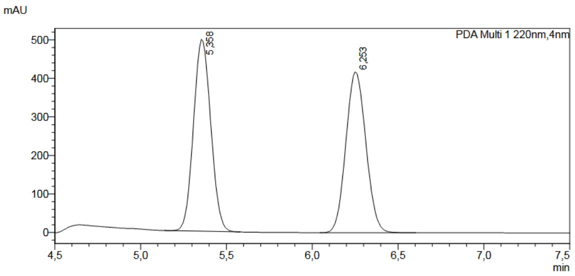

Peak#	Ret. Time	Area	Height	Mark	Area%
1	5,358	3490982	498230	M	50,700
2	6,253	3394565	417959	M	49,300
Total		6885547	916189		100,000

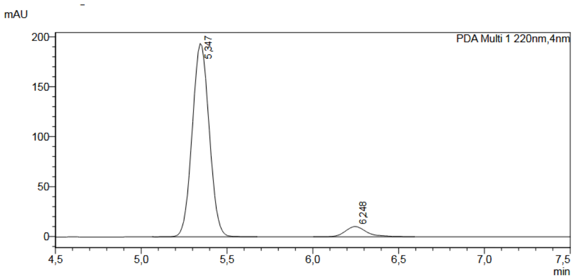

Peak#	Ret. Time	Area	Height	Mark	Area%
1	5,347	1333547	193896	M	93,767
2	6,248	88650	10220	M	6,233
Total		1422197	204116		100,000

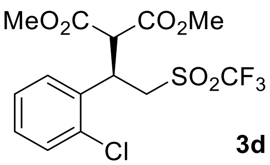

3d

Prepared according to the general procedure from triflone **1d** (189 mg, 0.70 mmol, c = 1M), catalyst **2d** (16 mg, 0.035 mmol, 5 mol%) and dimethyl malonate (140 mg, 1.05 mmol, 1.5 equiv) in toluene at +5 °C. After 20 h, reaction mixture was directly purified using column chromatography on silica gel (eluent: hexanes/ethyl acetate 100:0 to 85:15) to afford **3d** (223 mg, 0.55 mmol, 80%, 95% ee) as a colourless oil.

^{1}H NMR (400 MHz, $CDCl_3$): δ 7.45-7.40 (m, 1H), 7.36-7.32 (m, 1H), 7.30-7.25 (m, 2H), 4.68-4.58 (m, 1H), 4.16 (dd, *J* = 14.9, 10.1 Hz, 1H), 4.10-4.03 (m, 2H), 3.72 (s, 3H), 3.67 (s, 3H).

^{19}F NMR (376 MHz, $CDCl_3$): δ -78.6 (s).

^{13}C NMR (100 MHz, $CDCl_3$): δ 167.6, 167.1, 133.8, 133.7, 130.5, 129.7, 129.5, 127.2, 119.2 (q, *J* = 327 Hz), 54.0, 53.0, 52.9, 50.3, 35.6.

HRMS (ESI) m/z: $[M+H]^+$ Calcd for $C_{14}H_{14}ClF_3O_6S$: 403.0225; Found 403.0224.

$[\alpha]_D^{25}$ = +22.1 (c 1.09, $CHCl_3$, 95% ee).

Enantiomeric excess was determined by HPLC analysis using a Phenomenex Lux® Amylose-1 column (eluent: hexane/i-PrOH 90:10, flow rate 1.0 mL/min, λ = 209 nm): ent-(R) t_r = 4.41 min and ent-(S) t_r = 7.35 min.

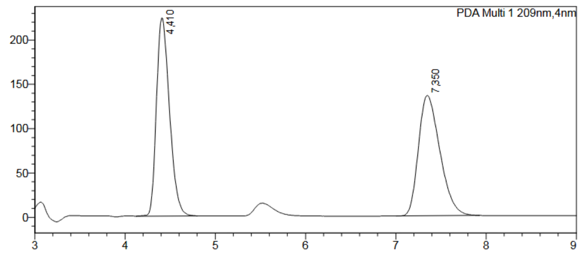

Peak#	Ret. Time	Area	Height	Mark	Area%
1	4,410	2244394	223327	M	49,992
2	7,350	2245156	135755	M	50,008
Total		4489550	359082		100,000

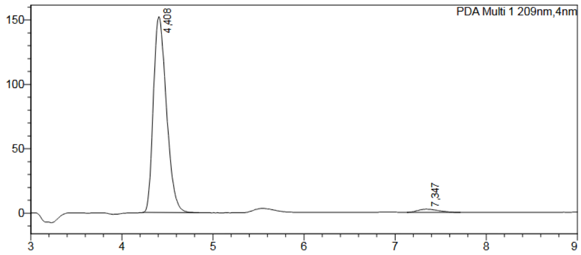

Peak#	Ret. Time	Area	Height	Mark	Area%
1	4,408	1518915	152056	M	97,555
2	7,347	38076	2467	M	2,445
Total		1556990	154523		100,000

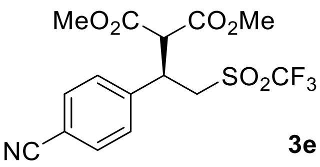

3e

Prepared according to the general procedure from triflone **1e** (130 mg, 0.50 mmol, c = 0.5M), catalyst **2d** (11 mg, 0.025 mmol, 5 mol%), and dimethyl malonate (80 mg, 0.75 mmol, 1.5 equiv) in toluene at at +5 °C with stirring. Both triflone **1e** and adduct **3e** have limited solubility in toluene and reaction mixture remains a suspension. After 20 h, reaction mixture was diluted with DCM and directly purified using column chromatography on silica gel (eluent: hexanes/ethyl acetate 100:0 to 80:20) to afford **3e** (193 mg, 0.49 mmol, 98%, 91% ee) as a white solid.

^{1}H NMR (400 MHz, $CDCl_3$): δ 7.69-7.65 (m, 2H), 7.47-7.43 (m, 2H), 4.18 (ddd, *J* = 10.7, 7.6, 3.3 Hz, 1H), 4.06 (dd, *J* = 14.6, 3.3 Hz, 1H), 3.90-3.82 (m, 2H), 3.76 (s, 3H), 3.63 (s, 3H).

^{19}F NMR (376 MHz, $CDCl_3$): δ -78.5 (s).

^{13}C NMR (100 MHz, $CDCl_3$): δ 167.2, 166.6, 142.1, 132.7, 129.0, 119.0 (q, *J* = 327 Hz), 118.1, 112.6, 55.7, 53.2, 53.1, 51.6, 38.6.

HRMS (ESI) m/z: $[M+H]^+$ Calcd for $C_{15}H_{14}F_3NO_6S$: 394.0567; Found 394.0569.

$[\alpha]_D^{25}$ = +13.3 (c 1.00, $CHCl_3$, 91% ee).

Enantiomeric excess was determined by HPLC analysis using a Phenomenex Lux® Amylose-1 column (eluent: hexane/i-PrOH 80:20, flow rate 1.0 mL/min, λ = 220 nm): ent-(R) t_r = 5.09 min and ent-(S) t_r = 12.50 min.

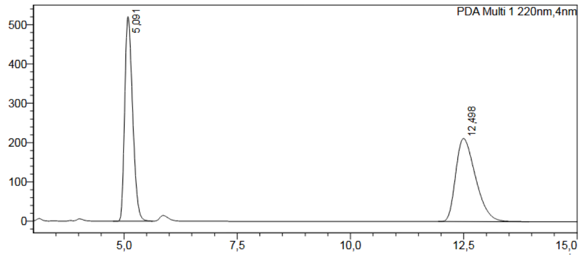

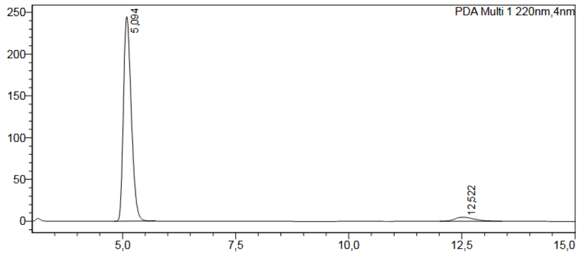

Peak#	Ret. Time	Area	Height	Mark	Area%
1	5,091	6336777	520282	M	49,583
2	12,498	6443371	211354	M	50,417
Total		12780148	731636		100,000

Peak#	Ret. Time	Area	Height	Mark	Area%
1	5,094	2968172	244930		95,401
2	12,522	143098	4869		4,599
Total		3111270	249799		100,000

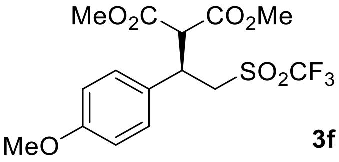

3f

Prepared according to the general procedure from triflone **1f** (186 mg, 0.70 mmol, c = 1M), catalyst **2d** (16 mg, 0.035 mmol, 5 mol%), and dimethyl malonate (140 mg, 1.05 mmol, 1.5 equiv) in toluene at +5 °C. After 20 h, conversion (^{19}F NMR) was 45%. After 3 days, reaction mixture was directly purified using column chromatography on silica gel (eluent: hexanes/ethyl acetate 100:0 to 80:20) to afford **3f** (242 mg, 0.61 mmol, 87%, 92% ee) as a white solid.

High pressure experiment: small scale reaction using triflone **1f** (26 mg, 0.097 mmol, c = 0.5M), dimethyl malonate (20 mg, 0.151 mmol, 1.5 equiv) and catalyst **2d** (2.2 mg, 0.005 mmol, 5 mol%) in toluene (0.15 ml, 0.2ml total volume) conducted under high pressure conditions (9 kbar, 2 h) provided **3f** with >99% conversion (^{19}F NMR) and 91% ee.

^{1}H NMR (400 MHz, $CDCl_3$): δ 7.20 (d, *J* = 8.7 Hz, 2H), 6.87 (d, *J* = 8.7 Hz, 2H), 4.09 (ddd, *J* = 10.6, 7.7, 3.4 Hz, 1H), 4.02 (dd, *J* = 14.4, 3.5 Hz, 1H), 3.87-3.80 (m, 2H), 3.78 (s, 3H), 3.74 (s, 3H), 3.61 (s, 3H).

^{19}F NMR (376 MHz, $CDCl_3$): δ -78.8 (s).

^{13}C NMR (100 MHz, $CDCl_3$): δ 167.8, 167.1, 159.5, 129.1, 128.6, 120.8 (q, *J* = 328 Hz), 114.3, 56.5, 55.1, 52.9, 52.9, 52.4, 37.9.

HRMS (ESI) m/z: $[M+H]^+$ Calcd for $C_{15}H_{17}F_3O_7S$: 399.0720; Found 399.0718.

$[\alpha]_D^{25}$ = +10.4 (c 1.02, $CHCl_3$, 92% ee).

Enantiomeric excess was determined by HPLC analysis using a Phenomenex Lux® Amylose-1 column (eluent: hexane/i-PrOH 90:10, flow rate 1.0 mL/min, λ = 209 nm): ent-(R) t_r = 6.11 min and ent-(S) t_r = 12.31 min.

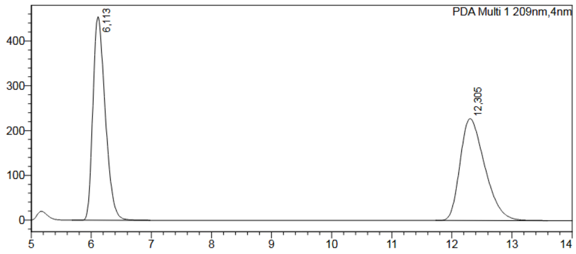

Peak#	Ret. Time	Area	Height	Mark	Area%
1	6,113	6503795	454295		50,007
2	12,305	6501902	227374		49,993
Total		13005697	681669		100,000

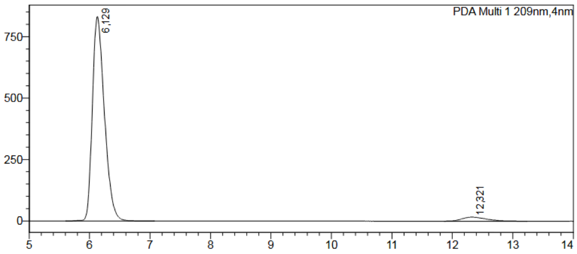

Peak#	Ret. Time	Area	Height	Mark	Area%
1	6,129	11573980	832086	M	96,173
2	12,321	460590	16356	M	3,827
Total		12034569	848442		100,000

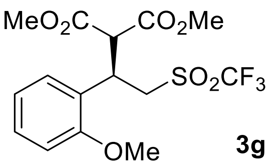

3g

Prepared according to the general procedure from triflone **1g** (134 mg, 0.50 mmol, c = 1M), catalyst **2d** (11 mg, 0.025 mmol, 5 mol%) and dimethyl malonate (99 mg, 0.75 mmol, 1.5 equiv) in toluene at +5 °C. After 20 h, reaction mixture was directly purified using column chromatography on silica gel (eluent: hexanes/ethyl acetate 100:0 to 80:20) to afford **3g** (178 mg, 0.45 mmol, 89%, 97% ee) as a white solid.

^{1}H NMR (400 MHz, $CDCl_3$): δ 7.29 (ddd, *J* = 8.3, 7.5, 1.7 Hz, 1H), 7.20 (dd, *J* = 7.5, 1.7 Hz, 1H), 6.94-6.88 (m, 2H), 4.26-4.10 (m, 3H), 3.94-3.89 (m, 1H), 3.88 (s, 3H), 3.76 (s, 3H), 3.49 (s, 3H).

^{19}F NMR (376 MHz, $CDCl_3$): δ -78.8 (s).

^{13}C NMR (100 MHz, $CDCl_3$): δ 168.1, 167.2, 157.3, 131.1, 130.0, 123.6, 120.8, 119.2 (d, *J* = 327 Hz), 111.1, 55.4, 53.9, 52.9, 52.5, 50.5, 36.6.

HRMS (ESI) m/z: $[M+H]^+$ Calcd for $C_{15}H_{17}F_3O_7S$: 399.0720; Found 399.0720.

$[\alpha]_D^{25}$ = -13.1 (c 1.04, $CHCl_3$, 97% ee).

Enantiomeric excess was determined by HPLC analysis using a Phenomenex Lux® Amylose-2 column (eluent: hexane/i-PrOH 95:5, flow rate 1.0 mL/min, λ = 220 nm): ent-(R) t_r = 6.28 min and ent-(S) t_r = 7.34 min.

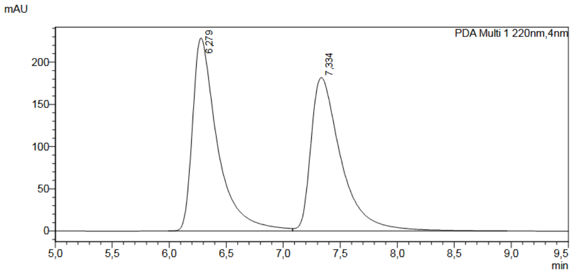

Peak#	Ret. Time	Area	Height	Mark	Area%
1	6,279	3483407	228505		50,999
2	7,334	3346918	181735	V	49,001
Total		6830324	410240		100,000

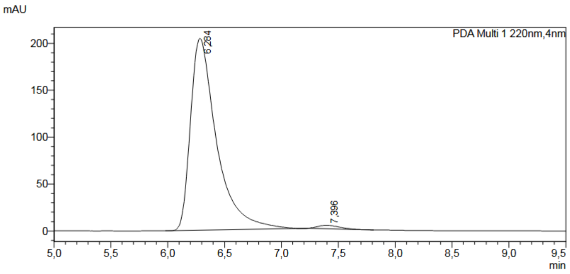

Peak#	Ret. Time	Area	Height	Mark	Area%
1	6,284	3273482	204508	M	98,512
2	7,396	49457	3686	M	1,488
Total		3322939	208194		100,000

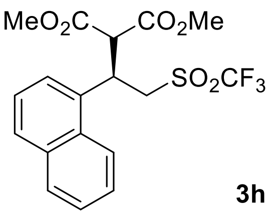

3h

Prepared according to the general procedure from triflone **1h** (292 mg, 1.02 mmol, c = 0.5M), catalyst **2d** (23 mg, 0.050 mmol, 5 mol%) and dimethyl malonate (200 mg, 1.51 mmol, 1.5 equiv) in toluene at +5 °C. After 2 days, reaction mixture was directly purified using column chromatography on silica gel (eluent: hexanes/ethyl acetate 100:0 to 80:20) to afford **3h** (413 mg, 0.98 mmol, 97%, 94% ee) as yellow oil.

^{1}H NMR (400 MHz, $CDCl_3$): δ 8.14 (d, *J* = 8.6 Hz, 1H), 7.89 (dd, *J* = 8.2, 1.4 Hz, 1H), 7.82 (dd, *J* = 7.3, 2.0 Hz, 1H), 7.61 (ddd, *J* = 8.5, 6.8, 1.4 Hz, 1H), 7.55-7.43 (m, 3H), 5.09 (s, 1H), 4.25-4.22 (m, 2H), 4.03-3.97 (m, 1H), 3.67 (s, 3H), 3.61 (s, 3H).

^{19}F NMR (376 MHz, $CDCl_3$): δ -78.7 (s).

^{13}C NMR (100 MHz, $CDCl_3$): δ 167.9, 167.4, 134.1, 132.6, 130.5, 129.3, 129.2, 127.1, 126.1, 125.0, 124.6, 122.0, 119.2 (q, *J* = 327 Hz), 55.7, 53.0, 52.8, 51.5, 32.6.

HRMS (ESI) m/z: $[M+H]^+$ Calcd for $C_{18}H_{17}F_3O_6S$: 419.0771; Found 419.0768.

$[\alpha]_D^{25}$ = +45.8 (c 1.05, $CHCl_3$, 94% ee).

Enantiomeric excess was determined by HPLC analysis using a Phenomenex Lux® Amylose-1 column (eluent: hexane/i-PrOH 95:5, flow rate 1.0 mL/min, λ = 209 nm): ent-(R) t_r = 8.89 min and ent-(S) t_r = 12.02 min.

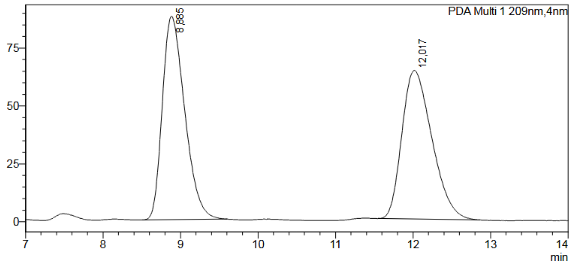

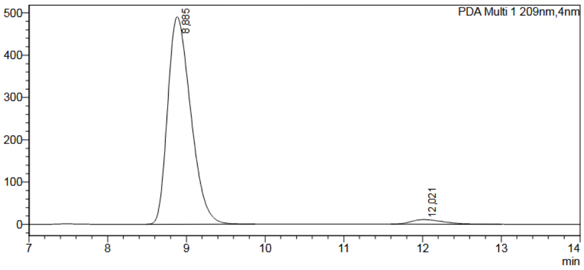

Peak#	Ret. Time	Area	Height	Mark	Area%
1	8,885	1764725	87789	M	50,527
2	12,017	1727923	64136	M	49,473
Total		3492649	151925		100,000

Peak#	Ret. Time	Area	Height	Mark	Area%
1	8,885	10004021	490288	M	97,048
2	12,021	304295	10799	M	2,952
Total		10308317	501087		100,000

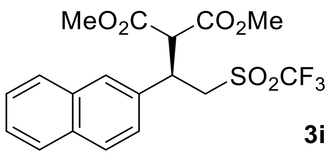

3i

Prepared according to the general procedure from triflone **1i** (143 mg, 0.50 mmol, c = 1M), catalyst **2d** (11 mg, 0.025 mmol, 5 mol%) and dimethyl malonate (99 mg, 0.75 mmol, 1.5 equiv) in toluene at +5 °C. After 20 h, conversion (^{19}F NMR) was 96%. After 2 days, reaction mixture was directly purified using column chromatography on silica gel (eluent: hexanes/ethyl acetate 100:0 to 80:20) to afford **3i** (201 mg, 0.48 mmol, 96%, 87% ee) as a white solid.

^{1}H NMR (400 MHz, $CDCl_3$): δ 7.87-7.75 (m, 4H), 7.53-7.46 (m, 2H), 7.37 (dd, *J* = 8.5, 2.0 Hz, 1H), 4.31 (ddd, *J* = 10.4, 7.4, 3.5 Hz, 1H), 4.14 (dd, *J* = 14.6, 3.5 Hz, 1H), 4.01 (dd, *J* = 14.6, 9.8 Hz, 1H), 3.93 (d, *J* = 7.5 Hz, 1H), 3.74 (s, 3H), 3.59 (s, 3H).

^{19}F NMR (376 MHz, $CDCl_3$): δ -78.6 (s).

^{13}C NMR (100 MHz, $CDCl_3$): δ 167.8, 167.1, 134.3, 133.2, 133.0, 129.0, 128.0, 127.7, 127.5, 126.6, 125.0, 119.2 (q, *J* = 327 Hz), 56.5, 53.0, 52.9, 52.1, 38.8.

HRMS (ESI) m/z: $[M+H]^+$ Calcd for $C_{18}H_{17}F_3O_6S$: 419.0771; Found 419.0771.

$[M+Na]^+$ Calcd for $C_{18}H_{17}F_3O_6S$ 441.0590; Found 441.0591.

$[\alpha]_D^{25}$ = +16.1 (c 0.79, $CHCl_3$, 87% ee).

Enantiomeric excess was determined by HPLC analysis using a Phenomenex Lux® Cellulose-1 column (eluent: hexane/i-PrOH 90:10, flow rate 1.0 mL/min, λ = 220 nm): ent-(R) t_r = 4.77 min and ent-(S) t_r = 5.74 min.

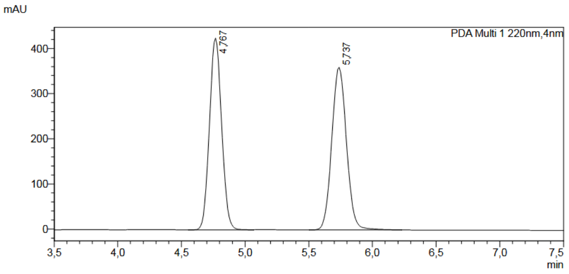

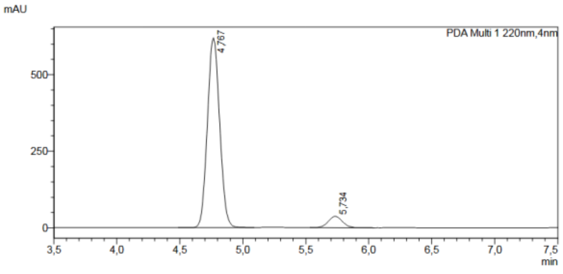

Peak#	Ret. Time	Area	Height	Mark	Area%
1	4,767	2820232	424468	M	49,310
2	5,737	2899121	360384	M	50,690
Total		5719353	784852		100,000

Peak#	Ret. Time	Area	Height	Mark	Area%
1	4,767	4186668	619328		93,608
2	5,734	285868	36584		6,392
Total		4472535	655912		100,000

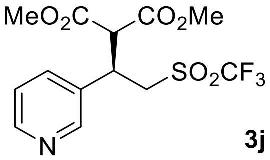

3j

Prepared according to the general procedure from triflone **1j** (165 mg, 0.70 mmol, c = 0.5M), catalyst **2d** (16 mg, 0.035 mmol, 5 mol%) and dimethyl malonate (140 mg, 1.05 mmol, 1.5 equiv) in toluene at +5 °C. After 20 h, reaction mixture was directly purified using column chromatography on silica gel (eluent: hexanes/ethyl acetate 100:0 to 50:50) to afford **3j** (250 mg, 0.68 mmol, 97%, 83% ee) as yellow solid.

^{1}H NMR (400 MHz, $CDCl_3$): δ 8.59-8.57 (m, 2H), 7.68 (dt, *J* = 8.1, 1.9 Hz, 1H), 7.31 (dd, *J* = 8.0, 4.8 Hz, 1H), 4.15 (ddd, *J* = 10.5, 7.5, 3.5 Hz, 1H), 4.08 (dd, *J* = 14.6, 3.5 Hz, 1H), 3.94-3.85 (m, 2H), 3.76 (s, 3H), 3.64 (s, 3H).

^{19}F NMR (376 MHz, $CDCl_3$): δ -78.5 (s).

^{13}C NMR (100 MHz, $CDCl_3$): δ 167.4, 166.8, 149.9, 149.6, 135.6, 132.6, 123.6, 119.1 (q, *J* = 327 Hz), 55.9, 53.2, 53.1, 51.7, 36.4.

HRMS (ESI) m/z: $[M+H]^+$ Calcd for $C_{13}H_{14}F_3NO_6S$: 370.0567; Found 370.0565.

$[\alpha]_D^{25}$ = +10.6 (c 0.99, $CHCl_3$, 83% ee).

Enantiomeric excess was determined by HPLC analysis using a Phenomenex Lux® Cellulose-1 column (eluent: hexane/i-PrOH 80:20, flow rate 1.0 mL/min, λ = 209 nm): ent-(R) t_r = 4.15 min and ent-(S) t_r = 4.73 min.

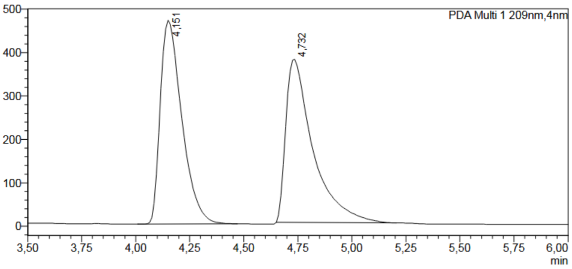

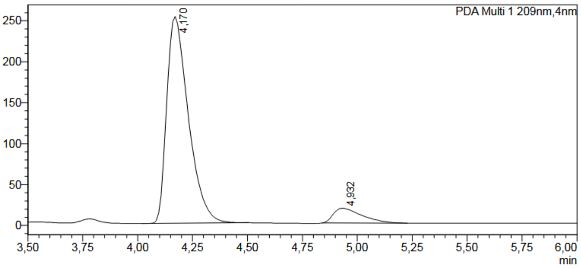

Peak#	Ret. Time	Area	Height	Mark	Area%
1	4,151	3255627	469302	M	49,737
2	4,732	3290110	376057	M	50,263
Total		6545737	845359		100,000

Peak#	Ret. Time	Area	Height	Mark	Area%
1	4,170	1730253	252503	M	91,380
2	4,932	163212	18173	M	8,620
Total		1893464	270677		100,000

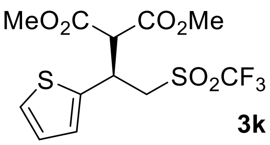

3k

Prepared according to the general procedure from triflone **1k** (172 mg, 0.71 mmol, c = 1M) catalyst **2d** (16 mg, 0.035 mmol, 5 mol%) and dimethyl malonate (140 mg, 1.05 mmol, 1.5 equiv) in toluene at +5 °C. After 20 h, reaction mixture was directly purified using column chromatography on silica gel (eluent: hexanes/ethyl acetate 100:0 to 85:15) to afford **3k** (212 mg, 0.57 mmol, 80%, 93% ee) as a white solid.

^{1}H NMR (400 MHz, $CDCl_3$): δ 7.29-7.25 (m, 1H), 7.03 (d, *J* = 3.6 Hz, 1H), 6.96 (dd, *J* = 5.1, 3.6 Hz, 1H), 4.45 (ddd, *J* = 9.8, 6.3, 3.8 Hz, 1H), 4.09 (dd, *J* = 14.7, 3.8 Hz, 1H), 3.97-3.86 (m, 2H), 3.75 (s, 3H), 3.71 (s, 3H).

^{19}F NMR (376 MHz, $CDCl_3$): δ -78.7 (s).

^{13}C NMR (100 MHz, $CDCl_3$): δ 167.5, 167.0, 139.4, 127.2, 127.0, 125.9, 119.2 (q, *J* = 327 Hz), 56.6, 53.1 (d, *J* = 1.3 Hz), 53.1, 53.0, 34.1.

HRMS (ESI) m/z: $[M+H]^+$ Calcd for $C_{12}H_{13}F_3O_6S_2$: 375.0178; Found 375.0176.

$[\alpha]_D^{25}$ = +23.6 (c 1.05, $CHCl_3$, 93% ee).

Enantiomeric excess was determined by HPLC analysis using a Phenomenex Lux® Amylose-1 column (eluent: hexane/i-PrOH 90:10, flow rate 1.0 mL/min, λ = 235 nm): ent-(S) t_r = 5.05 min and ent-(R) t_r = 6.00 min.

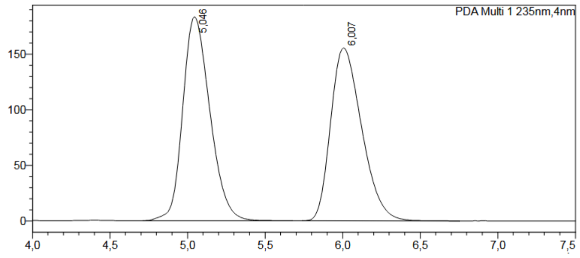

Peak#	Ret. Time	Area	Height	Mark	Area%
1	5,046	2240297	183387	M	50,868
2	6,007	2163807	155495	M	49,132
Total		4404105	338882		100,000

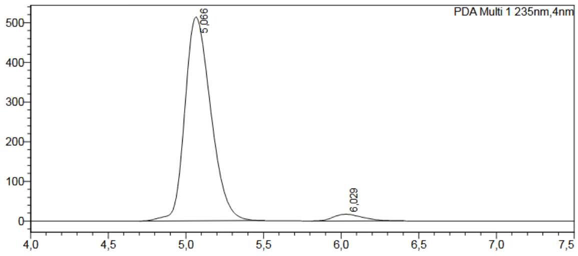

Peak#	Ret. Time	Area	Height	Mark	Area%
1	5,066	5988856	514417	M	96,422
2	6,029	222257	16819	M	3,578
Total		6211113	531235		100,000

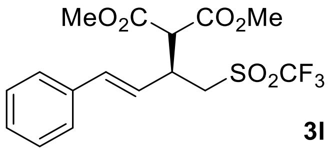

3l

Prepared according to the general procedure from triflone **1l** (78 mg, 0.30 mmol, c = 1M), catalyst **2d** (6.5 mg, 0.015 mmol, 5 mol%) and dimethyl malonate (59 mg, 0.45 mmol, 1.5 equiv) in toluene at +5 °C. After 20 h, conversion (^{19}F NMR) was 26%, and after 5 days, 75%. After 7 days, reaction mixture was directly purified using column chromatography on silica gel (eluent: hexanes/ethyl acetate 100:0 to 85:15) to afford **3l** (97 mg, 0.25 mmol, 82%, 91% ee) as a white solid.

Additional experiment conducted at rt for 5 days gave 83% yield (^{19}F NMR) and 88% ee.

High pressure reaction: 0.9 mL teflon vial was loaded with triflone **1l** (121 mg, 0.46 mmol, c = 0.5 M), dimethyl malonate (89 mg, 0.67 mmol, 1.5 equiv), catalyst **2d** (10 mg, 0.022 mmol, 5 mol%), filled up with toluene, and homogenous reaction mixture was subjected to high pressure conditions (9 kbar, 2 h). After decompression reaction mixture was directly purified on chromatographic column (eluent: hexanes/ethyl acetate 100:0 to 85:15) to afford **3l** (175 mg, 0.44 mmol, 96%, >99% conv. (^{19}F NMR), 73% ee) as a white solid.

^{1}H NMR (400 MHz, $CDCl_3$): δ 7.38-7.22 (m, 5H), 6.64 (d, *J* = 15.7 Hz, 1H), 6.21 (dd, *J* = 15.7, 8.8 Hz, 1H), 3.86-3.62 (m, 10H).

^{19}F NMR (376 MHz, $CDCl_3$): δ -78.6 (s).

^{13}C NMR (100 MHz, $CDCl_3$): δ 167.6, 167.5, 135.7, 135.2, 128.6, 128.3, 126.6, 124.6, 119.2 (q, *J* = 327 Hz), 54.7, 52.9 (d, *J* = 2.1 Hz), 51.3, 36.8.

HRMS (ESI) m/z: $[M+H]^+$ Calcd for $C_{16}H_{17}F_3O_6S$: 395.0771; Found 395.0770.

$[\alpha]_D^{25}$ = +11.6 (c 1.01, $CHCl_3$, 73% ee).

Enantiomeric excess was determined by HPLC analysis using a Phenomenex Lux® Amylose-1 column (eluent: hexane/i-PrOH 95:5, flow rate 1.0 mL/min, λ = 245 nm): ent-(R) t_r = 6.14 min and ent-(S) t_r = 8.20 min.

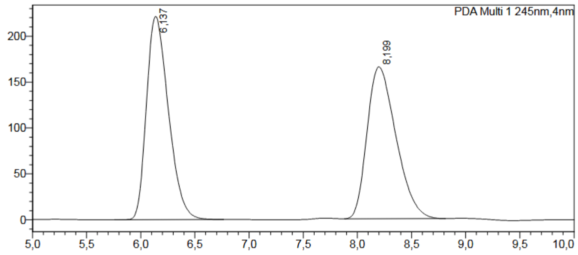

Peak#	Ret. Time	Area	Height	Mark	Area%
1	6,137	3120288	221092	M	50,325
2	8,199	3079944	165432	M	49,675
Total		6200232	386524		100,000

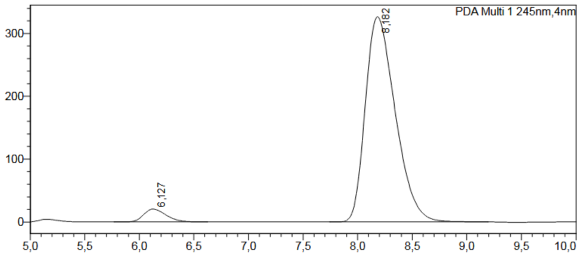

Peak#	Ret. Time	Area	Height	Mark	Area%
1	6,127	285362	20259	M	4,386
2	8,182	6220622	326492	M	95,614
Total		6505984	346751		100,000

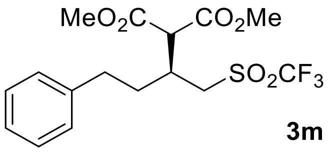

3m

Prepared according to modified general procedure from triflone **1m** (79 mg, 0.30 mmol, c = 0.5M), catalyst **2d** (6.5 mg, 0.015 mmol, 5 mol%), and dimethyl malonate (59 mg, 0.45 mmol, 1.5 equiv) in toluene at -15 °C. After 20 h, conversion (^{19}F NMR) was 81%. After 2 days, reaction mixture was directly purified using column chromatography on silica gel (eluent: hexanes/ethyl acetate 100:0 to 90:10) to afford **3m** (98 mg, 0.25 mmol, 83%, 87% ee) as a colourless oil.

Reactions conducted at +5 °C, c[**1m**] = 0.5M and c[**1m**] = 1M after 20 h provided product **3m** in 93% conv. (^{19}F NMR) and 78% ee and >99% conv. (^{19}F NMR) and 82% ee respectively.

^{1}H NMR (400 MHz, $CDCl_3$): δ 7.32-7.27 (m, 2H), 7.24-7.15 (m, 3H), 3.87 (d, *J* = 4.4 Hz, 1H), 3.82-3.74 (m, 1H), 3.76 (s, 3H), 3.76 (s, 3H), 3.41 (dd, *J* = 14.8, 7.5 Hz, 1H), 2.87 (dp, *J* = 10.6, 3.9, 3.1 Hz, 1H), 2.78-2.65 (m, 2H), 2.11 (ddt, *J* = 13.9, 9.3, 6.4 Hz, 1H), 1.97-1.83 (m, 1H).

^{19}F NMR (376 MHz, $CDCl_3$): δ -78.6 (s).

^{13}C NMR (100 MHz, $CDCl_3$): δ 168.1, 167.9, 139.9, 128.6, 128.2, 126.4, 119.3 (q, *J* = 327 Hz), 53.0, 52.8, 52.3, 50.6, 33.7, 32.9, 32.1.

HRMS (ESI) m/z: $[M+H]^+$ Calcd for $C_{16}H_{19}F_3O_6S$: 397.0927; Found 397.0927.

$[\alpha]_D^{25}$ = +4.9 (c 1.00, $CHCl_3$, 87% ee).

Enantiomeric excess was determined by HPLC analysis using a Phenomenex Lux® Amylose-1 column (eluent: hexane/i-PrOH 98:2, flow rate 1.0 mL/min, λ = 209 nm): ent-(R) t_r = 4.93 min and ent-(S) t_r = 6.12 min.

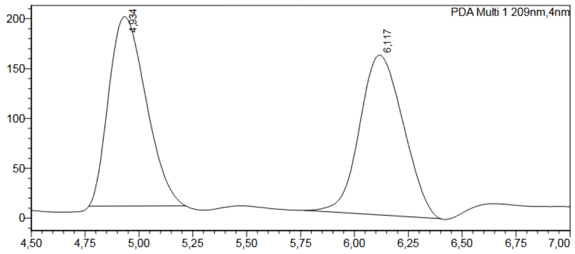

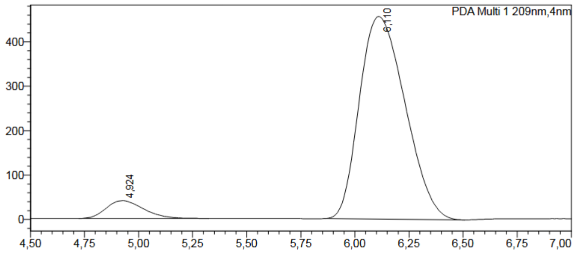

Peak#	Ret. Time	Area	Height	Mark	Area%
1	4,934	2290429	189550	M	50,366
2	6,117	2257147	160244	M	49,634
Total		4547576	349794		100,000

Peak#	Ret. Time	Area	Height	Mark	Area%
1	4,924	478121	39956	M	6,651
2	6,110	6710925	456261	M	93,349
Total		7189045	496217		100,000

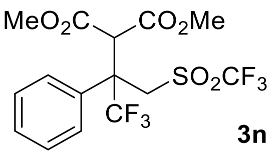

3n

High pressure reaction: A 0.9 mL teflon vial was loaded with triflone **1n** (140 mg, 0.46 mmol, c = 0.5 M), dimethyl malonate (89 mg, 0.67 mmol, 1.5 equiv), catalyst **2d** (10 mg, 0.022 mmol, 5 mol%), filled up with toluene, and homogenous reaction mixture was subjected to high pressure conditions (9 kbar, 20 h). After decompression reaction mixture was directly purified on chromatographic column (silica gel, eluent: hexanes/ethyl acetate 100:0 to 95:5) to afford **3n** (156 mg, 0.36 mmol, 78%, 88% ^{19}F NMR yield, 61% ee) as a colourless oil.

Reaction conducted at 1 atm according to general procedure at c[**1n**] = 0.5M after 20 h gave 1% conversion (^{19}F NMR) and after 5 d 3.5% conversion (^{19}F NMR).

^{1}H NMR (400 MHz, $CDCl_3$): δ 7.59-7.54 (m, 2H), 7.45-7.39 (m, 3H), 5.06 (dd, *J* = 15.4, 1.5 Hz, 1H), 4.83 (d, *J* = 15.4 Hz, 1H), 4.37 (s, 1H), 3.83 (s, 3H), 3.48 (s, 3H).

^{19}F NMR (376 MHz, $CDCl_3$): δ -64.0 (s), -79.3 (s).

^{13}C NMR (100 MHz, $CDCl_3$): δ 165.9, 165.5, 131.6, 129.5, 128.8, 127.1 (q, *J* = 2.1 Hz), 125.2 (q, *J* = 286.1 Hz), 119.4 (q, *J* = 328 Hz), 56.3 (d, *J* = 2.1 Hz), 54.4 (q, *J* = 27.0 Hz), 53.7, 53.2, 48.0.

HRMS (ESI) m/z: $[M+H]^+$ Calcd for $C_{15}H_{14}F_6O_6S$: 437.0488; Found 437.0491.

$[\alpha]_D^{25}$ = -27.5 (c 1.04, $CHCl_3$, 61% ee).

Enantiomeric excess was determined by HPLC analysis using a Phenomenex Lux® Cellulose-2 column (eluent: hexane/i-PrOH 99:1, flow rate 1.0 mL/min, λ = 209 nm): minor enantiomer t_r = 3.43 min and major enantiomer t_r = 4.13 min.

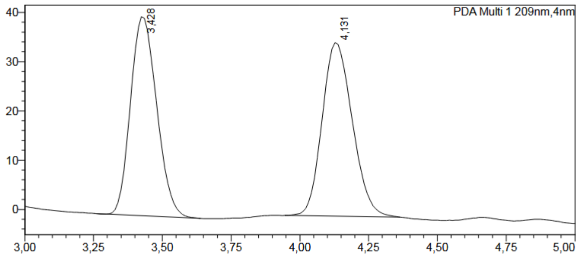

Peak#	Ret. Time	Area	Height	Mark	Area%
1	3,428	267749	40406	M	49,881
2	4,131	269023	35222	M	50,119
Total		536772	75628		100,000

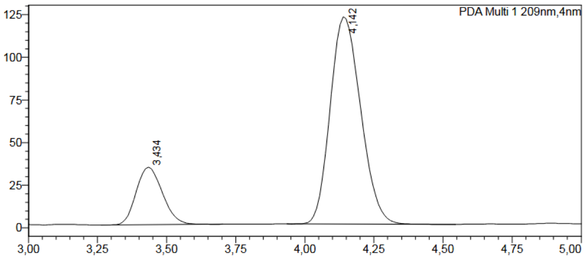

Peak#	Ret. Time	Area	Height	Mark	Area%
1	3,434	222597	33706	M	19,254
2	4,142	933506	121503	M	80,746
Total		1156104	155209		100,000

IV.5. Analytical data for adducts 4a-d

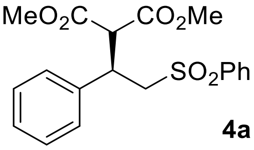

4a

High pressure reaction: A 0.5mL teflon vial was loaded with sulfone **1p** (122 mg, 0.5 mmol, c = 1 M), dimethyl malonate (132 mg, 1.00 mmol, 2 equiv), catalyst **2d** (22 mg, 0.05 mmol, 10 mol%), filled up with toluene, and homogenous reaction mixture was subjected to 9 kbar of hydrostatic pressure for 20 h. After decompression reaction mixture was directly purified on chromatographic column (silica gel, 100:0 to 90:10 hexanes/ethyl acetate) to afford **4a** (20 mg, 0.053 mmol, 11%, 20% ^{1}H NMR yield, 90% ee) as a white solid.

In reaction conducted at 1 atm according to general procedure at c[**1p**] = 1M no product was observed after 7 d.

^{1}H NMR (400 MHz, $CDCl_3$): δ 7.64 (dd, *J* = 7.7, 2.0 Hz, 2H), 7.51 (t, *J* = 7.5 Hz, 1H), 7.37 (t, J = 7.8 Hz, 2H), 7.17-7.12 (m, 3H), 7.07 (dd, *J* = 6.6, 3.1 Hz, 2H), 3.97 (td, *J* = 8.2, 5.1 Hz, 1H), 3.81-3.74 (m, 3H), 3.69 (s, 3H), 3.51 (s, 3H).

^{13}C NMR (100 MHz, $CDCl_3$): δ 167.8, 167.2, 139.3, 137.2, 133.2, 128.9, 128.4, 128.1, 127.7, 127.7, 58.2, 56.9, 52.7, 52.5, 40.1.

HRMS (ESI) m/z: $[M+H]^+$ Calcd for $C_{19}H_{20}O_6S$: 377.1053; Found 377.1054.

$[\alpha]_D^{25}$ = -2.5 (c 0.91, $CHCl_3$, 90% ee).

Enantiomeric excess was determined by HPLC analysis using a Phenomenex Lux® Amylose-1 column (eluent: hexane/i-PrOH 90:10, flow rate 1.0 mL/min, λ = 209 nm): ent-(S) t_r = 18.52 min and ent-(R) t_r = 19.72 min

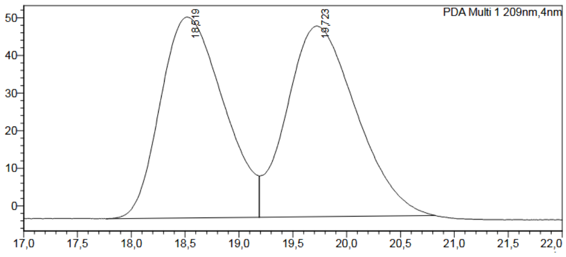

Peak#	Ret. Time	Area	Height	Mark	Area%
1	18,519	2182075	53373	M	48,911
2	19,723	2279223	50644	V M	51,089
Total		4461298	104017		100,000

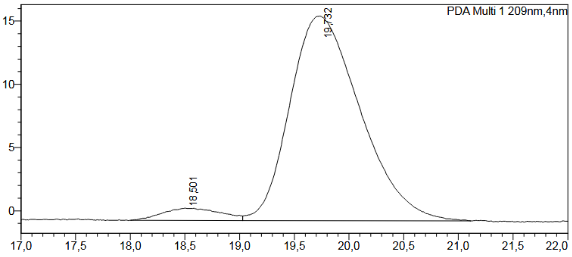

Peak#	Ret. Time	Area	Height	Mark	Area%
1	18,501	36364	961		4,771
2	19,732	725892	16166	SV	95,229
Total		762256	17127		100,000

Spectral and chromatographic data of compound **4b** were reported in our pervious publication: Kopyt, M.; Tryniszewski, M.; Barbasiewicz, M.; Kwiatkowski, P. *Org. Lett.* **2023**, *25*, 6818-6822.

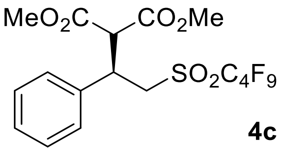

4c

Prepared according to the general procedure from nonaflone **1r** (272 mg, 0.70 mmol, c = 1M), catalyst **2d** (15 mg, 0.035 mmol, 5 mol%), and dimethyl malonate (140 mg, 1.06 mmol, 1.5 equiv) in toluene at +5 °C. After 2 h, conversion (^{19}F NMR) was 68%. After 20 h, reaction mixture was directly purified using column chromatography on silica gel (eluent: hexanes/ethyl acetate 100:0 to 85:15) to afford **4c** (332 mg, 0.64 mmol, 91%, 91% ee) as a white solid.

^{1}H NMR (400 MHz, $CDCl_3$): δ 7.40-7.24 (m, 5H), 4.21-4.09 (m, 2H), 3.90 (dd, *J* = 14.0, 9.7 Hz, 1H), 3.85 (d, *J* = 7.6 Hz, 1H), 3.74 (s, 3H), 3.61 (s, 3H).

^{19}F NMR (376 MHz, $CDCl_3$): δ -80.5 (t, *J* = 9.7 Hz, 3F), -113.3 (t, *J* = 13.9 Hz, 2F), -121.1 (dqd, *J* = 12.1, 9.0, 8.5, 4.4 Hz, 2F), -125.7 (tt, *J* = 13.6, 4.0 Hz, 2F).

^{13}C NMR (100 MHz, $CDCl_3$): δ 167.8, 167.1, 136.9, 129.0, 128.5, 127.9, 118.9-104.8 (m, 4C), 56.4, 53.4, 52.9, 52.8, 38.6.

HRMS (ESI) m/z: $[M+H]^+$ Calcd for $C_{17}H_{15}F_9O_6S$: 519.0518; Found 519.0522.

$[\alpha]_D^{25}$ = +8.6 (c 1.02, $CHCl_3$, 91% ee).

Enantiomeric excess was determined by HPLC analysis using a Phenomenex Lux® Amylose-1 column (eluent: hexane/i-PrOH 95:5, flow rate 1.0 mL/min, λ = 209 nm): ent-(R) t_r = 4.28 min and ent-(S) t_r = 4.96 min.

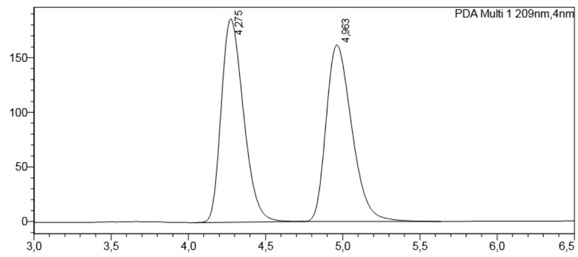

Peak#	Ret. Time	Area	Height	Mark	Area%
1	4,275	1872046	186260	M	49,937
2	4,963	1876780	161713	M	50,063
Total		3748826	347973		100,000

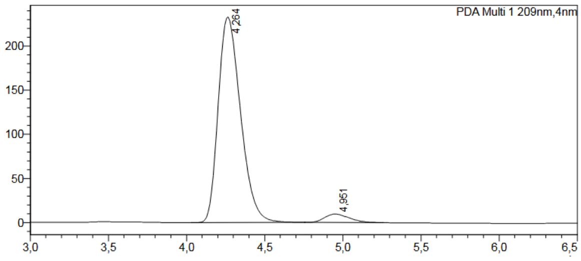

Peak#	Ret. Time	Area	Height	Mark	Area%
1	4,264	2323873	233117	M	95,714
2	4,951	104061	9449	M	4,286
Total		2427934	242566		100,000

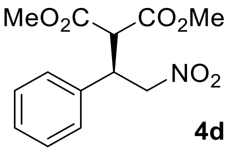

4d

Prepared according to general procedure from nitrostyrene **1s** (149 mg, 1 mmol, c = 0.5M; crystallized from MeOH before use), dimethyl malonate (200 mg, 1.5 mmol, 1.5 equiv) and catalyst **2d** (23 mg, 0.052 mmol, 5 mol%) in toluene at +5 °C. After 2 h, reaction mixture was directly purified using column chromatography on silica gel (eluent: hexanes/ethyl acetate 100:0 to 90:10) to afford **4d** (246 mg, 0.88 mmol, 88%, >99% conv. (^{1}H NMR), 97% ee) as a white solid.

Reaction conducted at rt for 2 h at c[**4d**] = 0.5M gave 96% yield (^{1}H NMR), >99% conv. (^{1}H NMR) and 95% ee.

^{1}H NMR (400 MHz, $CDCl_3$) δ 7.37 – 7.20 (m, 5H), 4.96 – 4.84 (m, 2H), 4.25 (td, J = 8.9, 5.1 Hz, 1H), 3.87 (d, J = 9.0 Hz, 1H), 3.76 (s, 3H), 3.56 (s, 3H).

^{13}C NMR (100 MHz, $CDCl_3$): δ 167.8, 167.2, 136.0, 129.4, 129.0, 128.4, 127.8, 54.7, 53.0, 52.8, 42.9.

^{1}H NMR spectra matched literature: Okino, T.; Hoashi, Y.; Furukawa, T.; Xu, X.; Takemoto, Y. *J. Am. Chem. Soc.* **2005**, 127 (1), 119–125.

Enantiomeric excess was determined by HPLC analysis using a Phenomenex Lux® Cellulose-2 column (eluent: hexane/i-PrOH 90:10, flow rate 1.0 mL/min, λ = 209 nm): ent-(R) t_r = 4.28 min and ent-(S) t_r = 4.96 min.

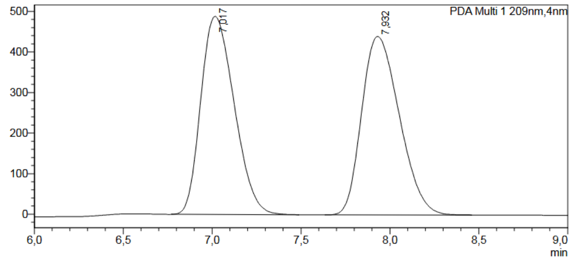

Peak#	Ret. Time	Area	Height	Mark	Area%
1	7,017	6396666	488578	M	49,602
2	7,932	6499329	440012	M	50,398
Total		12895994	928590		100,000

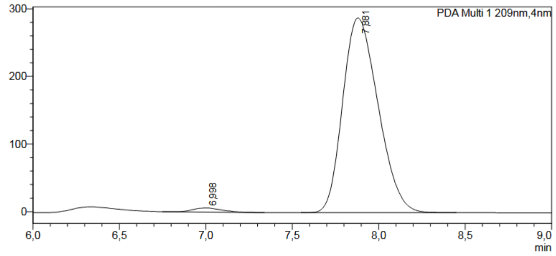

Peak#	Ret. Time	Area	Height	Mark	Area%
1	6,998	68139	6004	M	1,635
2	7,881	4098153	287997	M	98,365
Total		4166292	294002		100,000

IV.6. Analytical data for adducts 5a-h

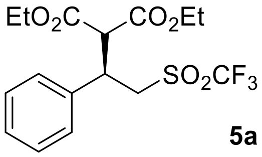

5a

Prepared according to the general procedure from triflone **1a** (165 mg, 0.70 mmol, c = 1M), catalyst **2d** (16 mg, 0.035 mmol, 5 mol%), and diethyl malonate (168 mg, 1.05 mmol, 1.5 equiv) in toluene at +5 °C. After 20 h, conversion (^{19}F NMR) was 82%. After 2 days, reaction mixture was directly purified using column chromatography on silica gel (eluent: hexanes/ethyl acetate 100:0 to 85:15) to afford **5a** (260 mg, 0.66 mmol, 93%, 90% ee) as a white solid.

^{1}H NMR (400 MHz, $CDCl_3$): δ 7.38-7.28 (m, 5H), 4.21 (p, *J* = 7.2 Hz, 2H), 4.16-4.01 (m, 4H), 3.86 (dd, *J* = 14.4, 10.2 Hz, 1H), 3.78 (d, *J* = 7.9 Hz, 1H), 1.24 (t, *J* = 7.1 Hz, 3H), 1.10 (t, *J* = 7.1 Hz, 3H).

^{19}F NMR (376 MHz, $CDCl_3$): δ -78.8 (s).

^{13}C NMR (100 MHz, $CDCl_3$): δ 167.4, 166.7, 136.9, 128.9, 128.5, 128.1, 119.1 (q, *J*=327 Hz), 62.2, 62.0, 56.7, 52.4, 38.6, 13.9, 13.7.

HRMS (ESI) m/z: $[M+H]^+$ Calcd for $C_{16}H_{19}F_3O_6S$: 397.0927; Found 397.0923.

$[\alpha]_D^{25}$ = +5.8 (c 0.98, $CHCl_3$, 90% ee).

Enantiomeric excess was determined by HPLC analysis using a Phenomenex Lux® Amylose-1 column (eluent: hexane/i-PrOH 90:10, flow rate 1.0 mL/min, λ = 220 nm): ent-(R) t_r = 5.17 min and ent-(S) t_r = 11.36 min.

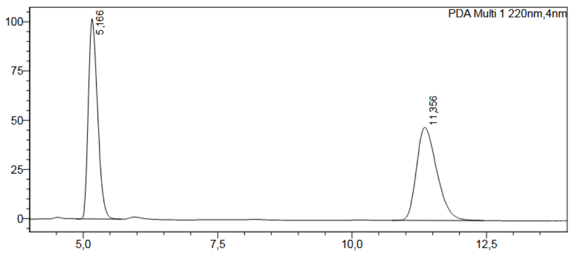

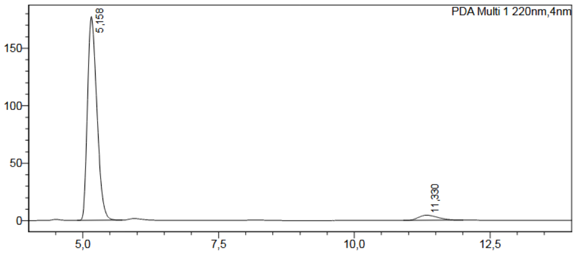

Peak#	Ret. Time	Area	Height	Mark	Area%
1	5,166	1222655	101780	M	49,655
2	11,356	1239657	47226	M	50,345
Total		2462312	149007		100,000

Peak#	Ret. Time	Area	Height	Mark	Area%
1	5,158	2149576	177213		95,055
2	11,330	111823	4445		4,945
Total		2261399	181658		100,000

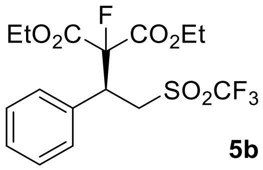

5b

Prepared according to the general procedure from triflone **1a** (165 mg, 0.70 mmol, c = 1M), catalyst **2d** (16 mg, 0.035 mmol, 5 mol%), diethyl fluoromalonate (187 mg, 1.05 mmol, 1.5 equiv) with $NaHCO_3$ additive (57 mg, 0.70 mmol, 1 equiv). After 20 h, ^{19}F NMR conversion was 58%. (Without $NaHCO_3$ additive, conversion reaches approx. 40% and reaction does not progress further). After 4 days, reaction mixture was directly purified using column chromatography on silica gel (eluent: hexanes/ethyl acetate 100:0 to 90:10) to afford **5b** (218 mg, 0.53 mmol, 76%, 93% ee) as a white solid.

High pressure experiment: small scale reaction using triflone **1a** (12 mg, 0.051 mmol, c = 0.5M), diethyl fluoromalonate (13.5 mg, 0.076 mmol, 1.5 equiv), catalyst **2d** (1.1 mg, 0.0025 mmol, 5 mol%) in toluene (0.075 ml, 0.1 ml total volume) conducted under high pressure conditions (9 kbar, 2 h) provided **5b** with >99% conversion (^{19}F NMR) and 91% ee.

^1H NMR (400 MHz, $CDCl_3$): δ 7.38-7.33 (m, 5H), 4.47-4.33 (m, 3H), 4.08-3.94 (m, 2H), 3.86-3.75 (m, 2H), 1.35 (t, *J* = 7.1 Hz, 3H), 1.02 (t, *J* = 7.1 Hz, 3H).

^{19}F NMR (376 MHz, $CDCl_3$): δ -78.6 (s), -172.4 (d, *J* = 29.4 Hz).

^{13}C NMR (100 MHz, $CDCl_3$): δ 164.4 (d, *J* = 25.2 Hz), 163.5 (d, *J* = 25.9 Hz), 133.5, 129.3 (d, *J* = 2.2 Hz), 129.1, 128.8, 119.1 (q, *J* = 327 Hz), 96.0 (d, *J* = 207 Hz), 63.8, 63.0, 51.1 (d, *J* = 6.1 Hz), 43.0 (d, *J* = 19.3 Hz), 13.8, 13.6.

HRMS (ESI) m/z: $[M+H]^+$ Calcd for $C_{16}H_{18}F_4O_4S$: 415.0833; Found 415.0833.

$[M+Na]^+$ Calcd for $C_{16}H_{18}F_4O_4S$: 437.0652; Found 437.0652.

$[\alpha]_D^{25}$ = -18.2 (c 1.02, $CHCl_3$, 93% ee).

Enantiomeric excess was determined by HPLC analysis using a Phenomenex Lux® Cellulose-2 column (eluent: hexane/i-PrOH 90:10, flow rate 1.0 mL/min, λ = 220 nm): ent-(R) t_r = 3.20 min and ent-(S) t_r = 5.09 min.

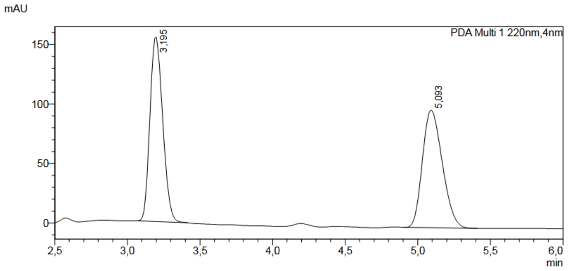

Peak#	Ret. Time	Area	Height	Mark	Area%
1	3,195	950175	154657	M	50,220
2	5,093	941856	98628		49,780
Total		1892031	253284		100,000

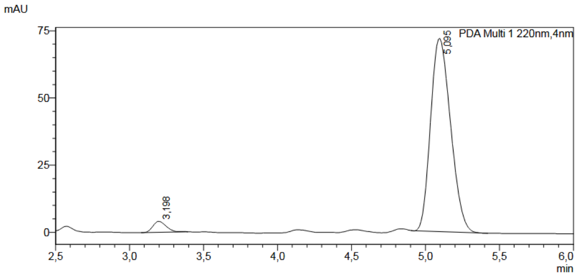

Peak#	Ret. Time	Area	Height	Mark	Area%
1	3,198	25612	4077	M	3,636
2	5,095	678733	71930	M	96,364
Total		704344	76007		100,000

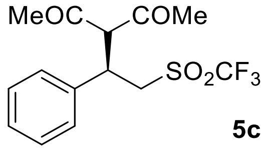

5c

Prepared according to the general procedure from triflone **1a** (71 mg, 0.30 mmol, c = 1M), catalyst **2d** (6.5 mg, 0.015 mmol, 5 mol%) and acetylacetone (45 mg, 0.45 mmol, 1.5 equiv) in toluene at +5 °C. After 20 h, reaction mixture was directly purified using column chromatography on silica gel (eluent: hexanes/ethyl acetate 100:0 to 85:15) to afford **5c** (88 mg, 0.26 mmol, 87%, 91% ee) as a white solid.

^{1}H NMR (400 MHz, $CDCl_3$): δ 7.40-7.25 (m, 5H), 4.29 (d, *J* = 9.4 Hz, 1H), 4.18 (td, *J* = 9.0, 4.4 Hz, 1H), 3.73-3.59 (m, 2H), 2.22 (s, 3H), 1.97 (s, 3H).

^{19}F NMR (376 MHz, $CDCl_3$): δ -78.8 (s).

^{13}C NMR (100 MHz, $CDCl_3$): δ 201.9, 201.2, 136.7, 129.2, 128.6, 128.1, 119.1 (q, *J* = 328 Hz), 72.0, 52.4, 38.4, 30.5, 30.1.

HRMS (ESI) m/z: $[M+H]^+$ Calcd for $C_{14}H_{15}F_3O_4S$: 337.0716; Found 337.0714.

$[M+Na]^+$ Calcd for $C_{14}H_{15}F_3O_4S$: 359.0535; Found 359.0533.

$[\alpha]_D^{25}$ = -80.3 (c 1.03, $CHCl_3$, 91% ee).

Enantiomeric excess was determined by HPLC analysis using a Phenomenex Lux® Cellulose-1 column (eluent: hexane/i-PrOH 90:10, flow rate 1.0 mL/min, λ = 220 nm): ent-(S) t_r = 5.95 min and ent-(R) t_r = 6.31 min.

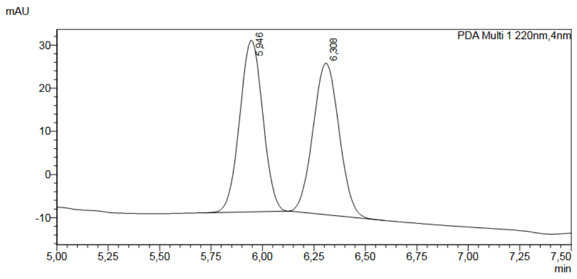

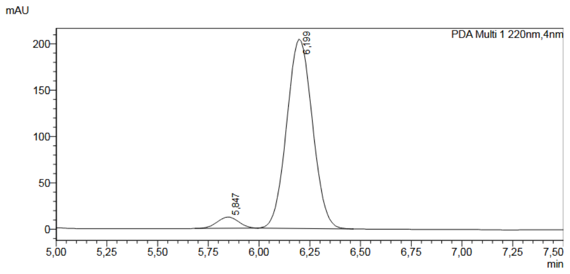

Peak#	Ret. Time	Area	Height	Mark	Area%
1	5,946	310911	39742	M	50,151
2	6,308	309043	35128	M	49,849
Total		619954	74870		100,000

Peak#	Ret. Time	Area	Height	Mark	Area%
1	5,847	88564	11969	M	4,683
2	6,199	1802479	204410	M	95,317
Total		1891043	216379		100,000

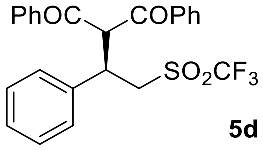

5d

Prepared according to the general procedure from triflone **2a** (118 mg, 0.50 mmol, c = 1M), catalyst **2d** (11 mg, 0.025 mmol, 5 mol%) and benzoylacetophenone (168 mg, 0.75 mmol, 1.5 equiv) in toluene at +5 °C. After 2 days, reaction mixture was directly purified using column chromatography on silica gel (eluent: hexanes/ethyl acetate 100:0 to 85:15) to afford **5d** (170 mg, 0.37 mmol, 74%; 79% ee for derivative **13**) as a white solid.

Separation of enantiomers of compound **5d** using available chiral HPLC columns was unsuccessful, therefore enantiomeric excess was determined after cyclization to oxazole **13**. (see page S-69).

High pressure reaction: 0.5 ml Teflon vial was loaded with triflone **1a** (59 mg, 0.250 mmol, c = 0.5M), benzoylacetophenone (84 mg, 0.375 mmol, 1.5 equiv), catalyst **2d** (5.5 mg, 0.0125 mmol, 5 mol%) and filled up with toluene (0.35 ml) then subjected to high pressure conditions (9 kbar for 2 h) to obtain **5d** (87 mg, 0.189 mmol, 75%, 54% ee for **13**)

^{1}H NMR (400 MHz, $CDCl_3$): δ 7.81 (d, *J* = 7.9 Hz, 2H), 7.74 (d, *J* = 8.0 Hz, 2H), 7.56-7.49 (m, 2H), 7.37 (dt, *J* = 12.0, 7.7 Hz, 4H), 7.30-7.15 (m, 5H), 5.75 (d, *J* = 6.0 Hz, 1H), 4.41 (ddd, *J* = 9.8, 6.1, 2.9 Hz, 1H), 4.23 (dd, *J* = 14.7, 2.9 Hz, 1H), 4.02 (dd, *J* = 14.7, 10.5 Hz, 1H).

^{19}F NMR (376 MHz, $CDCl_3$): δ -78.7 (s).

^{13}C NMR (100 MHz, $CDCl_3$): δ 194.4, 193.9, 137.3, 136.0, 135.3, 134.1, 134.0, 128.92, 128.91, 128.6, 128.5, 128.4, 128.3, 119.2 (q, *J* = 327 Hz), 60.7, 51.6, 39.7.

HRMS (ESI) m/z: $[M+H]^+$ Calcd for $C_{24}H_{19}F_3O_4S$: 461.1029; Found 461.1026.

$[\alpha]_D^{25}$ = -8.5 (c 0.77, $CHCl_3$, 79% ee).

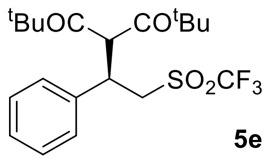

5e

High-pressure reaction: A 0.9 mL teflon vial was loaded with triflone **1a** (106 mg, 0.45 mmol, c = 0.5M), dipivaloylmethane (125 mg, 0.675 mmol, 1.5 equiv), catalyst **2d** (10 mg, 0.022 mmol, 5 mol%), filled up with toluene, and homogenous reaction mixture was subjected to 9 kbar of hydrostatic pressure for 2 h. After decompression reaction mixture was directly purified on chromatographic column (silica gel, eluent: hexanes/ethyl acetate 100:0 to 95:5) to afford **5e** (177 mg, 0.42 mmol, 94%, >99% conv. ^{19}F NMR, 88% ee) as a white solid.

Reaction conducted under atmospheric pressure using triflone **1a** (71 mg, 0.30 mmol, c = 1M), dipivaloylmethane (83 mg, 0.45 mmol, 1.5 equiv), catalyst **2d** (6.5 mg, 0.015 mmol, 5 mol%) in toluene (0.140 ml, 0.3 ml total volume) provided **5e** with conversion (^{19}F NMR) 11% after 20 h and 45% after 5 days. Purification after 5 d provided **5e** (47 mg, 0.112 mmol, 36%, 92% ee).

^{1}H NMR (400 MHz, $CDCl_3$): δ 7.37-7.23 (m, 5H), 4.93 (d, *J* = 3.4 Hz, 1H), 4.59 (dd, *J* = 15.3, 11.0 Hz, 1H), 3.98 (dd, *J* = 10.9, 3.2 Hz, 1H), 3.83 (dd, *J* = 15.2, 1.6 Hz, 1H), 1.38 (s, 9H), 0.77 (s, 9H).

^{19}F NMR (376 MHz, $CDCl_3$): δ -78.9 (s).

^{13}C NMR (100 MHz, $CDCl_3$): δ 209.4, 209.0, 137.9, 129.1, 128.5, 127.6, 119.1 (q, *J* = 327 Hz), 59.5, 49.6, 45.5, 44.8, 38.9, 28.1, 25.5.

HRMS (ESI) m/z: $[M+H]^+$ Calcd for $C_{20}H_{27}F_3O_4S$: 421.1655; Found 421.1653.

$[M+Na]^+$ Calcd for $C_{20}H_{27}F_3O_4S$: 443.1474; Found 443.1474.

$[\alpha]_D^{25}$ = -152.3 (c 0.99, $CHCl_3$, 88% ee).

Enantiomeric excess was determined by HPLC analysis using a Phenomenex Lux® Cellulose-4 column (eluent: hexane/i-PrOH 99:1, flow rate 1.0 mL/min, λ = 209 nm): ent-(R) t_r = 3.80 min and ent-(S) t_r = 4.29 min.

Racemate

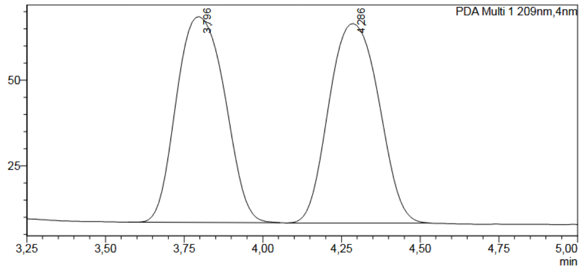

Peak#	Ret. Time	Area	Height	Mark	Area%
1	3,796	643189	60032	M	49,946
2	4,286	644587	58172	M	50,054
Total		1287776	118205		100,000

Reaction ran under 9 kbar (isolated)

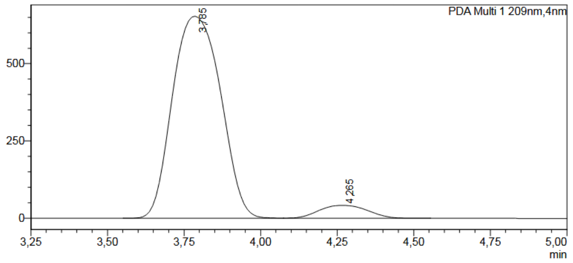

PDA Ch1 209nm

Peak#	Ret. Time	Area	Height	Mark	Area%
1	3,785	7160957	653377	M	93,895
2	4,265	465564	41922	V M	6,105
Total		7626521	695299		100,000

Reaction ran under atmospheric pressure

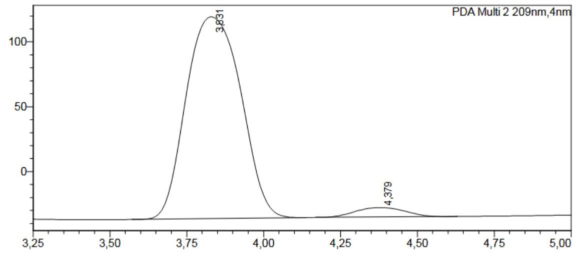

Peak#	Ret. Time	Area	Height	Mark	Area%
1	3,831	1929102	155608	M	95,911
2	4,379	82248	7258	M	4,089
Total		2011350	162865		100,000

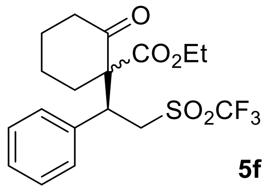

5f

Prepared according to the general procedure from triflone **1a** (165 mg, 0.70 mmol, c = 1M), catalyst **2d** (16 mg, 0.035 mmol, 5 mol%) and ethyl 2-oxocyclohexanecarboxylate (178 mg, 1.05 mmol, 1.5 equiv) in toluene at +5 °C. After 20 h, conversion (^{19}F NMR) was 41%. After 4 days, reaction mixture was directly purified using column chromatography on silica gel (eluent: hexanes/ethyl acetate 100:0 to 85:15) to afford **5f** (207 mg, 0.51 mmol, 73%, >20:1 dr, 91% ee (major)/50% ee (minor)) as a colourless oil..

High pressure reaction: Small scale reaction using triflone **1a** (12 mg, 0.051 mmol, c = 0.5M), ethyl 2-oxocyclohexanecarboxylate (13 mg, 0.076 mmol, 1.5 equiv), catalyst **2d** (1.1 mg, 0.0025 mmol, 5 mol%) and toluene (0.075 ml, 0.1 ml total volume) conducted under high pressure conditions (9 kbar, 2 h) provided **5f** with 98% yield (^{19}F NMR), >20:1 dr, 87% ee (major)/55% ee (minor).

^{1}H NMR (400 MHz, $CDCl_3$): δ 7.34-7.29 (m, 3H), 7.21-7.17 (m, 2H), 4.18 (q, *J* = 7.1 Hz, 2H), 4.09-4.00 (m, 1H), 3.78 (dd, *J* = 6.3, 2.7 Hz, 2H), 2.56-2.41 (m, 2H), 2.12 (dq, *J* = 13.5, 3.2 Hz, 1H), 2.03 (ddq, *J* = 12.1, 6.0, 3.1 Hz, 1H), 1.79-1.70 (m, 1H), 1.70-1.55 (m, 2H), 1.48 (td, *J* = 12.8, 3.9 Hz, 1H), 1.22 (t, *J* = 7.1 Hz, 3H).

^{19}F NMR (376 MHz, $CDCl_3$): δ -78.9 (s).

^{13}C NMR (100 MHz, $CDCl_3$): δ 207.2, 169.4, 135.9, 129.5, 128.4, 128.4, 119.2 (q, *J* = 328 Hz), 64.1, 62.0, 52.9 (d, *J* = 1.1 Hz), 43.4, 41.4, 36.8, 27.8, 22.4, 13.9.

HRMS (ESI) m/z: $[M+H]^+$ Calcd for $C_{18}H_{21}F_3O_5S$: 407.1135; Found 407.1133.

$[M+Na]^+$ Calcd for $C_{18}H_{21}F_3O_5S$: 429.0954; Found 429.0954.

$[\alpha]_D^{25}$ = +67.1 (c 1.07, $CHCl_3$, >20:1 dr, 91%/50% ee).

Enantiomeric excess was determined by HPLC analysis using a Phenomenex Lux® Cellulose-4 column (eluent: hexane/i-PrOH 96:4, flow rate 1.0 mL/min, λ = 209 nm): major pair of diastereoisomers: 1-(R): t_r = 4.61 min, 1-(S): t_r = 5.87 min; minor pair of diastereoisomers: 1-(R): t_r = 3.80 min, 1-(S): t_r = 4.27 min

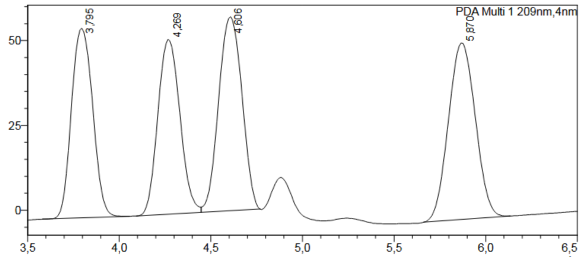

Peak#	Ret. Time	Area	Height	Mark	Area%
1	3,795	451197	55839	M	23,459
2	4,269	433769	51550	M	22,553
3	4,606	505882	57164	V M	26,302
4	5,870	532494	52069	M	27,686
Total		1923343	216622		100,000

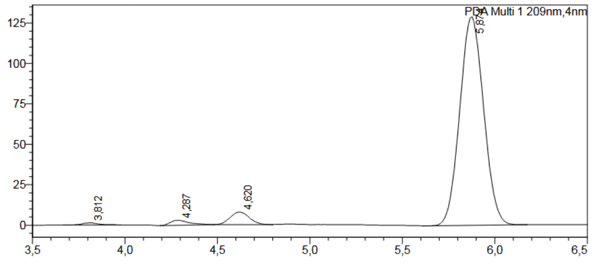

Peak#	Ret. Time	Area	Height	Mark	Area%
1	3,812	7545	1267	M	0,607
2	4,287	22771	3111	M	1,831
3	4,620	55031	7656	M	4,425
4	5,874	1158314	128934	M	93,137
Total		1243661	140968		100,000

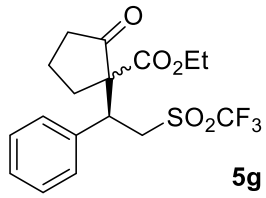

5g

Prepared according to the general procedure from triflone **1a** (71 mg, 0.30 mmol, c = 1M), catalyst **2d** (6.5 mg, 0.015 mmol, 5 mol%) and ethyl 2-oxocyclopentanecarboxylate (70 mg, 0.45 mmol, 1.5 equiv) in toluene at +5 °C. After 20 h, reaction mixture was directly purified using column chromatography on silica gel (eluent: hexanes/ethyl acetate 100:0 to 85:15) to afford **5g** (100 mg, 0.25 mmol, 85%, 10.5:1 dr, 88% ee (major)/78% ee (minor)) as a colourless oil.

^{1}H NMR (400 MHz, $CDCl_3$): δ 7.36-7.29 (m, 5H), 4.34 (dd, *J* = 14.8, 2.1 Hz, 1H), 4.21 (qd, *J* = 7.1, 2.2 Hz, 2H), 4.03 (ddd, *J* = 14.8, 11.1, 0.9 Hz, 1H), 3.78 (dd, *J* = 11.1, 2.1 Hz, 1H), 2.45-2.29 (m, 2H), 2.19-2.08 (m, 1H), 2.01-1.83 (m, 3H), 1.26 (t, *J* = 7.1 Hz, 3H).

^{19}F NMR (376 MHz, $CDCl_3$): δ -78.8 (s, major), -78.9 (s, minor)

^{13}C NMR (100 MHz, $CDCl_3$): δ 212.3, 169.2, 136.0, 129.4, 128.8, 128.5, 119.2 (q, *J* = 328 Hz), 63.0, 62.2, 51.5, 41.8, 37.9, 31.6, 19.1, 13.9.

HRMS (ESI) m/z: $[M+H]^+$ Calcd for $C_{17}H_{19}F_3O_5S$: 393.0978; Found 393.0976.

$[M+Na]^+$ Calcd for $C_{17}H_{19}F_3O_5S$: 415.0798; Found 415.0797.

$[\alpha]_D^{25}$ = -19.2 (c 1.02, $CHCl_3$, 10.5:1 dr, 88%/78% ee).

Enantiomeric excess was determined by HPLC analysis using a Phenomenex Lux® Cellulose-2 column (eluent: hexane/i-PrOH 95:5, flow rate 1.0 mL/min, λ = 209 nm): major pair of diastereoisomers: 1-(S): t_r = 4.77 min, 1-(R): t_r = 6.32 min, minor pair of diastereoisomers: 1-(R): t_r = 4.24 min, 1-(S): t_r = 5.29 min.

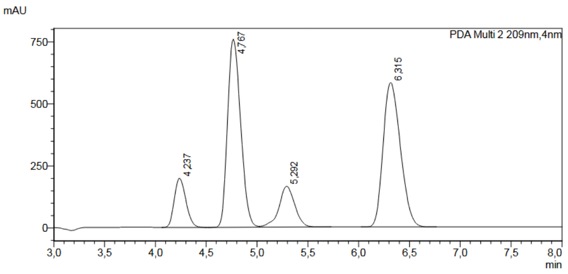

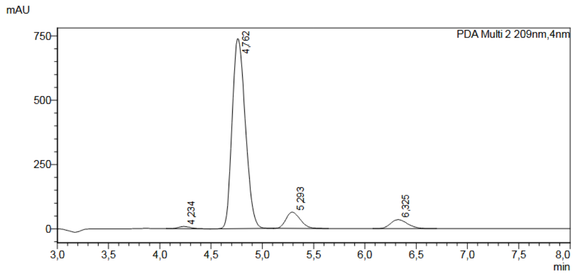

Peak#	Ret. Time	Area	Height	Mark	Area%
1	4,237	1569733	198184	M	9,198
2	4,767	6812351	758131	M	39,917
3	5,292	1793558	164319	V M	10,509
4	6,315	6890709	582833	M	40,376
Total		17066351	1703467		100,000

Peak#	Ret. Time	Area	Height	Mark	Area%
1	4,234	77816	9049	M	1,005
2	4,762	6643145	737868	M	85,754
3	5,293	616426	63242	M	7,957
4	6,325	409390	35306	M	5,285
Total		7746778	845465		100,000

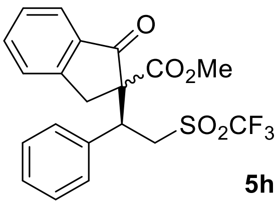

5h

Prepared according to the general procedure from triflone **1a** (168 mg, 0.71 mmol, c = 1M), catalyst **2d** (16 mg, 0.035 mmol, 5 mol%) and 2-methoxycarbonyl-1-indanone (200 mg, 1.05 mmol, 1.5 equiv) in toluene at +5 °C. After 20 h, reaction mixture was directly purified using column chromatography on silica gel (eluent: hexanes/ethyl acetate 100:0 to 80:20) to afford **5h** (288 mg, 0.68 mmol, 95%, 1.6:1 dr, 88% ee (major)/78% ee (minor)) as a thick yellow oil.

^{1}H NMR (400 MHz, $CDCl_3$): δ 7.77 (d, *J* = 7.7 Hz, 1H major), 7.71 (d, *J* = 7.7 Hz, 1H minor), 7.61 (t, *J* = 7.5 Hz, 1H minor), 7.49 (t, *J* = 7.5 Hz, 1H major), 7.42-7.25 (m, 3H major + 4H minor), 7.18 (d, *J* = 7.8 Hz, 1H major), 7.14 (s, 3H major + 3H minor), 4.71 (d, *J* = 14.7 Hz, 1H minor), 4.31-4.16 (m, 3H major + 1H minor), 3.85 (d, *J* = 10.9 Hz, 1H minor), 3.74 (s, 3H minor), 3.72 (s, 3H major), 3.58 (d, *J* = 17.7 Hz, 1H minor), 3.44 (d, *J* = 17.5 Hz, 1H major), 3.19 (d, *J* = 17.9 Hz, 1H minor), 3.13 (d, *J* = 17.5 Hz, 1H major).

^{19}F NMR (376 MHz, $CDCl_3$): δ -78.8 (s, minor), -78.9 (s, major).

^{13}C NMR (100 MHz, $CDCl_3$): δ 202.3 (major), 199.9 (minor), 171.3 (major), 169.9 (minor), 152.4 (major), 152.2 (minor), 136.7 (minor), 136.3 (major), 136.0 (minor), 135.8 (major), 134.9 (major), 133.8 (minor), 129.1, 129.1, 128.9, 128.6, 128.5, 128.5, 128.2 (minor), 128.0 (major), 126.1 (minor), 126.0 (major), 125.3 (minor), 124.4 (major), 119.2 (q, *J* = 328 Hz), 63.2 (minor), 62.0 (major), 53.3 (major), 53.2 (minor), 52.6 (major), 51.9 (minor), 43.0 (major), 42.9 (minor), 36.6 (major), 35.6 (minor).

HRMS (ESI) m/z: $[M+H]^+$ Calcd for $C_{20}H_{17}F_3O_5S$: 427.0822; Found 427.0818.

$[\alpha]_D^{25}$ = +30.7 (c 0.96, $CHCl_3$, 1.6:1 dr, 88%/78% ee).

Enantiomeric excess was determined by HPLC analysis using a Phenomenex Lux® Cellulose-4 column (eluent: hexane/i-PrOH 95:5, flow rate 1.0 mL/min, λ = 245 nm): major pair of diastereoisomers: 1-(S): t_r = 7.34 min, 1-(R): t_r = 8.36 min, minor pair of diastereoisomers: 1-(R): t_r = 7.84 min, 1-(S): t_r = 8.94 min.

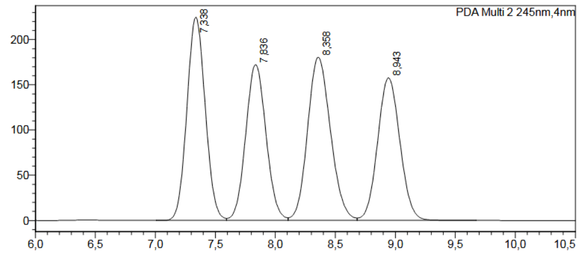

Peak#	Ret. Time	Area	Height	Mark	Area%
1	7,338	2396472	224807		26,985
2	7,836	2037455	172341	V	22,942
3	8,358	2402966	180332	V	27,058
4	8,943	2043851	157711	V	23,014
Total		8880744	735192		100,000

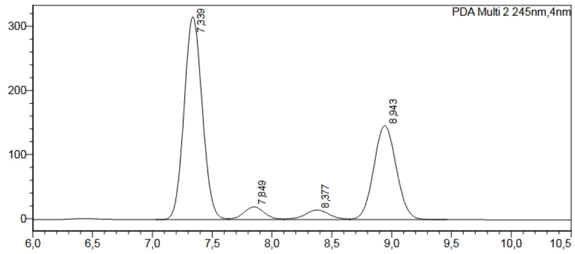

Peak#	Ret. Time	Area	Height	Mark	Area%
1	7,339	3346659	316353	M	58,387
2	7,849	239178	19834	V M	4,173
3	8,377	217700	15035	V M	3,798
4	8,943	1928360	146650	V M	33,643
Total		5731897	497872		100,000

IV.7. Analytical data for adducts 6a-e

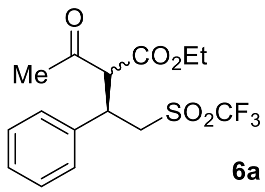

6a

Prepared according to the general procedure from triflone **1a** (242 mg, 1.02 mmol, c = 1M), catalyst **2d** (22 mg, 0.050 mmol, 5 mol%) and ethyl acetoacetate (195 mg, 1.50 mmol, 1.5 equiv) in toluene at +5 °C. After 20 h, reaction mixture was directly purified using column chromatography on silica gel (eluent: hexanes/ethyl acetate 100:0 to 85:15) to afford **6a** (337 mg, 0.92 mmol, 90%, 1.25:1 dr; 90% ee after decarboxylation to compound **7a**) as a colourless oil.

Separation of diastereoisomers of compound **6a** using available chiral HPLC columns was unsuccessful, therefore enantiomeric excess was determined after decarboxylation to compound **7a** (see page S-57).

^{1}H NMR (400 MHz, $CDCl_3$): δ 7.38-7.25 (m, 5H major + 5H minor), 4.21 (qd, *J* = 7.1, 2.6 Hz, 2H minor), 4.18-4.10 (m, 2H major), 4.04-3.98 (m, 1H major + 3H minor), 3.97-3.88 (m, 1H major + 1H minor), 3.78 (ddd, *J* = 14.4, 9.7, 5.9 Hz, 2H major), 2.22 (s, 3H major), 2.07 (s, 3H minor), 1.25 (t, *J* = 7.1 Hz, 3H minor), 1.06 (t, *J* = 7.1 Hz, 3H major).

^{19}F NMR (376 MHz, $CDCl_3$): δ -78.8 (s, minor), -78.9 (s, major).

Major diastereoisomer: **^{13}C NMR** (100 MHz, $CDCl_3$): δ 201.4, 166.8, 137.0, 128.9, 128.4, 128.2, 119.1 (q, *J* = 327 Hz), 63.5, 62.1, 52.3, 38.1, 30.3, 13.7.

Minor diastereoisomer: **^{13}C NMR** (100 MHz, $CDCl_3$): δ 200.4, 167.5, 137.0, 129.1, 128.5, 128.1, 119.1 (q, *J* = 327 Hz), 63.2, 62.2, 52.4, 38.2, 30.3, 13.9.

HRMS (ESI) m/z: $[M+H]^+$ Calcd for $C_{15}H_{17}F_3O_5S$: 367.0822; Found 367.0822.

$[M+Na]^+$ Calcd for $C_{15}H_{17}F_3O_5S$: 389.0641; Found 389.0640.

$[\alpha]_D^{25}$ = -58.1 (c 0.99, $CHCl_3$, 1.25:1 dr, avg. 90% ee).

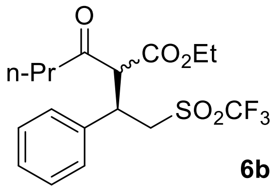

6b

Prepared according to the general procedure from triflone **1a** (165 mg, 0.70 mmol, c = 1M), catalyst **2d** (16 mg, 0.035 mmol, 5 mol%) and ethyl butyrylacetate (165 mg, 1.05 mmol, 1.5 equiv) in toluene at +5 °C. After 20 h, reaction mixture was directly purified using column chromatography on silica gel (eluent: hexanes/ethyl acetate 100:0 to 90:10) to afford **6b** (262 mg, 0.66 mmol, 95%, 1:1 dr; 92% ee after decarboxylation to compound **7b**) as a white solid.

Separation of diastereoisomers of compound **6b** using available chiral HPLC columns was unsuccessful, therefore enantiomeric excess was determined after decarboxylation to compound **7b** (see page S-58).

^{1}H NMR (400 MHz, $CDCl_3$): δ 7.37-7.25 (m, 2x5H), 4.20 (dtt, *J* = 10.7, 7.2, 3.6 Hz, 2H), 4.13 (ddd, *J* = 8.5, 5.4, 3.7 Hz, 2H), 4.06-3.90 (m, 6H), 3.80 (dt, *J* = 14.3, 10.8 Hz, 2H), 2.53 (dt, *J* = 17.7, 7.3 Hz, 1H), 2.43 (dt, *J* = 17.8, 7.1 Hz, 1H), 2.32 (dt, *J* = 17.8, 7.0 Hz, 1H), 2.14 (dt, *J* = 17.7, 7.1 Hz, 1H), 1.57 (h, *J* = 7.3 Hz, 2H), 1.41 (h, *J* = 7.3 Hz, 2H), 1.24 (t, *J* = 7.1 Hz, 3H), 1.08 (t, *J* = 7.1 Hz, 3H), 0.86 (t, *J* = 7.4 Hz, 3H), 0.71 (t, *J* = 7.4 Hz, 3H).

^{19}F NMR (376 MHz, $CDCl_3$): δ -78.8 (s), -78.9 (s).

^{13}C NMR (100 MHz, $CDCl_3$): δ 203.9, 202.6, 167.5, 166.9, 137.1, 137.0, 129.0, 128.9, 128.4, 128.3, 128.1, 128.1, 119.1 (q, *J* = 327 Hz), 62.8, 62.6, 62.1, 62.0, 52.5, 52.2, 45.5, 45.3, 38.4, 38.2, 16.6, 16.4, 13.9, 13.7, 13.3, 13.1.

HRMS (ESI) m/z: $[M+H]^+$ Calcd for $C_{17}H_{21}F_3O_5S$: 395.1135; Found 395.1132.

$[\alpha]_D^{25}$ = -13.4 (c 1.01, $CHCl_3$, 1:1 dr, avg. 92% ee).

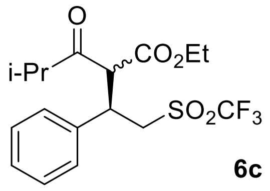

6c

Prepared according to the general procedure from triflone **1a** (165 mg, 0.70 mmol, c = 1M), catalyst **2d** (16 mg, 0.035 mmol, 5 mol%) and ethyl isobutyrylacetate (165 mg, 1.05 mmol, 1.5 equiv) in toluene at +5 °C. After 20 h, reaction mixture was directly purified using column chromatography on silica gel (eluent: hexanes/ethyl acetate 100:0 to 90:10) to afford **6c** (264 mg, 0.67 mmol, 96%, 1:1 dr; 91% ee after decarboxylation to compound **7c**) as a white solid.

Separation of diastereoisomers of compound **6c** using available chiral HPLC columns was unsuccessful, therefore enantiomeric excess was determined after decarboxylation to compound **7c** (see page S-59).

^{1}H NMR (400 MHz, $CDCl_3$): δ 7.37-7.25 (m, 2x5H), 4.25-3.90 (m, 11H), 3.81 (dd, *J* = 14.6, 9.1 Hz, 1H), 2.60 (hept, *J* = 7.1 Hz, 1H), 2.45 (hept, *J* = 6.8 Hz, 1H), 1.24 (t, *J* = 7.1 Hz, 3H), 1.10 (t, *J* = 7.1 Hz, 3H), 1.05 (d, *J* = 6.8 Hz, 3H), 1.01 (d, *J* = 6.9 Hz, 3H), 0.93 (d, *J* = 7.0 Hz, 3H), 0.75 (d, *J* = 6.9 Hz, 3H).

^{19}F NMR (376 MHz, $CDCl_3$): δ -78.8 (s), -78.9 (s).

^{13}C NMR (100 MHz, $CDCl_3$): δ 208.0, 206.5, 167.4, 167.0, 137.2, 137.0, 129.0, 128.9, 128.4, 128.2, 128.1, 119.1 (q, *J* = 328 Hz), 62.1, 62.1, 61.3, 61.1, 52.3, 51.9, 42.0, 41.6, 38.7, 38.4, 17.8, 17.7, 17.5, 17.3, 13.9, 13.7.

HRMS (ESI) m/z: $[M+H]^+$ Calcd for $C_{17}H_{21}F_3O_5S$: 395.1135; Found 395.1133.

$[\alpha]_D^{25}$ = -16.9 (c 1.05, $CHCl_3$, 1:1 dr, avg. 91% ee).

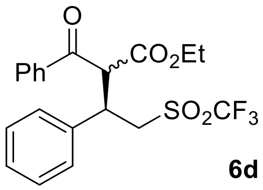

6d

Prepared according to the general procedure from triflone **1a** (165 mg, 0.70 mmol, c = 1M), catalyst **2d** (16 mg, 0.035 mmol, 5 mol%) and ethyl benzoylacetate (200 mg, 1.05 mmol, 1.5 equiv) in toluene at -15 °C. After 20 h, reaction mixture was directly purified using column chromatography on silica gel (eluent: hexanes/ethyl acetate 100:0 to 85:15) to afford **6d** (281 mg, 0.66 mmol, 94%, 1.4:1 dr, 92% ee (major)/92% ee (minor)) as a white solid.

Small-scale reaction conducted at +5 °C gave >99% conv. (^{19}F NMR) and 84% ee (major)/81% ee (minor).

^{1}H NMR (400 MHz, $CDCl_3$): δ 7.94 (dd, *J* = 8.3, 1.3 Hz, 2H major), 7.84 (dd, *J* = 8.3, 1.4 Hz, 2H minor), 7.62-7.53 (m, 1H major + 1H minor), 7.49-7.39 (m, 1H major + 1H minor) 7.35-7.20 (m, 6H major + 6H minor), 4.83 (d, *J* = 7.8 Hz, 1H minor), 4.82 (d, *J* = 8.4 Hz, 1H major) 4.39 (ddd, *J* = 11.1, 8.4, 3.3 Hz, 1H major), 4.29 (ddd, *J* = 10.7, 7.8, 3.0 Hz, 1H minor), 4.20-4.10 (m, 3H minor), 4.00-3.88 (m, 3H major + 1H minor), 3.81 (dd, *J* = 14.3, 10.2 Hz, 1H major), 1.15 (t, *J* = 7.1 Hz, 3H minor), 0.95 (t, *J* = 7.1 Hz, 3H major).

^{19}F NMR (376 MHz, $CDCl_3$): δ -78.8 (s).

Major diastereoisomer: **^{13}C NMR** (100 MHz, $CDCl_3$): δ 193.0, 167.0, 136.8, 135.9, 134.2, 128.9, 128.8, 128.5, 128.2, 119.2 (q, *J* = 328 Hz), 62.1, 58.9, 52.7, 38.9, 13.6.

Minor diastereoisomer: **^{13}C NMR** (100 MHz, $CDCl_3$): δ 192.9, 167.7, 137.5, 135.9, 134.0, 129.0, 128.8, 128.5, 128.3, 119.2 (q, *J* = 327 Hz), 62.3, 58.1, 52.5, 38.9, 13.8.

HRMS (ESI) m/z: $[M+H]^+$ Calcd for $C_{20}H_{19}F_3O_5S$ 429.0978; Found 429.0979.

$[M-H]^-$ Calcd for $C_{20}H_{19}F_3O_5S$: 427.0822; Found 427.0832.

$[\alpha]_D^{25}$ = +2.4 (c 1.04, $CHCl_3$, 1.4:1 dr, 92%/92% ee).

Enantiomeric excess was determined by HPLC analysis using a Phenomenex Lux® Amylose-1 column (eluent: hexane/i-PrOH 97:3, flow rate 1.0 mL/min, λ = 245 nm): major pair of diastereoisomers: 3-(S): t_r = 7.01 min, 3-(R): t_r = 12.93 min, minor pair of diastereoisomers: 3-(S): t_r = 11.99 min, 3-(R): t_r = 15.83 min.

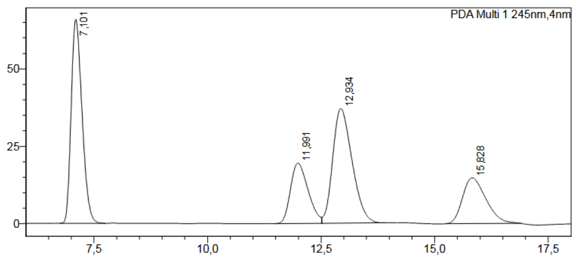

Peak#	Ret. Time	Area	Height	Mark	Area%
1	7,101	1096074	65834		33,794
2	11,991	522364	19437		16,105
3	12,934	1096452	37005	V	33,806
4	15,828	528523	14788	M	16,295
Total		3243413	137063		100,000

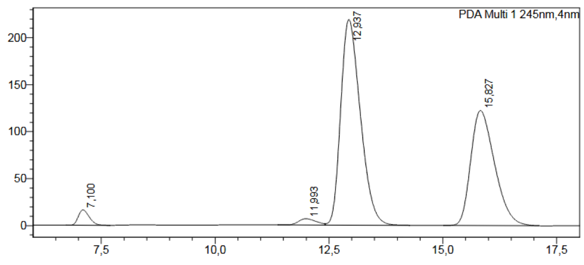

Peak#	Ret. Time	Area	Height	Mark	Area%
1	7,100	273943	16379	M	2,383
2	11,993	178488	6713	M	1,553
3	12,937	6571629	218946	V M	57,167
4	15,827	4471341	122558	M	38,897
Total		11495402	364597		100,000

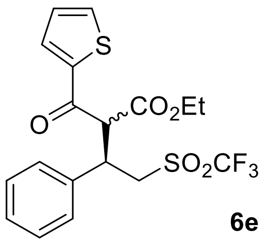

6e

Prepared according to the general procedure from triflone **1a** (165 mg, 0.70 mmol, c =1M), catalyst **2d** (16 mg, 0.035 mmol, 5 mol%) and ethyl 2-thiophenecarbonylacetate (208 mg, 1.05 mmol, 1.5 equiv) in toluene at -15 °C. After 20 h, reaction mixture was directly purified using column chromatography on silica gel (eluent: hexanes/ethyl acetate 100:0 to 85:15) to afford **6e** (295 mg, 0.68 mmol, 97%, 1.5:1 dr; 88% ee after decarboxylation to compound **7e**) as a white solid.

Separation of diastereoisomers of compound **6e** using available chiral HPLC columns was unsuccessful, therefore enantiomeric excess was determined after decarboxylation to compound **7e** (see page S-61).

Small-scale reaction conducted at +5 °C gave >99% conv. (^{19}F NMR) and 81% ee (for compound **7e**, measured after decarboxylation).

^{1}H NMR (400 MHz, $CDCl_3$): δ 7.75-7.71 (m, 2H major + 1H minor), 7.68 (dd, *J* = 4.9, 1.1 Hz, 1H minor), 7.34-7.29 (m, 5H major + 5H minor), 7.11 (ddd, *J* = 5.0, 3.9, 1.2 Hz, 1H major + 1H minor), 4.63 (d, *J* = 8.4 Hz, 1H major), 4.63 (dd, *J* = 8.1, 1.9 Hz, 1H minor), 4.36 (ddd, *J* = 11.1, 8.2, 3.1 Hz, 1H major), 4.28 (ddd, *J* = 10.7, 7.8, 2.9 Hz, 1H minor), 4.19 (q, *J* = 7.2 Hz, 2H minor), 4.14 (dd, *J* = 14.7, 3.1 Hz, 1H minor), 4.03-3.90 (m, 3H major + 1H minor), 3.80 (dd, *J* = 14.3, 10.5 Hz, 1H major), 1.19 (t, *J* = 7.1 Hz, 3H minor), 1.01 (t, *J* = 7.1 Hz, 3H major).

^{19}F NMR (376 MHz, $CDCl_3$): δ -78.2 (s, major), -78.2 (s, minor).

^{13}C NMR (100 MHz, $CDCl_3$): δ 185.3 (major), 184.9 (minor), 167.3 (major), 166.8 (minor), 143.0 (major), 142.7 (minor), 137.2 (major), 136.6 (minor), 136.1 (major), 135.77 (minor), 133.9 (major), 133.4 (minor), 129.0 (minor), 128.8 (major), 128.5, 128.4, 128.4, 128.3, 128.1, 119.1 (q, *J* = 327 Hz, minor), 119.1 (q, *J* = 328 Hz, major), 62.4 (minor), 62.2 (major), 59.9 (major), 59.4 (minor), 52.4, 39.0 (minor), 38.8 (major), 13.8 (minor), 13.6 (major).

HRMS (ESI) m/z: $[M+H]^+$ Calcd for $C_{18}H_{17}F_3O_5S_2$: 435.0542; Found 435.0540.

$[\alpha]_D^{25}$ = +11.3 (c 0.99, $CHCl_3$, 1.5:1 dr, avg. 88% ee).

V. Other transformations

V.1. Hydrolysis-decarboxylation sequence

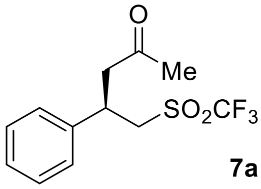

7a

Adduct **6a** (200 mg, 0.55 mmol) was dissolved in mixture (3:1, 1.5 mL) of acetic acid and 6 M aqueous solution of HCl, and heated with stirring in aluminum thermoblock at 95 °C overnight. Then, reaction mixture was diluted with water and ethyl acetate, aqueous layer was extracted three times with ethyl acetate, combined organic layers were washed with water, saturated aqueous solution of $NaHCO_3$, dried with Na_2SO_4, evaporated and purified using column chromatography (eluent: hexanes/ethyl acetate 100:0 to 80:20) to afford **7a** (145 mg, 0.49 mmol, 90%, 90% ee) as a colourless oil.

One-pot addition-decarboxylation procedure: triflone **1a** (118 mg, 0.500 mmol, c = 1M), ethyl acetoacetate (98 mg, 0.753 mmol, 1.5 equiv) and catalyst **2d** (11 mg, 0.025 mmol, 5 mol%) were dissolved in toluene (0.27 ml) and left for 20 h at +5 °C. After that time, one drop of acetic acid was added and toluene was evaporated. Residue was dissolved in acetic acid/6M aqueous HCl mixture (3:1, 1 ml) and heated with stirring in an aluminum thermoblock at 95 °C overnight, then worked up and purified as above, providing **7a** (123 mg, 0.418 mmol, 84% after two steps, 90% ee) as a colourless oil.

^{1}H NMR (400 MHz, $CDCl_3$): δ 7.38-7.24 (m, 5H), 3.95 (p, *J* = 6.9 Hz, 1H), 3.75 (ddd, *J* = 14.0, 7.5, 0.8 Hz, 1H), 3.51 (dd, *J* = 14.0, 6.6 Hz, 1H), 3.09 (dd, *J* = 17.8, 6.3 Hz, 1H), 3.01 (dd, *J* = 17.8, 7.2 Hz, 1H), 2.08 (s, 3H).

^{19}F NMR (376 MHz, $CDCl_3$): δ -78.7 (s).

^{13}C NMR (100 MHz, $CDCl_3$): δ 205.2, 140.1, 129.1, 127.9, 127.3, 119.2 (q, *J* = 328 Hz), 53.8, 48.1, 34.2, 30.3.

HRMS (ESI) m/z: $[M+H]^+$ Calcd for $C_{12}H_{13}F_3O_3S$: 295.0610; Found 295.0610.

$[M+Na]^+$ Calcd for $C_{12}H_{13}F_3O_3S$: 317.0430; Found 317.0429.

$[\alpha]_D^{25}$ = +4.5 (c 1.05, $CHCl_3$, 90% ee).

Enantiomeric excess was determined by HPLC analysis using a Phenomenex Lux® Cellulose-1 column (eluent: hexane/i-PrOH 90:10, flow rate 1.0 mL/min, λ = 209 nm): ent-(R) t_r = 9.11 min and ent-(S) t_r = 11.31 min.

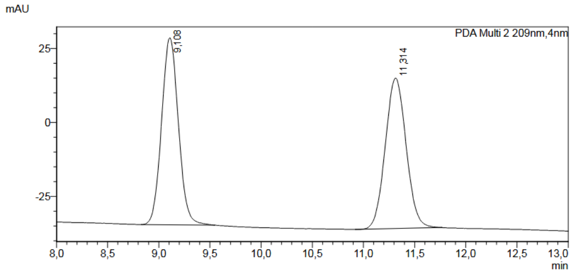

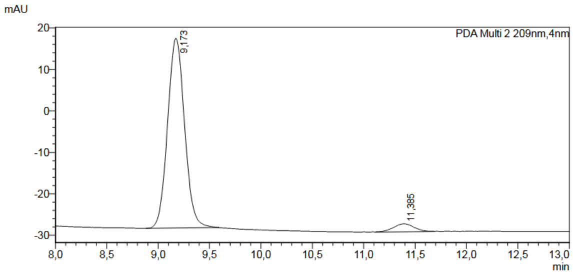

Peak#	Ret. Time	Area	Height	Mark	Area%
1	9,108	723039	63172	M	50,036
2	11,314	721991	50816	M	49,964
Total		1445030	113989		100,000

Peak#	Ret. Time	Area	Height	Mark	Area%
1	9,173	526174	45753	M	95,245
2	11,385	26271	1887		4,755
Total		552445	47640		100,000

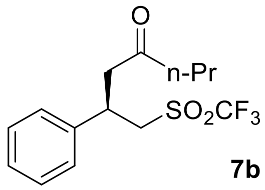

7b

Adduct **6b** (100 mg, 0.25 mmol) was dissolved in mixture (3:1, 3 mL) of acetic acid and 6 M aqueous solution of HCl, and heated with stirring in an aluminum thermoblock at 95 °C overnight. Reaction mixture was diluted with water and ethyl acetate, aqueous layer was extracted three times with ethyl acetate, combined organic layers were washed with water, saturated aqueous solution of $NaHCO_3$, dried with Na_2SO_4, evaporated and purified using column chromatography (silica gel, eluent: hexanes/ethyl acetate 100:0 to 95:5) to obtain ketone **7b** (75 mg, 0.23 mmol, 92%, 92% ee) as colourless oil.

^{1}H NMR (400 MHz, $CDCl_3$): δ 7.37-7.32 (m, 2H), 7.30-7.23 (m, 3H), 3.97 (p, J = 6.8 Hz, 1H), 3.76 (dd, J = 14.0, 7.6 Hz, 1H), 3.52 (dd, J = 14.0, 6.5 Hz, 1H), 3.02 (dd, J = 8.6, 6.7 Hz, 2H), 2.30 (q, J = 7.1 Hz, 2H), 1.52 (h, J = 7.4 Hz, 2H), 0.83 (t, J = 7.4 Hz, 3H).

^{19}F NMR (376 MHz, $CDCl_3$): δ -78.7 (s).

^{13}C NMR (100 MHz, $CDCl_3$): δ 207.6, 140.2, 129.0, 127.9, 127.3, 119.2 (q, J = 328 Hz), 53.8, 47.3, 45.1, 34.2, 16.9, 13.5.

HRMS (ESI) m/z: $[M+H]^+$ Calcd for $C_{14}H_{17}F_3O_3S$: 323.0923; Found 323.0920.

$[\alpha]_D^{25}$ = -1.5 (c 1.08, $CHCl_3$, 92% ee).

Enantiomeric excess was determined by HPLC analysis using a Phenomenex Lux® Cellulose-2 column (eluent: hexane/i-PrOH 98:2, flow rate 1.0 mL/min, λ = 209 nm): ent-(S) t_r = 4.46 min and ent-(R) t_r = 5.12 min.

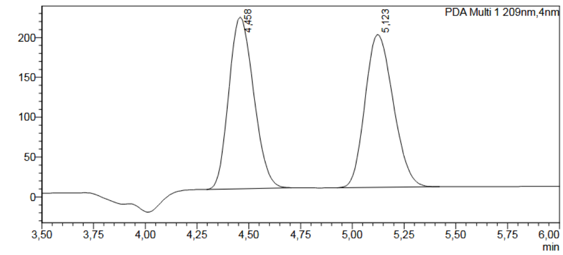

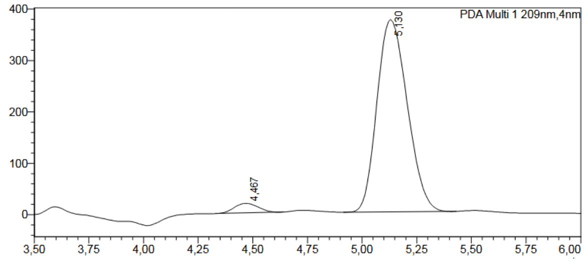

Peak#	Ret. Time	Area	Height	Mark	Area%
1	4,458	1800956	215419	M	49,763
2	5,123	1818096	191607	M	50,237
Total		3619052	407026		100,000

Peak#	Ret. Time	Area	Height	Mark	Area%
1	4,467	142173	18648	M	3,788
2	5,130	3611116	374425	M	96,212
Total		3753289	393073		100,000

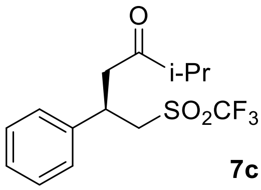

7c

Adduct **6c** (100 mg, 0.25 mmol) was dissolved in mixture (3:1, 3 mL) of acetic acid and 6 M aqueous solution of HCl, and heated with stirring in an aluminum thermoblock at 95 °C overnight. Reaction mixture was diluted with water and ethyl acetate, aqueous layer was extracted three times with ethyl acetate, combined organic layers were washed with water, saturated aqueous solution of $NaHCO_3$, dried with Na_2SO_4, evaporated and purified using column chromatography (silica gel, eluent: hexanes/ethyl acetate 100:0 to 95:5) to obtain ketone **7c** (77 mg, 0.24 mmol, 94%, 91% ee) as a colourless oil.

^{1}**H NMR** (400 MHz, $CDCl_3$): δ 7.34 (ddd, *J* = 7.9, 6.4, 1.6 Hz, 2H), 7.30-7.24 (m, 3H), 3.97 (p, *J* = 6.9 Hz, 1H), 3.78 (dd, *J* = 14.0, 7.6 Hz, 1H), 3.53 (dd, *J* = 14.0, 6.5 Hz, 1H), 3.07 (dd, *J* = 6.7, 2.8 Hz, 2H), 2.49 (hept, *J* = 6.9 Hz, 1H), 1.02 (d, *J* = 6.9 Hz, 3H), 0.98 (d, *J* = 6.9 Hz, 3H).

^{19}**F NMR** (376 MHz, $CDCl_3$): δ -78.7 (s).

^{13}**C NMR** (100 MHz, $CDCl_3$): δ 211.3, 140.3, 129.0, 127.8, 127.3, 119.2 (q, *J* = 327 Hz), 53.8, 45.2, 41.1, 34.3, 17.7, 17.7.

HRMS (ESI) m/z: $[M+H]^+$ Calcd for $C_{14}H_{17}F_3O_3S$: 323.0923; Found 323.0921.

$[\alpha]_D^{25}$ = -6.0 (c 1.02, $CHCl_3$, 91% ee).

Enantiomeric excess was determined by HPLC analysis using a Phenomenex Lux® Cellulose-1 column (eluent: hexane/i-PrOH 95:5, flow rate 1.0 mL/min, λ = 209 nm): ent-(R) t_r = 6.28 min and ent-(S) t_r = 8.45 min.

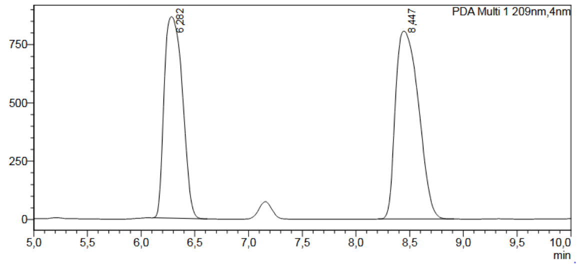

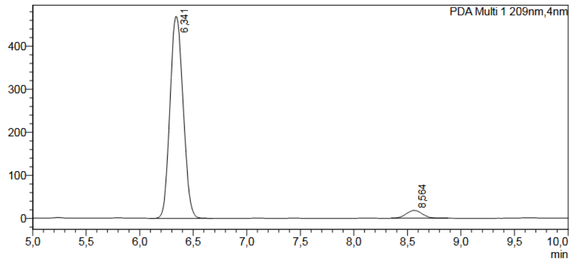

PDA Ch1 209nm

Peak#	Ret. Time	Area	Height	Mark	Area%
1	6,282	10239104	865270	M	45,879
2	8,447	12078636	806911	M	54,121
Total		22317740	1672182		100,000

PDA Ch1 209nm

Peak#	Ret. Time	Area	Height	Mark	Area%
1	6,341	4007939	468211	M	95,455
2	8,564	190848	18103	M	4,545
Total		4198787	486313		100,000

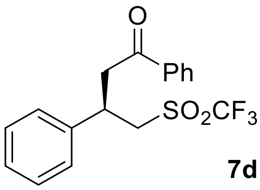

7d

Adduct **6d** (171 mg, 0.40 mmol) was dissolved in mixture (3:1, 1.5 mL) of acetic acid and 6 M aqueous solution of HCl, and heated with stirring in an aluminum thermoblock at 95 °C overnight. Reaction mixture was diluted with water and ethyl acetate, aqueous layer was extracted three times with ethyl acetate, combined organic layers were washed with water, saturated aqueous solution of $NaHCO_3$, dried with Na_2SO_4, evaporated and purified using column chromatography (eluent: hexanes/ethyl acetate 100:0 to 80:20) to obtain ketone **7d** (137 mg, 0.385 mmol, 96%, 90% ee) as a white solid.

^{1}H NMR (400 MHz, $CDCl_3$): δ 7.92-7.87 (m, 2H), 7.59-7.54 (m, 1H), 7.44 (dd, *J* = 8.4, 7.1 Hz, 2H), 7.38-7.24 (m, 5H), 4.17 (p, *J* = 6.9 Hz, 1H), 3.88 (dd, *J* = 14.1, 7.2 Hz, 1H), 3.66-3.52 (m, 1H), 3.59 (dd, *J* = 9.0, 6.8 Hz, 2H).

^{19}F NMR (376 MHz, $CDCl_3$): δ -78.7 (s).

^{13}C NMR (100 MHz, $CDCl_3$): δ 196.6, 140.3, 136.3, 133.6, 129.1, 128.7, 128.0, 127.9, 127.4, 119.3 (q, *J* = 328 Hz), 54.1, 43.7, 34.6.

HRMS (ESI) m/z: $[M+H]^+$ Calcd for $C_{17}H_{15}F_3O_3S$: 357.0767; Found 357.0765.

$[\alpha]_D^{25}$ = +9.7 (c 1.04, $CHCl_3$, 90% ee).

Enantiomeric excess was determined by HPLC analysis using a Phenomenex Lux® Cellulose-1 column (eluent: hexane/i-PrOH 90:10, flow rate 1.0 mL/min, λ = 209 nm): ent-(R) t_r = 7.17 min and ent-(S) t_r = 9.37 min.

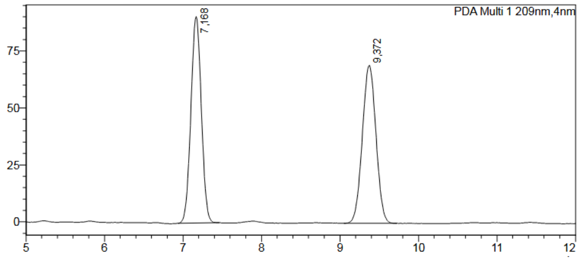

Peak#	Ret. Time	Area	Height	Mark	Area%
1	7,168	835657	90611	M	49,990
2	9,372	835976	69308	M	50,010
Total		1671633	159919		100,000

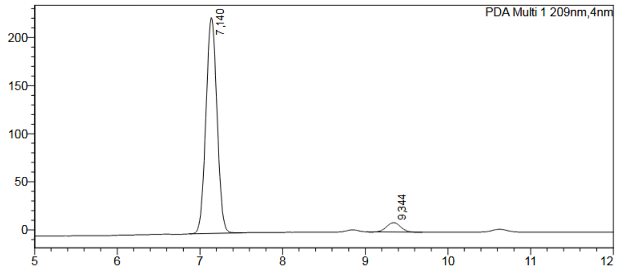

Peak#	Ret. Time	Area	Height	Mark	Area%
1	7,140	2086111	224191	M	94,752
2	9,344	115550	9780	M	5,248
Total		2201661	233971		100,000

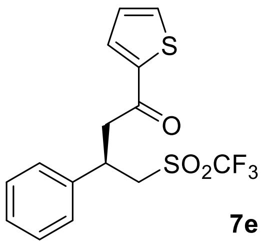

7e

Adduct **6e** (150 mg, 0.35 mmol) was dissolved in mixture (3:1, 3 mL) of acetic acid and 6 M aqueous solution of HCl, and heated with stirring in an aluminum thermoblock at 95 °C overnight. Reaction mixture was diluted with water and ethyl acetate, aqueous layer was extracted three times with ethyl acetate, organic layers were washed with water, saturated aqueous solution of $NaHCO_3$, dried with Na_2SO_4, evaporated and purified using column chromatography (silica gel, eluent: hexanes/ethyl acetate 100:0 to 90:10) to obtain ketone **7e** (120 mg, 0.33 mmol, 96%, 88% ee) as a yellowish oil.

^{1}H NMR (400 MHz, $CDCl_3$): δ 7.67 (dd, *J* = 3.9, 1.1 Hz, 1H), 7.63 (dd, *J* = 4.9, 1.1 Hz, 1H), 7.38-7.24 (m, 5H), 7.10 (dd, *J* = 4.9, 3.9 Hz, 1H), 4.14 (p, *J* = 6.9 Hz, 1H), 3.90 (dd, *J* = 14.1, 7.1 Hz, 1H), 3.61 (dd, *J* = 14.1, 7.0 Hz, 1H), 3.58-3.42 (m, 2H).

^{19}F NMR (376 MHz, $CDCl_3$): δ -78.7 (s).

^{13}C NMR (100 MHz, $CDCl_3$): δ 189.5, 143.4, 140.0, 134.3, 132.2, 129.1, 128.2, 128.0, 127.3, 119.2 (q, *J* = 328 Hz), 53.9, 44.2, 34.8.

HRMS (ESI) m/z: $[M+H]^+$ Calcd for $C_{15}H_{13}F_3O_3S_2$: 363.0331; Found 363.0328.

$[\alpha]_D^{25}$ = +7.2 (c 1.01, $CHCl_3$, 88% ee).

Enantiomeric excess was determined by HPLC analysis using a Phenomenex Lux® Cellulose-1 column (eluent: hexane/i-PrOH 90:10, flow rate 1.0 mL/min, λ = 260 nm): ent-(R) t_r = 8.42 min and ent-(S) t_r = 12.44 min.

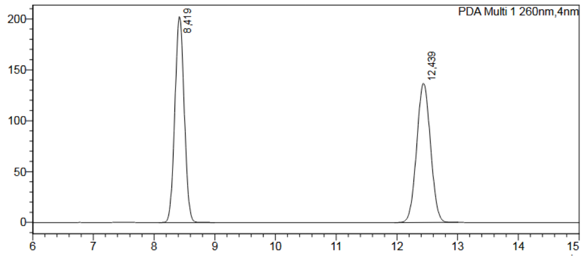

Peak#	Ret. Time	Area	Height	Mark	Area%
1	8,419	2167552	202287		50,003
2	12,439	2167309	136726		49,997
Total		4334861	339013		100,000

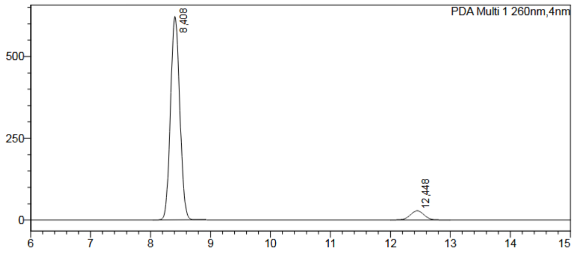

Peak#	Ret. Time	Area	Height	Mark	Area%
1	8,408	6696827	621919	M	93,863
2	12,448	437856	27746	M	6,137
Total		7134682	649665		100,000

V.2. Direct addition of acetone to triflone 1a

The experiment was carried out according to modified literature procedure for addition of acetone to nitrostyrene with catalyst **2k**: Günler, Z. I.; Alfonso, I.; Jimeno, C.; Pericàs, M. A. *Synthesis* **2017**, *49*, 319-325.

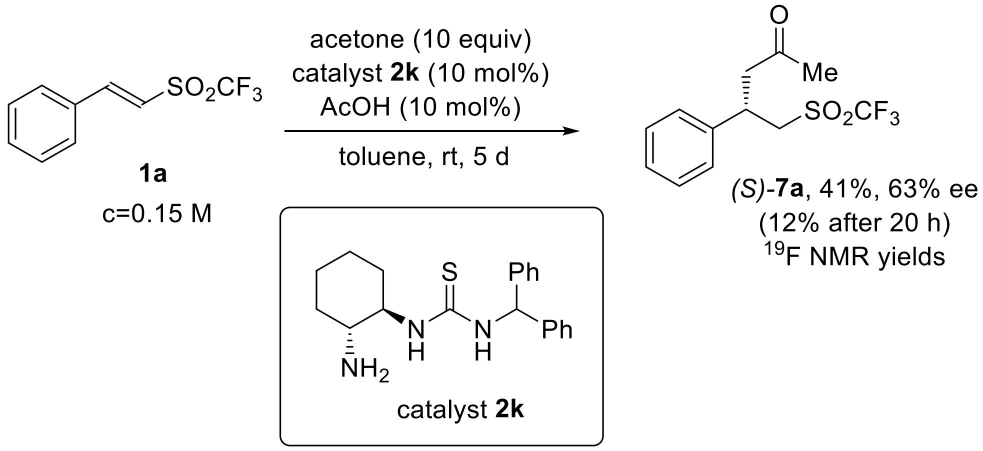

Triflone **1a** (12 mg, 0.05 mmol, c = 0.15 M), primary amine catalyst (1*R*,2*R*)-**2k** (1.7 mg, 0.005 mmol, 10 mol%), acetic acid (0.3 mg, 0.005 mmol, 10 mol%) and acetone (29 mg, 0.50 mmol, 10 equiv) were dissolved in toluene (0.29 ml) and homogenous reaction mixture was left at rt for 5 days. Then, reaction mixture was analyzed by ^{19}F NMR to determine yield, and filtered through pad of silicagel in order to remove catalyst, concentrated and residue was used to determine enantiomeric excess using HPLC analysis (41% yield (^{19}F NMR), 63% ee).

Analogous reaction conducted for 5 days at c[**1a**] = 0.5 M afforded **7a** in 73% yield (^{19}F NMR), and 37% ee.

In the reaction of **1a** with acetone and *(1R,2R)*-**2k**, the *(S)*-**7a** is formed as the major enantiomer.

In contrast, the reaction using ethyl acetoacetate and *(1R,2R)*-**2d**, with subsequent hydrolysis and decarboxylation, leads to *(R)*-**7a** (90% ee, Table 1 in the manuscript).

V.3. Cyclization of adduct 3a

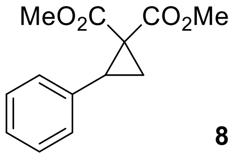

8

Adduct **3a** (154 mg, 0.42 mmol, 93% ee) was dissolved in THF (2 mL) and DBU was added (126 mg, 0.83 mmol, 2 equiv). Reaction vial was flushed with argon, closed and heated in an aluminum thermoblock at 60 °C overnight. Then, reaction mixture was cooled down to rt, quenched with water and aqueous solution of NH_4Cl, extracted three times with ethyl acetate, combined organic layers were washed with brine, dried with Na_2SO_4, evaporated and purified using preparative TLC (eluent: hexanes/ethyl acetate 99:1) to obtain cyclopropane **8** (73 mg, 0.31 mmol, 75%, <5% ee) as a colourless oil.

^{1}H NMR (400 MHz, $CDCl_3$): δ 7.32-7.15 (m, 5H), 3.79 (s, 3H), 3.36 (s, 3H), 3.23 (t, *J* = 8.6 Hz, 1H), 2.20 (dd, *J* = 8.0, 5.2 Hz, 1H), 1.75 (dd, *J* = 9.3, 5.2 Hz, 1H).

^{13}C NMR (100 MHz, $CDCl_3$): δ 170.2, 167.0, 134.5, 128.4, 128.1, 127.3, 52.8, 52.2, 37.2, 32.5, 19.1.

^{1}H and ^{13}C NMR data was consistent with the literature: Dalkilic, O.; Turbedaroglu, O.; Lafzi, F.; Kilic, H.; *J. Org. Chem.* **2023**, 88 (16), 11834–11846.

Enantiomeric excess was determined by HPLC analysis using a Phenomenex Lux® Cellulose-1 column (eluent: hexane/i-PrOH 99:1, flow rate 1.0 mL/min, λ = 209 nm): ent-(R) t_r = 5.57 min and ent-(S) t_r = 5.94 min.

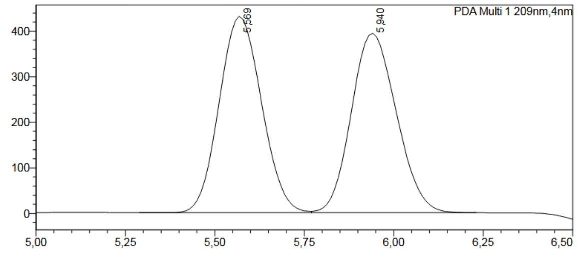

Peak#	Ret. Time	Area	Height	Mark	Area%
1	5,569	3550783	430788	M	50,729
2	5,940	3448687	393789	V M	49,271
Total		6999470	824577		100,000

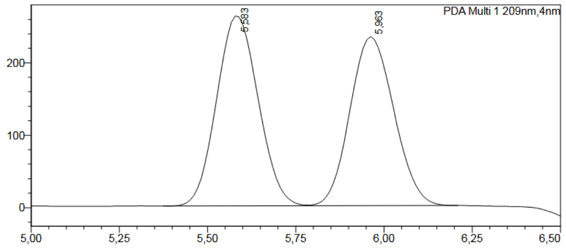

Peak#	Ret. Time	Area	Height	Mark	Area%
1	5,583	2153376	262134	M	51,571
2	5,963	2022189	233513	V M	48,429
Total		4175565	495647		100,000

V.4. Oxidative cyclization of adduct 3a

Prepared according to modified literature procedure: Miao, C.-B.; Zhang, M.; Tian, Z.-Y.; Xi, H.-T.; Sun, X.-Q.; Yang, H.-T.; *J. Org. Chem.* **2011**, 76 (23), 9809–9816.

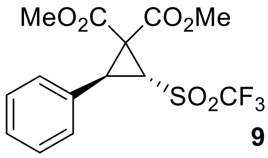

9

Adduct **3a** (184 mg, 0.5 mmol, 93% ee) and iodine (141 mg, 0.55 mmol, 1.1 equiv) were dissolved in toluene (7 mL) and DBU (380 mg, 2.5 mmol, 5 equiv, as solution in toluene, 1 mL) was added dropwise at rt with stirring. After 1 h reaction mixture was washed with aqueous solution of $Na_2S_2O_3$, aqueous layer was separated, extracted three times with ethyl acetate, combined organic layers were washed with brine, dried with Na_2SO_4 and evaporated. Residue was purified using column chromatography (eluent: hexanes/ethyl acetate 100:0 to 80:20) to obtain cyclopropane **9** (145 mg, 0.39 mmol, 79%, 92% ee, single diastereoisomer) as a colourless oil.

^{1}H NMR (400 MHz, $CDCl_3$): δ 7.38-7.32 (m, 3H), 7.27-7.22 (m, 2H), 4.00 (d, *J* = 7.5 Hz, 1H), 3.97 (dd, *J* = 7.6, 0.8 Hz, 1H), 3.89 (s, 3H), 3.57 (s, 3H).

^{19}F NMR (376 MHz, $CDCl_3$): δ -76.9 (s).

^{13}C NMR (100 MHz, $CDCl_3$): δ 163.6, 163.5, 129.8, 128.9, 128.8, 128.1, 119.4 (q, *J* = 327 Hz), 53.9, 53.7, 43.3, 41.3 (q, *J* = 1.6 Hz), 34.1.

HRMS (ESI) m/z: $[M+Na]^+$ Calcd for $C_{14}H_{13}F_3O_6S$: 389.0277; Found 389.0279.

$[\alpha]_D^{25}$ = -17.5 (c 1.02, $CHCl_3$, 92% ee).

Enantiomeric excess was determined by HPLC analysis using a Phenomenex Lux® Cellulose-3 column (eluent: hexane/i-PrOH 99:1, flow rate 1.0 mL/min, λ = 209 nm): ent-(2S,3R) t_r = 5.33 min and ent-(2R,3S) t_r = 6.24 min.

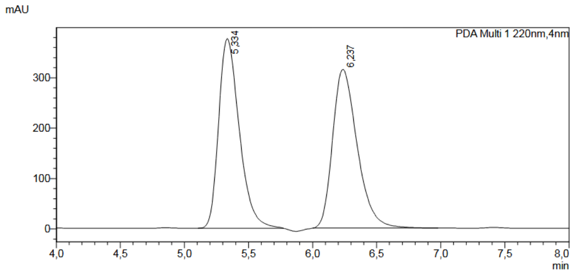

Peak#	Ret. Time	Area	Height	Mark	Area%
1	5,334	4217731	376421	M	50,030
2	6,237	4212591	315020	M	49,970
Total		8430322	691441		100,000

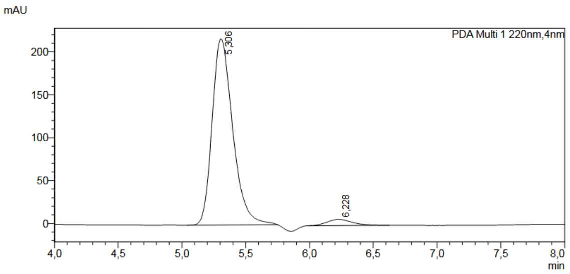

Peak#	Ret. Time	Area	Height	Mark	Area%
1	5,306	2440913	216624	M	96,137
2	6,228	98093	7139	M	3,863
Total		2539005	223763		100,000

Alternative attempt at synthesis of ethyl ester analogue of **9** from triflone **1a** and diethyl bromomalonate was based on modified literature procedure: S. Aitken, L.; E. Hammond, L.; Sundaram, R.; Shankland, K.; D. Brown, G.; A. Cobb, A. J. *Chem. Comm.* **2015**, 51 (70), 13558–13561. Cyclopropane derivative was obtained in presence of **2d** and potassium carbonate with high conversion but without enantioselectivity.

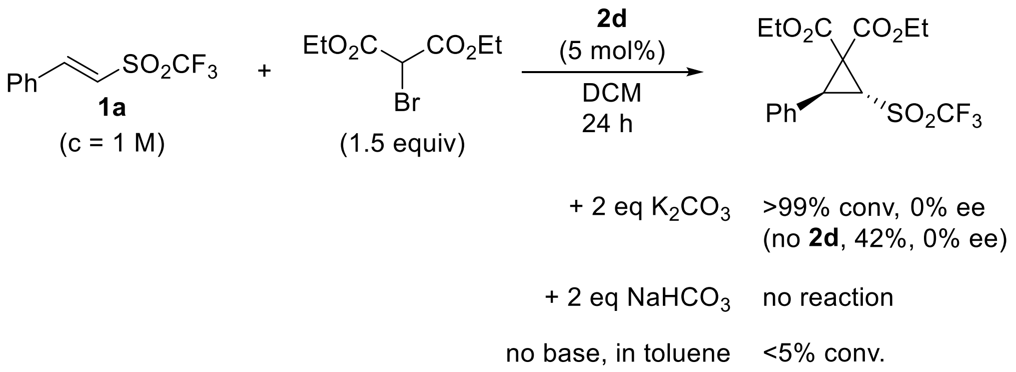

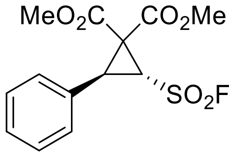

Reaction analogous to synthesis of **9** was conducted with **4b** as starting material. Adduct **4b** (159 mg, 0.5 mmol, 89% ee) and iodine (141 mg, 0.55 mmol, 1.1 equiv) were dissolved in toluene (7 ml) and DBU (380 mg, 2.5 mmol, 5 equiv, as solution in 1 ml of toluene) was added dropwise at rt with stirring. After 1 h reaction mixture was washed with aqueous $Na_2S_2O_3$ solution, aqueous layer was separated, extracted three times with ethyl acetate, combined organic layers were washed with brine, dried with Na_2SO_4 and evaporated. Residue was purified using column chromatography (hexane:ethyl acetate 100:0 to 80:20) to obtain cyclopropane (93 mg, 0.29 mol, 59%, 88% ee, single diastereoisomer) as a colourless oil.

^{1}H NMR (400 MHz, Chloroform-d) δ 7.37 – 7.32 (m, 3H), 7.28 – 7.24 (m, 2H), 4.16 (dd, J = 7.4, 3.8 Hz, 1H), 3.96 (dd, J = 7.4, 0.9 Hz, 1H), 3.89 (s, 3H), 3.57 (s, 3H).

^{19}F NMR (376 MHz, Chloroform-d) δ 62.0 (d, J = 3.7 Hz).

^{13}C NMR (100 MHz, Chloroform-d) δ 163.7, 163.6, 129.9, 128.8, 128.8, 128.1, 54.1, 53.6, 43.7, 42.9 (d, J = 31.4 Hz), 34.9.

HRMS (ESI) m/z: $[M+H]^+$ Calcd for $C_{13}H_{12}FO_6S$ 317.0490; Found 317.0491.

$[\alpha]_D^{25}$ = -36.4° (c 0.98, $CHCl_3$, 88% ee)

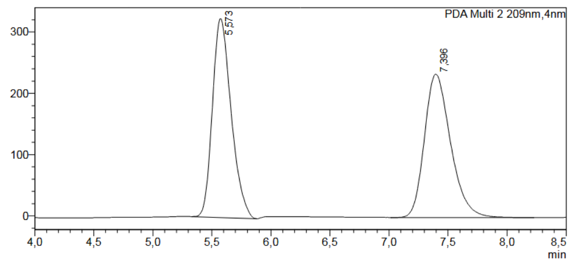

Peak#	Ret. Time	Area	Height	Mark	Area%
1	5,573	3578722	324044		49,792
2	7,396	3608606	234107		50,208
Total		7187328	558151		100,000

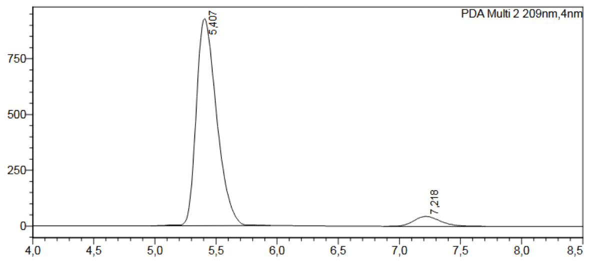

Peak#	Ret. Time	Area	Height	Mark	Area%
1	5,407	10756925	927681	M	94,136
2	7,218	670070	44241	M	5,864
Total		11426994	971921		100,000

V.5. Hydrolysis-decarboxylation of adduct 3a

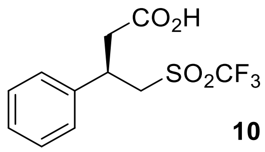

10

Adduct **3a** (97 mg, 0.26 mmol, 93% ee) was dissolved in mixture (3:1, 3 mL) of acetic acid and 6 M aqueous solution of HCl, and heated in an aluminum thermoblock at 95 °C overnight. Then, reaction mixture was cooled to rt, evaporated and purified using column chromatography (eluent: hexanes/ethyl acetate 100:0 + 0.1% acetic acid to 80:20 + 0.1% acetic acid) to obtain carboxylic acid **10** (70 mg, 0.24 mmol, 89%; 92% ee after esterification to **11**) as a white solid.

Compound **10** is too polar to analyze using available chiral HPLC columns, therefore enantiomeric excess was determined after esterification to compound **11** (see page S-67).

^{1}H NMR (400 MHz, $CDCl_3$): δ 9.62 (s, 1H), 7.38-7.28 (m, 3H), 7.27-7.22 (m, 2H), 3.90 (ddd, *J* = 14.0, 7.9, 6.2 Hz, 1H), 3.71 (dd, *J* = 14.1, 7.8 Hz, 1H), 3.53 (dd, *J* = 14.1, 6.2 Hz, 1H), 3.04 (dd, *J* = 16.8, 6.1 Hz, 1H), 2.88 (dd, *J* = 16.8, 8.1 Hz, 1H).

^{19}F NMR (376 MHz, $CDCl_3$): δ -78.5 (s).

^{13}C NMR (100 MHz, $CDCl_3$): δ 176.4, 139.3, 129.3, 128.3, 127.2, 119.2 (q, *J* = 327 Hz), 54.0, 39.5, 35.0.

HRMS (ESI) m/z: $[M-H]^-$ Calcd for $C_{11}H_{11}F_3O_4S$: 295.0246; Found 295.0253.

$[\alpha]_D^{25}$ = +10.2 (c 1.00, $CHCl_3$, 91% ee).

V.6. Esterification of acid 10

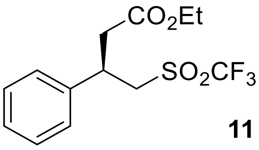

11

Carboxylic acid **10** (70 mg, 0.24 mmol) was dissolved in absolute ethanol (3 mL) and *p*-TSA (5 mg, ca. 3 mol%) was added. Reaction mixture was heated in an aluminum thermoblock in a sealed tube at 85 °C overnight, then evaporated and purified directly on chromatographic column (eluent: hexanes/ethyl acetate 100:0 to 90:10) to obtain ester **11** (70 mg, 0.22 mmol, 91%, 92% ee) as a colourless oil.

^{1}H NMR (400 MHz, $CDCl_3$): δ 7.40-7.23 (m, 5H), 4.07 (q, *J* = 7.1 Hz, 2H), 3.93 (p, *J* = 7.0 Hz, 1H), 3.78 (dd, *J* = 14.1, 7.4 Hz, 1H), 3.55 (dd, *J* = 14.1, 6.5 Hz, 1H), 2.97 (dd, *J* = 16.1, 6.5 Hz, 1H), 2.83 (dd, *J* = 16.2, 7.8 Hz, 1H), 1.16 (t, *J* = 7.1 Hz, 3H).

^{19}F NMR (376 MHz, $CDCl_3$): δ -78.6 (s).

^{13}C NMR (100 MHz, $CDCl_3$): δ 170.3, 139.7, 129.1, 128.1, 127.2, 119.2 (q, *J* = 327 Hz), 60.9, 54.0, 40.0, 35.3, 14.0.

HRMS (ESI) m/z: $[M+H]^+$ Calcd for $C_{13}H_{15}F_3O_4S$: 325.0716, Found 325.0715.

$[M+Na]^+$ Calcd for $C_{13}H_{15}F_3O_4S$: 347.0535; Found 347.0535.

$[\alpha]_D^{25}$ = +9.0 (c 0.92, $CHCl_3$, 92% ee).

Enantiomeric excess was determined by HPLC analysis using a Phenomenex Lux® Cellulose-1 column (eluent: hexane/i-PrOH 95:5, flow rate 1.0 mL/min, λ = 220 nm): ent-(R) t_r = 5.30 min and ent-(S) t_r = 6.34 min.

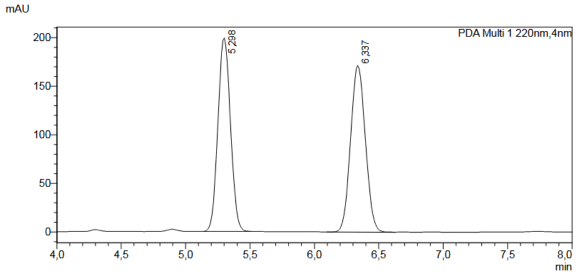

Peak#	Ret. Time	Area	Height	Mark	Area%
1	5,298	1348386	199223	M	49,582
2	6,337	1371145	171594	M	50,418
Total		2719531	370817		100,000

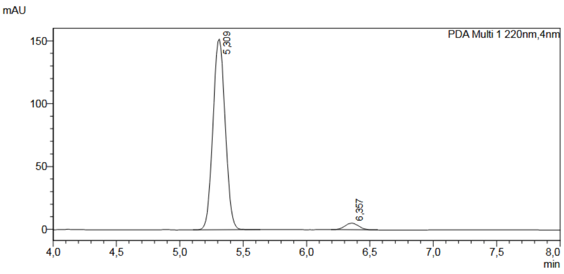

Peak#	Ret. Time	Area	Height	Mark	Area%
1	5,309	1022288	151719	M	96,200
2	6,357	40385	5290		3,800
Total		1062673	157009		100,000

V.7. Synthesis of oxazoles 12 and 13

Oxazoles **12** and **13** were prepared according to modified literature procedure: Liu, B.; Han, X.; Dong, Z.; Lv, H.; Zhou, H.-B.; Dong, C. *Tetrahedron Asymm.* **2013**, *24*, 1276-1280.

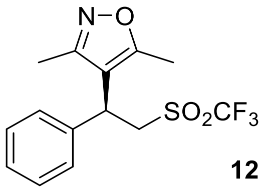

12

Adduct **5c** (110 mg, 0.33 mmol, 91% ee) and hydroxylamine hydrochloride (34 mg, 0.49 mmol, 1.5 equiv) were dissolved in ethanol (1.5 mL, 96%) and heated with stirring in an aluminum thermoblock at 60 °C overnight. After cooling down to rt, reaction mixture was divided between saturated aqueous solution of $NaHCO_3$ and ethyl acetate, aqueous layer was extracted three times with ethyl acetate, combined organic layers were dried with Na_2SO_4, evaporated and purified using column chromatography (silica, hexanes/ethyl acetate 100:0 to 85:15) to obtain isoxazole **12** (94 mg, 0.28 mmol, 86%, 91% ee) as a colourless oil.

^{1}H NMR (400 MHz, $CDCl_3$): δ 7.37 (dd, *J* = 8.2, 6.5 Hz, 2H), 7.33-7.26 (m, 1H), 7.20-7.16 (m, 2H), 4.70 (dd, *J* = 9.8, 5.4 Hz, 1H), 3.98-3.85 (m, 2H), 2.43 (s, 3H), 2.15 (s, 3H).

^{19}F NMR (376 MHz, $CDCl_3$): δ -78.7 (s).

^{13}C NMR (100 MHz, $CDCl_3$): δ 166.3, 158.5, 138.0, 129.2, 127.9, 126.7, 119.2 (q, *J* = 327 Hz), 112.5, 52.4, 32.4, 11.7, 10.9.

HRMS (ESI) m/z: $[M+H]^+$ Calcd for $C_{14}H_{14}F_3NO_3S$: 334.0719; Found 334.0717.

$[\alpha]_D^{25}$ = -17.1 (c 1.01, $CHCl_3$, 91% ee).

Enantiomeric excess was determined by HPLC analysis using a Phenomenex Lux® Cellulose-2 column (eluent: hexane/i-PrOH 90:10, flow rate 1.0 mL/min, λ = 209 nm): ent-(R) t_r = 4.70 min and ent-(S) t_r = 5.70 min.

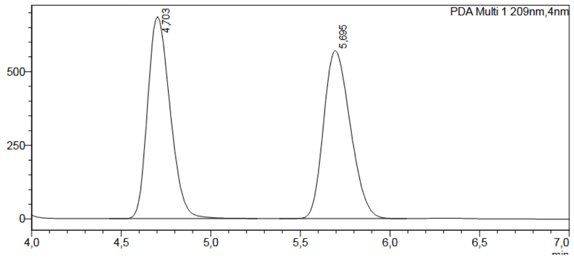

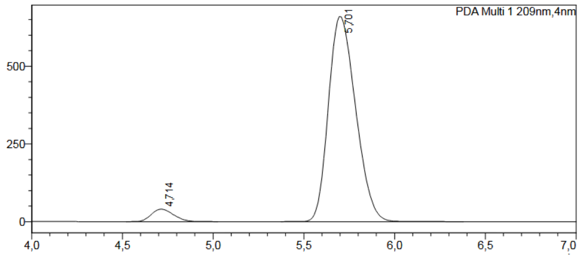

Peak#	Ret. Time	Area	Height	Mark	Area%
1	4,703	5976436	685064	M	50,200
2	5,695	5928898	570124	M	49,800
Total		11905335	1255188		100,000

Peak#	Ret. Time	Area	Height	Mark	Area%
1	4,714	343354	40499	M	4,743
2	5,701	6895590	659533	M	95,257
Total		7238944	700031		100,000

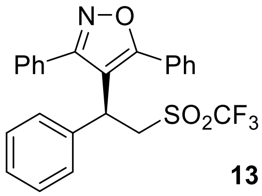

13

Adduct **5d** (106 mg, 0.23 mmol, undetermined ee) and hydroxylamine hydrochloride (24 mg, 0.35 mmol, 1.5 equiv) were dissolved in ethanol (1 mL, 96%) and heated with stirring in an aluminum thermoblock to 80 °C for 3 days. After cooling down to rt, reaction mixture was divided between saturated aqueous solution of $NaHCO_3$ and ethyl acetate, aqueous layer was extracted three times with ethyl acetate, combined organic layers were dried with Na_2SO_4, evaporated and purified using column chromatography (silica, eluent: hexanes/ethyl acetate 100:0 to 90:10) to obtain isoxazole **13** (87 mg, 0.19 mmol, 83%, 79% ee) as a colourless oil.

^{1}H NMR (400 MHz, $CDCl_3$): δ 7.61 (dd, J = 7.8, 1.8 Hz, 2H), 7.52-7.43 (m, 4H), 7.42-7.32 (m, 7H), 7.20-7.16 (m, 2H), 5.05 (dd, J = 10.2, 3.9 Hz, 1H), 3.76 (dd, J = 14.2, 10.1 Hz, 1H), 3.64 (dd, J = 14.2, 4.0 Hz, 1H).

^{19}F NMR (376 MHz, $CDCl_3$): δ -78.7 (s).

^{13}C NMR (100 MHz, $CDCl_3$): δ 168.0, 163.3, 139.2, 130.7, 129.9, 129.3, 129.1, 128.8, 128.7, 128.2, 128.0, 127.5, 127.0, 119.0 (q, J = 328 Hz), 112.0, 52.0, 32.3.

HRMS (ESI) m/z: $[M+H]^+$ Calcd for $C_{24}H_{18}F_3NO_3S$: 458.1032; Found 458.1030.

Enantiomeric excess was determined by HPLC analysis using a Phenomenex Lux® Cellulose-2 column (eluent: hexane/i-PrOH 95:5, flow rate 1.0 mL/min, λ = 209 nm): ent-(R) t_r = 6.37 min and ent-(S) t_r = 8.44 min.

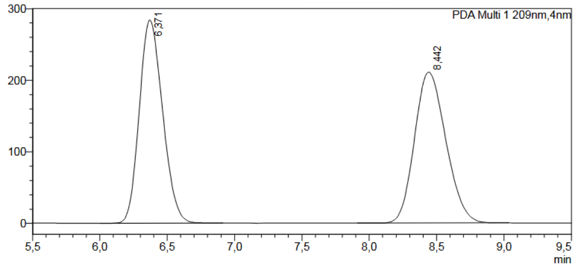

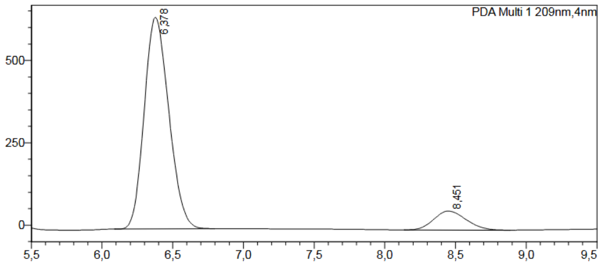

Peak#	Ret. Time	Area	Height	Mark	Area%
1	6,371	3419465	283819	M	50,038
2	8,442	3414242	210691	M	49,962
Total		6833707	494510		100,000

Peak#	Ret. Time	Area	Height	Mark	Area%
1	6,378	7797257	642583	M	89,504
2	8,451	914325	57409	M	10,496
Total		8711582	699992		100,000

V.8. DBU-catalyzed racemization of adduct 3a

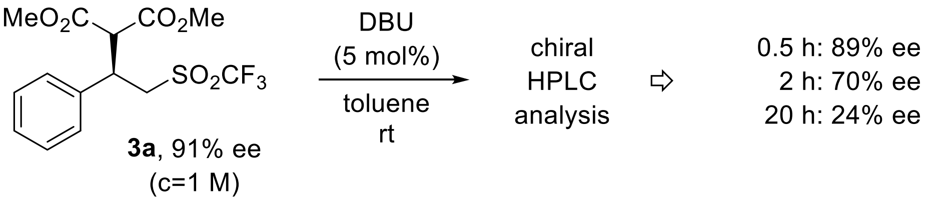

Adduct **3a** (37 mg, 0.10 mmol, 91% ee) was dissolved in toluene (0.056 ml, 0.1 ml total, c = 1 M) and DBU (0.7 mg, 5 mol%, added as 10% solution in toluene, 7 µl) was added. After 30 min, 2 h, and 20 h, 20 µl samples were taken, filtered through a plug of silica gel in Pasteur pipette, eluted with DCM in order to stop the reaction and concentrated. Enantiomeric excess was determined using HPLC analysis. Triflone **1a** was not observed in ^{19}F NMR during the reaction.

In a similar experiment involving nitrostyrene adduct **4d** no decrease in enantiomeric excess was observed.

V.9. Reversible elimination-addition of malonate in compound 3a in presence of DBU

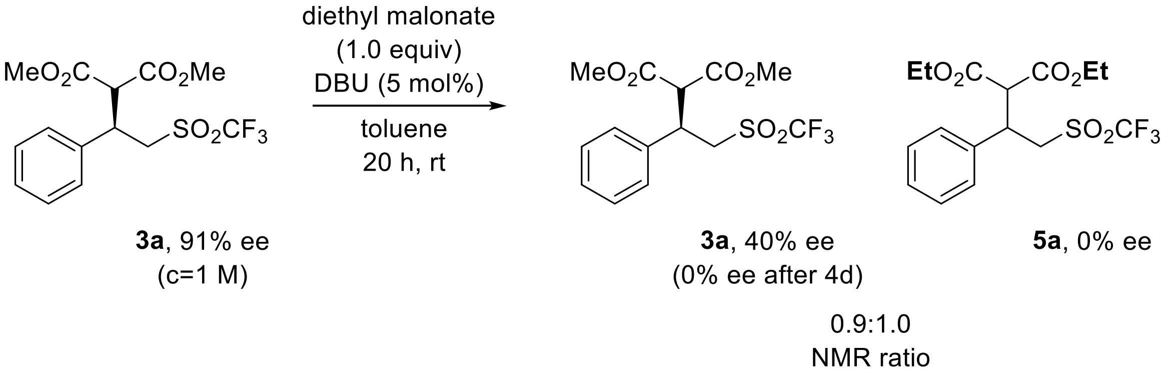

Adduct **3a** (37 mg, 0.10 mmol, 91% ee) and diethyl malonate (16 mg, 0.10 mmol, 1 equiv) were dissolved in toluene (0.04 ml, c = 1 M) and DBU (0.7 mg, 5 mol%, 10% solution in toluene, 7 µl) was added. After 20 h and 4 days, 20 µl samples were taken using microliter syringe, filtered through silica gel plug in Pasteur pipette, washed with DCM in order to stop the reaction and concentrated. The **3a** : **5a** ratio was measured with ^{1}H NMR. Enantiomeric excesses was determined using HPLC analysis. After 20 h, a mixture of compounds **3a** and **5a** in a ratio of 0.9:1 was obtained. The enantiomeric excess for **3a** decreased from 91% to 40% ee after 20 h, while compound **5a** was formed as a racemate.

V.10. Competitive experiments of malonate addition to 1a, 1s and 1r

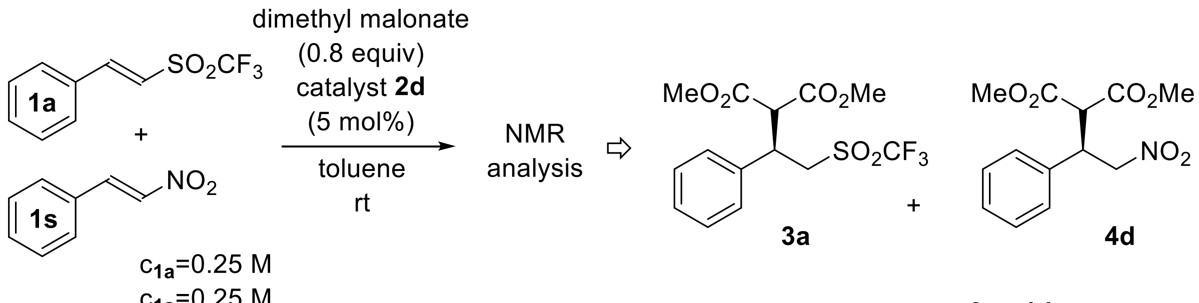

dimethyl malonate :	3a :	4d
0.25 h: 72 :	2 :	36
0.5 h: 24 :	4 :	72
1 h: 0 :	5 :	95

Triflone **1a** (11.8 mg, 0.05 mmol), nitrostyrene **1s** (7.5 mg, 0.05 mmol) and catalyst **2d** (1.1 mg, 2.5 µmol, 5 mol%) were dissolved in toluene (0.225 ml, 0.25 ml total volume) and dimethyl malonate (5.3 mg, 0.04 mmol, 0.8 equiv) was added. After 15 min, 30 min and 1 h, 50 µl samples were taken, filtered through silica gel plug in Pasteur pipette, eluted with DCM in order to stop the reaction and concentrated. Ratios of **3a**, **4d** and dimethyl malonate were determined using ^{1}H NMR.

Similar experiment, using 10 mol% of catalyst **2d** was carried out at rt to compare reactivity of triflone **1a** and nonaflone **1r**.

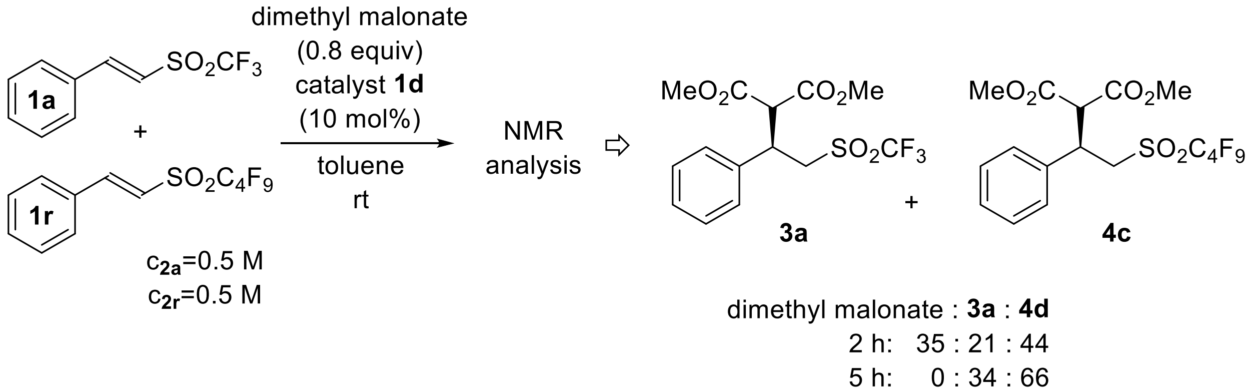

dimethyl malonate :	3a :	4d
2 h: 35 :	21 :	44
5 h: 0 :	34 :	66

Triflone **1a** (24 mg, 0.1 mmol), nonaflone **1r** (39 mg, 0.1 mmol) and catalyst **2d** (4.4 mg, 0.01 mmol, 10 mol%) were dissolved in toluene (0.122 ml, 0.2 ml total volume) and dimethyl malonate (11 mg, 0.08 mmol, 0.8 equiv) was added. After 2 h and 5 h, 20 µl samples were taken, filtered through silica gel plug in Pasteur pipette, eluted with DCM in order to stop the reaction and concentrated. Ratios of **3a**, **4d** and dimethyl malonate were determined using ^{1}H NMR.

VI. Single-crystal XRD analysis data for compound 3b (CCDC 2373150)

Experimental. Single colourless needle-shaped crystals of **3b** were obtained by recrystallisation from hexane/isopropanol 9:1. A suitable crystal 0.55×0.07×0.05 mm^3 was selected and mounted on a suitable support on an SuperNova, Dual, Cu at home/near, HyPix diffractometer. The crystal was kept at a steady *T*=100.00(10) K during data collection. The structure was solved with the ShelXT 2018/2 (Sheldrick, 2018) structure solution program using the Intrinsic Phasing solution method and by using **Olex2** (O.V. Dolomanov, L.J. Bourhis, R.J. Gildea, J.A.K. Howard, H. Puschmann; *J. Appl. Cryst.*, (2009), **42**, 339-341.) as the graphical interface. The model was refined with version 2018/3 of ShelXL 2018/3 (Sheldrick, 2015) using Least Squares minimisation.

The figure below was prepared using CCDC Mercury 3.8 (Macrae, C. F.; Edgington, P. R.; McCabe, P.; Pidcock, E.; Shields, G. P.; Taylor, R.; Towler, M.; Van De Streek, J. Mercury: Visualization and Analysis of Crystal Structures. *J. Appl. Crystallogr.* **2006**, *39*, 453-457). Ellipsoid contour probability level is 50%.

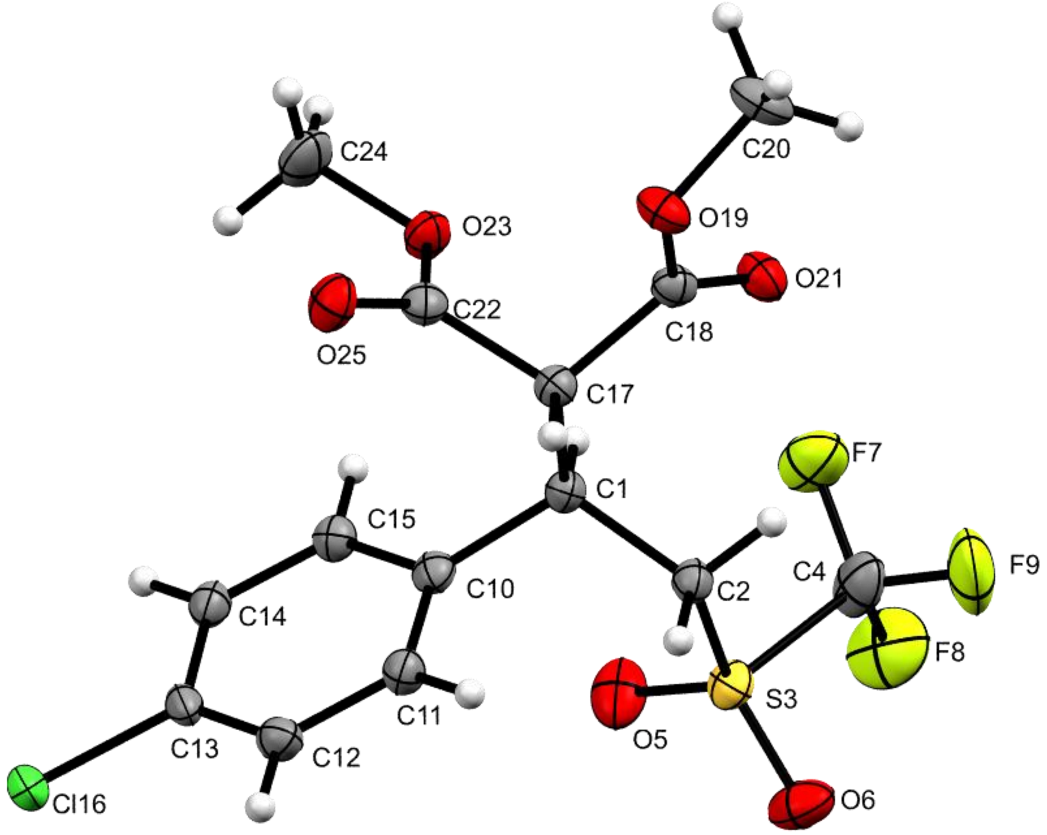

Crystal Data. $C_{14}H_{14}O_6F_3SCl$, M_r = 402.76, orthorhombic, $P2_12_12_1$ (No. 19), a = 5.82530(10) Å, b = 15.8084(2) Å, c = 18.8285(2) Å, *a* = *b* = *g* = 90°, *V* = 1733.89(4) Å^3, *T* = 100.00(10) K, *Z* = 4, *Z'* = 1, *m*(Cu K$_a$) = 3.644, 31014 reflections measured, 3082 unique (R_{int} = 0.0639) which were used in all calculations. The final wR_2 was 0.0726 (all data) and R_1 was 0.0267 (I > 2(I)).

**Compound**	**3b**
Formula	$C_{14}H_{14}O_6F_3SCl$
$D_{calc.}$/ g cm^{-3}	1.543
m/mm^{-1}	3.644
Formula Weight	402.76
Colour	colourless
Shape	needle
Size/mm^3	0.55×0.07×0.05
T/K	100.00(10)
Crystal System	orthorhombic
Flack Parameter	0.000(8)
Hooft Parameter	0.001(5)
Space Group	$P2_12_12_1$
a/Å	5.82530(10)
b/Å	15.8084(2)
c/Å	18.8285(2)
a/°	90
b/°	90
g/°	90
V/$Å^3$	1733.89(4)
Z	4
Z'	1
Wavelength/Å	1.54184
Radiation type	Cu K_a
Q_{min}/°	3.651
Q_{max}/°	67.007
Measured Refl.	31014
Independent Refl.	3082
Reflections with I > 2(I)	3018
R_{int}	0.0639
Parameters	228
Restraints	0
Largest Peak	0.223
Deepest Hole	-0.264
GooF	1.066
wR_2 (all data)	0.0726
wR_2	0.0722
R_1 (all data)	0.0272
R_1	0.0267

Experimental Extended. A colourless needle-shaped crystal with dimensions 0.55×0.07×0.05 mm^3 was mounted on a suitable support. Data were collected using an SuperNova, Dual, Cu at home/near, HyPix diffractometer operating at *T* = 100.00(10) K.

Data were measured using *w* scans of 0.5° per frame for 0.1/0.2/1.0 s using Cu K_a radiation. The diffraction pattern was indexed and the total number of runs and images was based on the strategy calculation from the program CrysAlisPro (Rigaku, V1.171.42.70a, 2022) The maximum resolution that was achieved was *Q* = 67.007° (0.84 Å).

The diffraction pattern was indexed The diffraction pattern was indexed and the total number of runs and images was based on the strategy calculation from the program CrysAlisPro (Rigaku, V1.171.42.70a, 2022)

and the unit cell was refined using CrysAlisPro (Rigaku, V1.171.42.70a, 2022) on 20951 reflections, 68% of the observed reflections.

Data reduction, scaling and absorption corrections were performed using CrysAlisPro (Rigaku, V1.171.42.70a, 2022). The final completeness is 100.00 % out to 67.007° in *Q*. A multi-scan absorption correction was performed using CrysAlisPro 1.171.42.70a (Rigaku Oxford Diffraction, 2022) using spherical harmonics,implemented in SCALE3 ABSPACK scaling algorithm. The absorption coefficient *m* of this material is 3.644 mm^{-1} at this wavelength (*l* = 1.542Å) and the minimum and maximum transmissions are 0.545 and 1.000.

The structure was solved and the space group $P2_12_12_1$ (# 19) determined by the ShelXT 2018/2 (Sheldrick, 2018) structure solution program using Intrinsic Phasing and refined by Least Squares using version 2018/3 of ShelXL 2018/3 (Sheldrick, 2015). All non-hydrogen atoms were refined anisotropically. Hydrogen atom positions were calculated geometrically and refined using the riding model. Hydrogen atom positions were calculated geometrically and refined using the riding model.

_exptl_absorpt_process_details: CrysAlisPro 1.171.42.70a (Rigaku Oxford Diffraction, 2022) using spherical harmonics,implemented in SCALE3 ABSPACK scaling algorithm.

Table 1: Fractional Atomic Coordinates (×10^4) and Equivalent Isotropic Displacement Parameters (Å^2×10^3) for **3b**. U_{eq} is defined as 1/3 of the trace of the orthogonalised U_{ij}.

Atom	x	y	z	U_{eq}
C1	6091(4)	5464.1(14)	3557.3(12)	17.0(5)
C2	6536(4)	4929.6(15)	4228.4(13)	19.3(5)
C4	3268(6)	3610.5(19)	4379.4(16)	35.9(7)
C10	5340(4)	6363.6(16)	3723.5(13)	19.4(5)
C11	6473(4)	6865.7(15)	4220.3(13)	20.9(5)
C12	5817(5)	7701.5(16)	4333.4(13)	22.6(5)
C13	4025(5)	8032.3(14)	3938.0(13)	21.2(5)
C14	2890(5)	7550.8(15)	3429.0(13)	21.0(5)
C15	3574(4)	6720.0(15)	3325.0(13)	19.8(5)
C17	8307(4)	5511.8(15)	3103.4(12)	18.2(5)
C18	9097(4)	4653.6(15)	2821.4(12)	19.6(5)
C20	12123(5)	4017.5(18)	2153.8(15)	29.8(6)
C22	7898(4)	6091.3(16)	2463.8(13)	21.2(5)
C24	5566(6)	6275.3(19)	1451.1(15)	35.2(7)
Cl16	3148.9(12)	9072.9(4)	4069.4(3)	28.32(17)
F7	3080(4)	3603.8(12)	3681.7(10)	47.0(5)
F8	1284(5)	3379.3(14)	4666.0(12)	62.4(7)
F9	4876(5)	3059.1(12)	4557.4(13)	59.3(7)
O5	2089(4)	5186.2(14)	4496.4(12)	37.6(5)
O6	4519(4)	4553.1(14)	5442.2(10)	36.1(5)
O19	11139(3)	4754.0(11)	2505.4(10)	24.6(4)
O21	8061(3)	4002.0(11)	2872.6(10)	26.0(4)
O23	6240(3)	5766.2(11)	2057.6(9)	24.5(4)
O25	8885(4)	6742.3(12)	2352.0(10)	28.8(4)
S3	3984.7(12)	4673.8(4)	4706.4(3)	23.86(16)

Table 2: Anisotropic Displacement Parameters (×10[4]) **3b**. The anisotropic displacement factor exponent takes the form: $-2p^2[h^2a^{*2} \times U_{11} + \ldots +2hka^* \times b^* \times U_{12}]$

Atom	U_{11}	U_{22}	U_{33}	U_{23}	U_{13}	U_{12}
C1	16.3(10)	17.2(11)	17.6(11)	0.7(9)	-0.6(9)	-0.3(9)
C2	20.1(12)	17.2(11)	20.8(11)	2.4(9)	2.2(9)	0.7(9)
C4	46.4(19)	26.2(13)	35.1(15)	6.5(12)	-6.7(14)	-14.2(13)
C10	19.5(12)	19.2(12)	19.4(11)	2.5(9)	4.0(9)	-0.7(9)
C11	22.2(13)	19.7(11)	21.0(11)	2.2(9)	-1.3(9)	0.3(10)
C12	27.6(13)	20.6(12)	19.6(11)	-1.7(9)	0.7(10)	-2.7(11)
C13	28.0(13)	14.3(10)	21.4(11)	1.6(9)	8.1(10)	3.3(10)
C14	20.8(12)	20.7(11)	21.5(12)	4.3(9)	1.4(10)	2.4(10)
C15	18.1(12)	19.9(11)	21.4(11)	0.9(9)	0.5(9)	-1.4(9)
C17	17.4(12)	18.0(11)	19.3(11)	-0.5(9)	1.1(9)	-0.8(9)
C18	19.8(11)	21.6(12)	17.6(11)	-1.1(9)	-1.5(9)	0.2(11)
C20	23.8(13)	31.9(14)	33.8(14)	-13.5(12)	3.8(11)	2.3(12)
C22	20.8(12)	22.1(12)	20.7(11)	0.0(10)	3.7(10)	1.3(10)
C24	46.7(19)	35.1(15)	23.9(13)	5.9(11)	-12.6(13)	-0.6(13)
Cl16	44.4(4)	17.8(3)	22.7(3)	-0.3(2)	4.2(3)	8.6(3)
F7	65.6(13)	42.6(10)	32.7(9)	-2.2(8)	-9.4(10)	-19.0(10)
F8	68.5(15)	54.6(12)	64.1(13)	3.5(11)	9.8(12)	-40.3(12)
F9	87.4(18)	21.9(9)	68.7(14)	4.3(9)	-23.8(13)	5.9(10)
O5	23.7(9)	37.6(11)	51.4(13)	13.7(10)	12.0(10)	3.0(9)
O6	41.9(12)	43.1(12)	23.2(9)	1.5(8)	5.1(8)	-13.1(10)
O19	21.1(9)	24.2(8)	28.4(9)	-7.6(7)	4.6(8)	-0.5(8)
O21	27.0(9)	19.9(9)	31.0(9)	-3.7(7)	3.3(8)	-1.0(8)
O23	30.1(10)	22.9(9)	20.5(8)	1.5(7)	-6.1(7)	-1.4(8)
O25	30.4(10)	24.9(9)	30.9(10)	5.8(7)	-1.2(9)	-6.2(8)
S3	24.9(3)	21.4(3)	25.2(3)	2.9(2)	4.1(2)	-4.4(2)

Table 3: Bond Lengths in Å for **3b**.

Atom	Atom	Length/Å
C1	C2	1.542(3)
C1	C10	1.520(3)
C1	C17	1.550(3)
C2	S3	1.784(2)
C4	F7	1.318(3)
C4	F8	1.327(4)
C4	F9	1.323(4)
C4	S3	1.838(3)
C10	C11	1.393(4)
C10	C15	1.392(4)
C11	C12	1.392(4)
C12	C13	1.385(4)
C13	C14	1.391(4)

Atom	Atom	Length/Å
C13	Cl16	1.740(2)
C14	C15	1.386(3)
C17	C18	1.528(3)
C17	C22	1.532(3)
C18	O19	1.339(3)
C18	O21	1.198(3)
C20	O19	1.457(3)
C22	O23	1.335(3)
C22	O25	1.197(3)
C24	O23	1.451(3)
O5	S3	1.425(2)
O6	S3	1.433(2)

Table 4: Bond Angles in ° for **3b**.

Atom	Atom	Atom	Angle/°
C2	C1	C17	109.8(2)
C10	C1	C2	113.09(19)
C10	C1	C17	107.92(18)
C1	C2	S3	113.44(17)
F7	C4	F8	109.3(3)
F7	C4	F9	107.8(3)
F7	C4	S3	111.1(2)
F8	C4	S3	108.3(2)
F9	C4	F8	109.4(3)
F9	C4	S3	110.9(2)
C11	C10	C1	122.3(2)
C15	C10	C1	118.7(2)
C15	C10	C11	118.8(2)
C12	C11	C10	120.9(2)
C13	C12	C11	118.9(2)
C12	C13	C14	121.5(2)
C12	C13	Cl16	120.1(2)
C14	C13	Cl16	118.4(2)
C15	C14	C13	118.6(2)
C14	C15	C10	121.3(2)
C18	C17	C1	113.54(19)
C18	C17	C22	107.74(19)
C22	C17	C1	109.4(2)
O19	C18	C17	108.5(2)
O21	C18	C17	125.7(2)
O21	C18	O19	125.8(2)
O23	C22	C17	109.4(2)
O25	C22	C17	125.3(2)
O25	C22	O23	125.3(2)
C18	O19	C20	117.2(2)
C22	O23	C24	115.7(2)
C2	S3	C4	103.15(14)
O5	S3	C2	112.12(12)
O5	S3	C4	104.53(15)
O5	S3	O6	120.80(14)
O6	S3	C2	109.71(12)
O6	S3	C4	104.56(13)

Table 5: Hydrogen Fractional Atomic Coordinates (×10^4) and Equivalent Isotropic Displacement Parameters (Å^2×10^3) for **3b**. U_{eq} is defined as 1/3 of the trace of the orthogonalised U_{ij}.

Atom	x	y	z	U_{eq}
H1	4862.62	5183.74	3270.49	20
H2A	7585.38	5244.49	4546.31	23
H2B	7314.08	4398.44	4088.51	23
H11	7710.91	6634.52	4485.36	25
H12	6586.37	8039.37	4676.2	27
H14	1670.89	7786.35	3158.34	25
H15	2823.33	6387.38	2974.87	24
H17	9563.03	5758.47	3400.36	22
H20A	11600.98	3999.08	1659.3	45
H20B	11628.47	3502.54	2399.94	45
H20C	13801.82	4055.4	2165.8	45
H24A	6901.26	6370.04	1144.58	53
H24B	4971.15	6820.61	1616.51	53
H24C	4370.44	5978.83	1182.5	53

VII. Reproductions of ^{1}H, ^{13}C and ^{19}F spectra

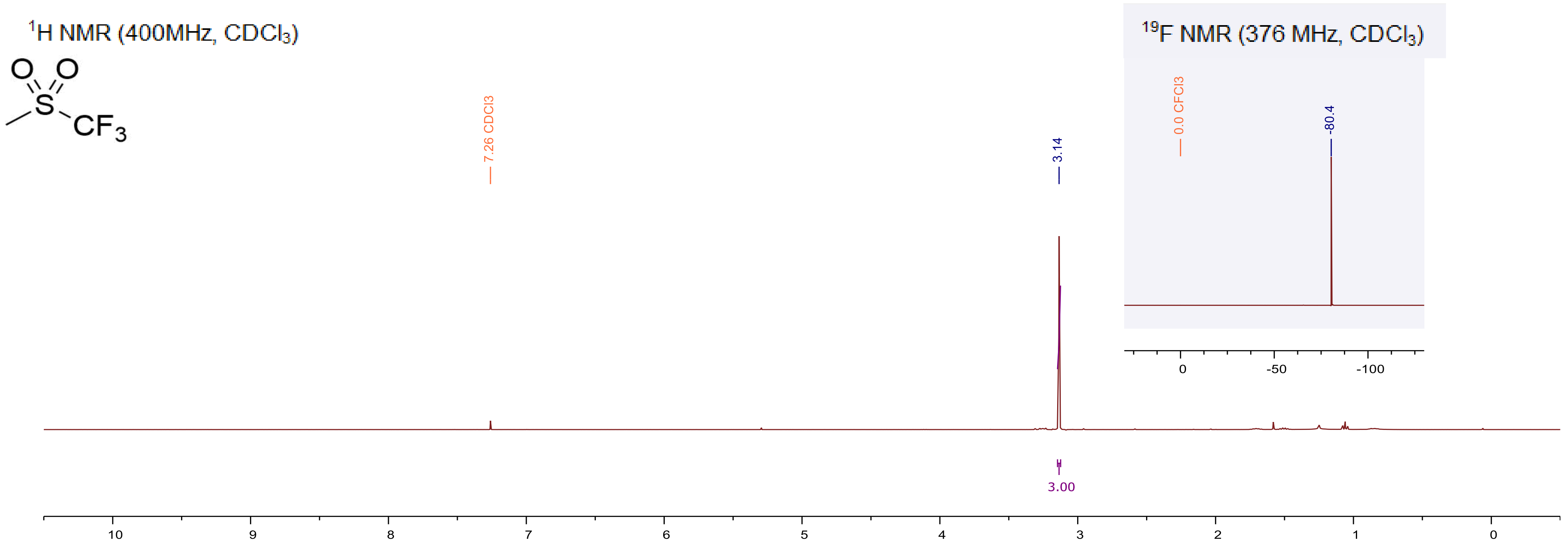

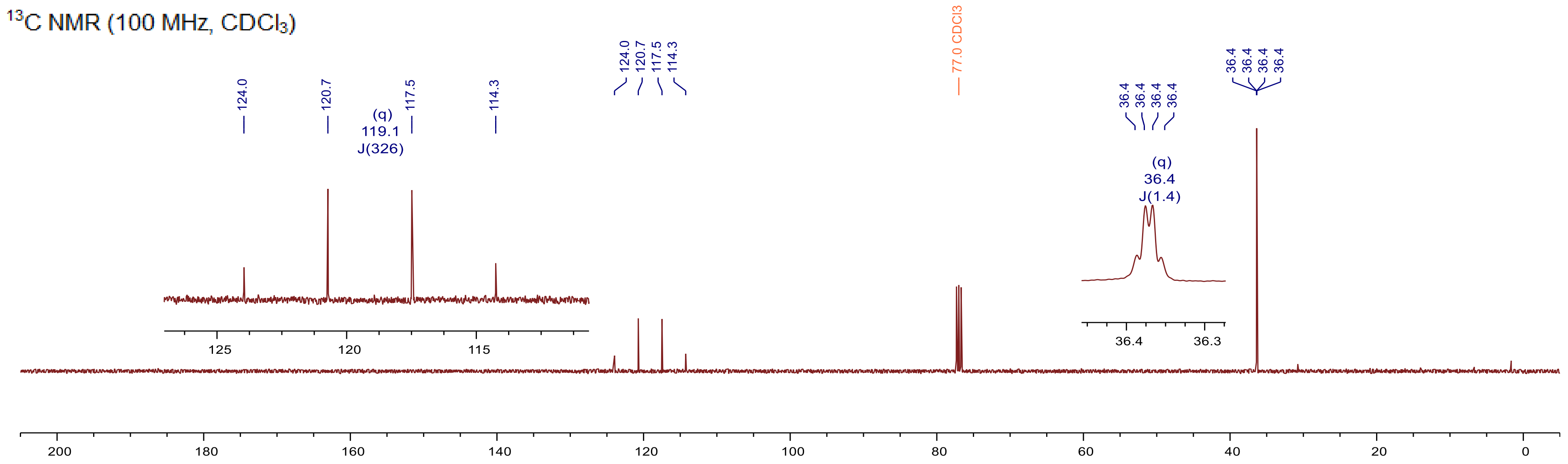

^{1}H NMR (400MHz, $CDCl_3$)

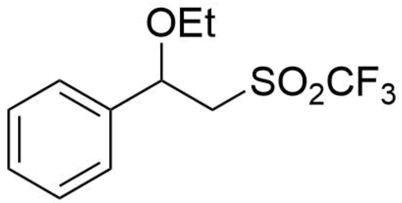

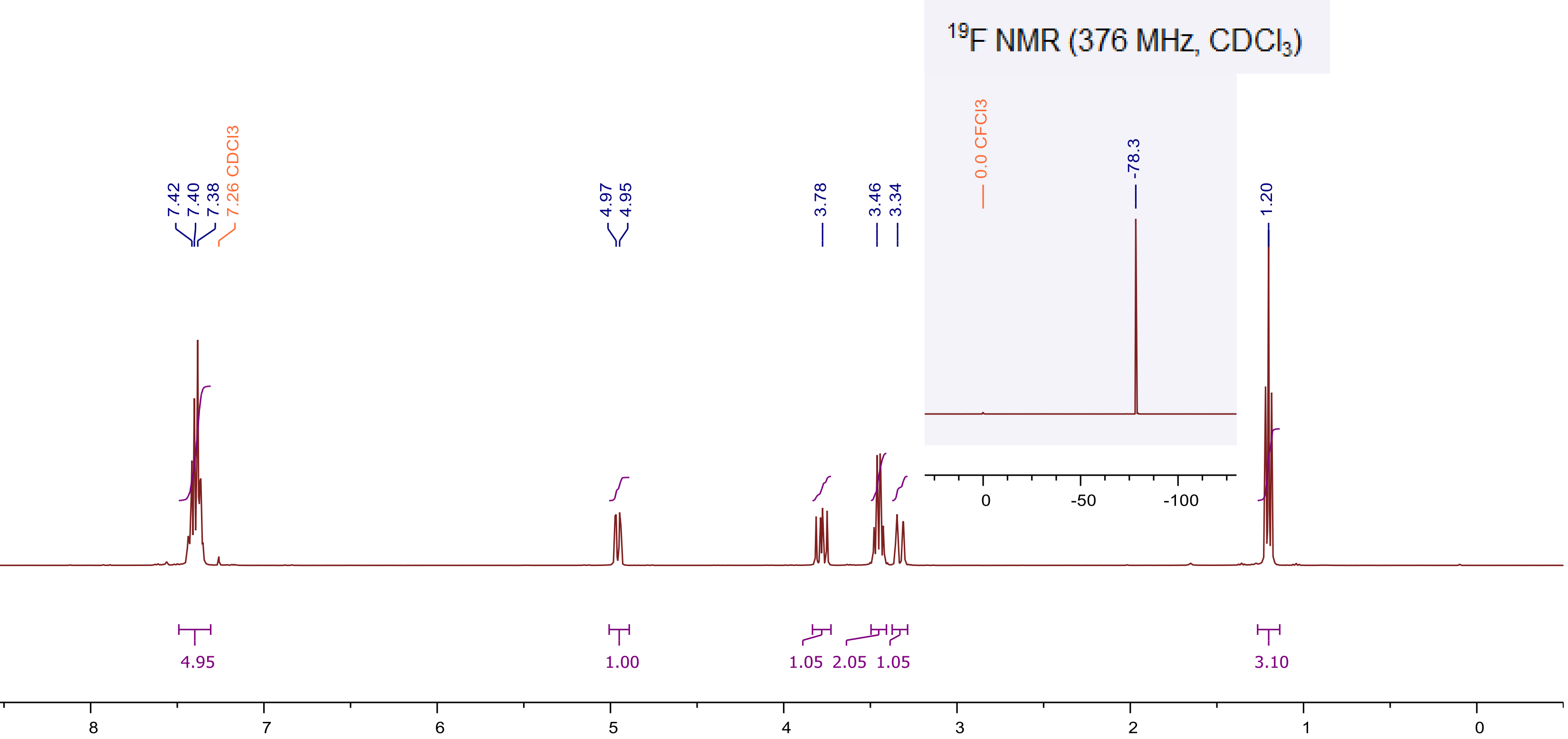

^{13}C NMR (100 MHz, $CDCl_3$)

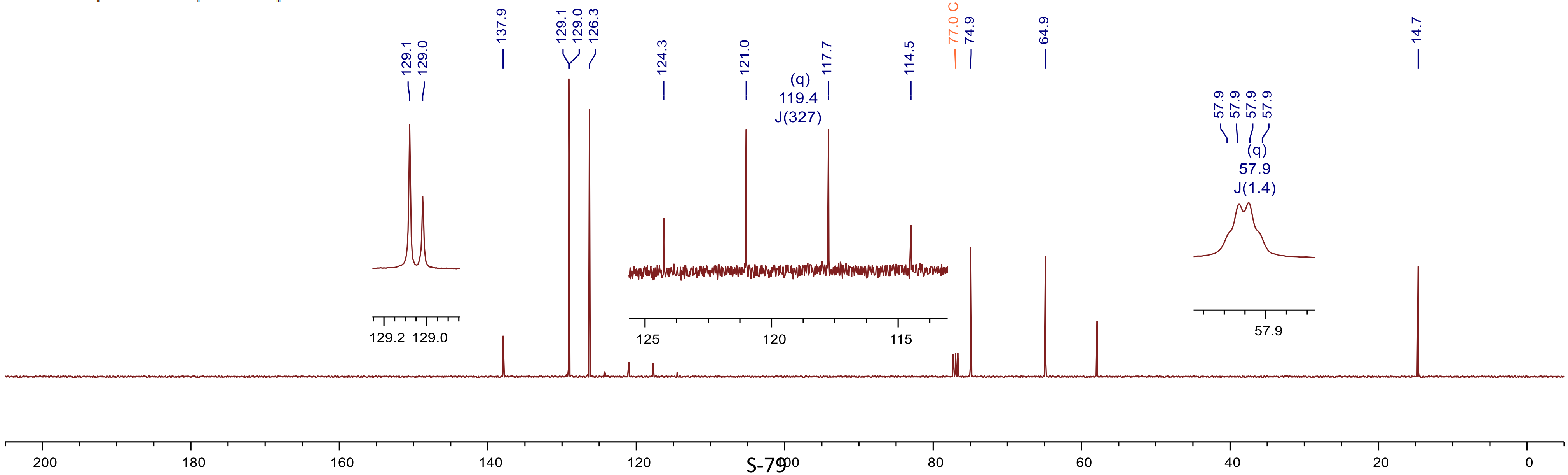

^{1}H NMR (400MHz, $CDCl_3$)

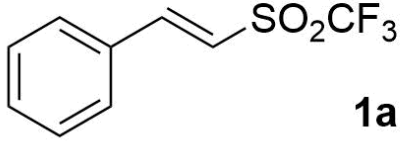

1a

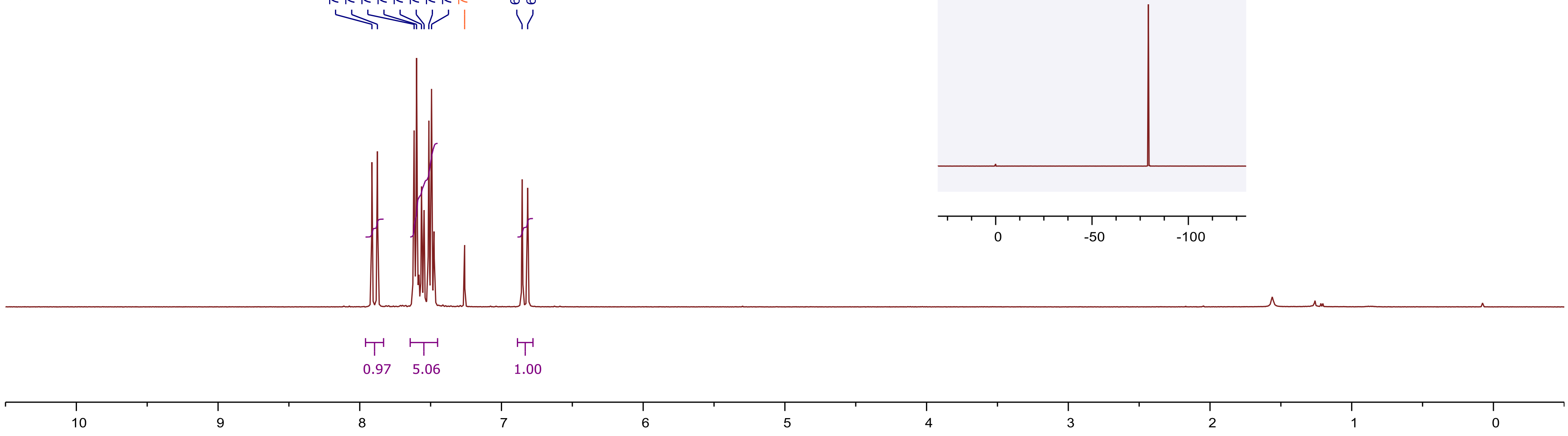

^{13}C NMR (100 MHz, $CDCl_3$)

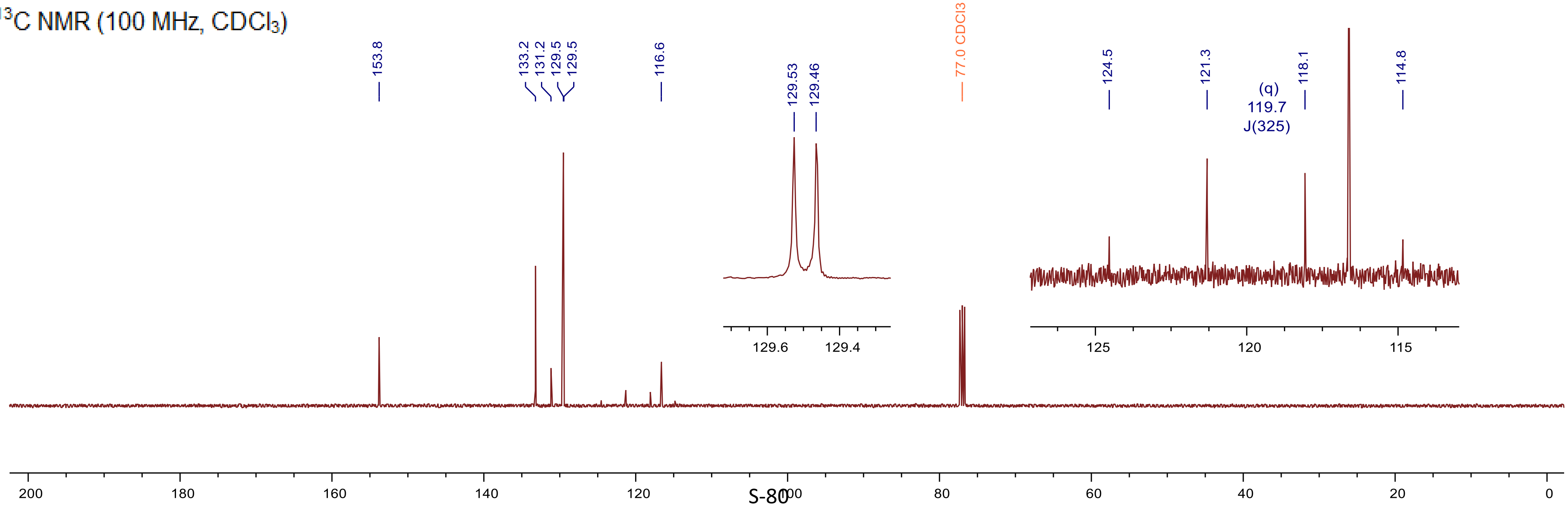

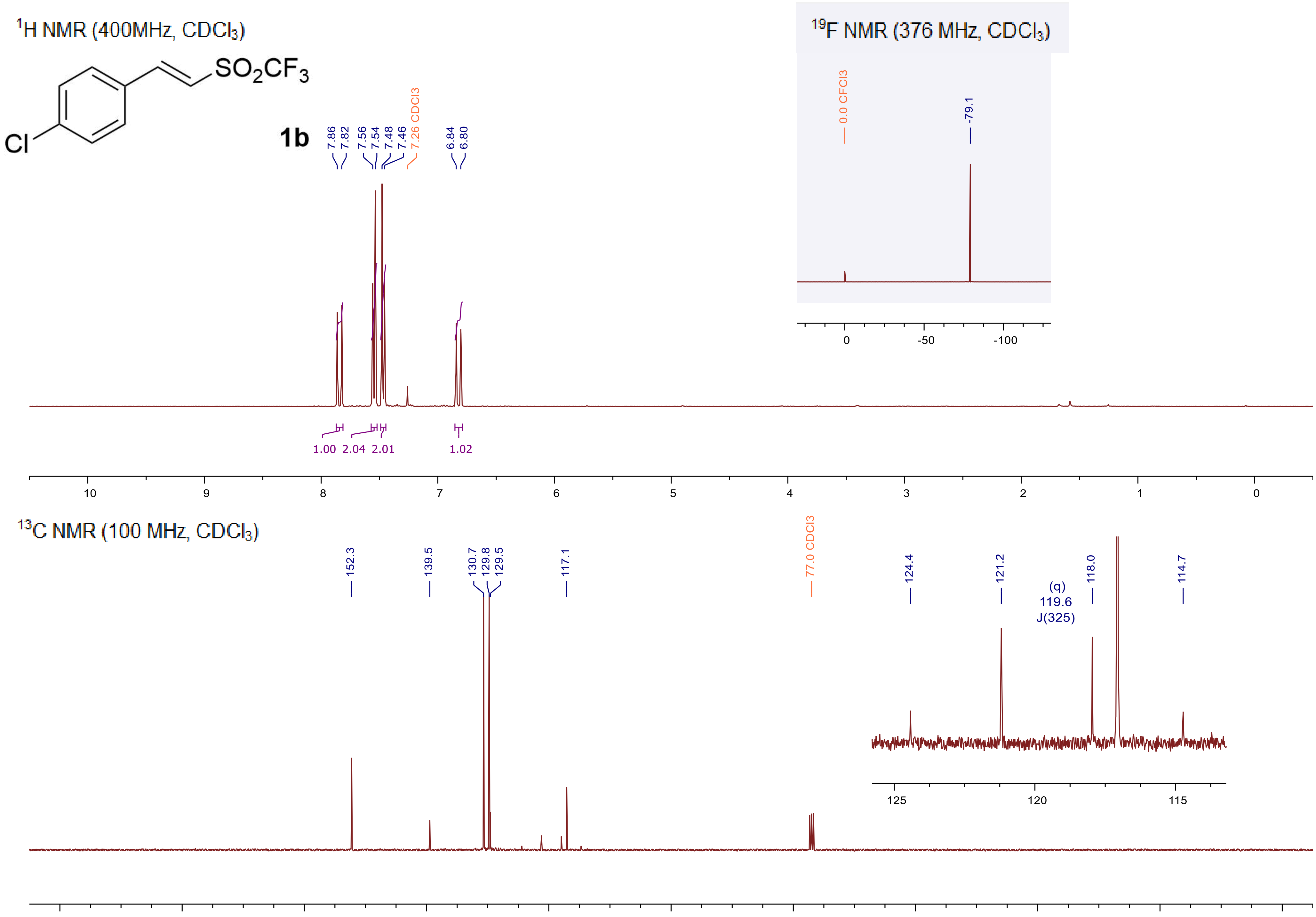

^{1}H NMR (400MHz, CDCl$_3$)
SO$_2$CF$_3$
Cl
1b
7.86
7.82
7.56
7.54
7.48
7.46
7.26 CDCl3
6.84
6.80
1.00
2.04
2.01
1.02
^{19}F NMR (376 MHz, CDCl$_3$)
0.0 CFCl3
-79.1
0
-50
-100
10
9
8
7
6
5
4
3
2
1
0
^{13}C NMR (100 MHz, CDCl$_3$)
152.3
139.5
130.7
129.8
129.5
117.1
77.0 CDCl3
124.4
121.2
(q)
119.6
J(325)
118.0
114.7
125
120
115
200
180
160
140
120
100
80
60
40
20
0

^1H NMR (400MHz, $CDCl_3$)

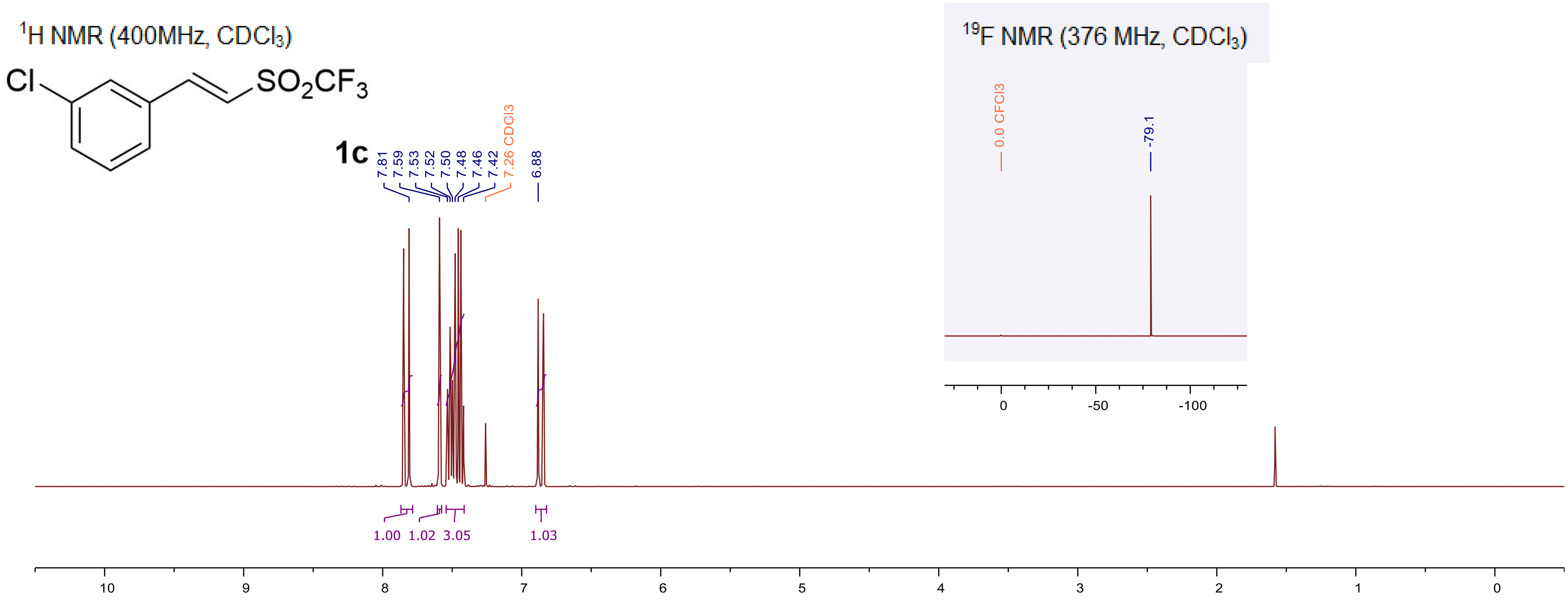

^{13}C NMR (100 MHz, $CDCl_3$)

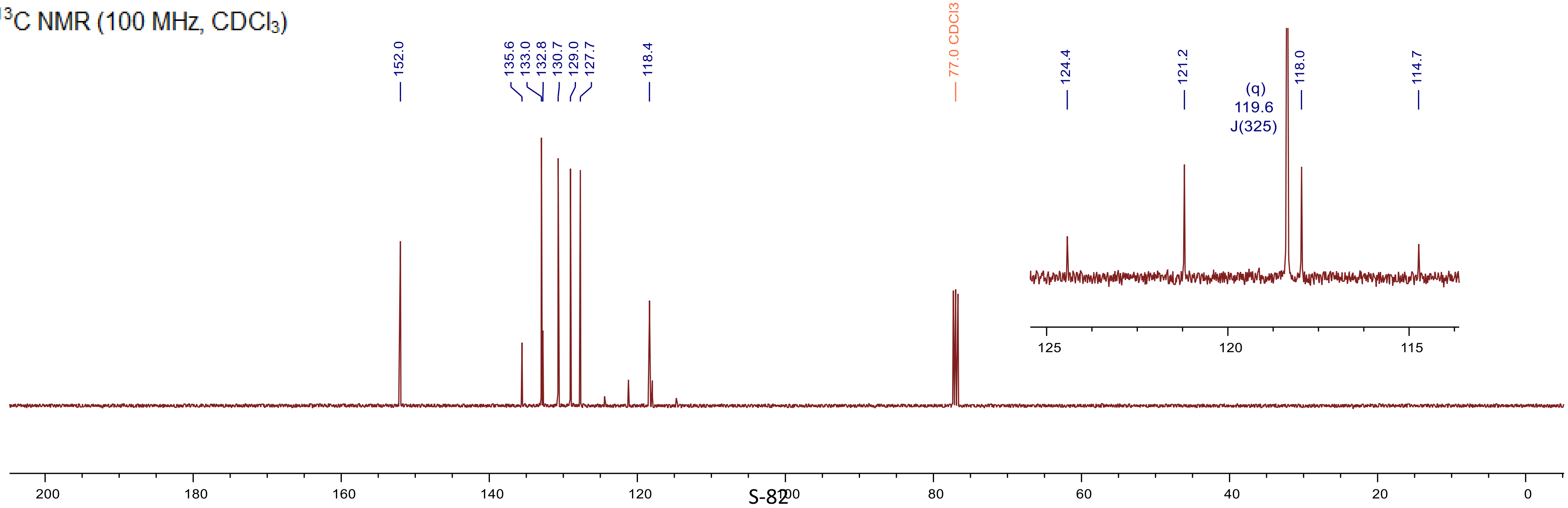

^{1}H NMR (400MHz, $CDCl_3$)

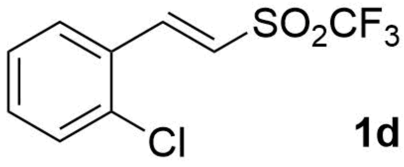

1d

^{19}F NMR (376 MHz, $CDCl_3$)

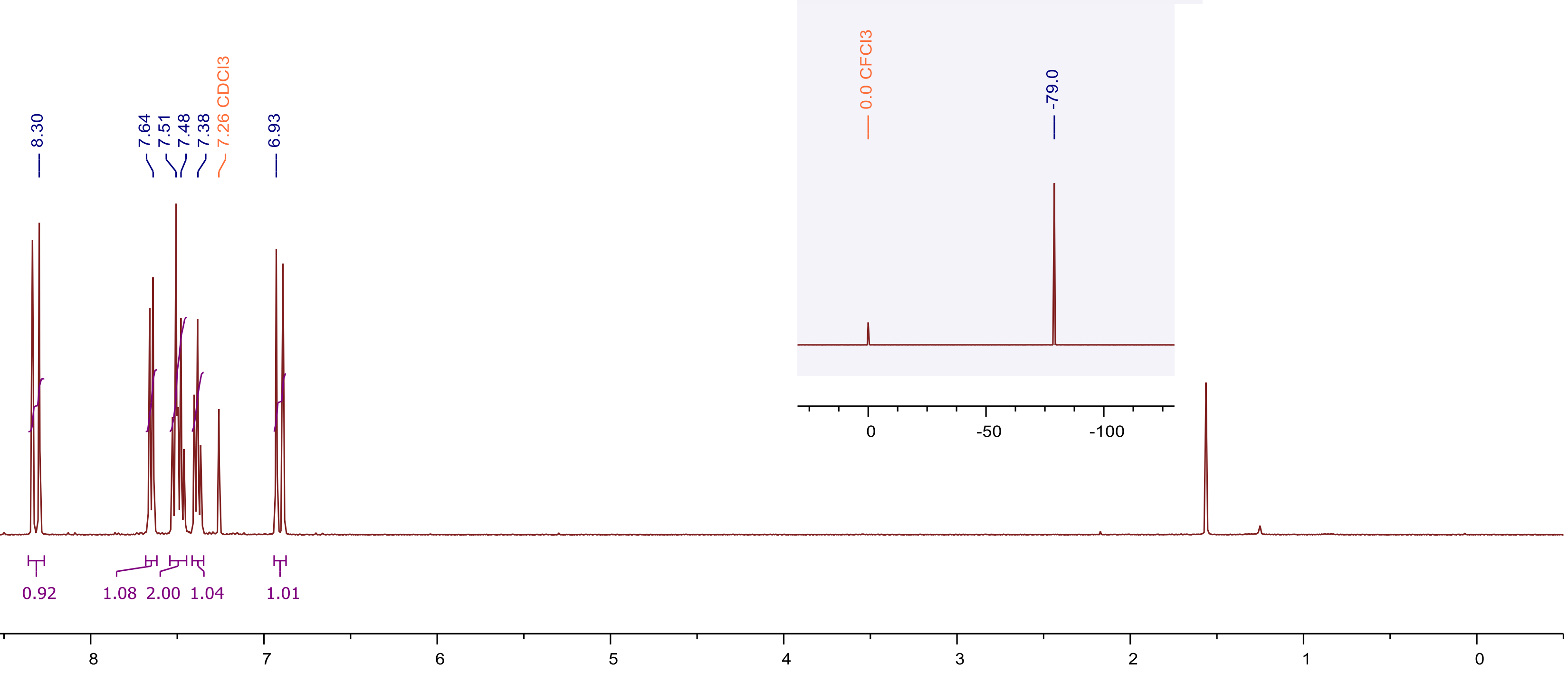

^{13}C NMR (100 MHz, $CDCl_3$)

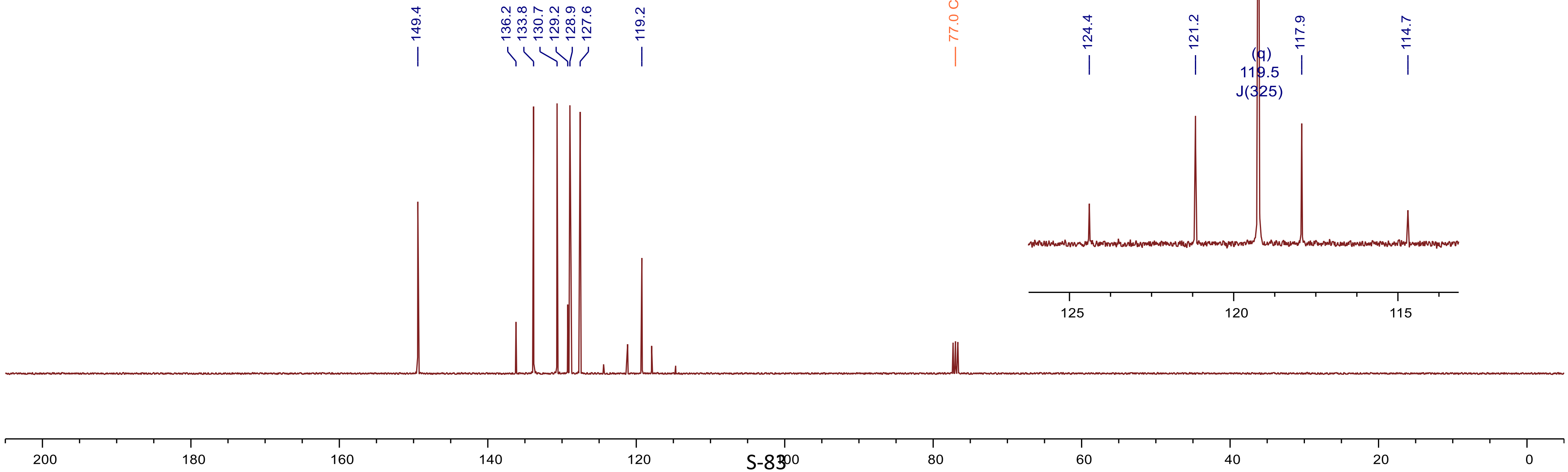

^{1}H NMR (400MHz, $CDCl_3$)

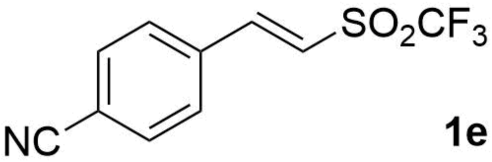

1e

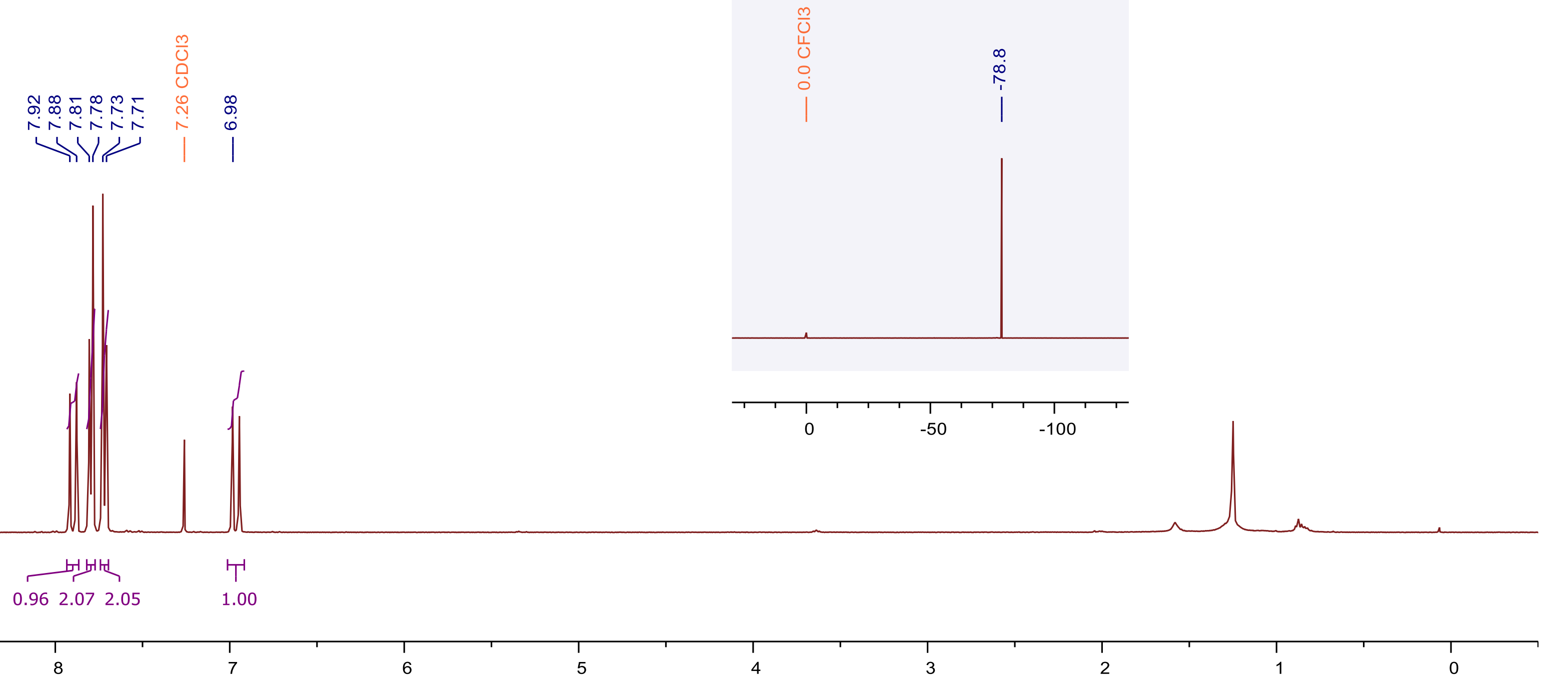

^{13}C NMR (100 MHz, $CDCl_3$)

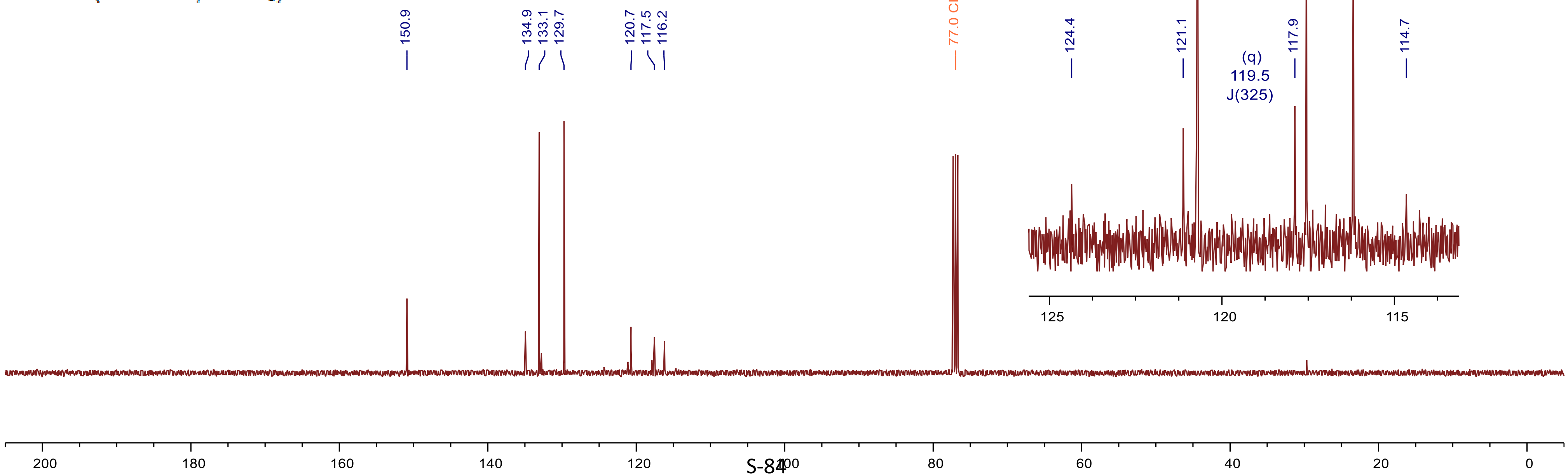

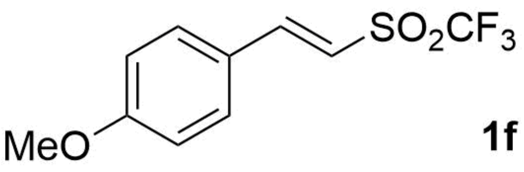
MeO
SO_2CF_3

1f

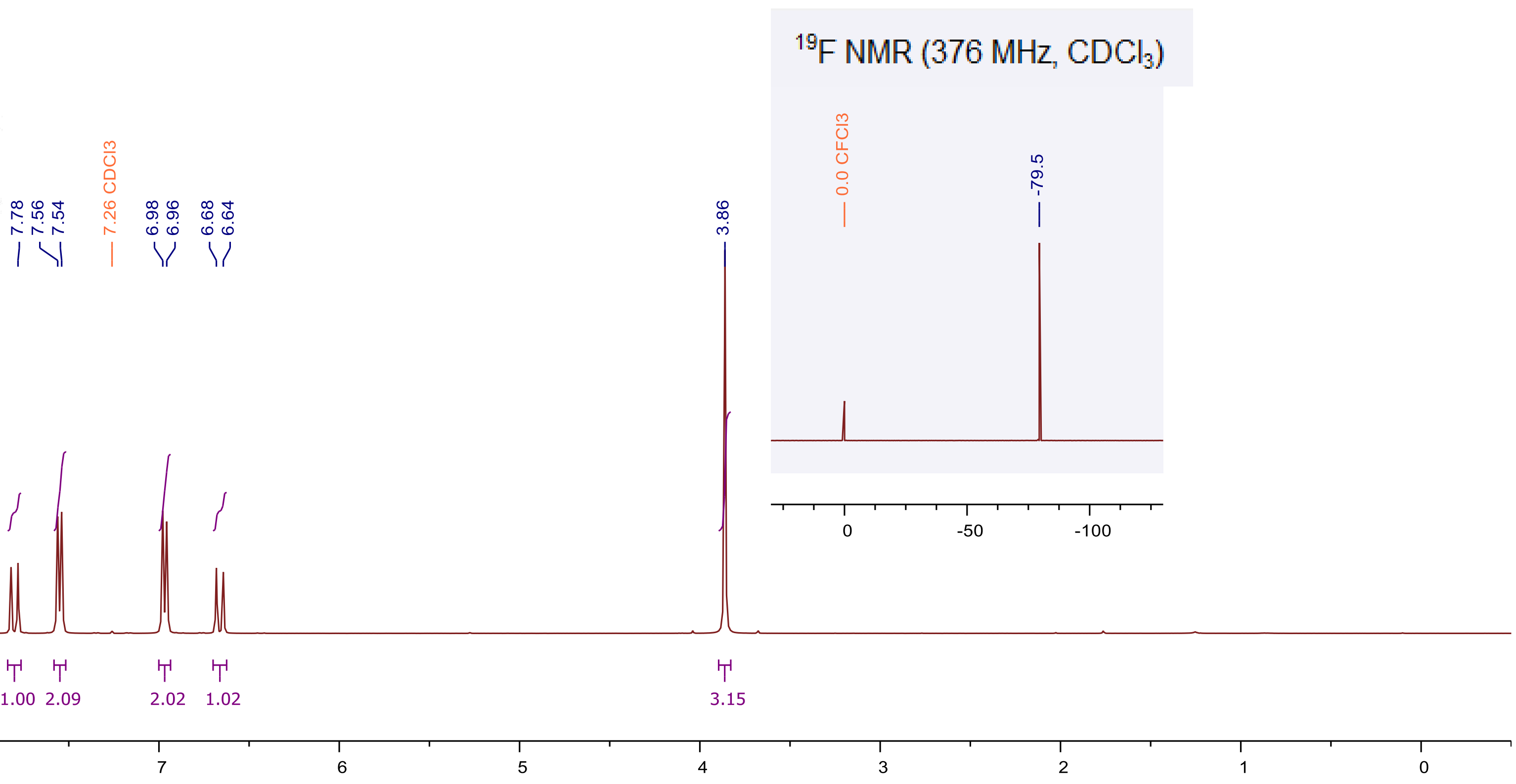
^{1}H NMR (400MHz, $CDCl_3$)
7.78
7.56
7.54
7.26 CDCl3
6.98
6.96
6.68
6.64
3.86
1.00
2.09
2.02
1.02
3.15
10
9
8
7
6
5
4
3
2
1
0
^{19}F NMR (376 MHz, $CDCl_3$)
0.0 CFCl3
-79.5
0
-50
-100

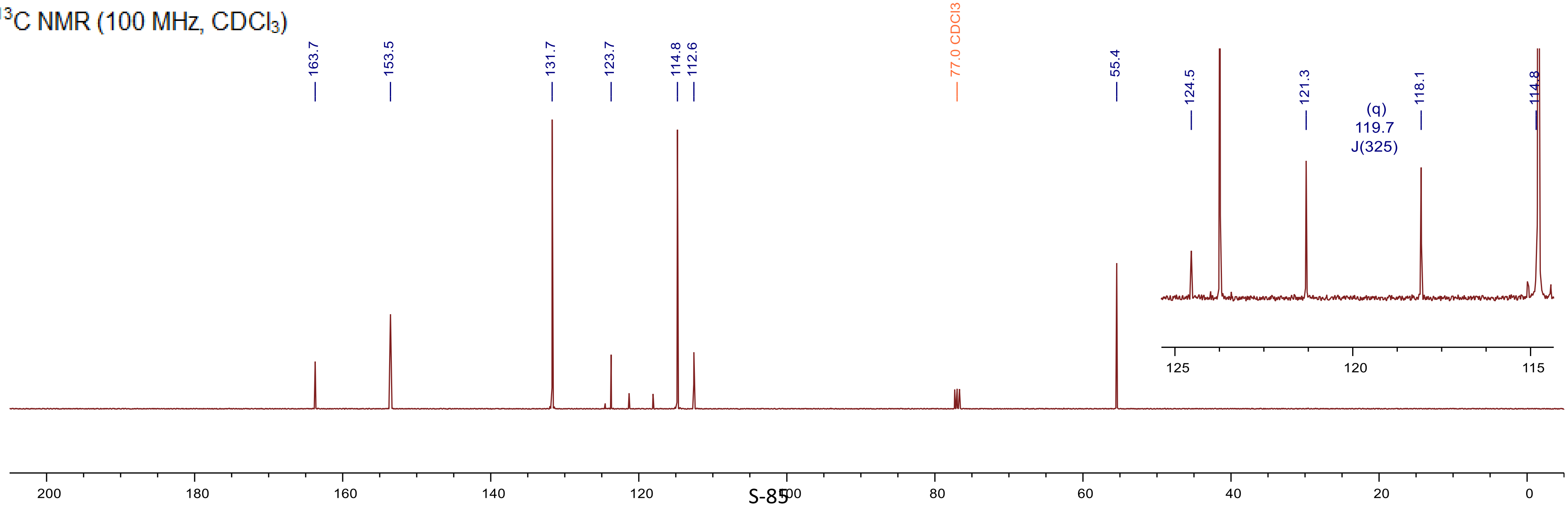
^{13}C NMR (100 MHz, $CDCl_3$)
163.7
153.5
131.7
123.7
114.8
112.6
77.0 CDCl3
55.4
124.5
121.3
(q)
119.7
J(325)
118.1
114.8
125
120
115
200
180
160
140
120
100
80
60
40
20
0

^{1}H NMR (400MHz, $CDCl_3$)

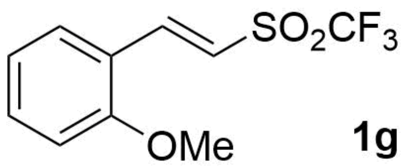

1g

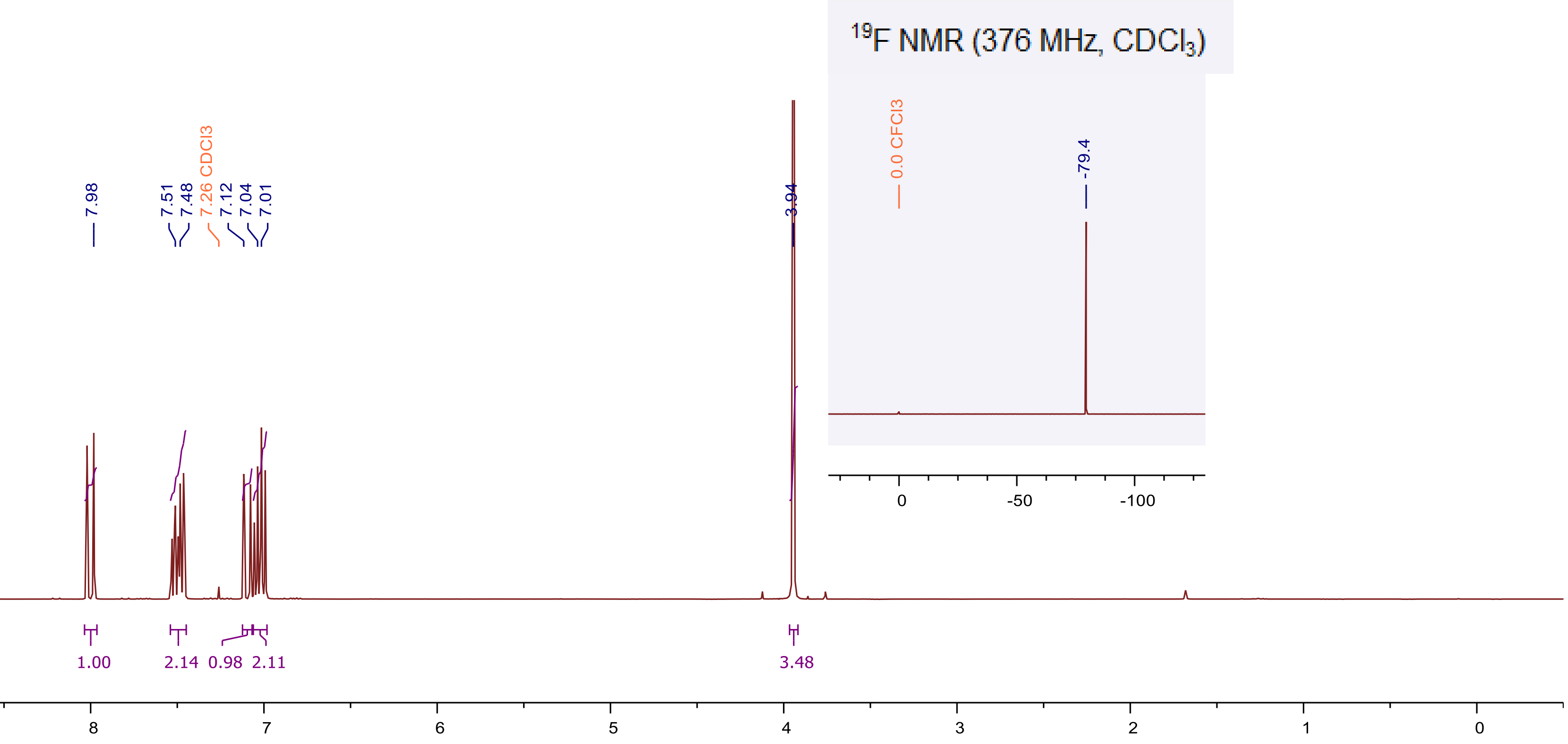

^{13}C NMR (100 MHz, $CDCl_3$)

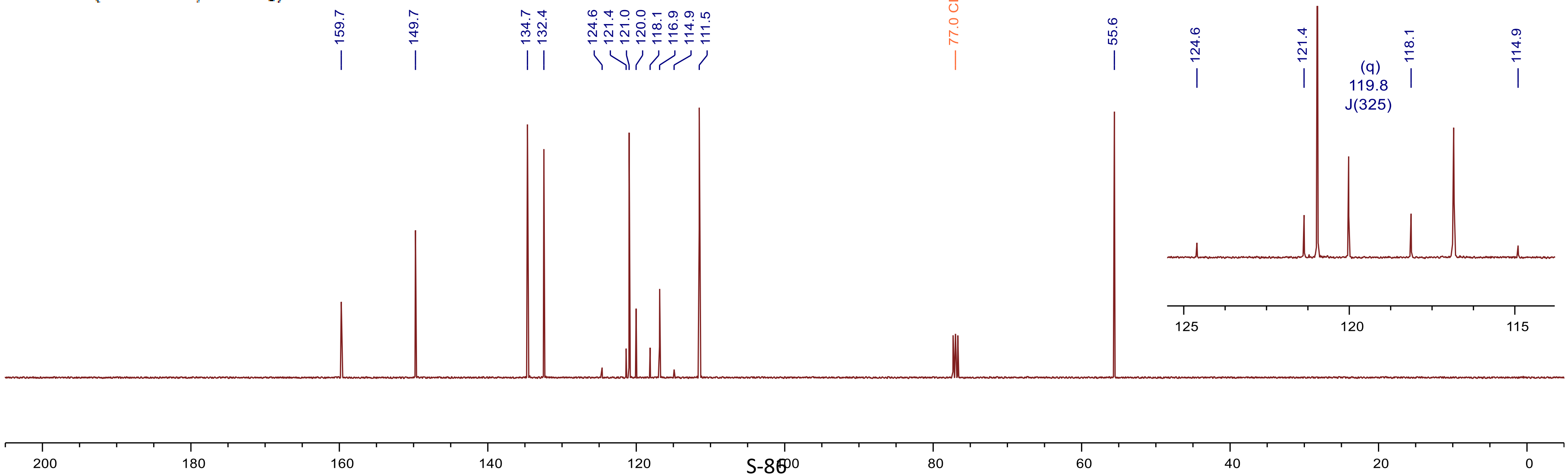

^{1}H NMR (400MHz, $CDCl_3$)

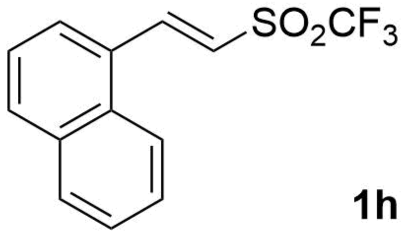

1h

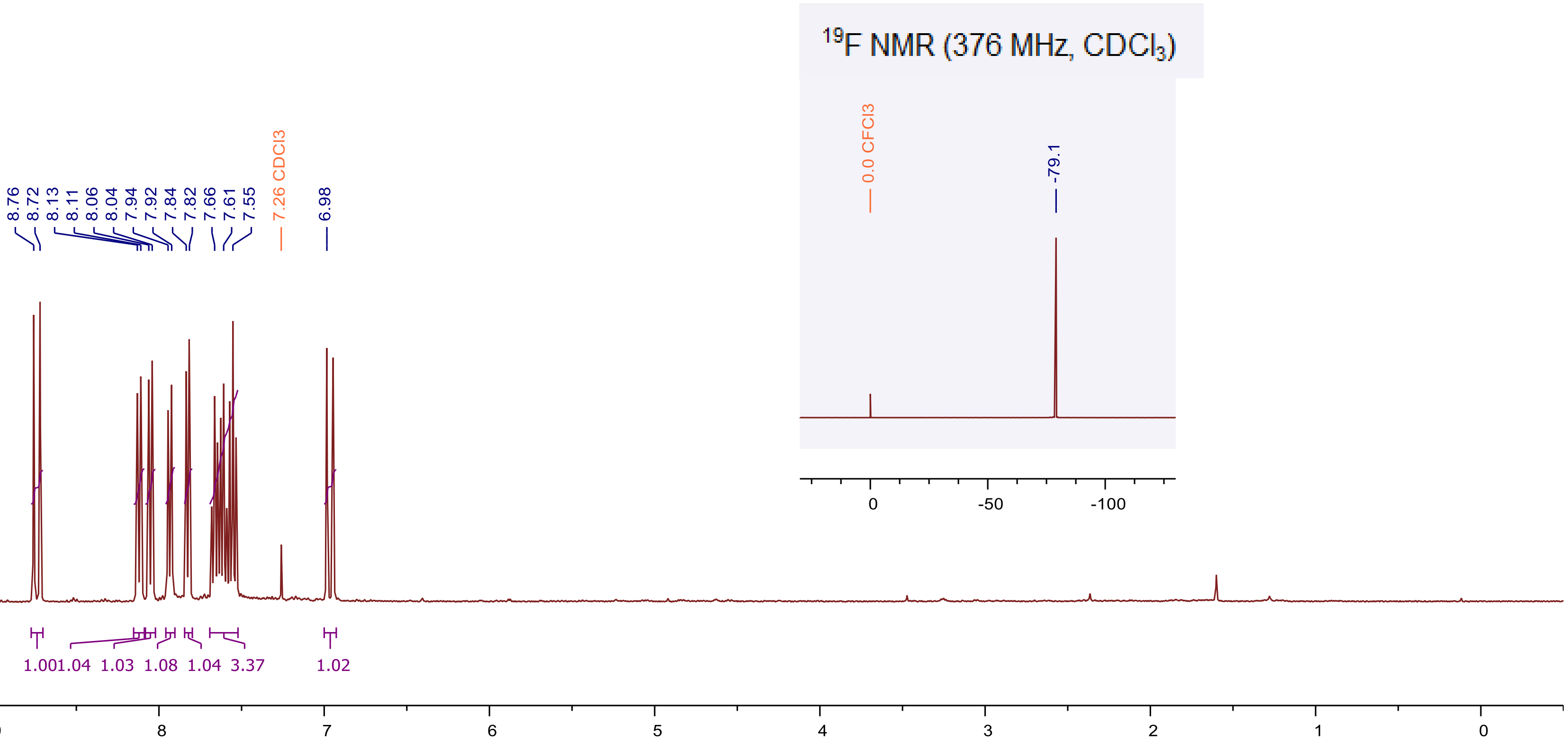

^{13}C NMR (100 MHz, $CDCl_3$)

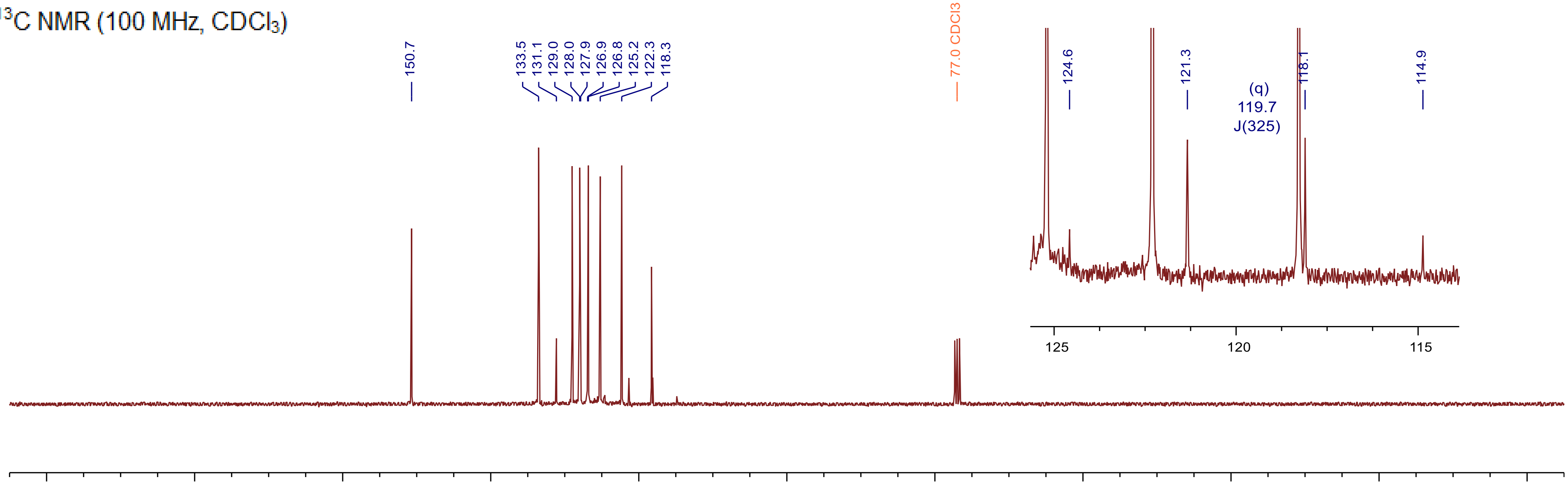

^{1}H NMR (400MHz, $CDCl_3$)

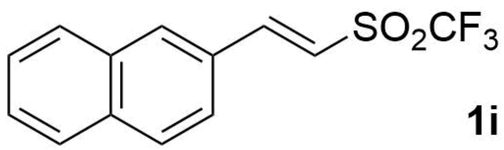

1i

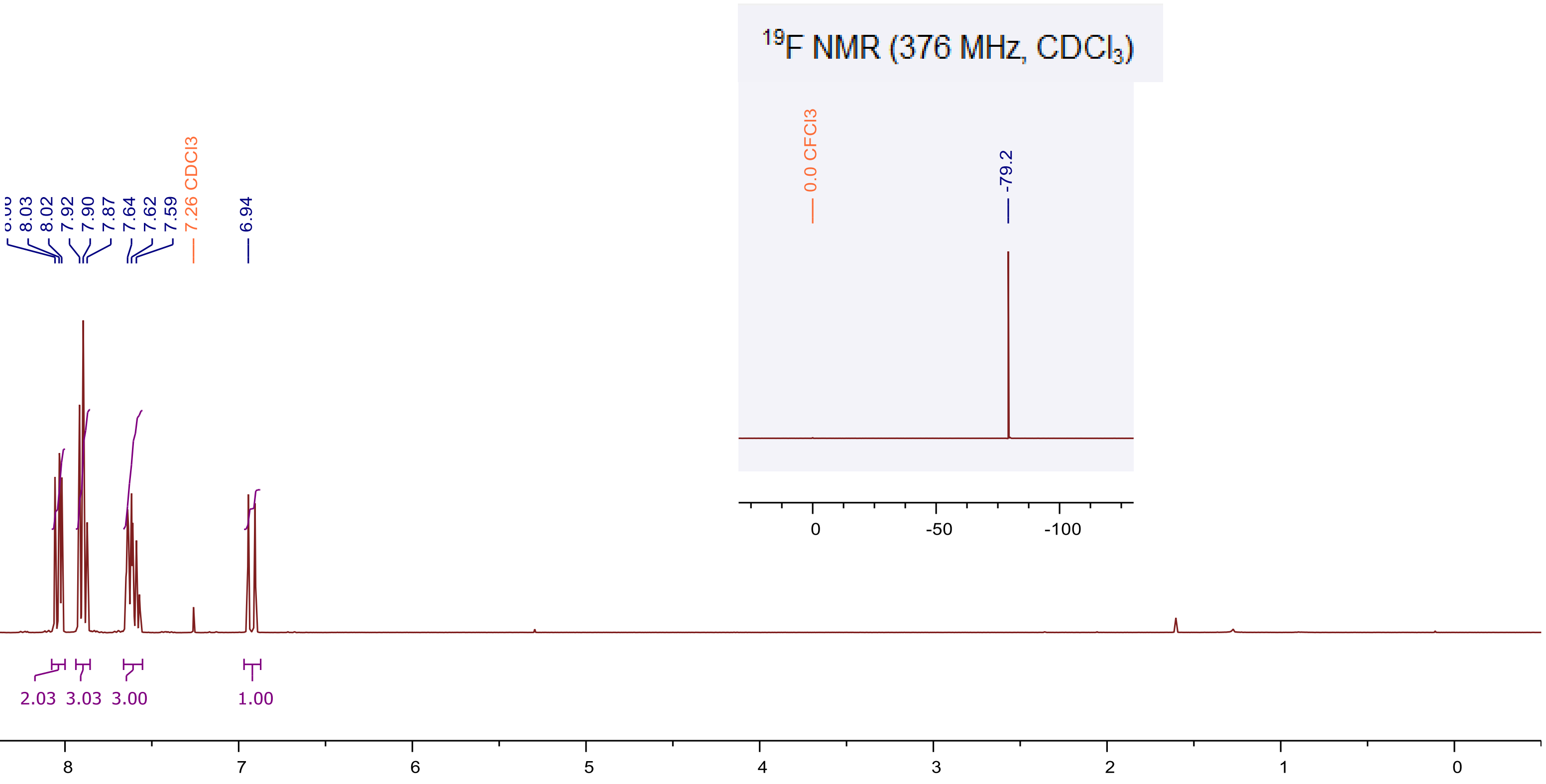

^{13}C NMR (100 MHz, $CDCl_3$)

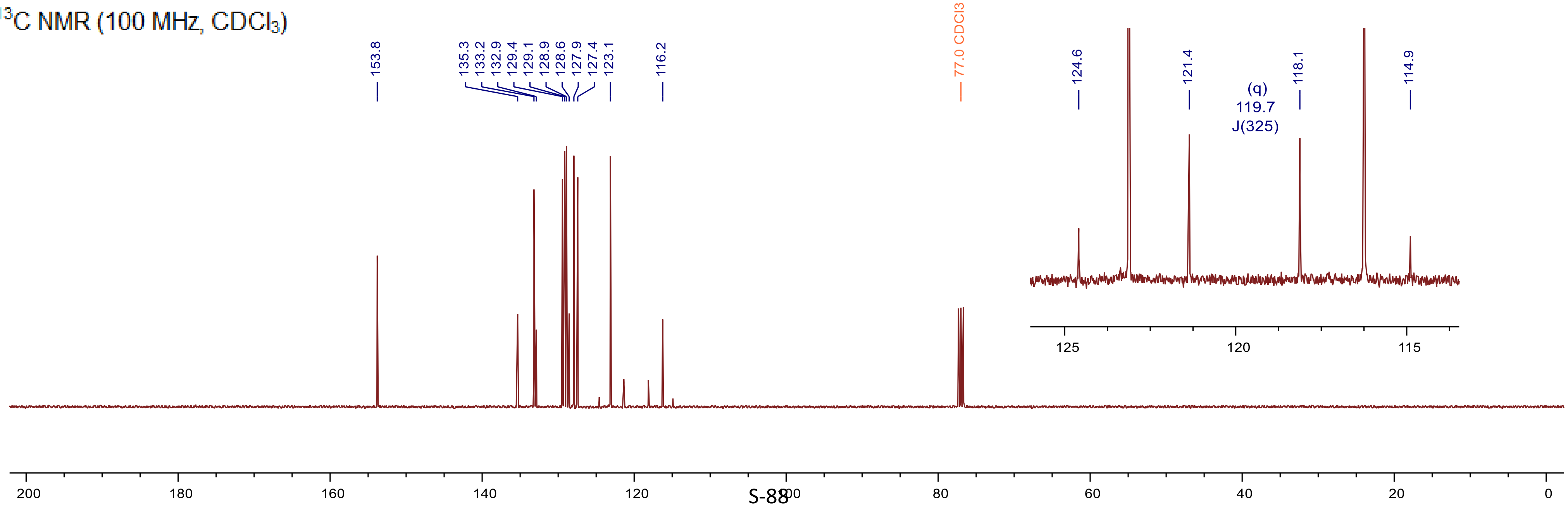

^{1}H NMR (400MHz, $CDCl_3$)

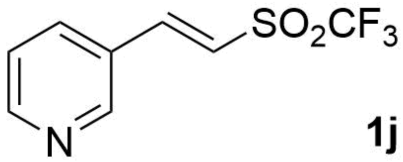

1j

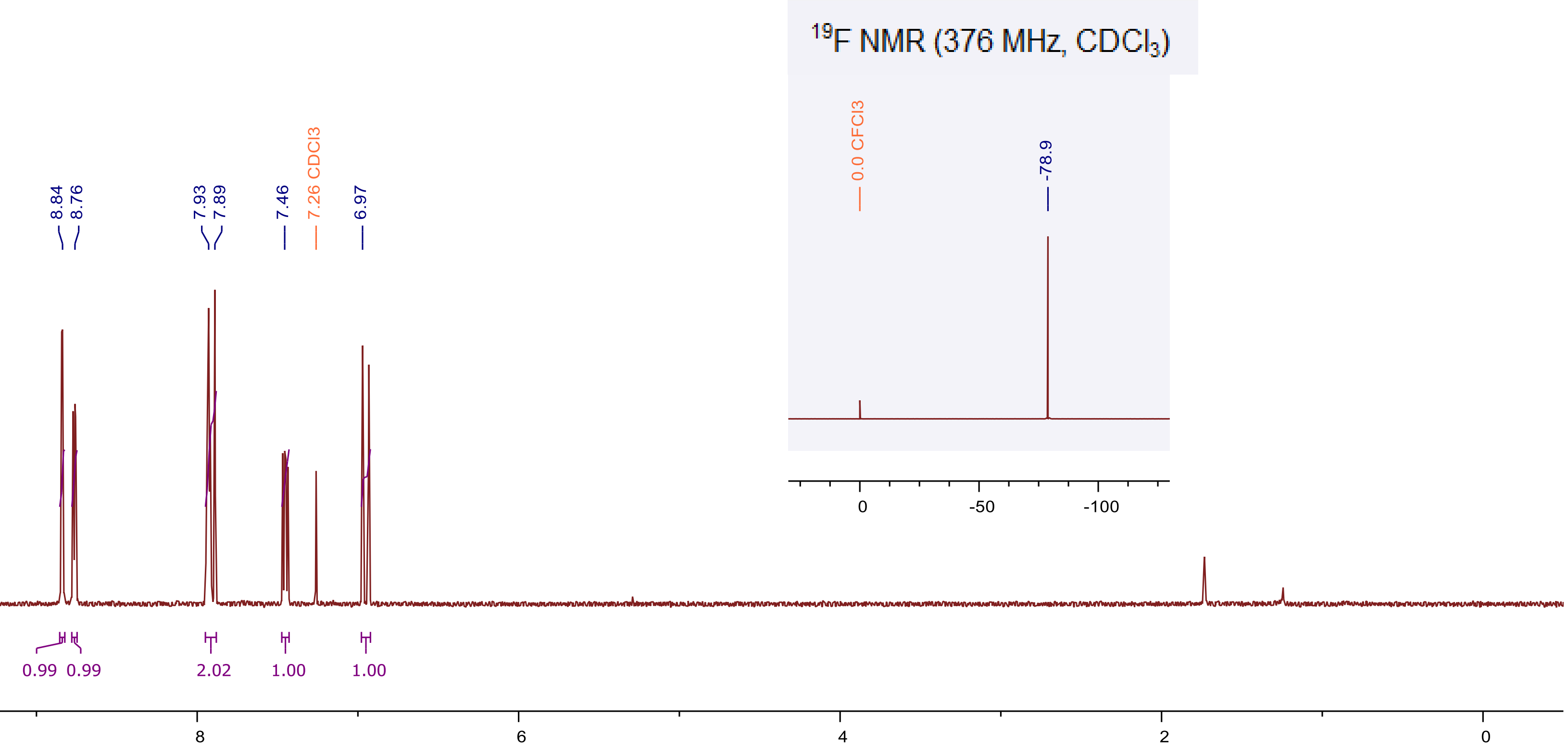

^{13}C NMR (100 MHz, $CDCl_3$)

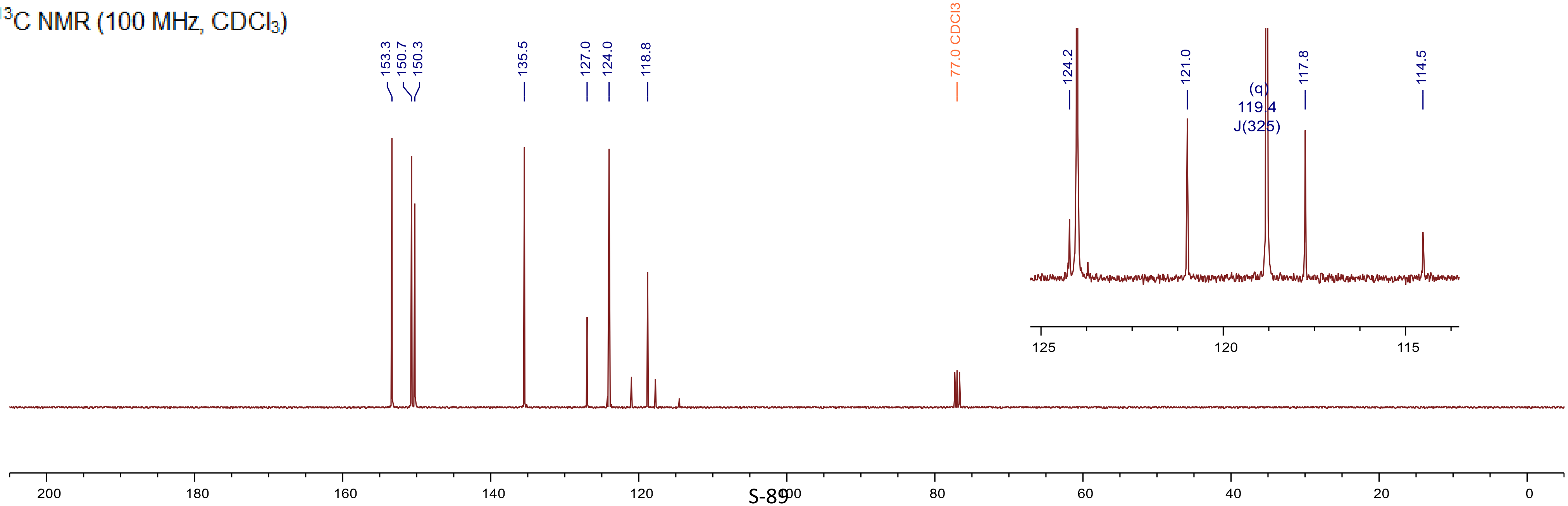

^{1}H NMR (400MHz, $CDCl_3$)

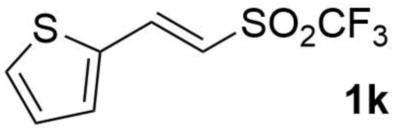

1k

^{19}F NMR (376 MHz, $CDCl_3$)

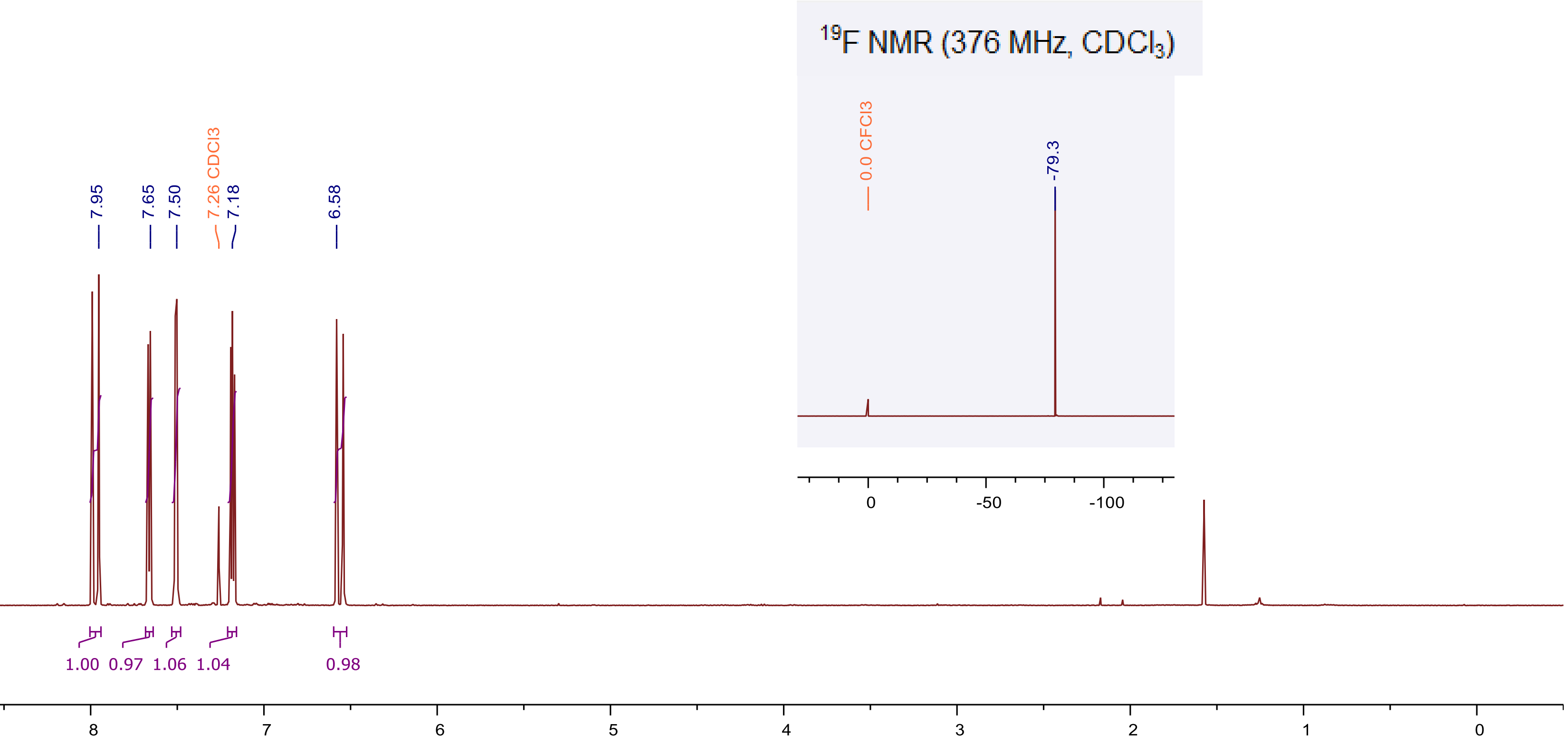

^{13}C NMR (100 MHz, $CDCl_3$)

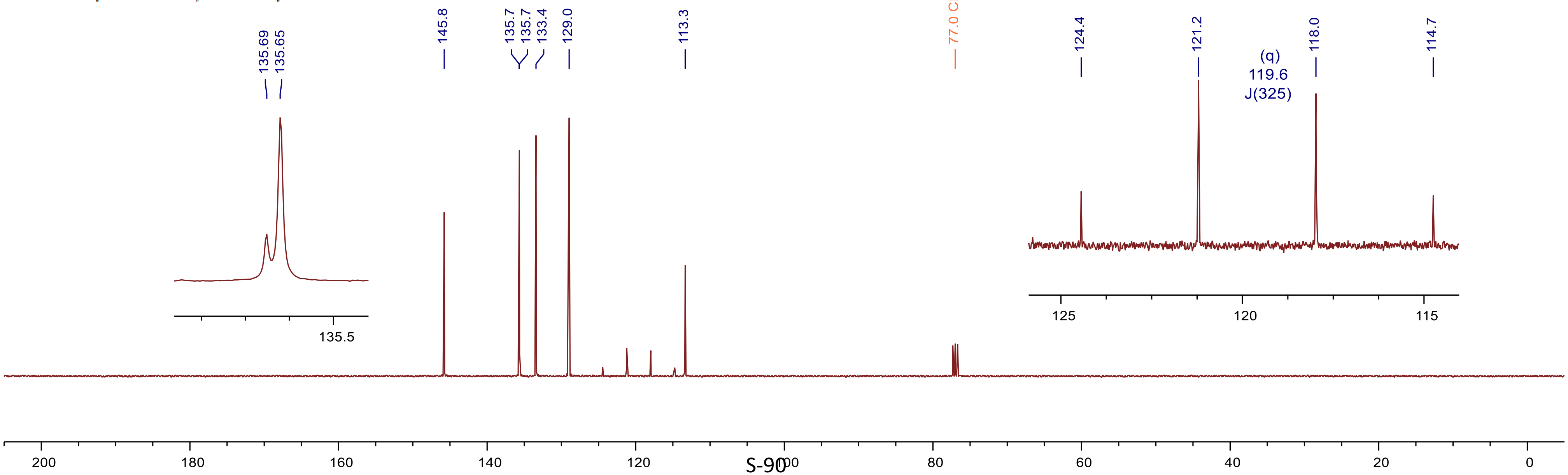

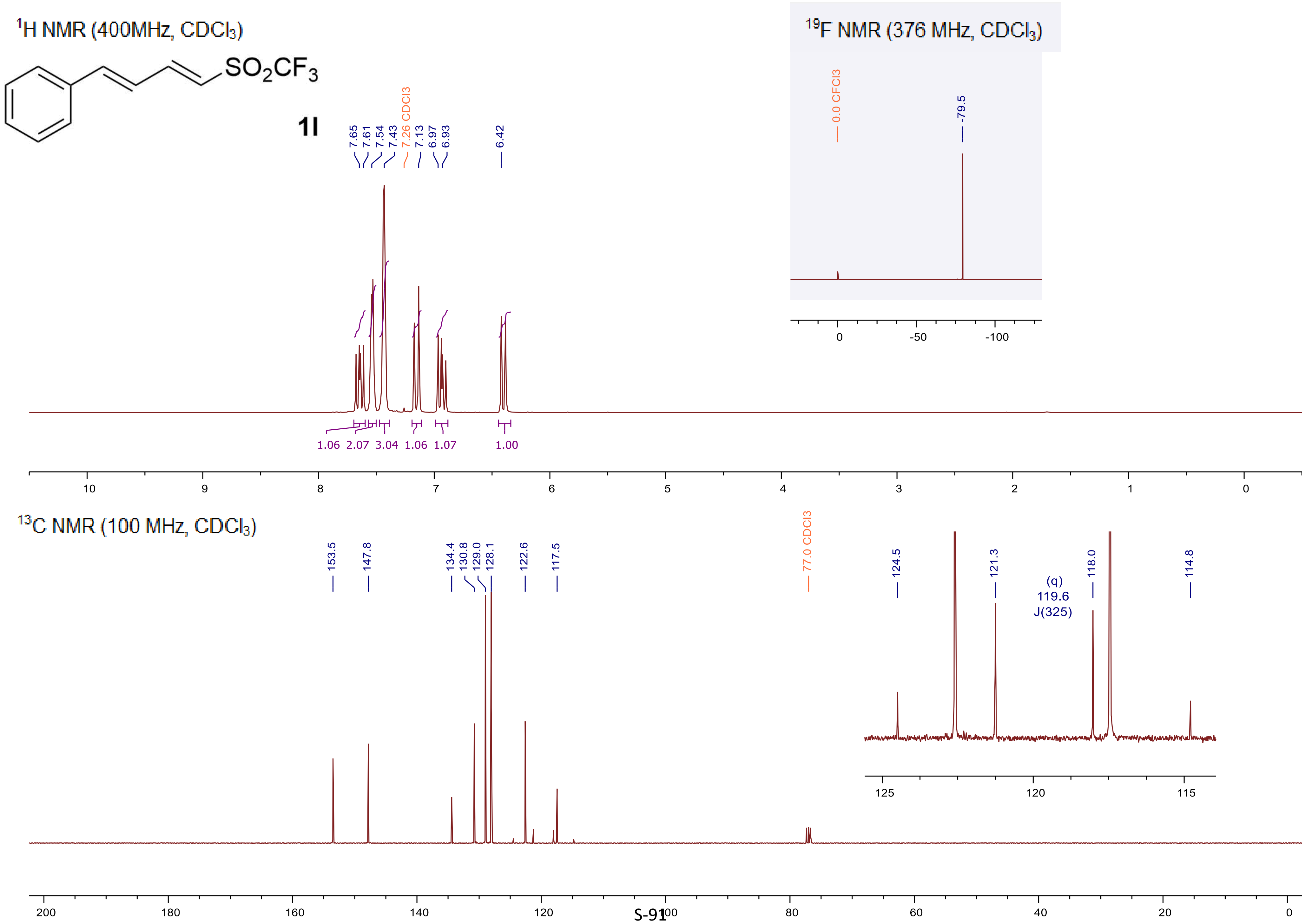

1H NMR (400MHz, CDCl3)
SO2CF3
1l
7.65
7.61
7.54
7.43
7.26 CDCl3
7.13
6.97
6.93
6.42
1.06 2.07 3.04 1.06 1.07 1.00
10 9 8 7 6 5 4 3 2 1 0
19F NMR (376 MHz, CDCl3)
0.0 CFCl3
-79.5
0 -50 -100
13C NMR (100 MHz, CDCl3)
153.5
147.8
134.4
130.8
129.0
128.1
122.6
117.5
77.0 CDCl3
124.5
121.3
(q)
119.6
J(325)
118.0
114.8
125 120 115
200 180 160 140 120 100 80 60 40 20 0

^{1}H NMR (400MHz, $CDCl_3$)

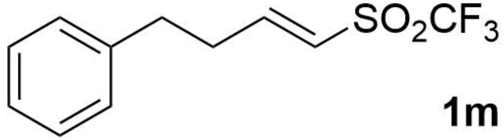

1m

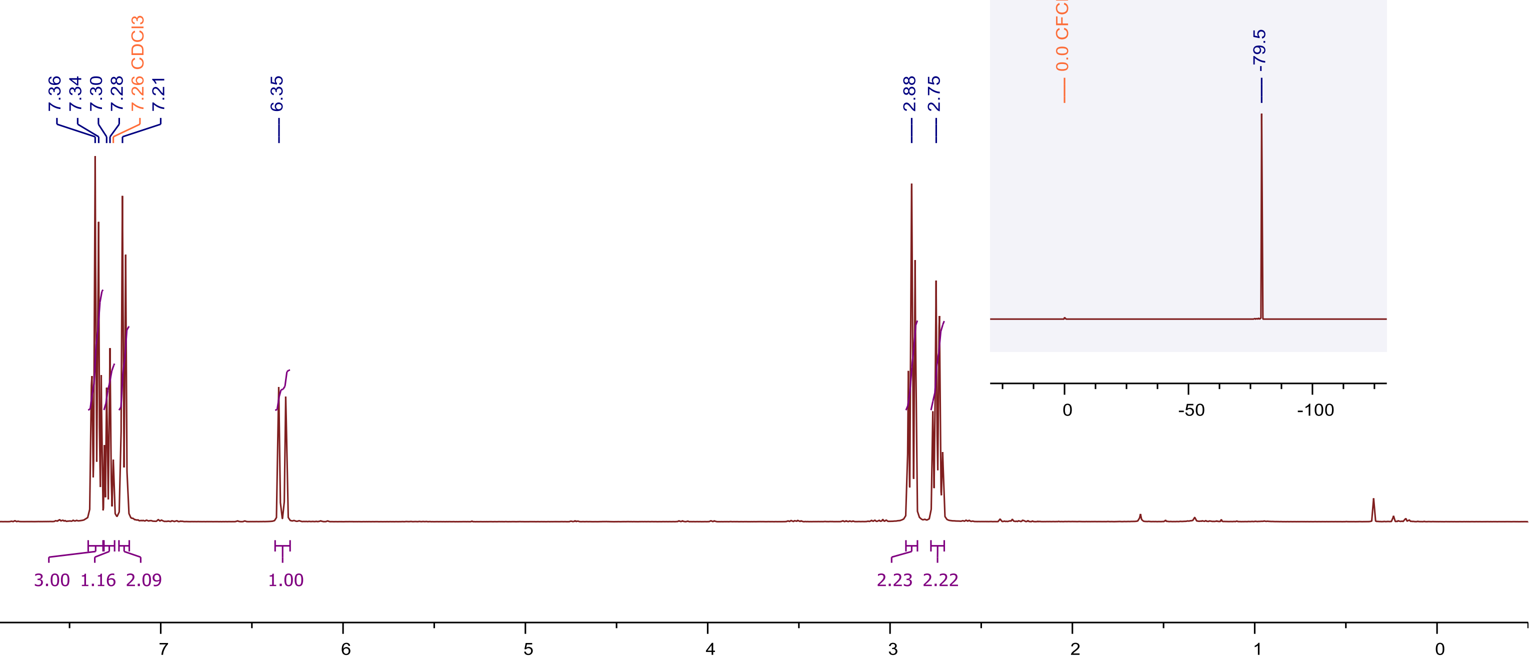

^{13}C NMR (100 MHz, $CDCl_3$)

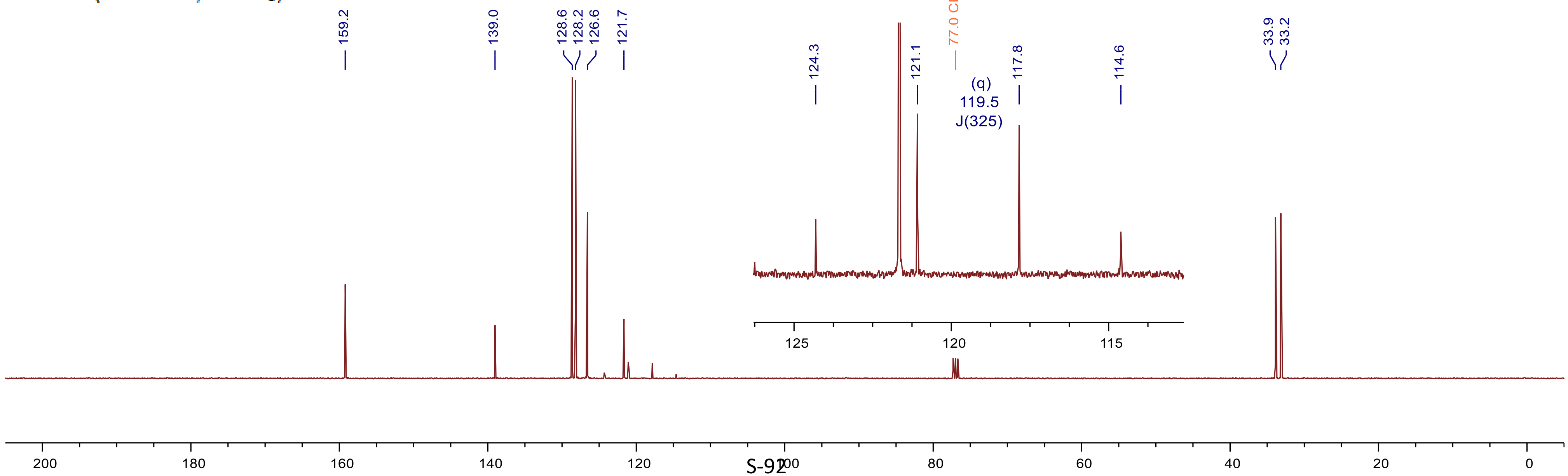

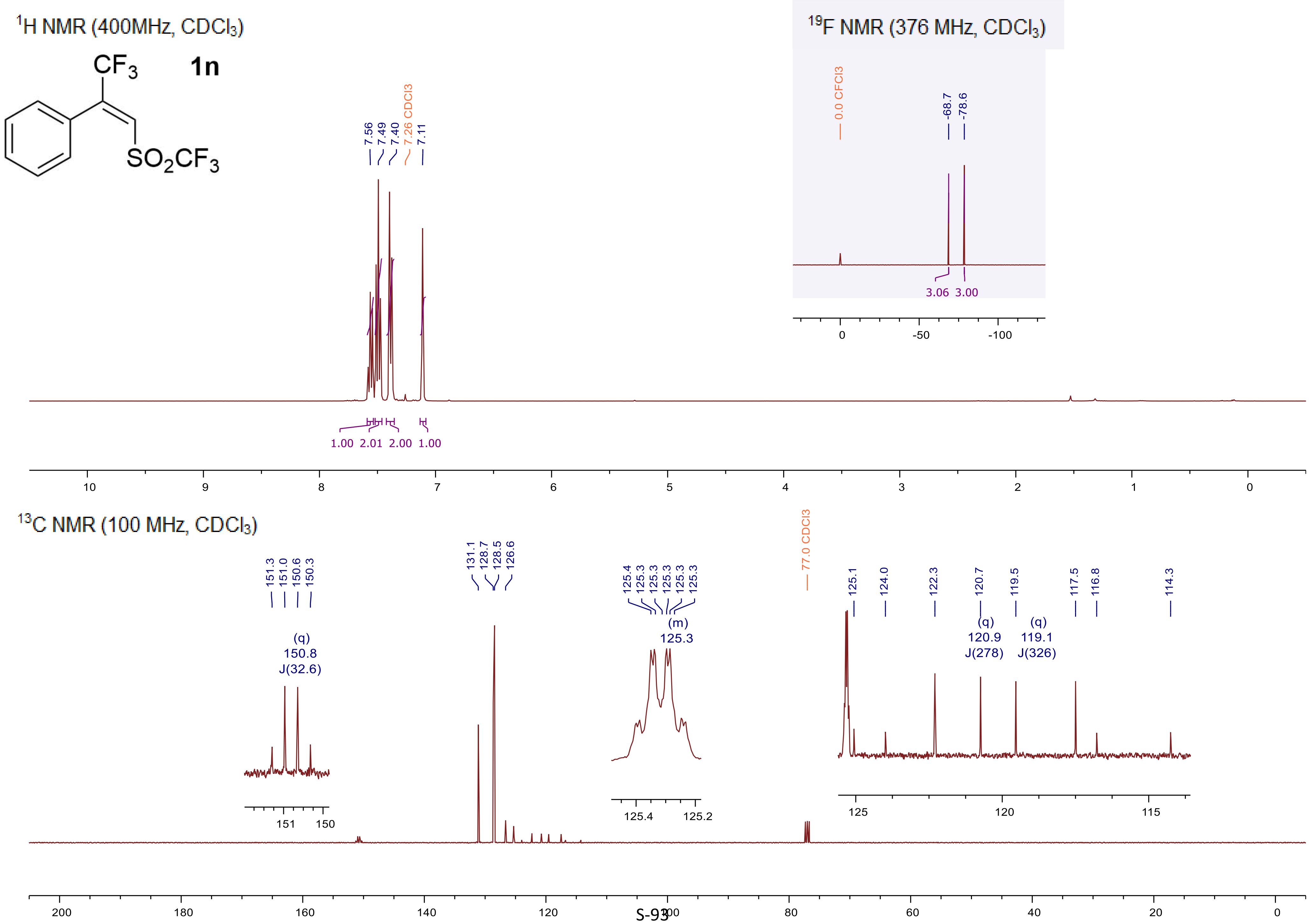

1H NMR (400MHz, CDCl3)
CF3
1n
SO2CF3
7.56
7.49
7.40
7.26 CDCl3
7.11
1.00 2.01 2.00 1.00
10 9 8 7 6 5 4 3 2 1 0
19F NMR (376 MHz, CDCl3)
0.0 CFCl3
-68.7
-78.6
3.06 3.00
0 -50 -100
13C NMR (100 MHz, CDCl3)
151.3
151.0
150.6
150.3
(q)
150.8
J(32.6)
151 150
131.1
128.7
128.5
126.6
125.4
125.3
125.3
125.3
125.3
125.3
(m)
125.3
125.4 125.2
77.0 CDCl3
125.1
124.0
122.3
120.7
119.5
117.5
116.8
114.3
(q)
120.9
J(278)
(q)
119.1
J(326)
125 120 115
200 180 160 140 120 100 80 60 40 20 0

^{1}H NMR (400MHz, $CDCl_3$)

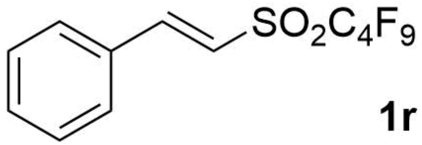

1r

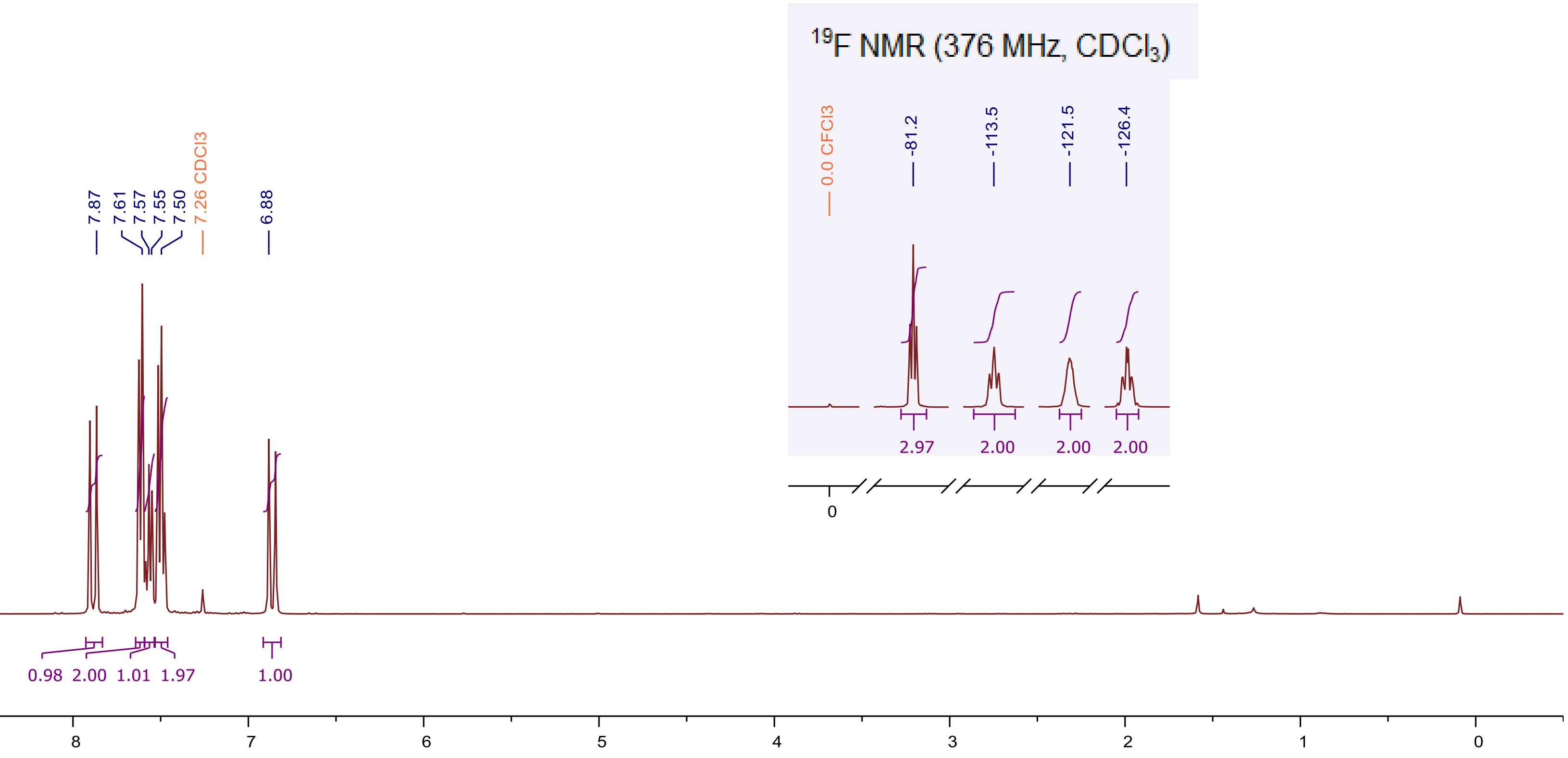

^{13}C NMR (100 MHz, $CDCl_3$)

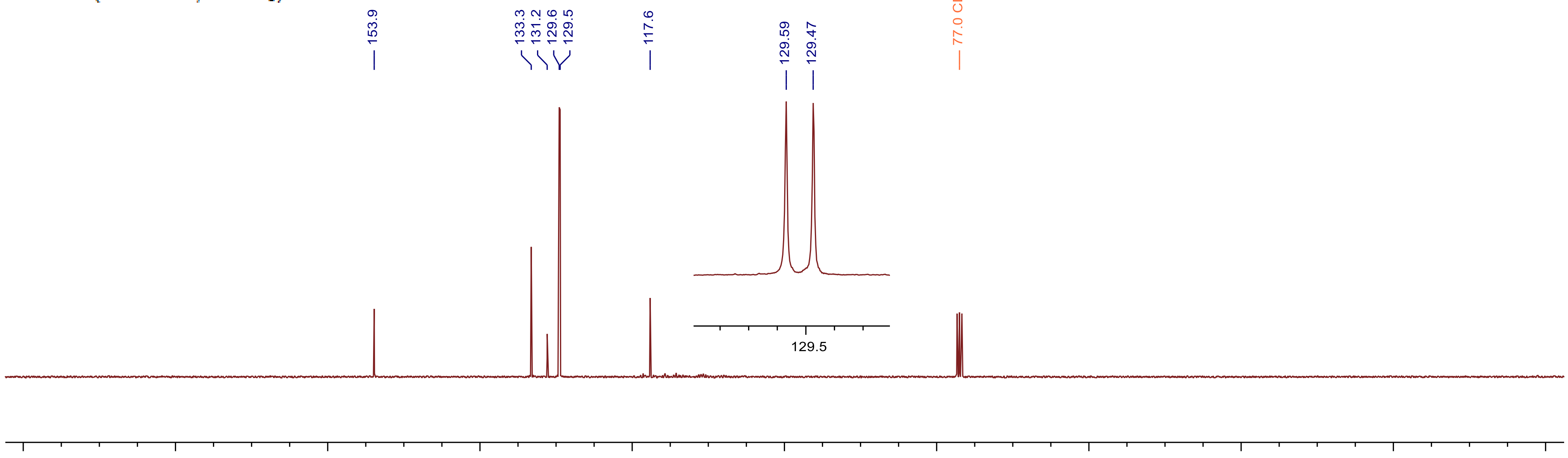

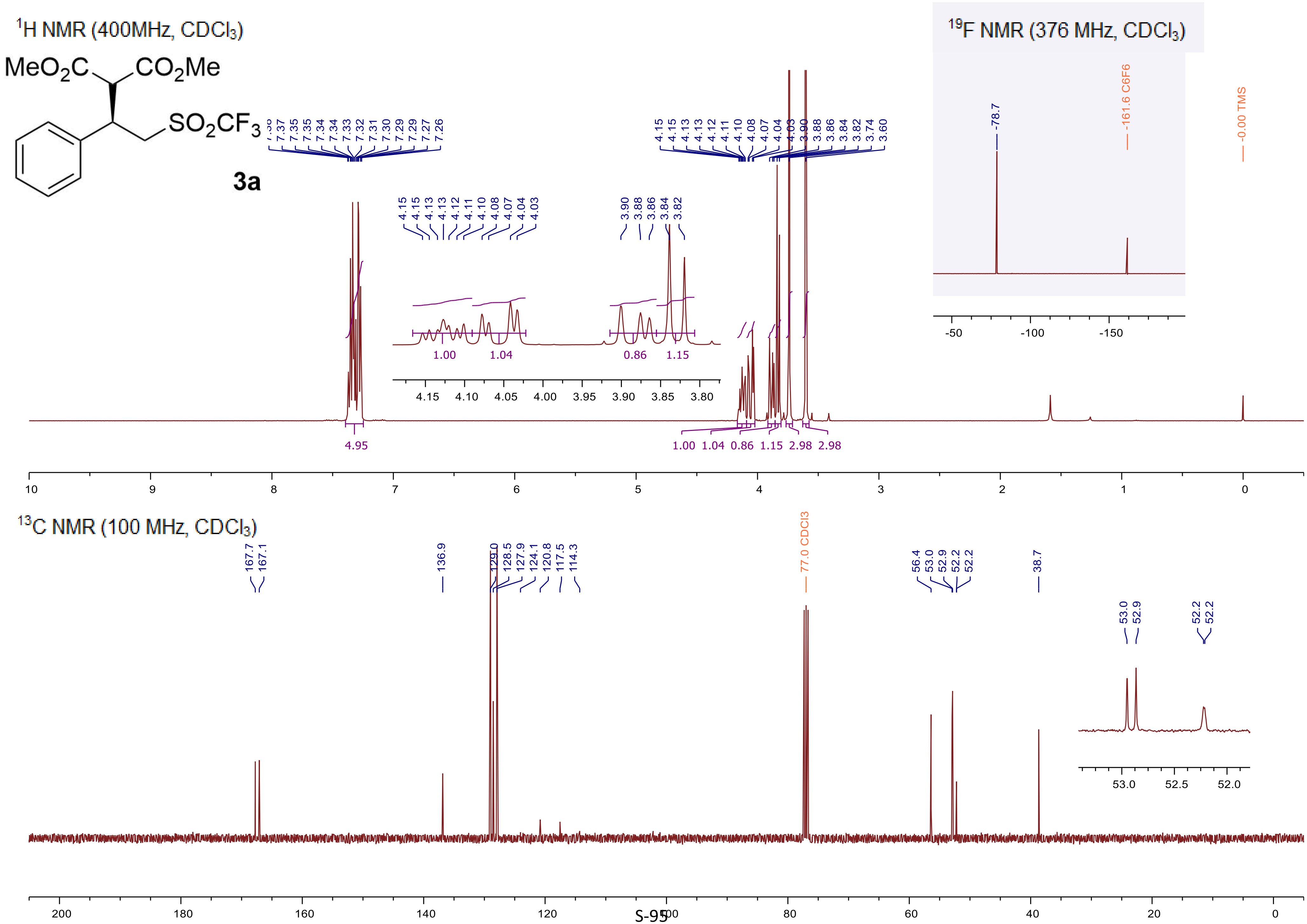

^{1}H NMR (400MHz, CDCl$_3$)
MeO$_2$C CO$_2$Me
SO$_2$CF$_3$
3a
^{19}F NMR (376 MHz, CDCl$_3$)
-78.7
-161.6 C6F6
-0.00 TMS
^{13}C NMR (100 MHz, CDCl$_3$)
167.7 167.1
136.9
129.0 128.5 127.9 124.1 120.8 117.5 114.3
77.0 CDCl3
56.4 53.0 52.9 52.2 52.2
38.7

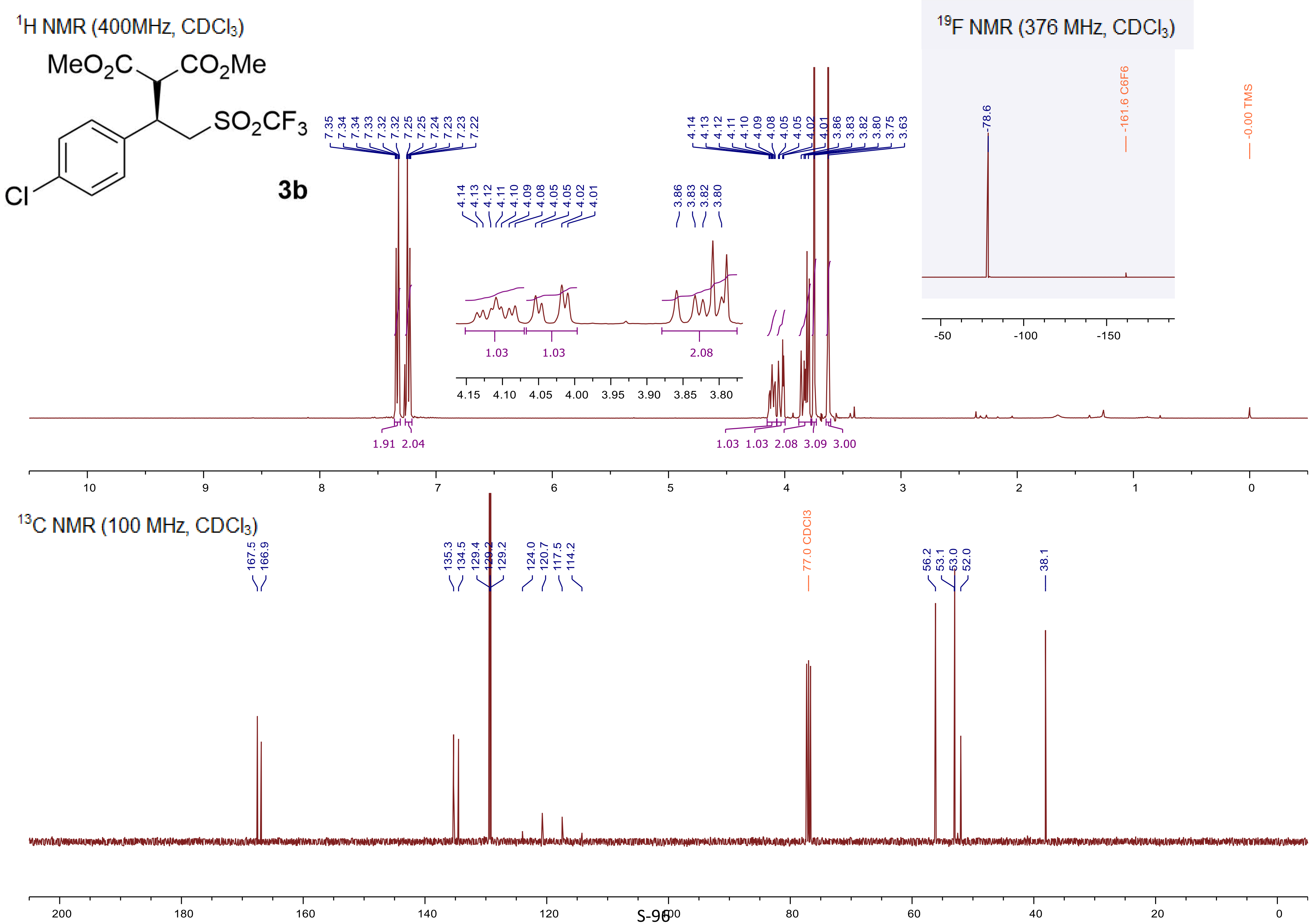

1H NMR (400MHz, CDCl3)
MeO2C
CO2Me
SO2CF3
Cl
3b
7.35 7.34 7.34 7.33 7.32 7.32 7.25 7.25 7.24 7.23 7.23 7.22
4.14 4.13 4.12 4.11 4.10 4.09 4.08 4.05 4.05 4.02 4.01 3.86 3.83 3.82 3.80 3.75 3.63
1.91 2.04
1.03 1.03 2.08 3.09 3.00
19F NMR (376 MHz, CDCl3)
-78.6
-161.6 C6F6
-0.00 TMS
13C NMR (100 MHz, CDCl3)
167.5 166.9
135.3 134.5 129.4 129.2 129.2 124.0 120.7 117.5 114.2
77.0 CDCl3
56.2 53.1 53.0 52.0
38.1

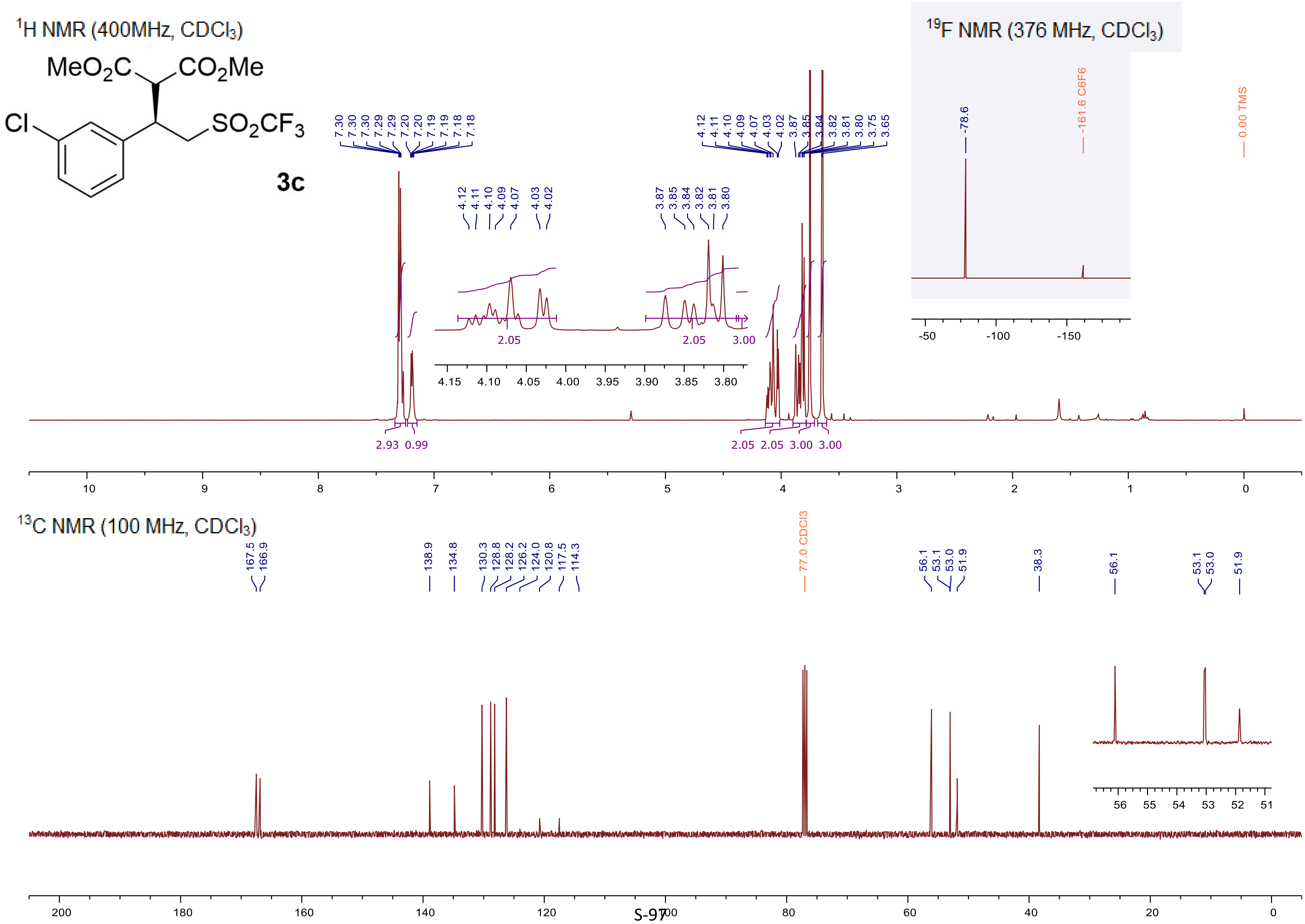

1H NMR (400MHz, CDCl3)
MeO2C CO2Me
Cl
SO2CF3
3c
7.30 7.30 7.30 7.29 7.29 7.20 7.20 7.19 7.19 7.18 7.18
4.12 4.11 4.10 4.09 4.07 4.03 4.02 3.87 3.85 3.84 3.82 3.81 3.80 3.75 3.65
2.93 0.99
2.05 2.05 3.00 3.00
19F NMR (376 MHz, CDCl3)
-78.6
-161.6 C6F6
0.00 TMS
13C NMR (100 MHz, CDCl3)
167.5 166.9
138.9 134.8
130.3 128.8 128.2 126.2 124.0 120.8 117.5 114.3
77.0 CDCl3
56.1 53.1 53.0 51.9
38.3

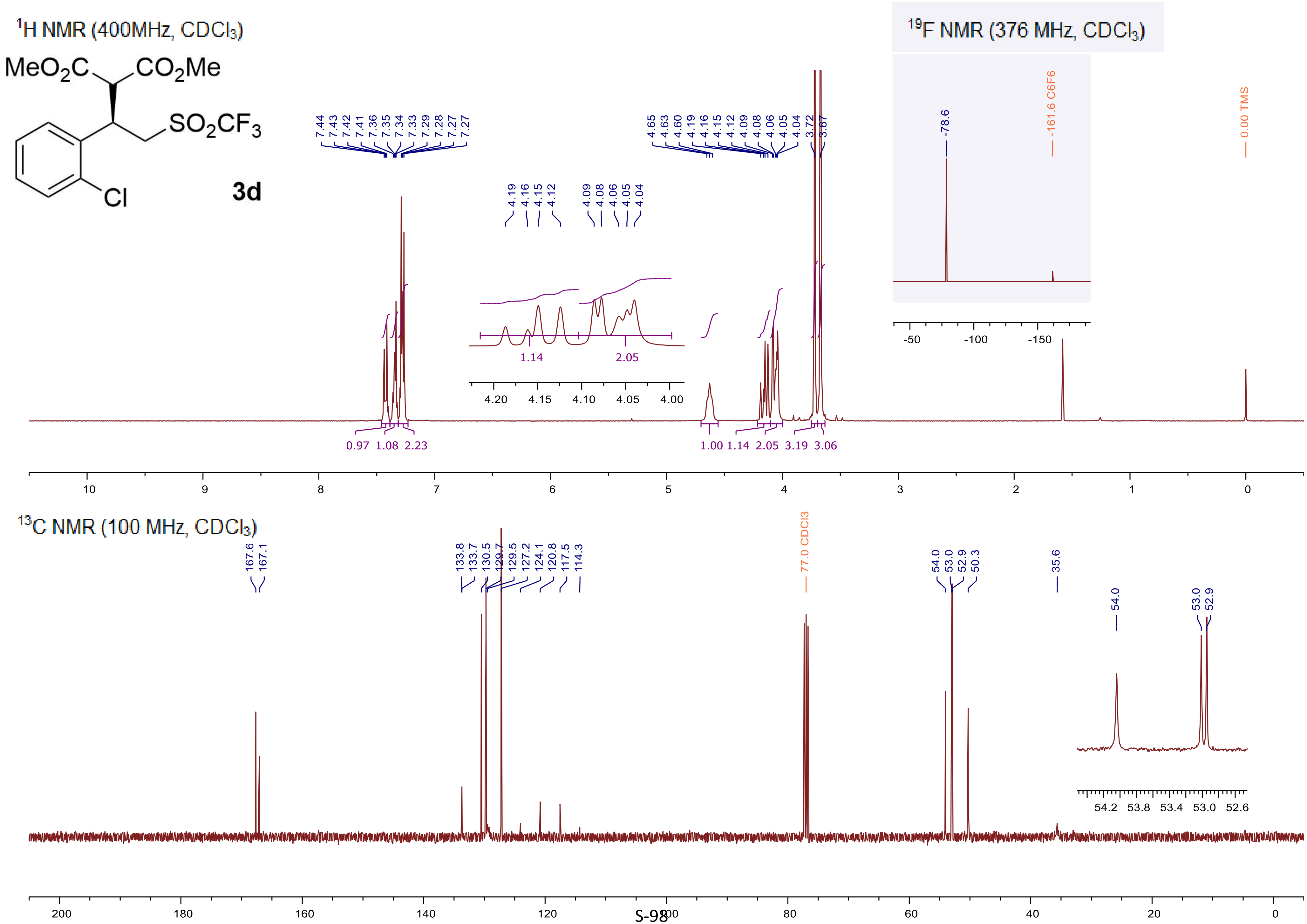

^{1}H NMR (400MHz, CDCl$_3$)
MeO$_2$C
CO$_2$Me
SO$_2$CF$_3$
Cl
3d
7.44 7.43 7.42 7.41 7.36 7.35 7.34 7.33 7.29 7.28 7.27 7.27
4.65 4.63 4.60 4.19 4.16 4.15 4.12 4.09 4.08 4.06 4.05 4.04 3.72 3.67
4.19 4.16 4.15 4.12 4.09 4.08 4.06 4.05 4.04
1.14 2.05
4.20 4.15 4.10 4.05 4.00
0.97 1.08 2.23
1.00 1.14 2.05 3.19 3.06
^{19}F NMR (376 MHz, CDCl$_3$)
-78.6
-161.6 C6F6
0.00 TMS
-50 -100 -150
10 9 8 7 6 5 4 3 2 1 0
^{13}C NMR (100 MHz, CDCl$_3$)
167.6 167.1
133.8 133.7 130.5 129.7 129.5 127.2 124.1 120.8 117.5 114.3
77.0 CDCl3
54.0 53.0 52.9 50.3
35.6
54.0
53.0 52.9
54.2 53.8 53.4 53.0 52.6
200 180 160 140 120 100 80 60 40 20 0

^{1}H NMR (400MHz, $CDCl_3$)

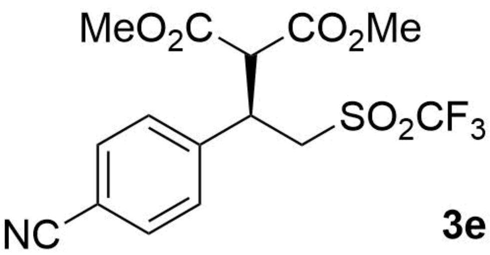

3e

^{19}F NMR (376 MHz, $CDCl_3$)

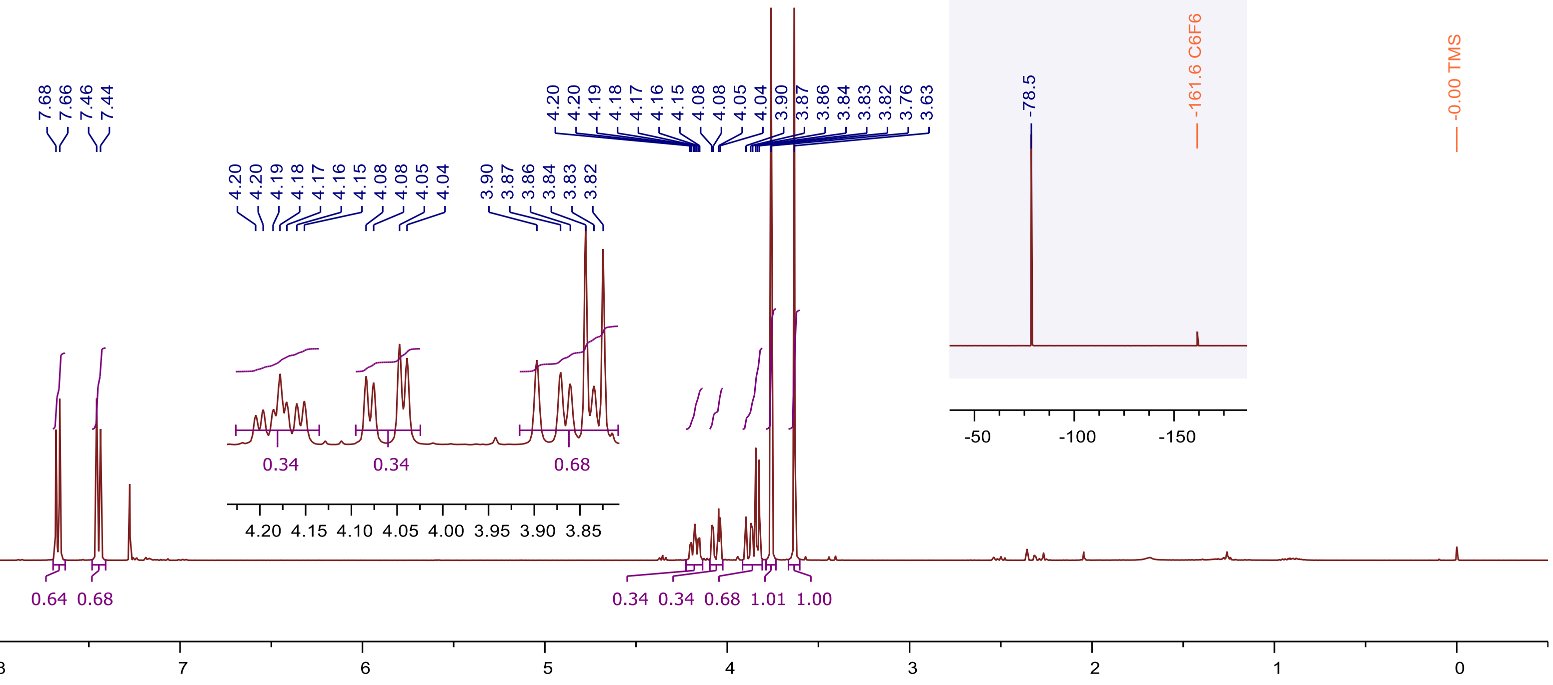

^{13}C NMR (100 MHz, $CDCl_3$)

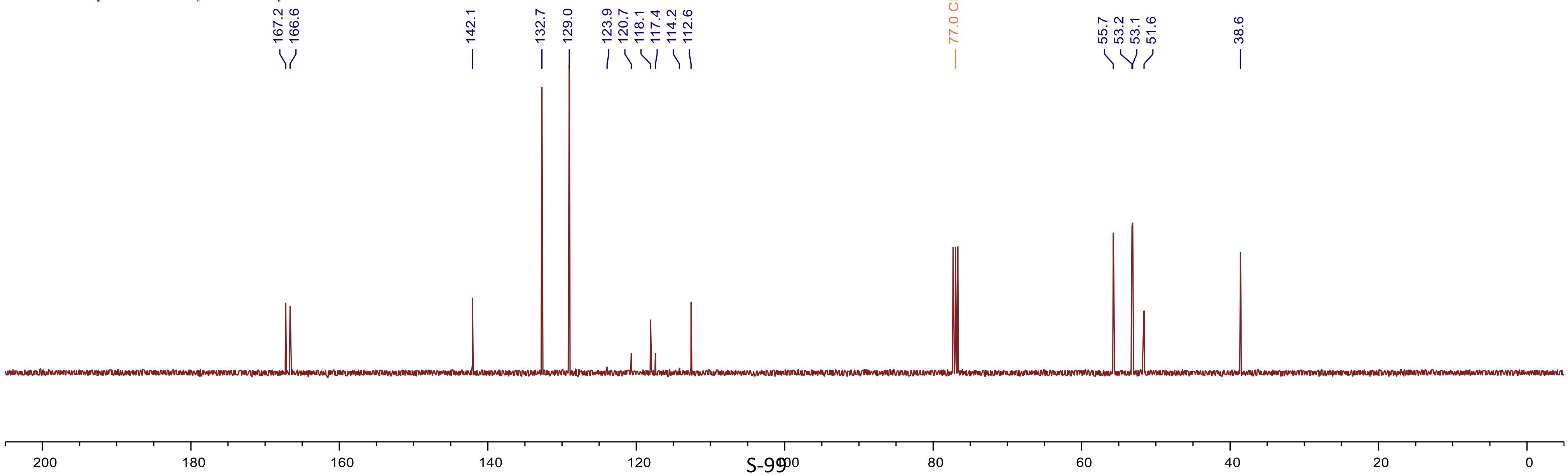

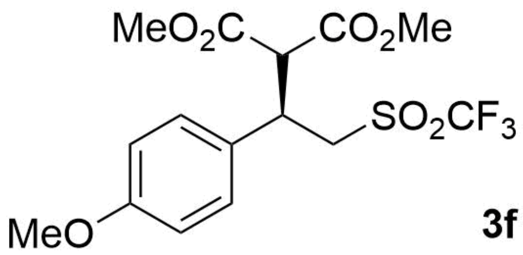
MeO₂C
CO₂Me
SO₂CF₃
MeO

3f

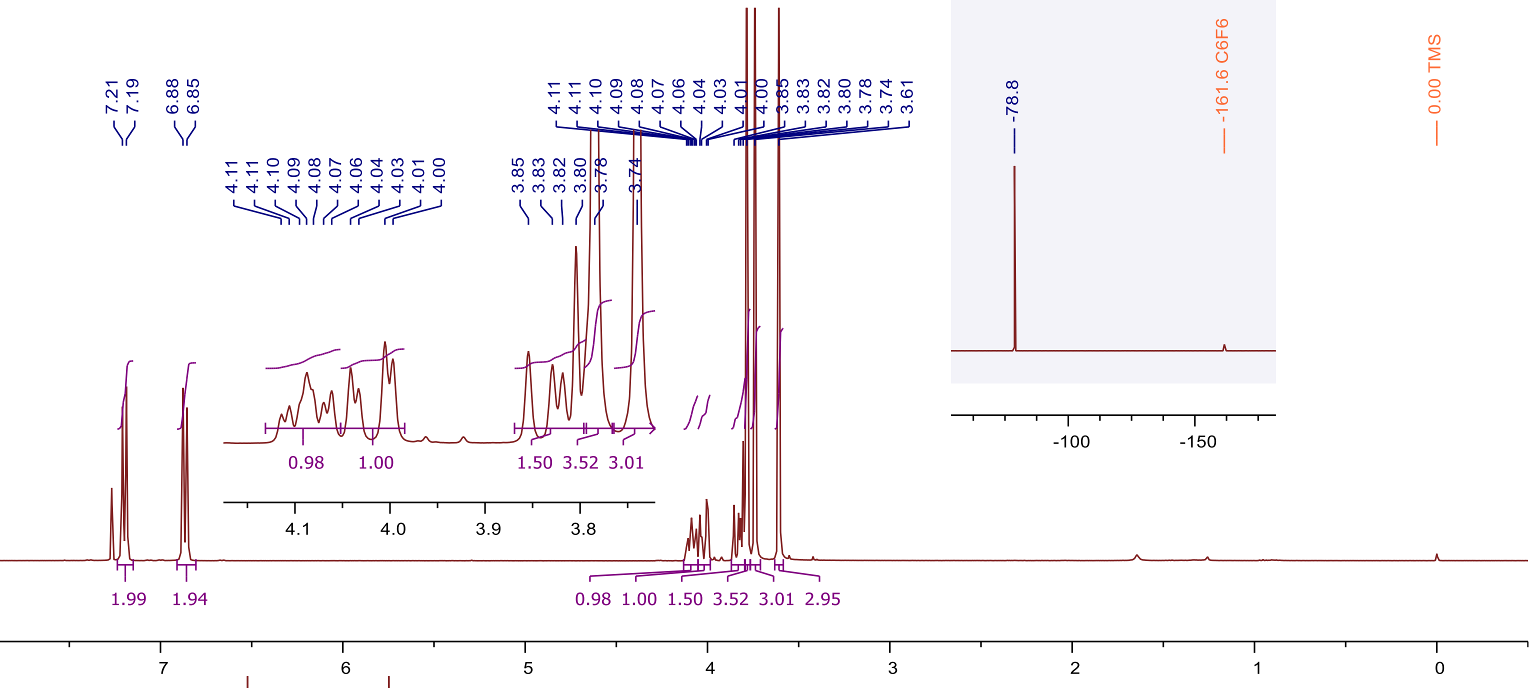
1H NMR (400MHz, CDCl3)
7.21 7.19 6.88 6.85
4.11 4.11 4.10 4.09 4.08 4.07 4.06 4.04 4.03 4.01 4.00 3.85 3.83 3.82 3.80 3.78 3.74 3.61
1.99 1.94 0.98 1.00 1.50 3.52 3.01 2.95
19F NMR (376 MHz, CDCl3)
-78.8
-161.6 C6F6
0.00 TMS

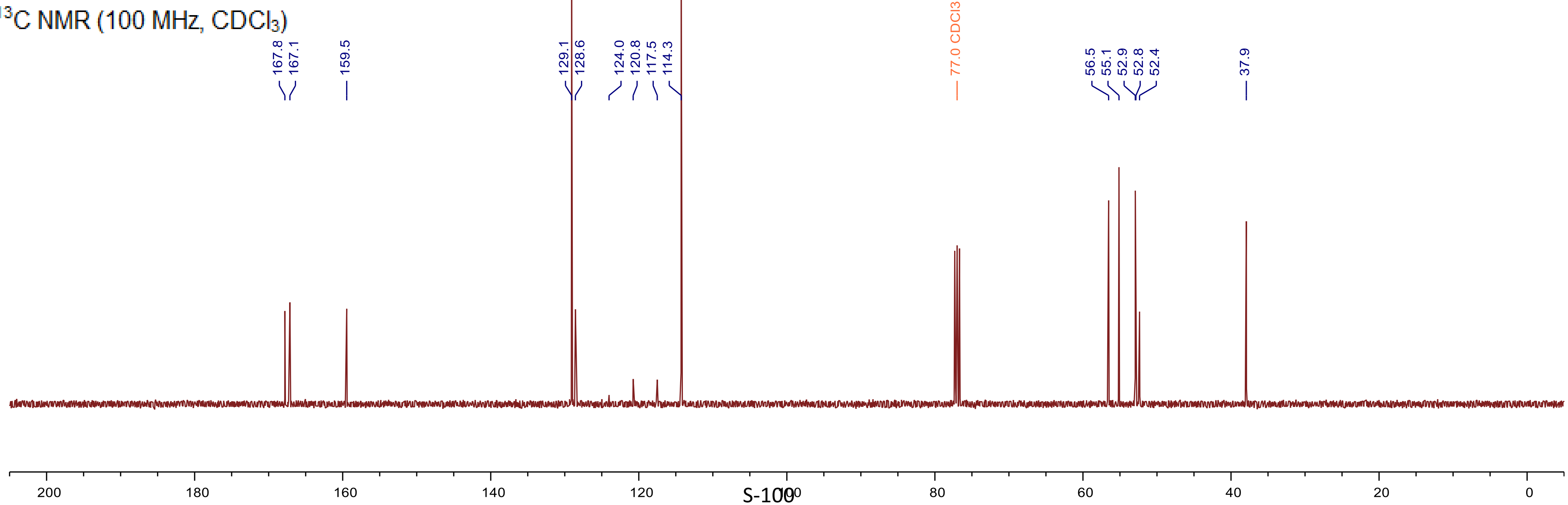
13C NMR (100 MHz, CDCl3)
167.8 167.1 159.5 129.1 128.6 124.0 120.8 117.5 114.3
77.0 CDCl3
56.5 55.1 52.9 52.8 52.4 37.9

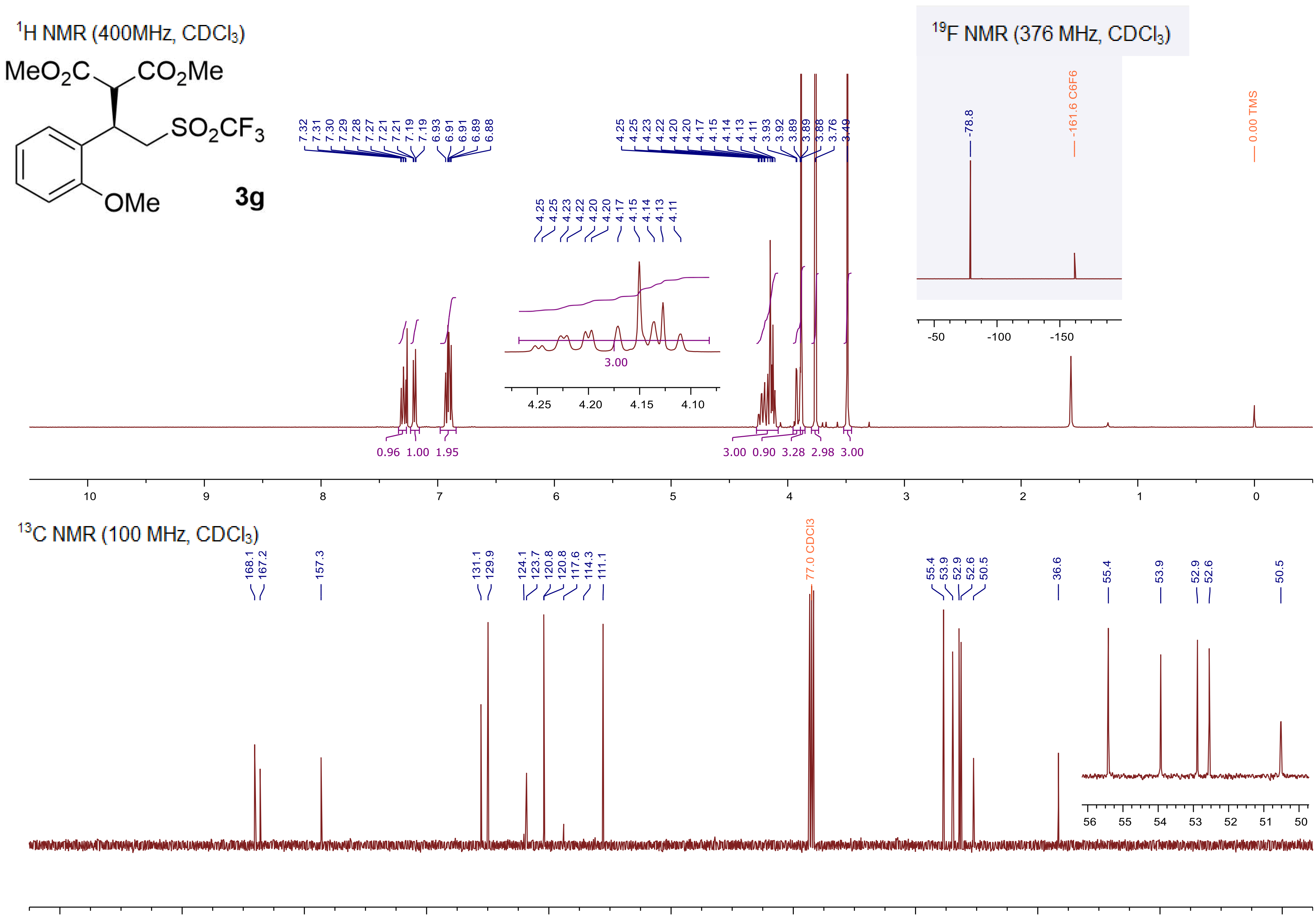

^{1}H NMR (400MHz, $CDCl_3$)
MeO_2C
CO_2Me
SO_2CF_3
OMe
3g
^{19}F NMR (376 MHz, $CDCl_3$)
-78.8
-161.6 C6F6
0.00 TMS
7.32 7.31 7.30 7.29 7.28 7.27 7.21 7.21 7.19 7.19 6.93 6.91 6.91 6.89 6.88
4.25 4.25 4.23 4.22 4.20 4.20 4.17 4.15 4.14 4.13 4.11 3.93 3.92 3.89 3.89 3.88 3.76 3.49
0.96 1.00 1.95
3.00 0.90 3.28 2.98 3.00
3.00
^{13}C NMR (100 MHz, $CDCl_3$)
168.1 167.2 157.3 131.1 129.9 124.1 123.7 120.8 120.8 117.6 114.3 111.1
77.0 CDCl3
55.4 53.9 52.9 52.6 50.5 36.6

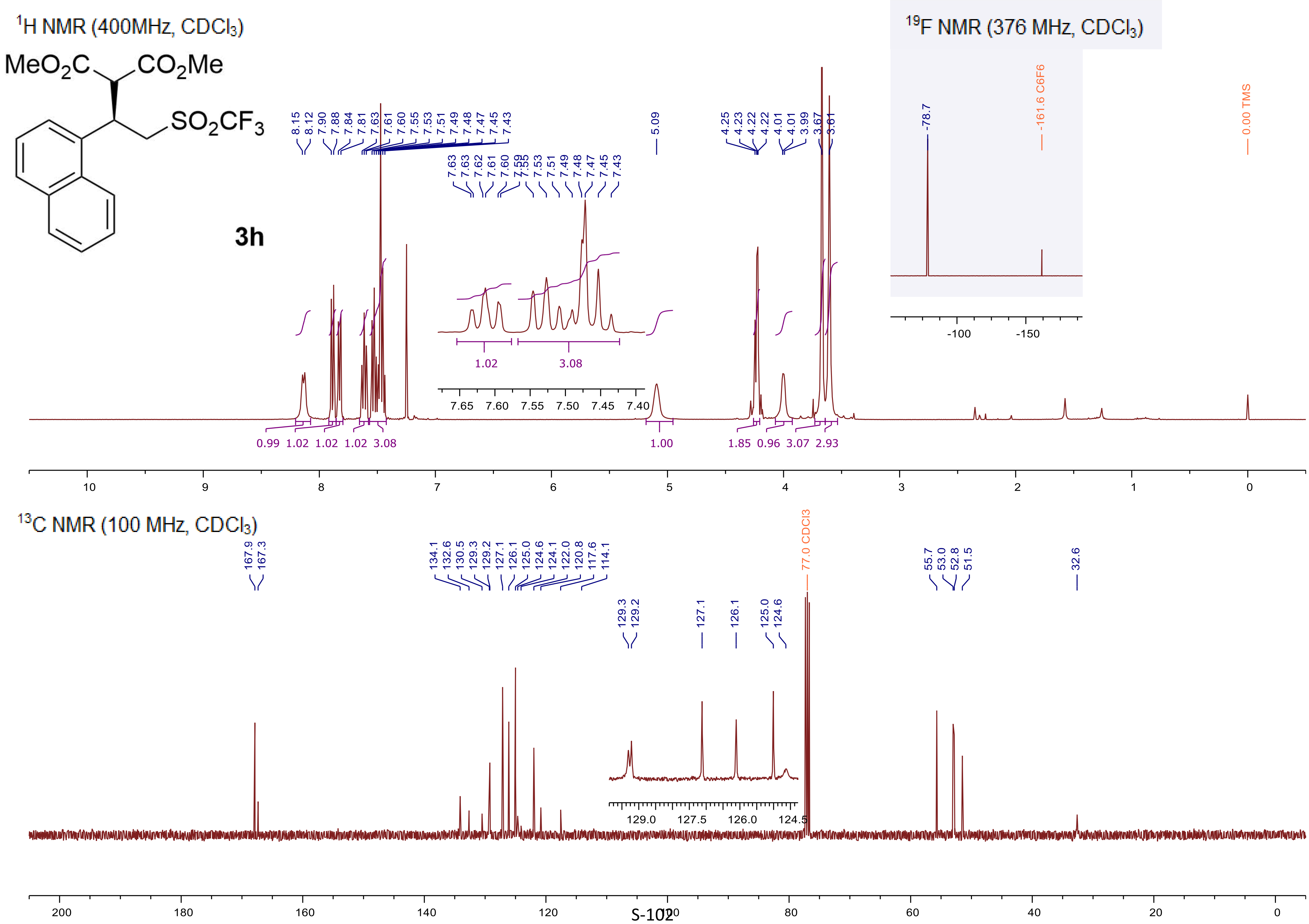

^{1}H NMR (400MHz, CDCl$_3$)
MeO$_2$C CO$_2$Me
SO$_2$CF$_3$
3h
^{19}F NMR (376 MHz, CDCl$_3$)
-78.7
-161.6 C6F6
0.00 TMS
-100
-150
8.15 8.12 7.90 7.88 7.84 7.81 7.63 7.61 7.60 7.55 7.53 7.51 7.49 7.48 7.47 7.45 7.43
5.09
4.25 4.23 4.22 4.22 4.01 4.01 3.99 3.67 3.61
7.63 7.63 7.62 7.61 7.60 7.59 7.55 7.53 7.51 7.49 7.48 7.47 7.45 7.43
1.02 3.08
7.65 7.60 7.55 7.50 7.45 7.40
0.99 1.02 1.02 1.02 3.08
1.00
1.85 0.96 3.07 2.93
10 9 8 7 6 5 4 3 2 1 0
^{13}C NMR (100 MHz, CDCl$_3$)
167.9 167.3
134.1 132.6 130.5 129.3 129.2 127.1 126.1 125.0 124.6 124.1 122.0 120.8 117.6 114.1
77.0 CDCl3
55.7 53.0 52.8 51.5
32.6
129.3 129.2
127.1
126.1
125.0 124.6
129.0 127.5 126.0 124.5
200 180 160 140 120 100 80 60 40 20 0

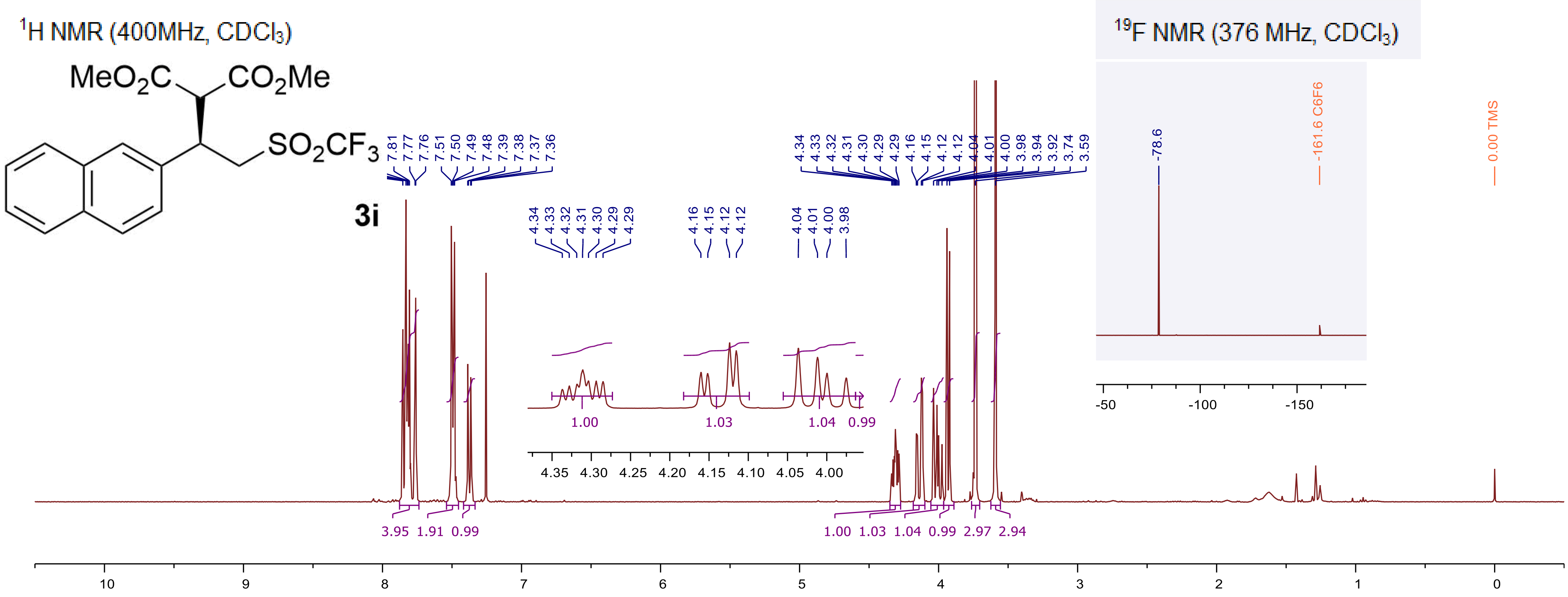

1H NMR (400MHz, CDCl3)
MeO2C
CO2Me
SO2CF3
3i
7.81 7.77 7.76 7.51 7.50 7.49 7.48 7.39 7.38 7.37 7.36
4.34 4.33 4.32 4.31 4.30 4.29 4.29 4.16 4.15 4.12 4.12 4.04 4.01 4.00 3.98 3.94 3.92 3.74 3.59
1.00 1.03 1.04 0.99
4.35 4.30 4.25 4.20 4.15 4.10 4.05 4.00
3.95 1.91 0.99
1.00 1.03 1.04 0.99 2.97 2.94
19F NMR (376 MHz, CDCl3)
-78.6
-161.6 C6F6
0.00 TMS
-50 -100 -150
10 9 8 7 6 5 4 3 2 1 0

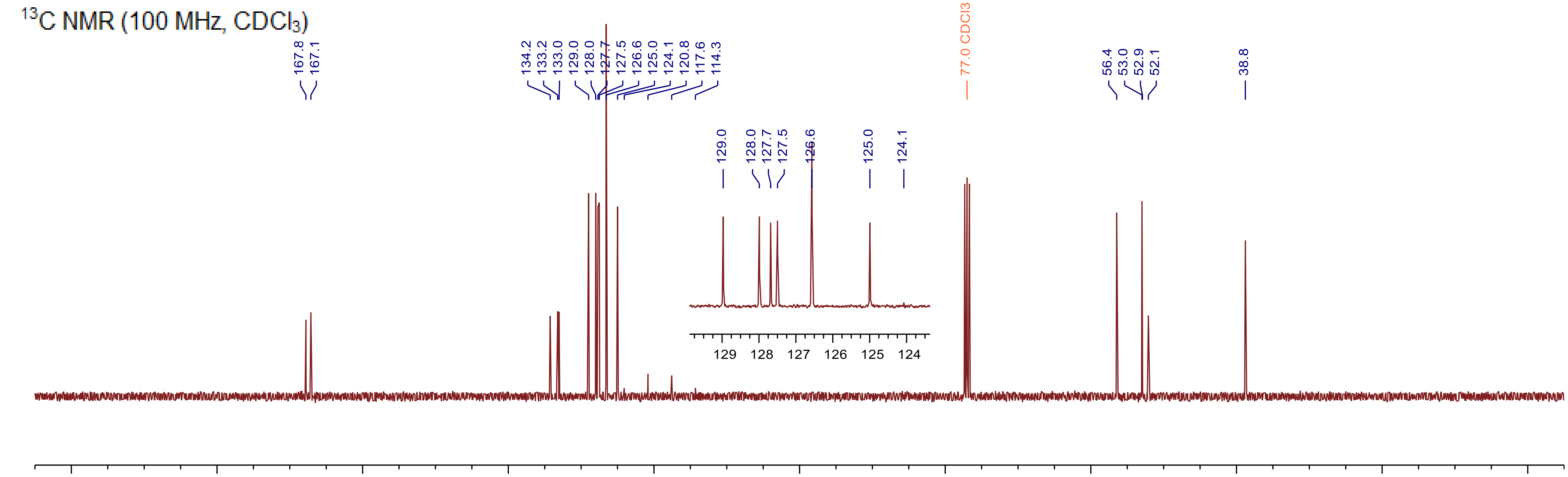

13C NMR (100 MHz, CDCl3)
167.8 167.1
134.2 133.2 133.0 129.0 128.0 127.7 127.5 126.6 125.0 124.1 120.8 117.6 114.3
77.0 CDCl3
56.4 53.0 52.9 52.1
38.8
129.0 128.0 127.7 127.5 126.6 125.0 124.1
129 128 127 126 125 124
200 180 160 140 120 100 80 60 40 20 0

^{1}H NMR (400MHz, $CDCl_3$)

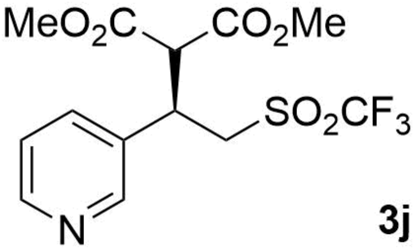

3j

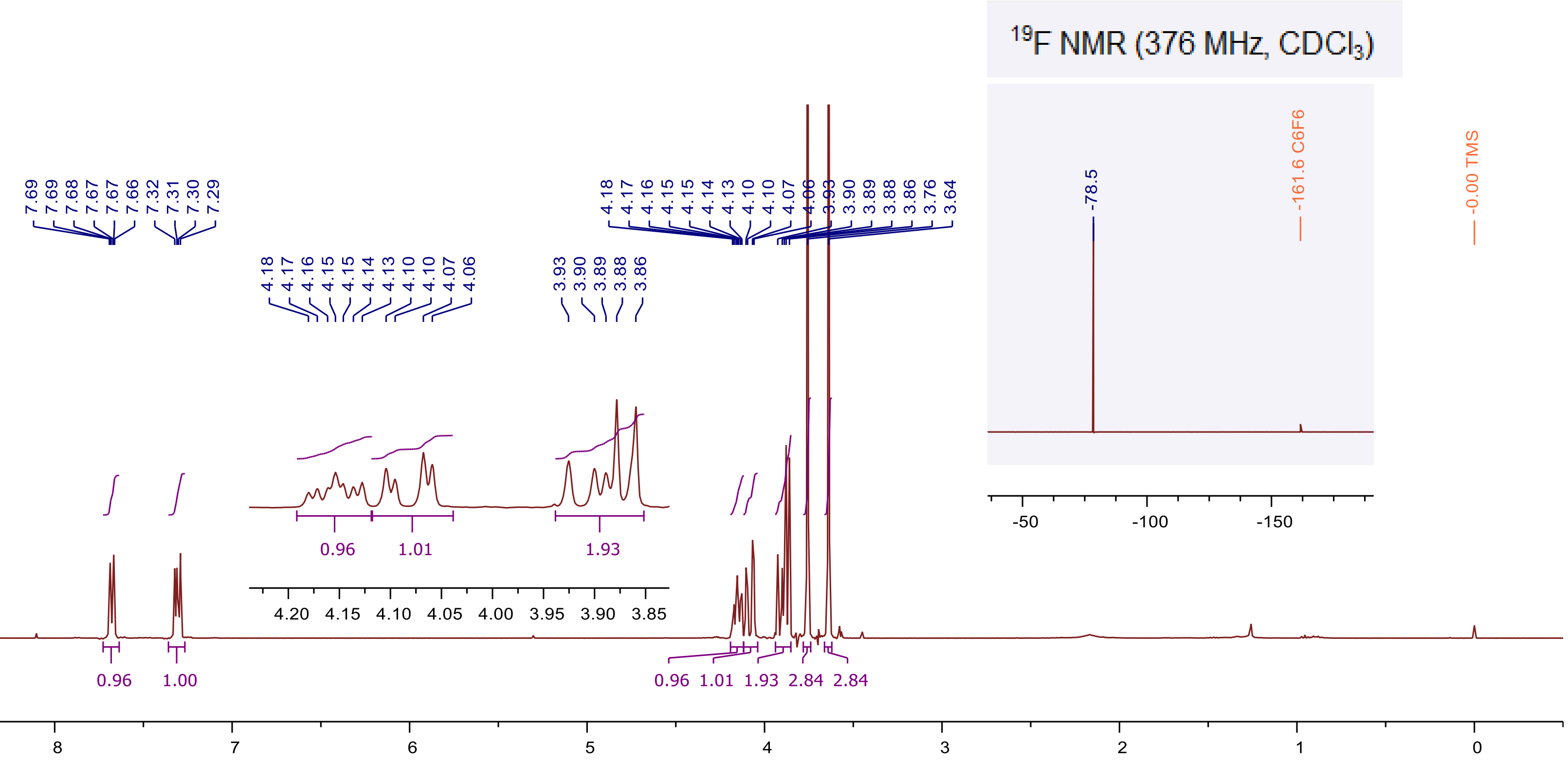

^{13}C NMR (100 MHz, $CDCl_3$)

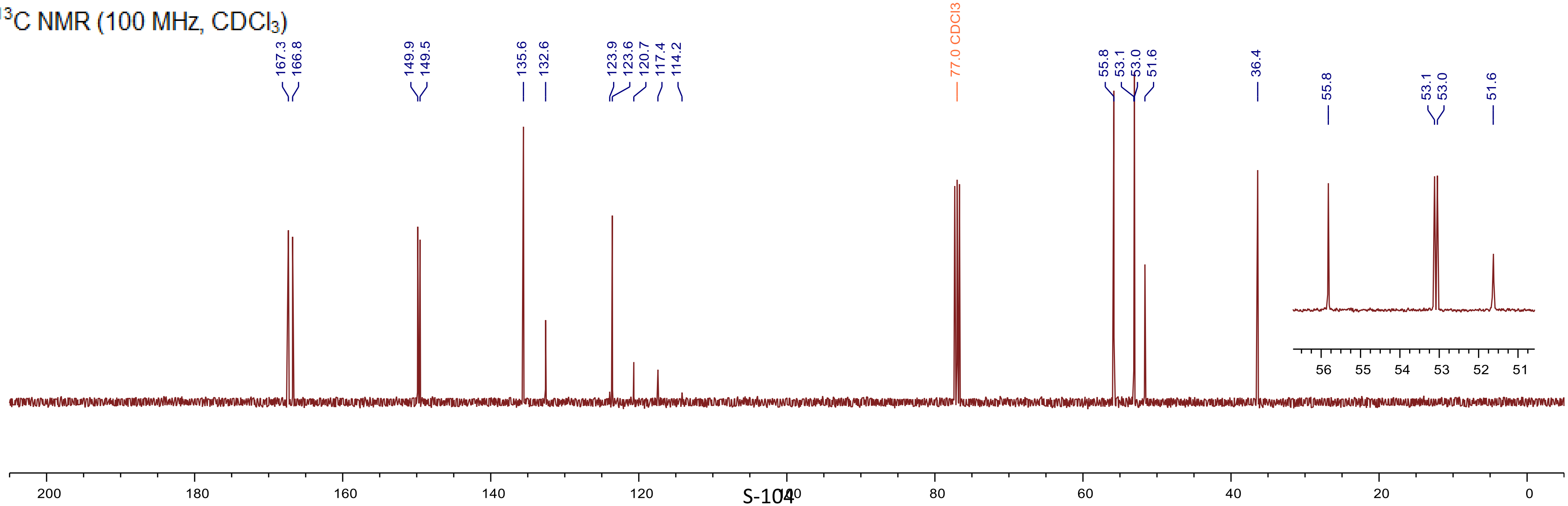

^{1}H NMR (400MHz, $CDCl_3$)

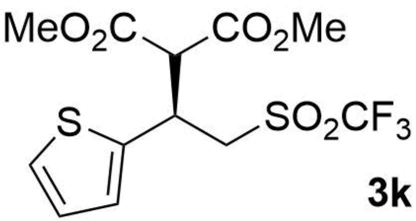

3k

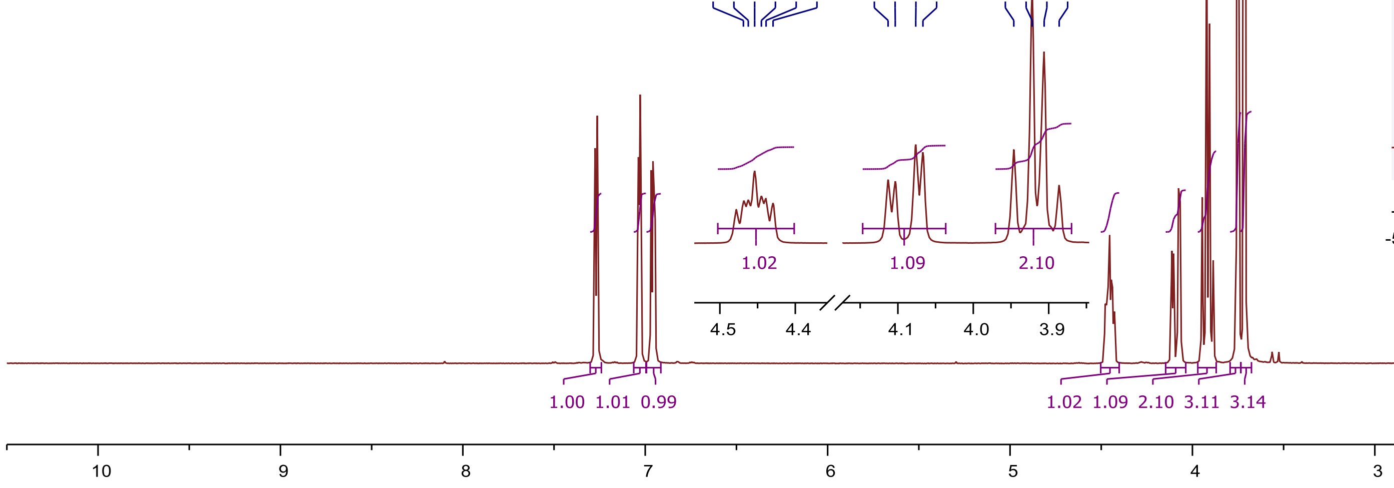

^{19}F NMR (376 MHz, $CDCl_3$)

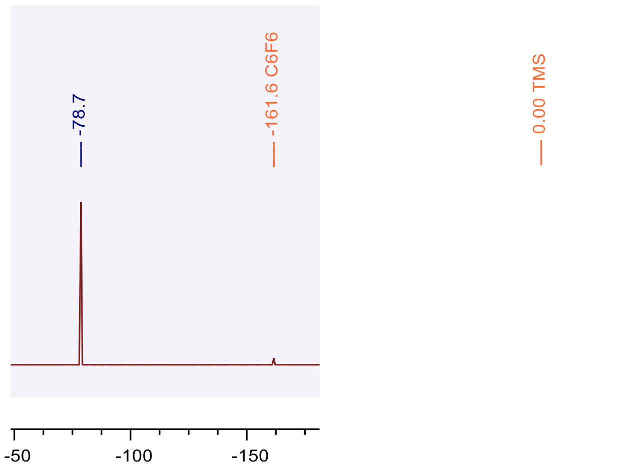

^{13}C NMR (100 MHz, $CDCl_3$)

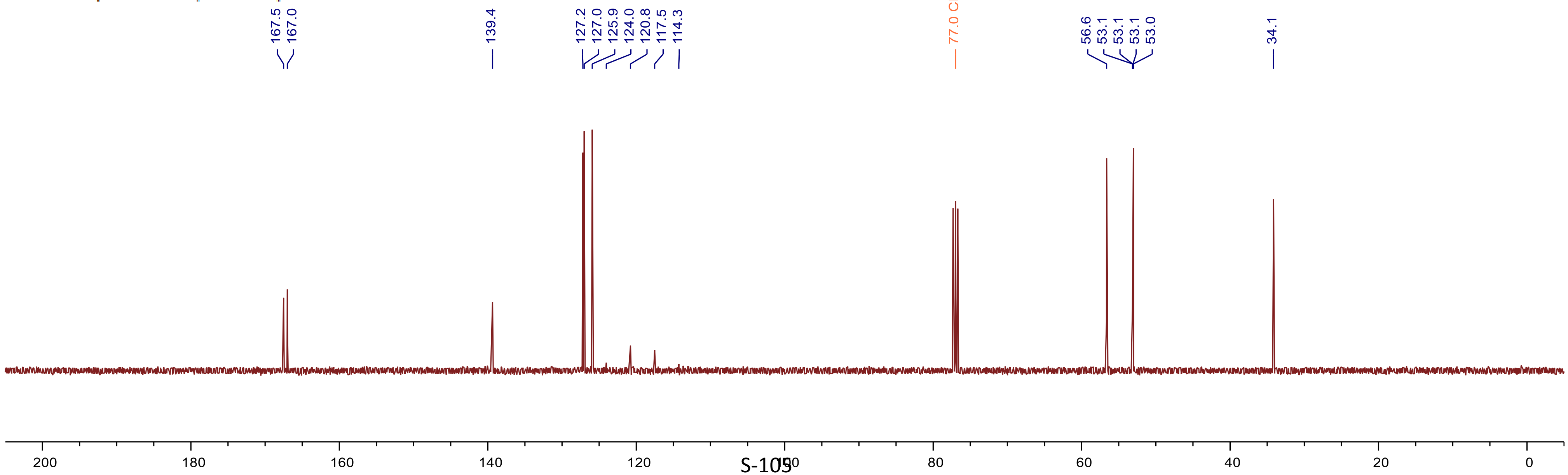

^{1}H NMR (400MHz, $CDCl_3$)

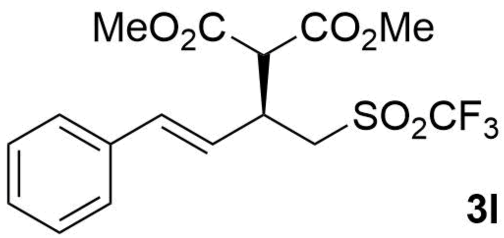

3l

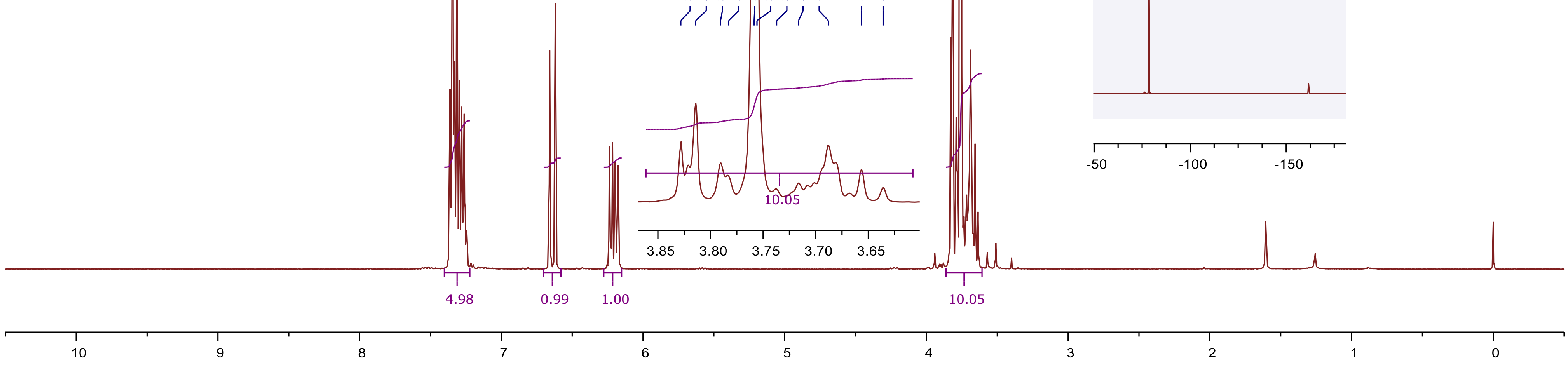

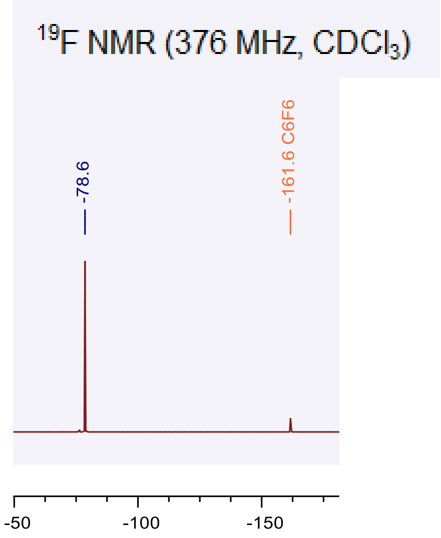

^{13}C NMR (100 MHz, $CDCl_3$)

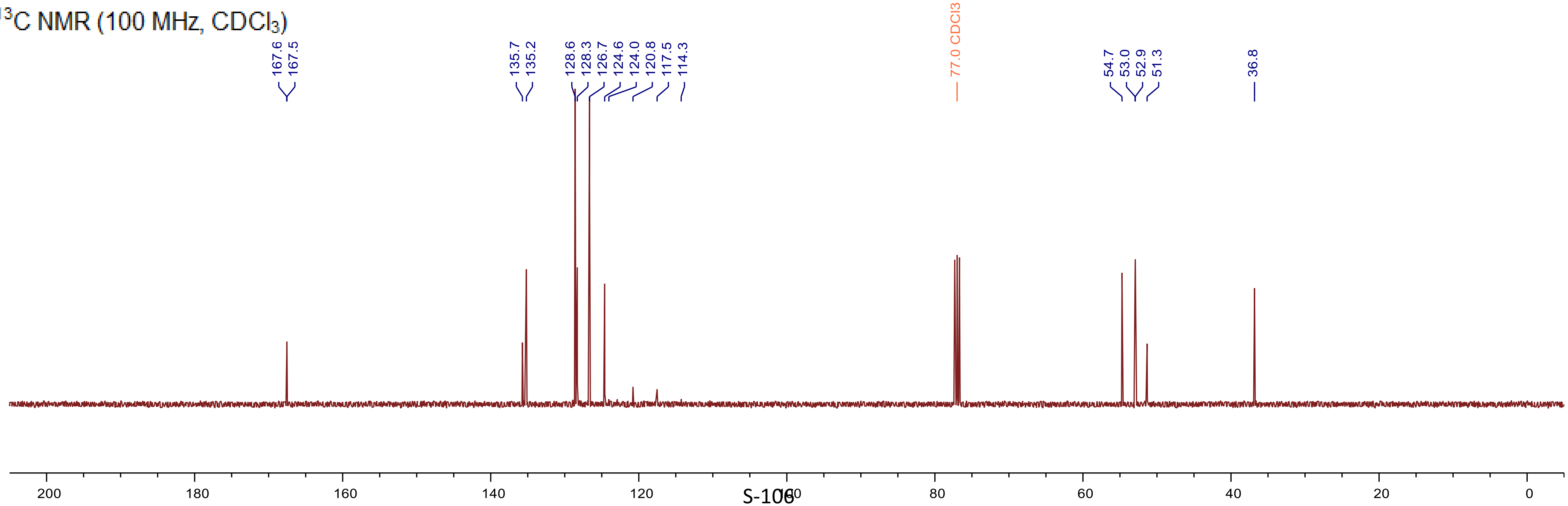

^{1}H NMR (400MHz, $CDCl_3$)

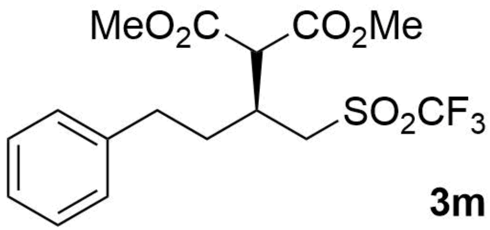

3m

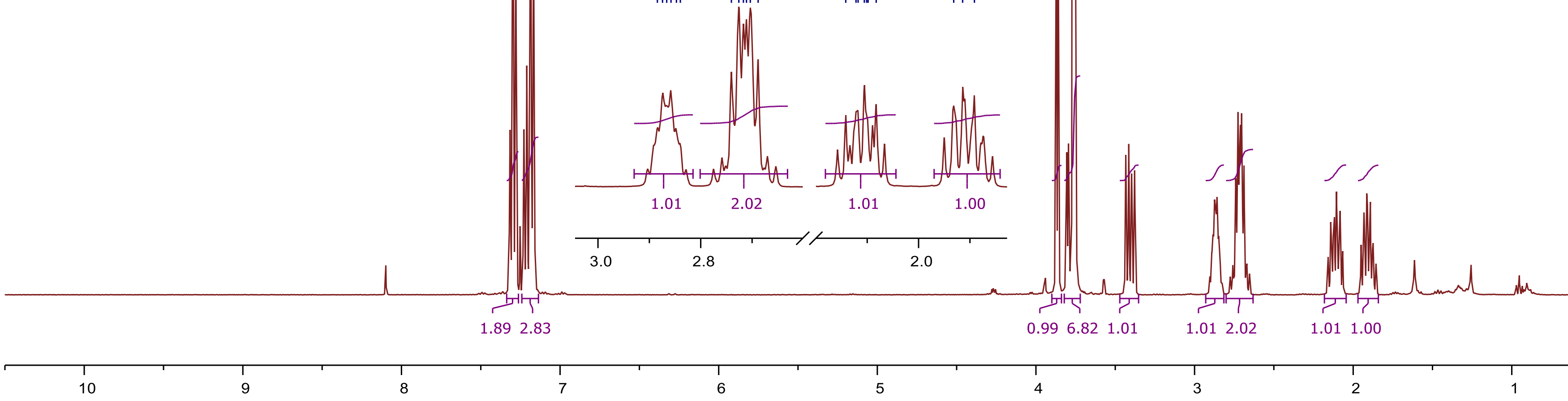

^{19}F NMR (376 MHz, $CDCl_3$)

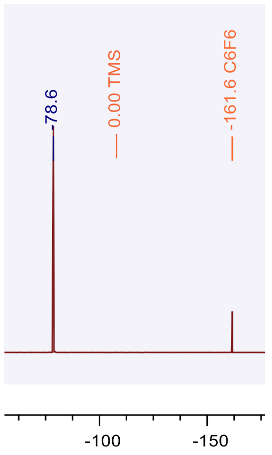

^{13}C NMR (100 MHz, $CDCl_3$)

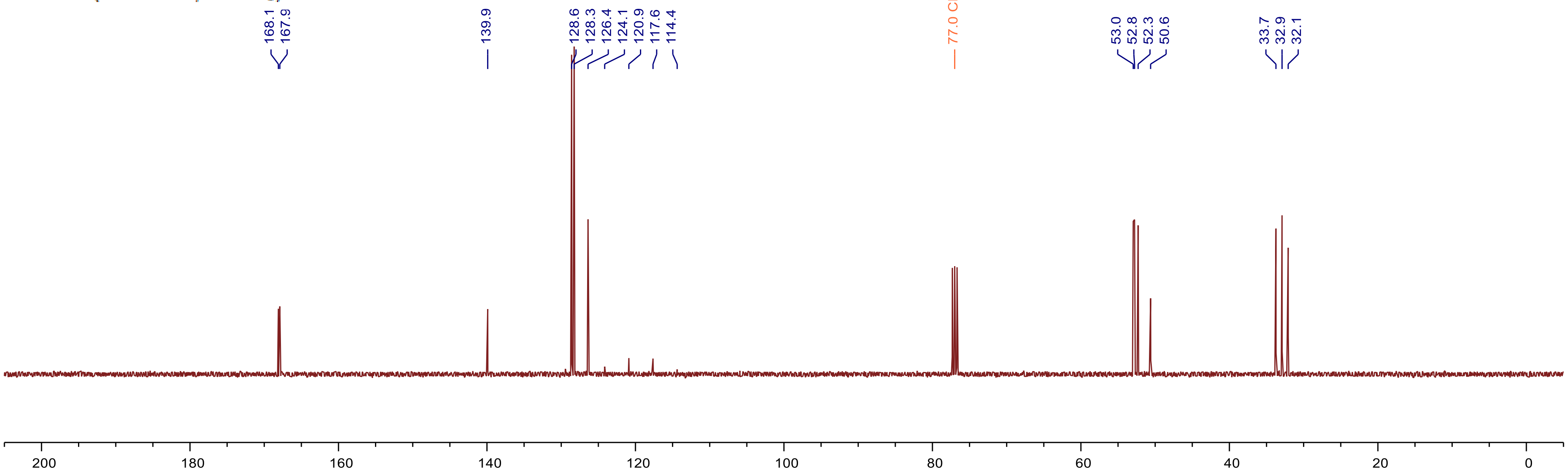

^{1}H NMR (400MHz, $CDCl_3$)

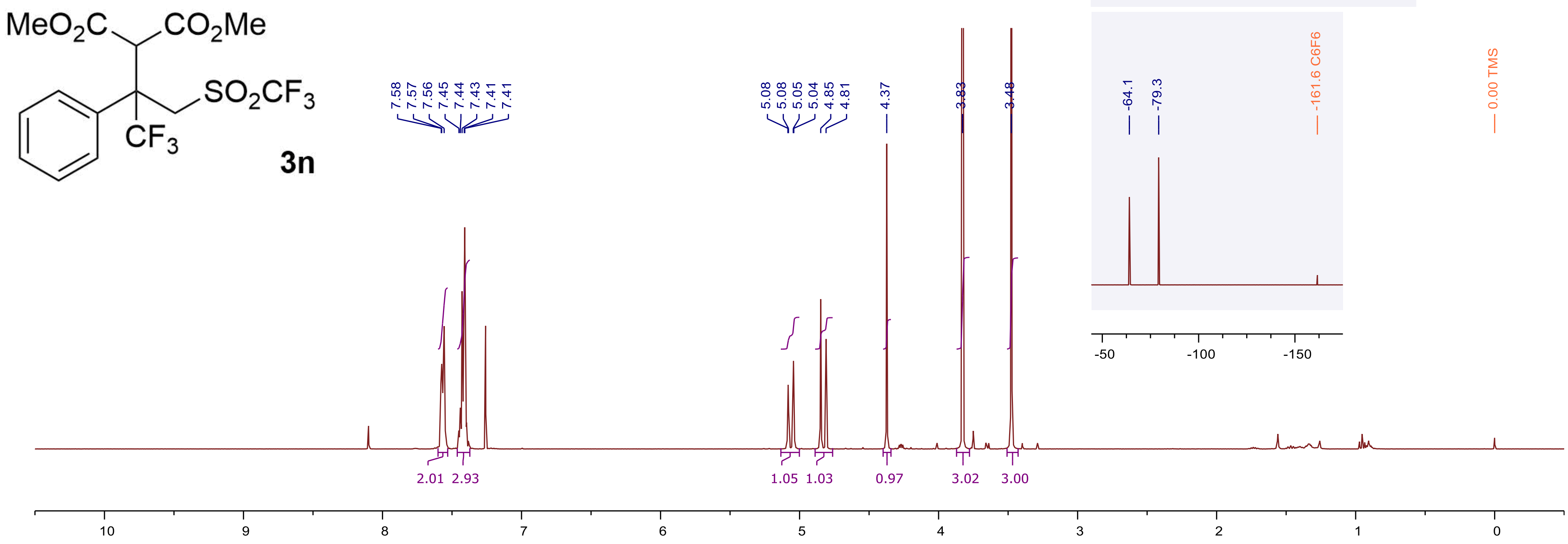

^{13}C NMR (100 MHz, $CDCl_3$)

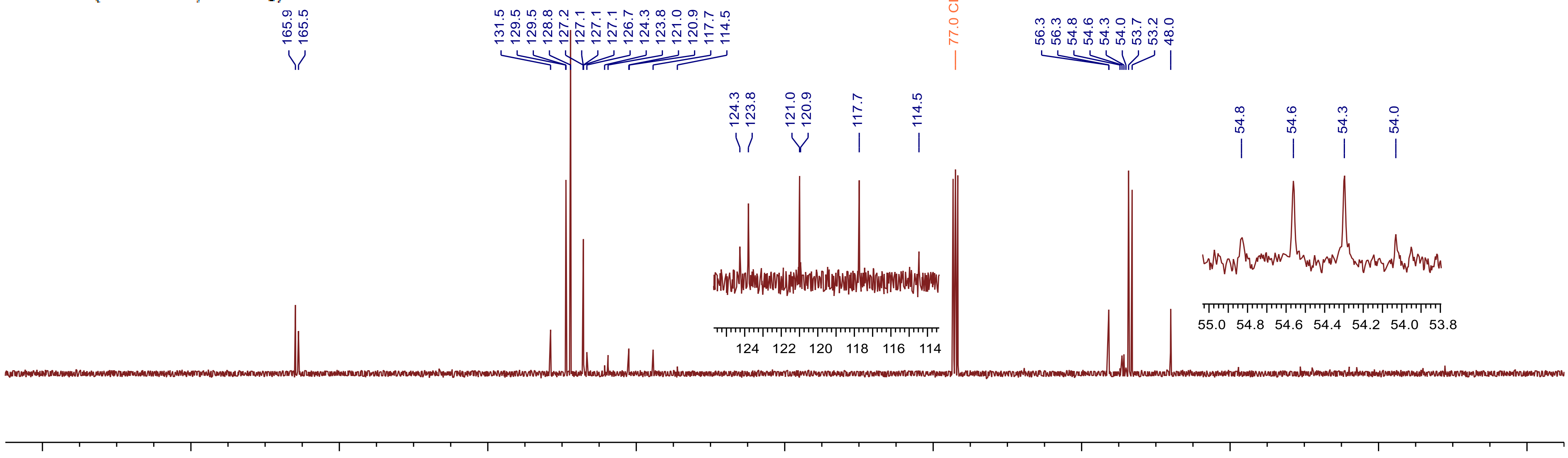

^{1}H NMR (400MHz, $CDCl_3$)

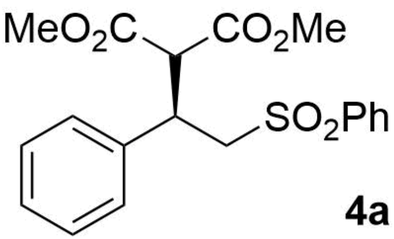

4a

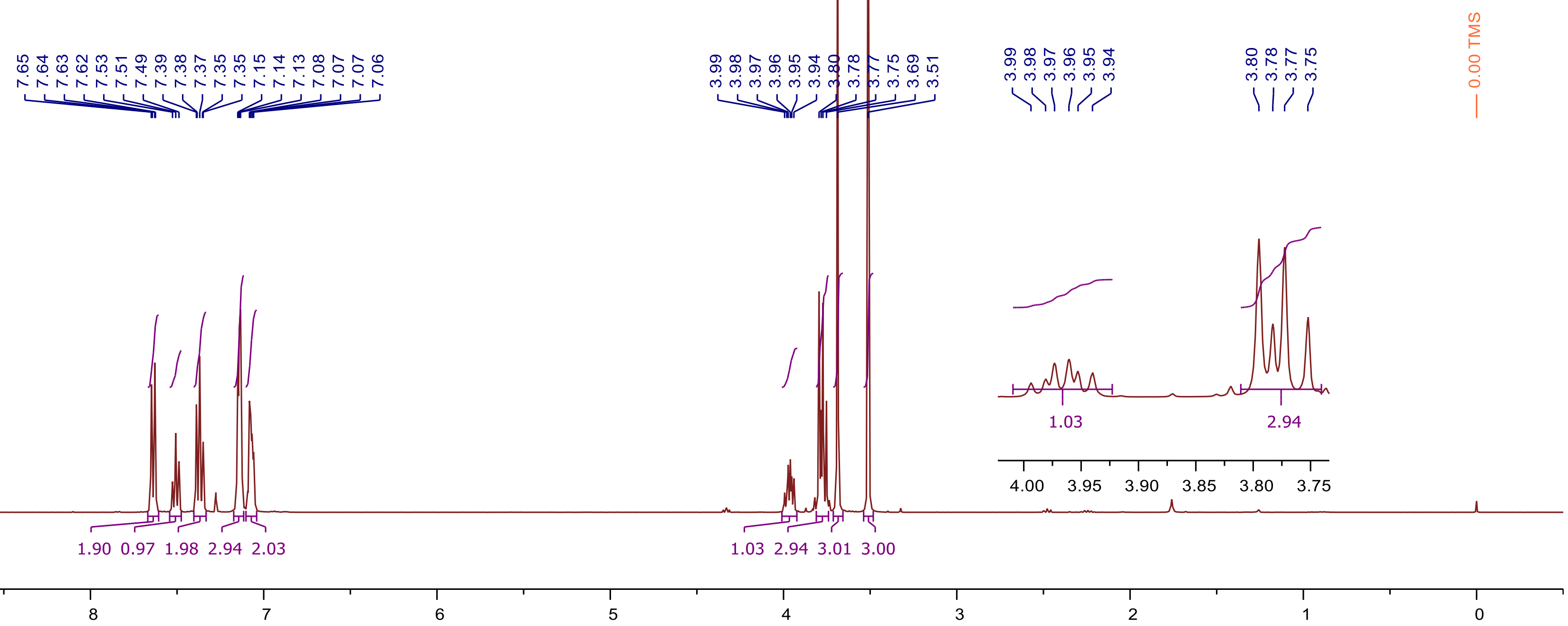

^{13}C NMR (100 MHz, $CDCl_3$)

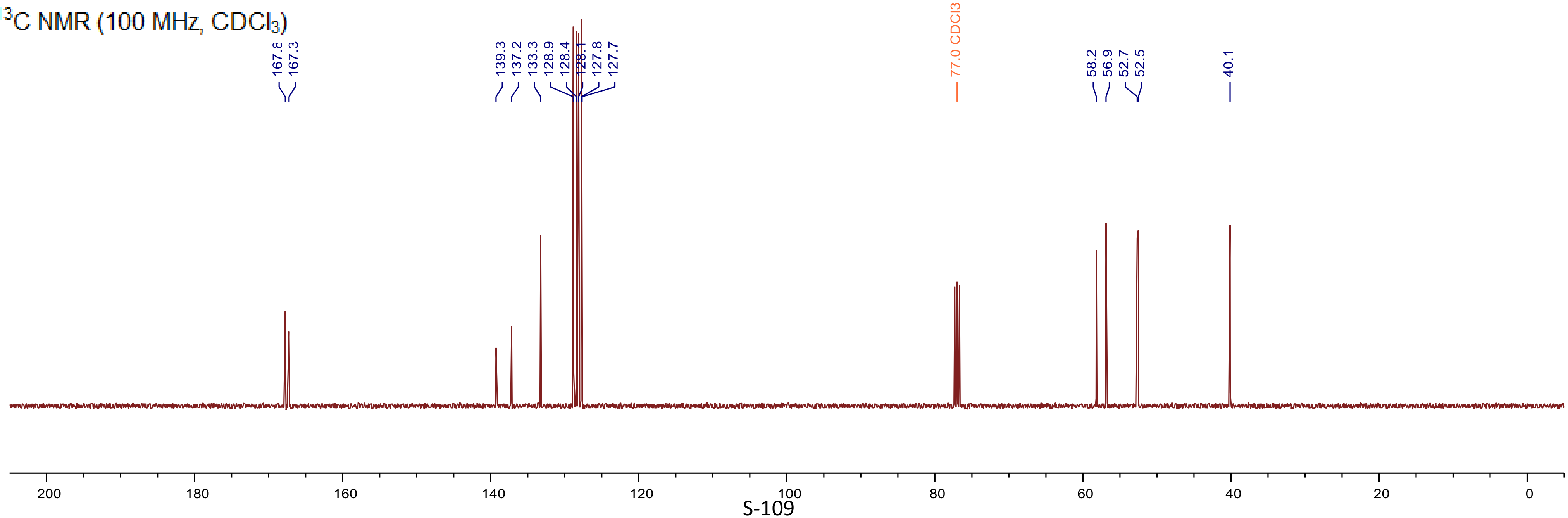

^{1}H NMR (400MHz, $CDCl_3$)

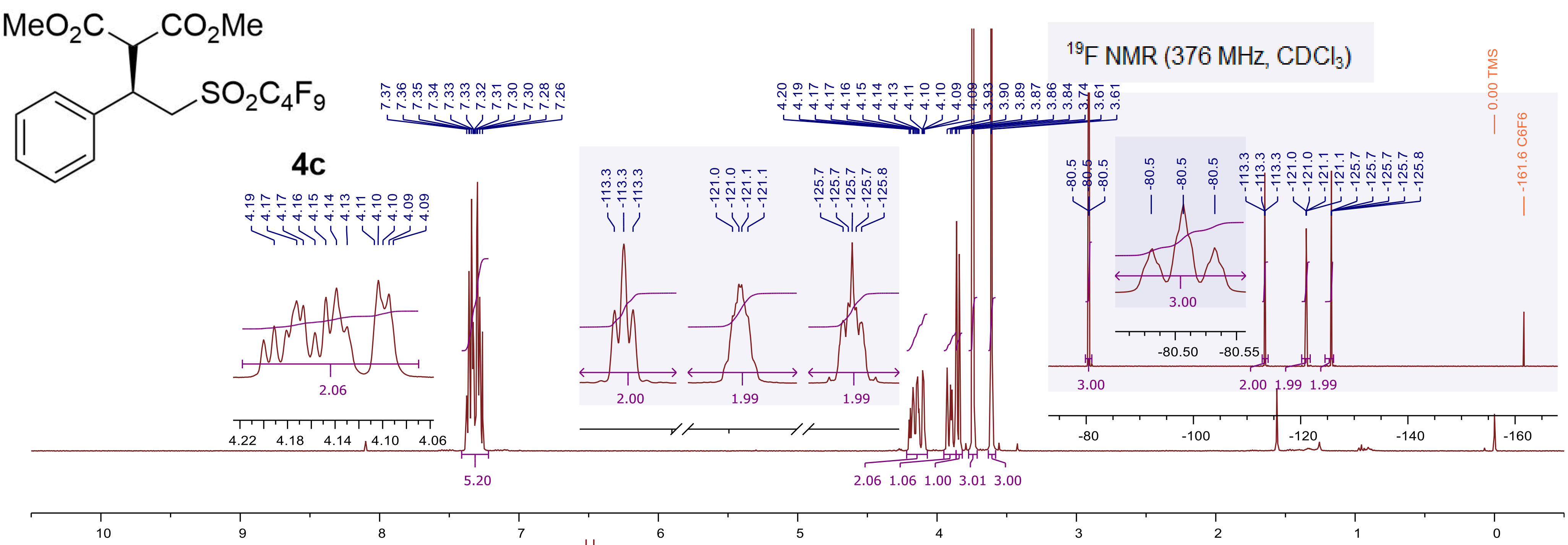

^{13}C NMR (100 MHz, $CDCl_3$)

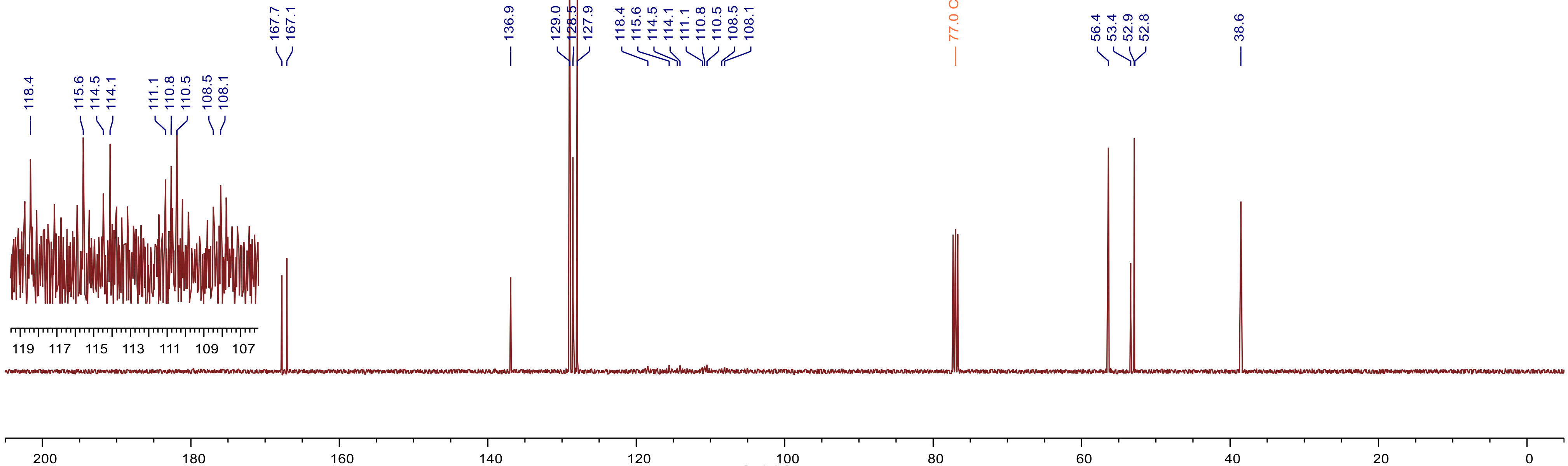

^{1}H NMR (400MHz, $CDCl_3$)

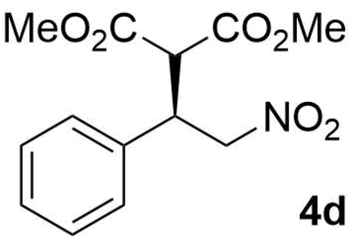

4d

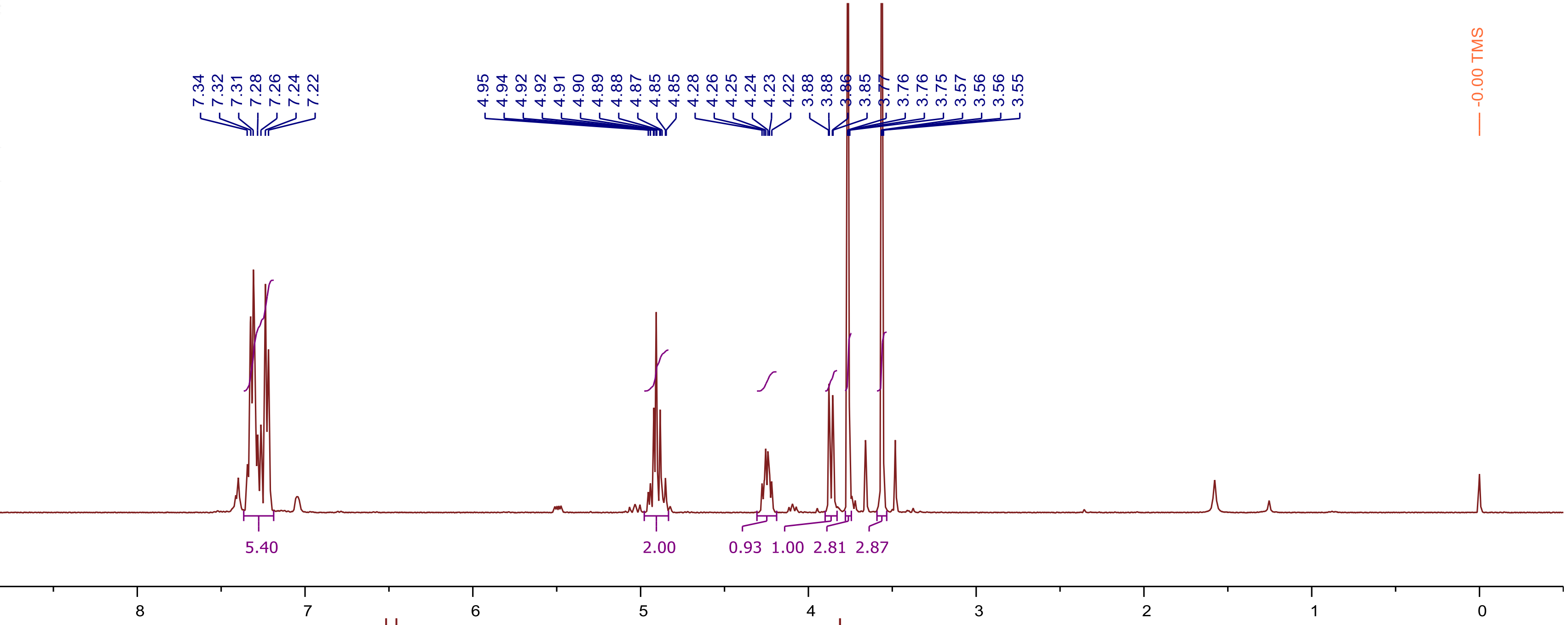

^{13}C NMR (100 MHz, $CDCl_3$)

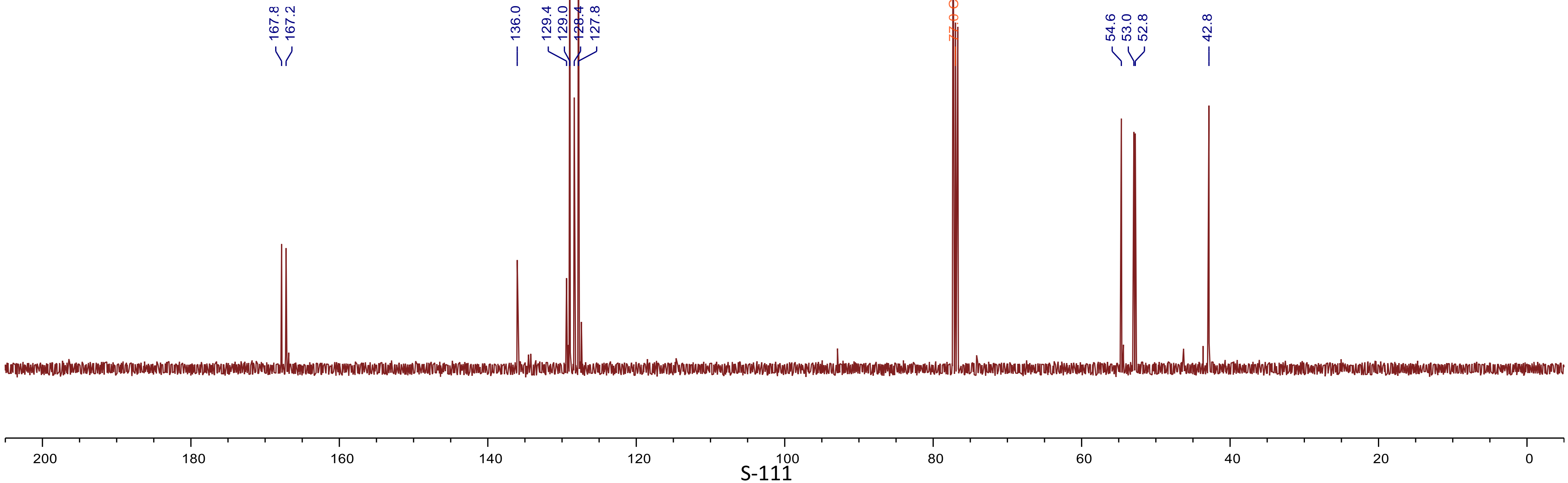

^{1}H NMR (400MHz, $CDCl_3$)

^{19}F NMR (376 MHz, $CDCl_3$)

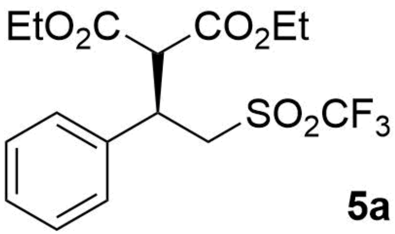

5a

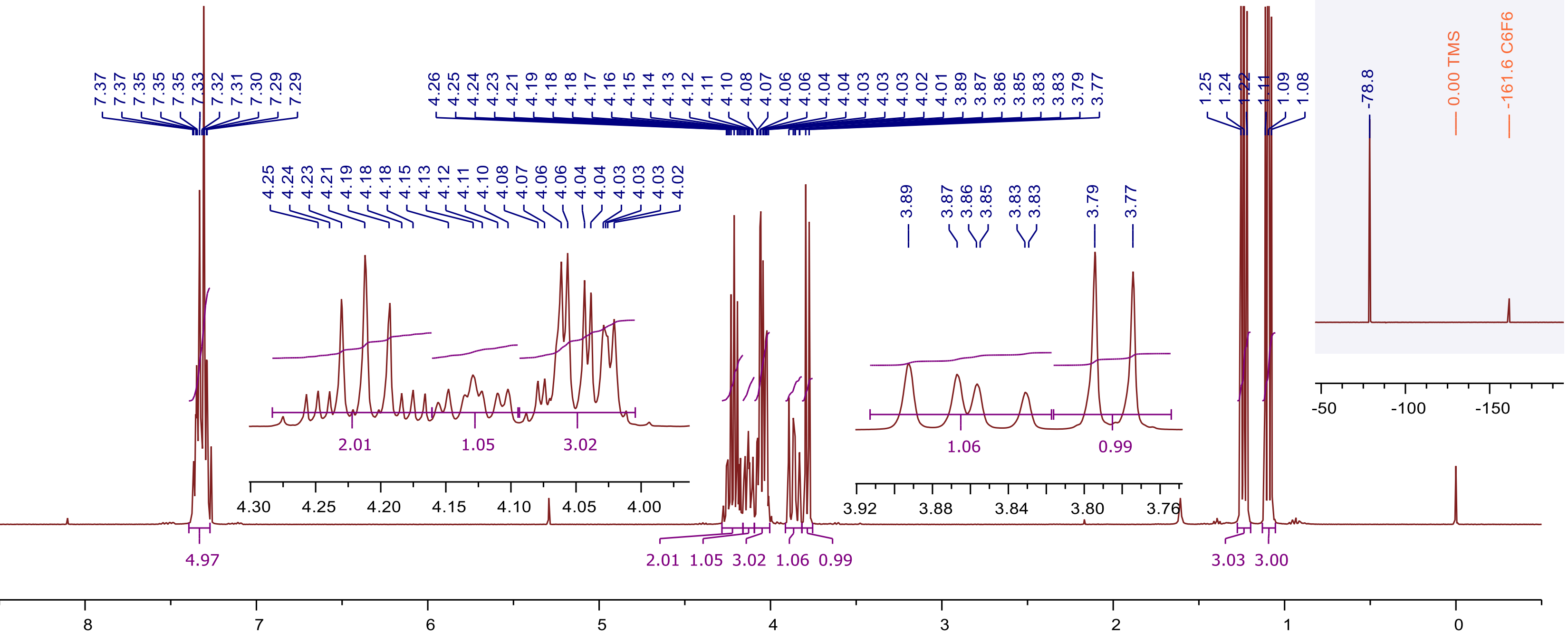

^{13}C NMR (100 MHz, $CDCl_3$)

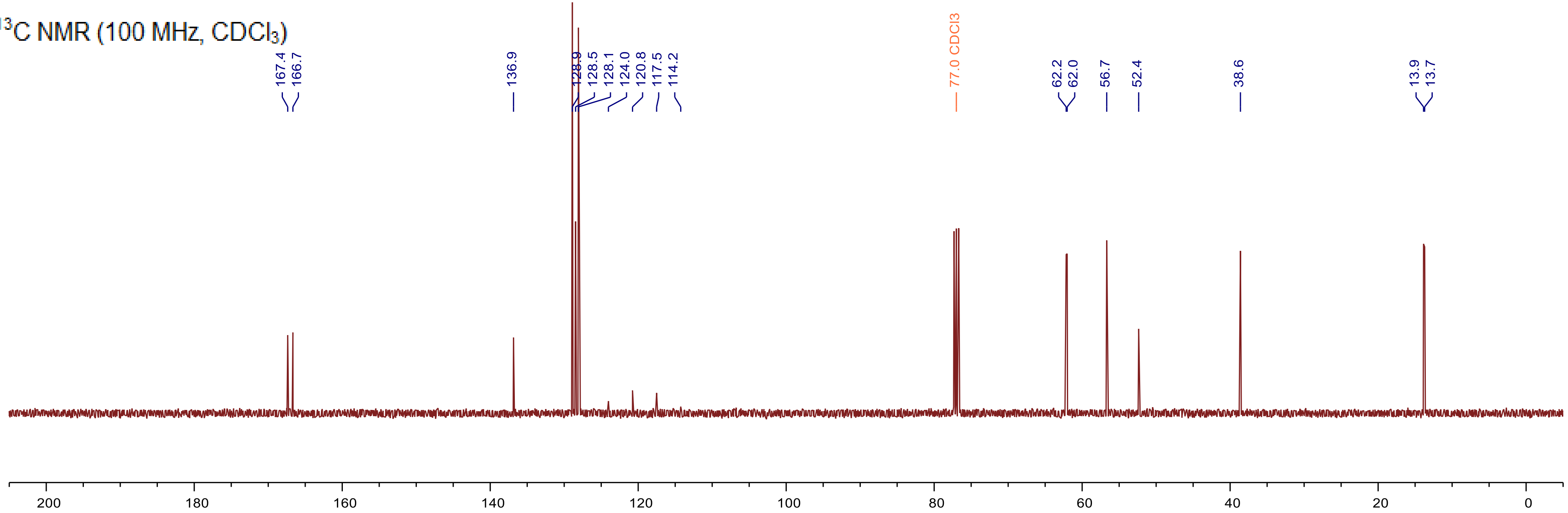

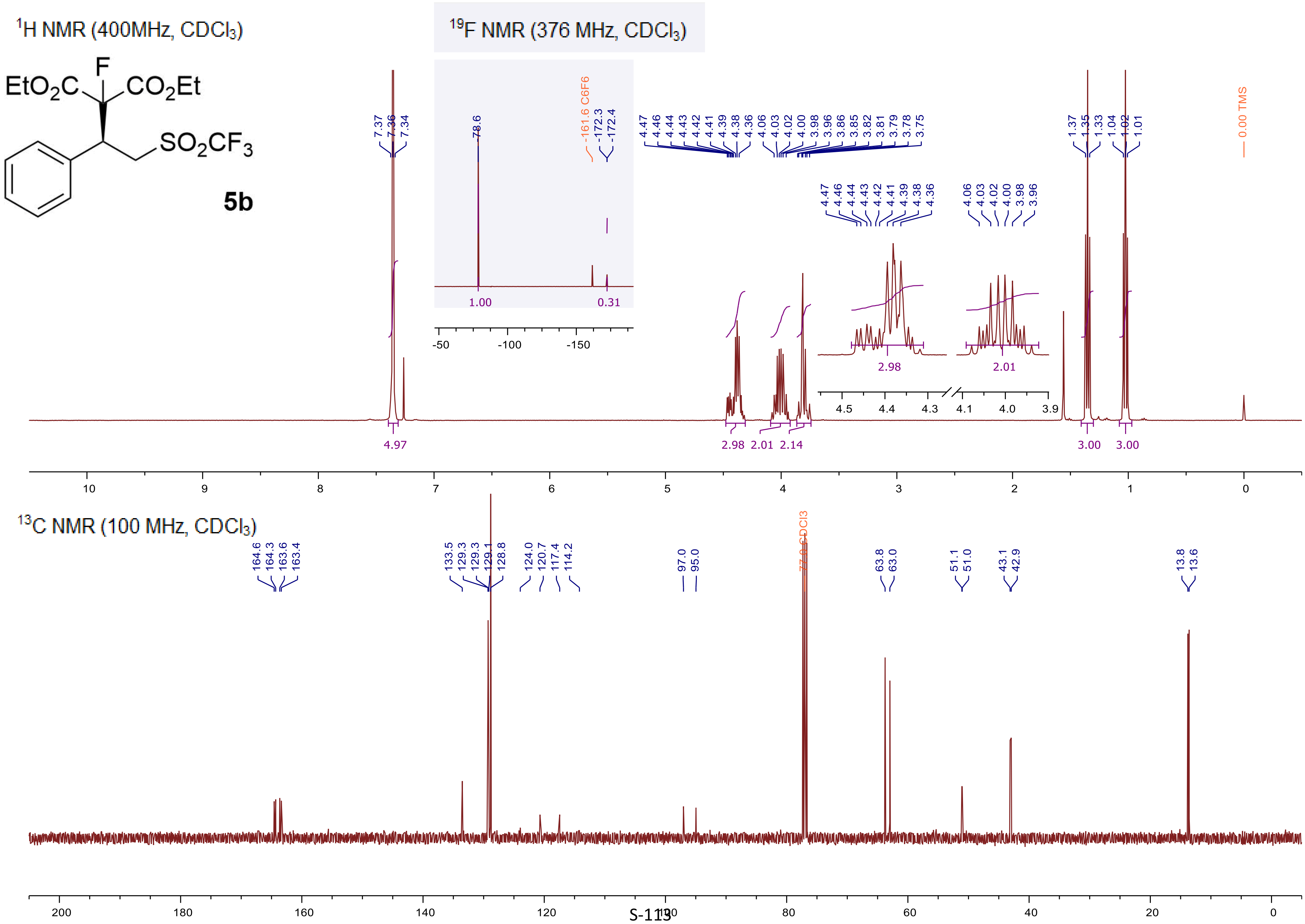
1H NMR (400MHz, CDCl3)
19F NMR (376 MHz, CDCl3)
13C NMR (100 MHz, CDCl3)
F
EtO2C
CO2Et
SO2CF3
5b
-161.6 C6F6
0.00 TMS
77.0 CDCl3

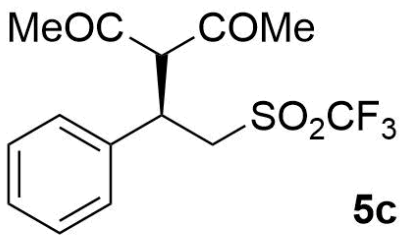
MeOC
COMe
SO2CF3

5c

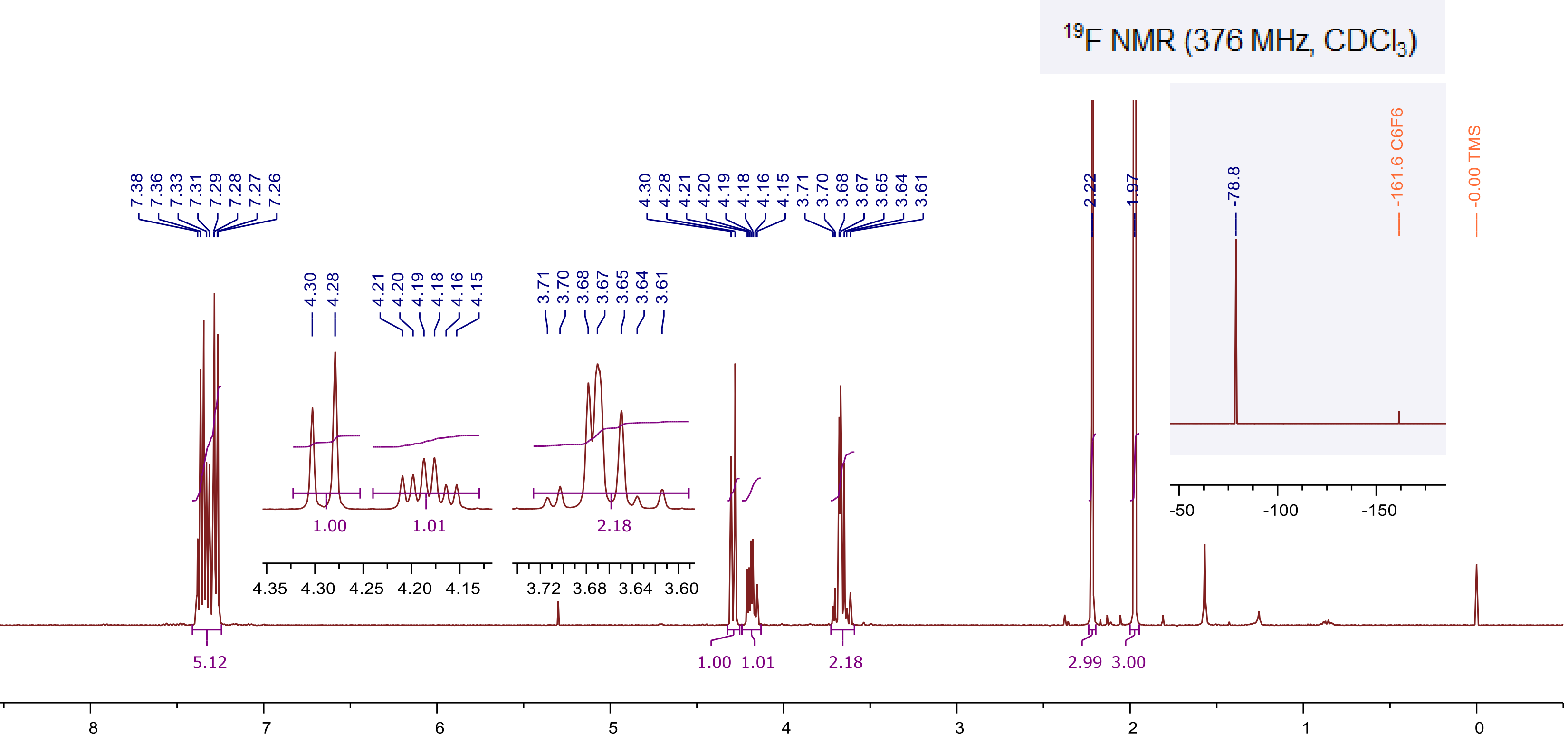
1H NMR (400MHz, CDCl3)
19F NMR (376 MHz, CDCl3)
-78.8
-161.6 C6F6
-0.00 TMS
5.12
1.00 1.01
2.18
2.99 3.00

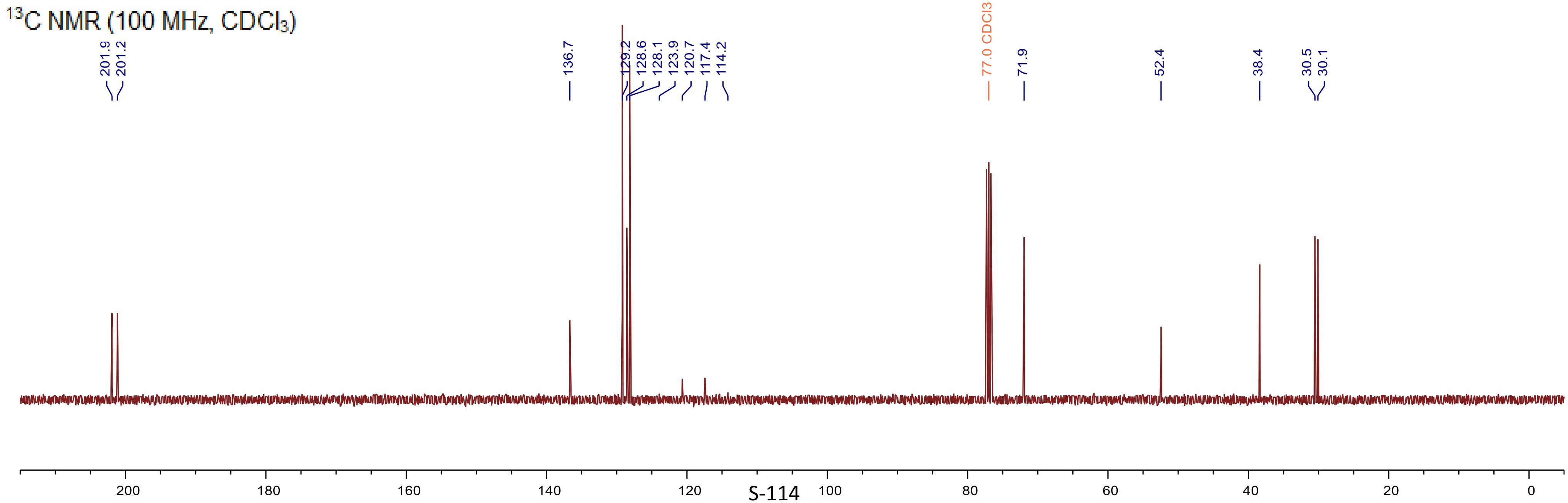
13C NMR (100 MHz, CDCl3)
201.9
201.2
136.7
129.2
128.6
128.1
123.9
120.7
117.4
114.2
77.0 CDCl3
71.9
52.4
38.4
30.5
30.1

^{1}H NMR (400MHz, $CDCl_3$)

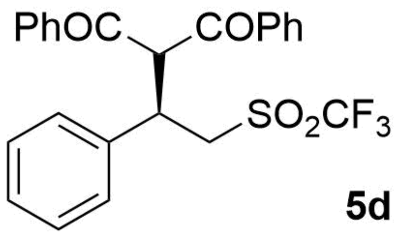

5d

^{19}F NMR (376 MHz, $CDCl_3$)

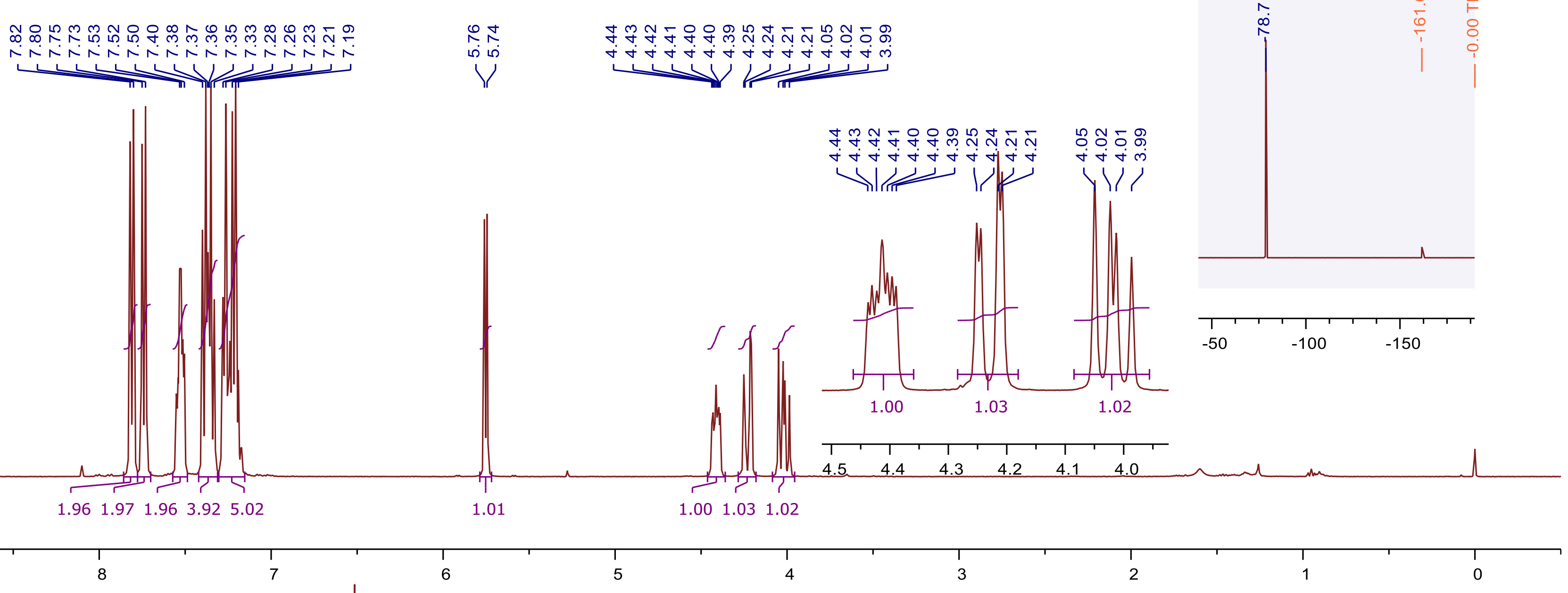

^{13}C NMR (100 MHz, $CDCl_3$)

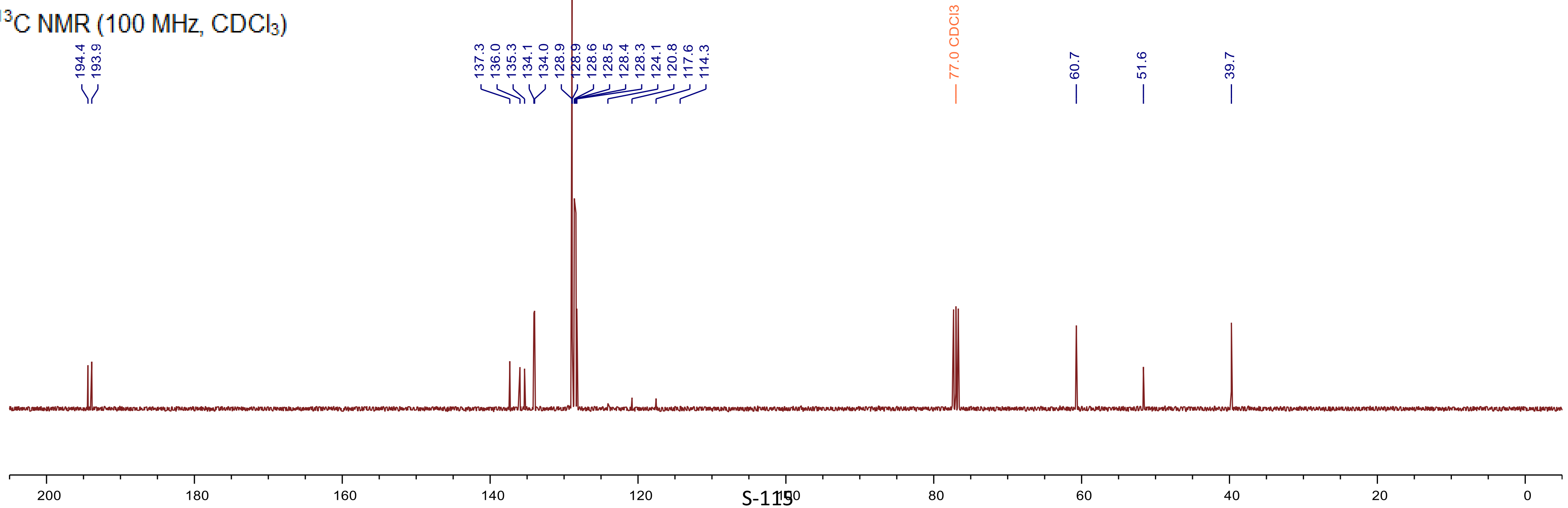

^{1}H NMR (400MHz, $CDCl_3$)

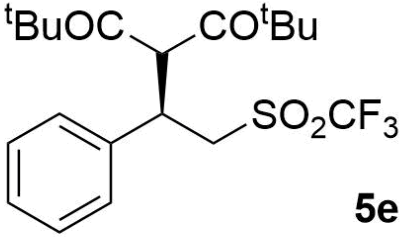

5e

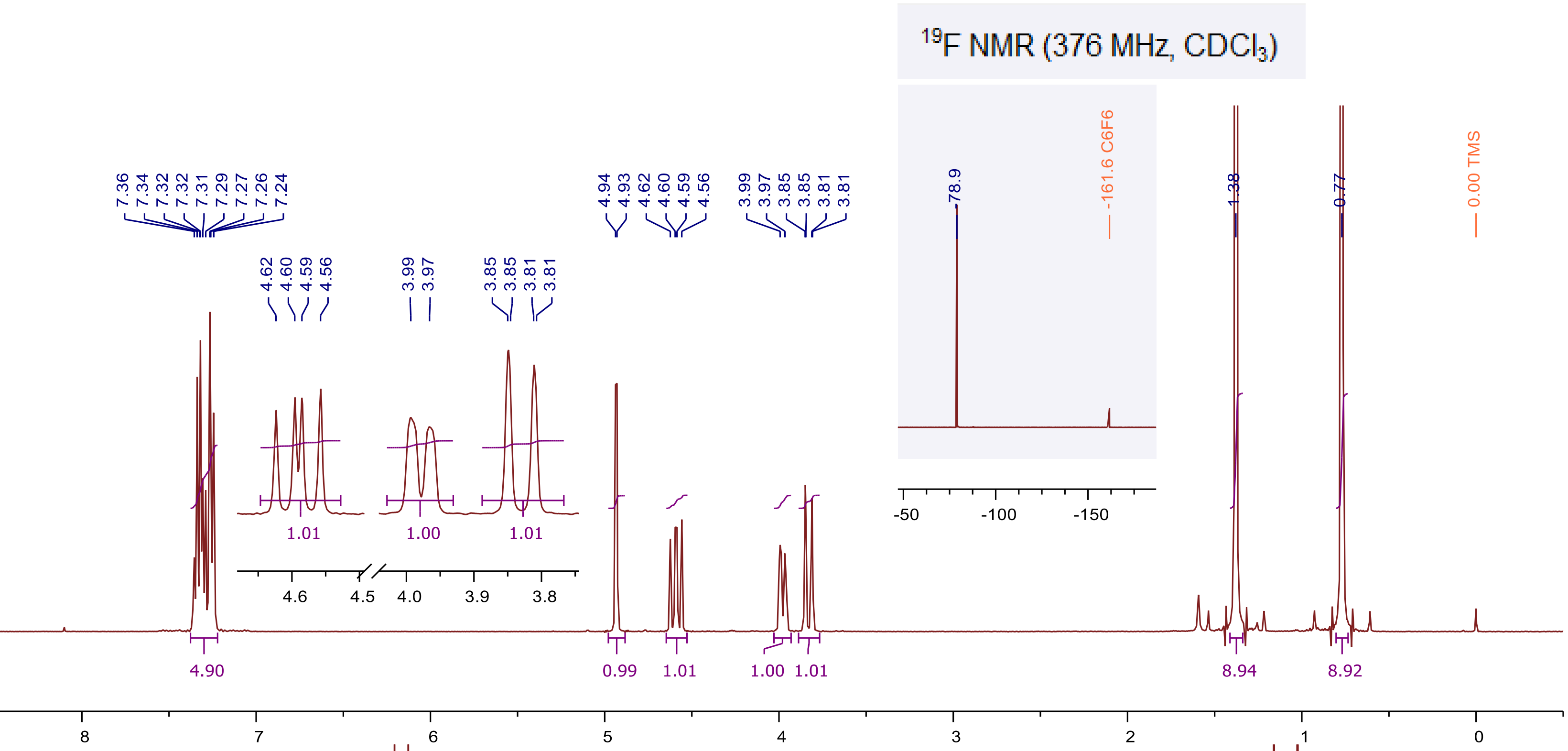

^{13}C NMR (100 MHz, $CDCl_3$)

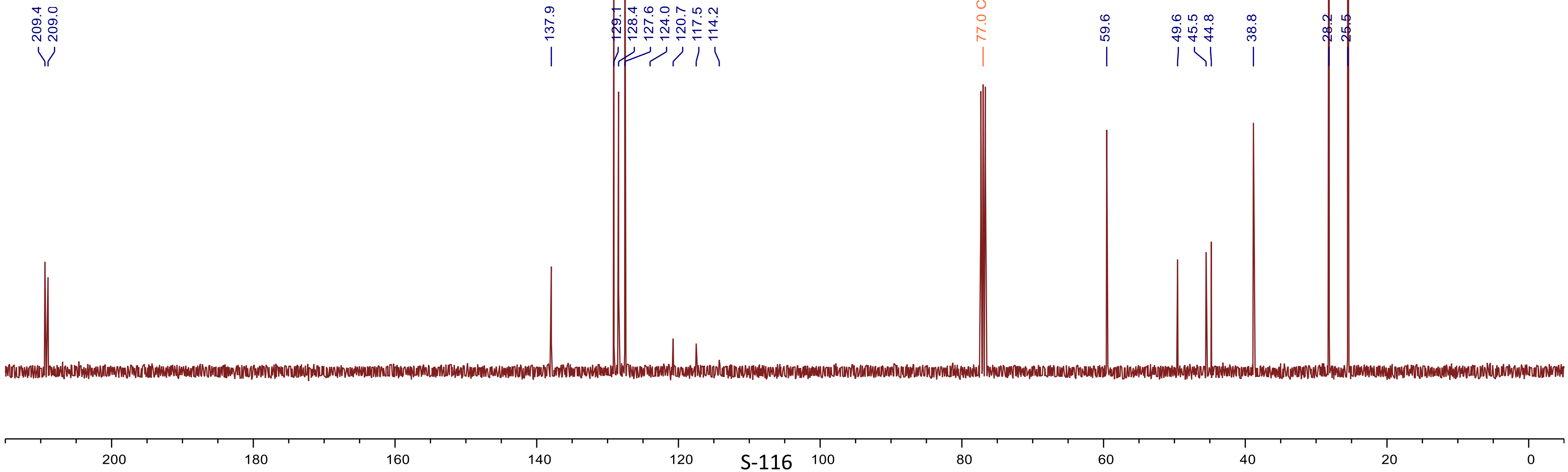

^{1}H NMR (400MHz, $CDCl_3$)

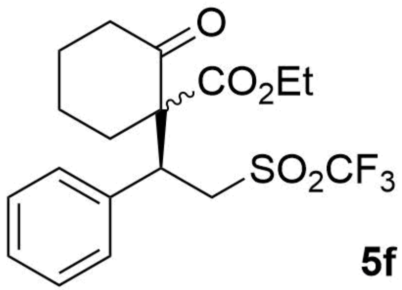

5f

^{19}F NMR (376 MHz, $CDCl_3$)

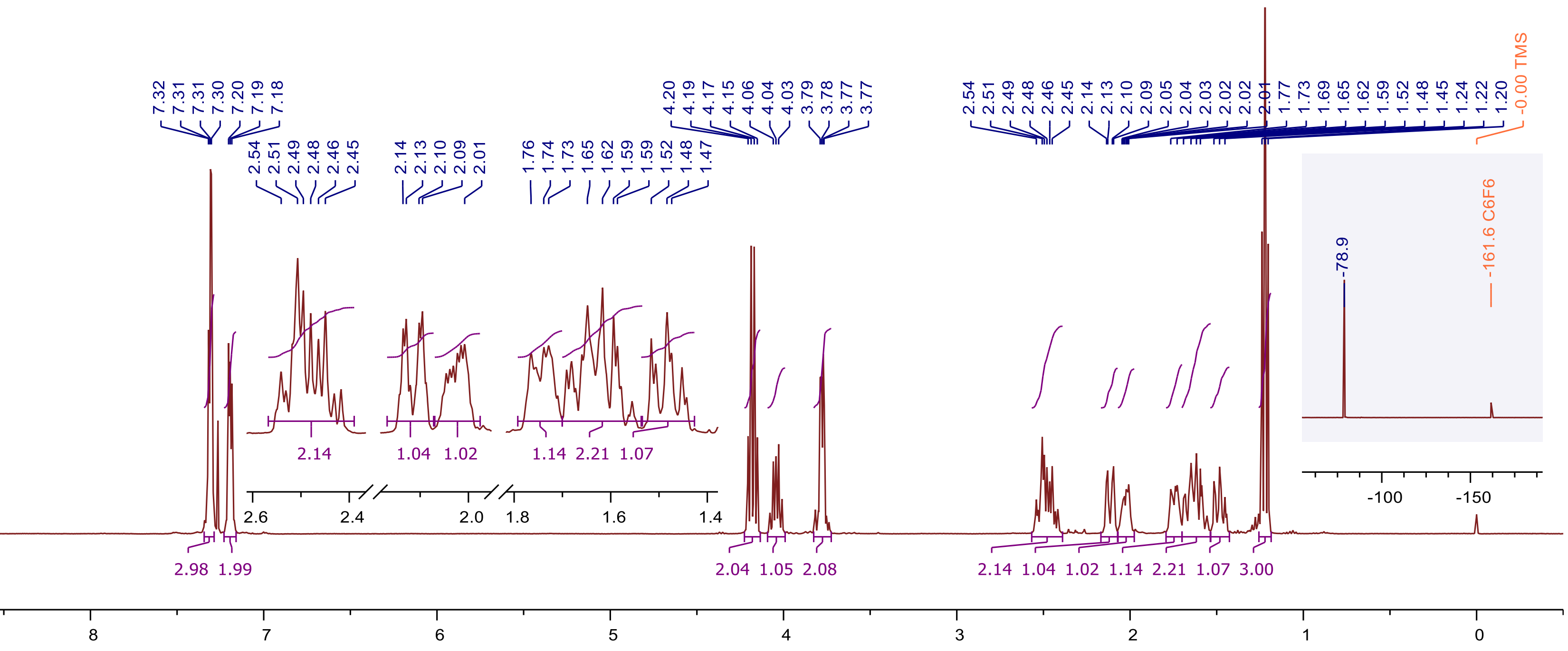

^{13}C NMR (100 MHz, $CDCl_3$)

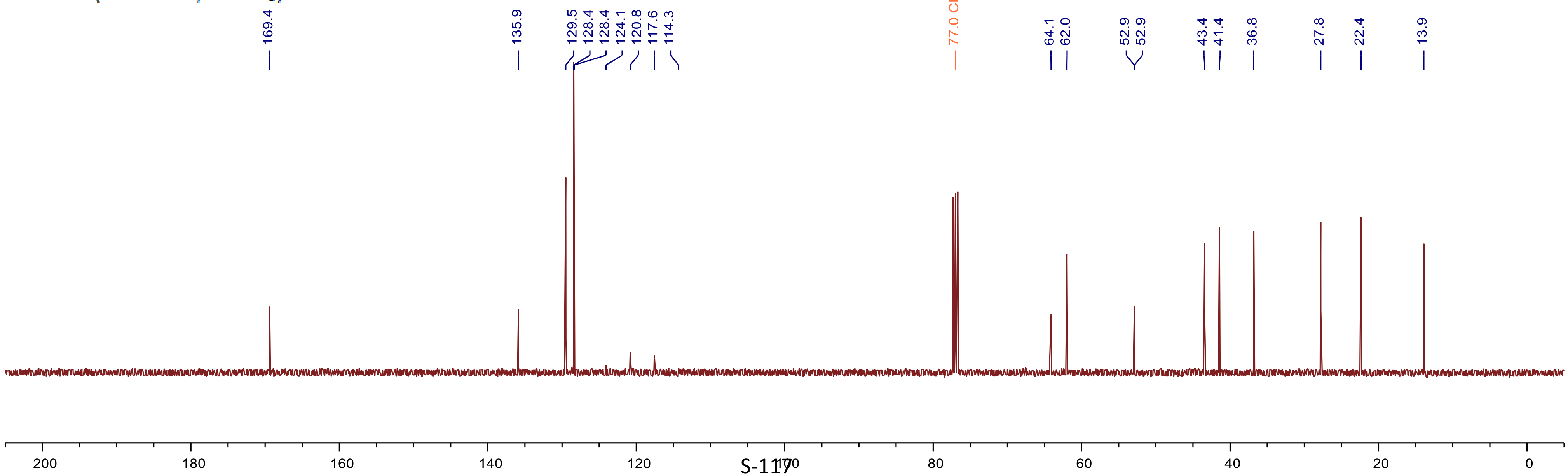

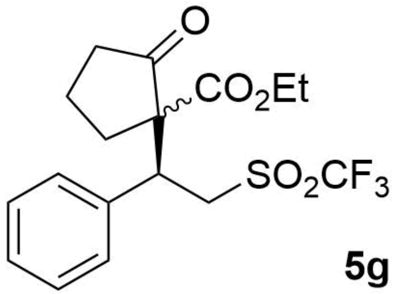

5g

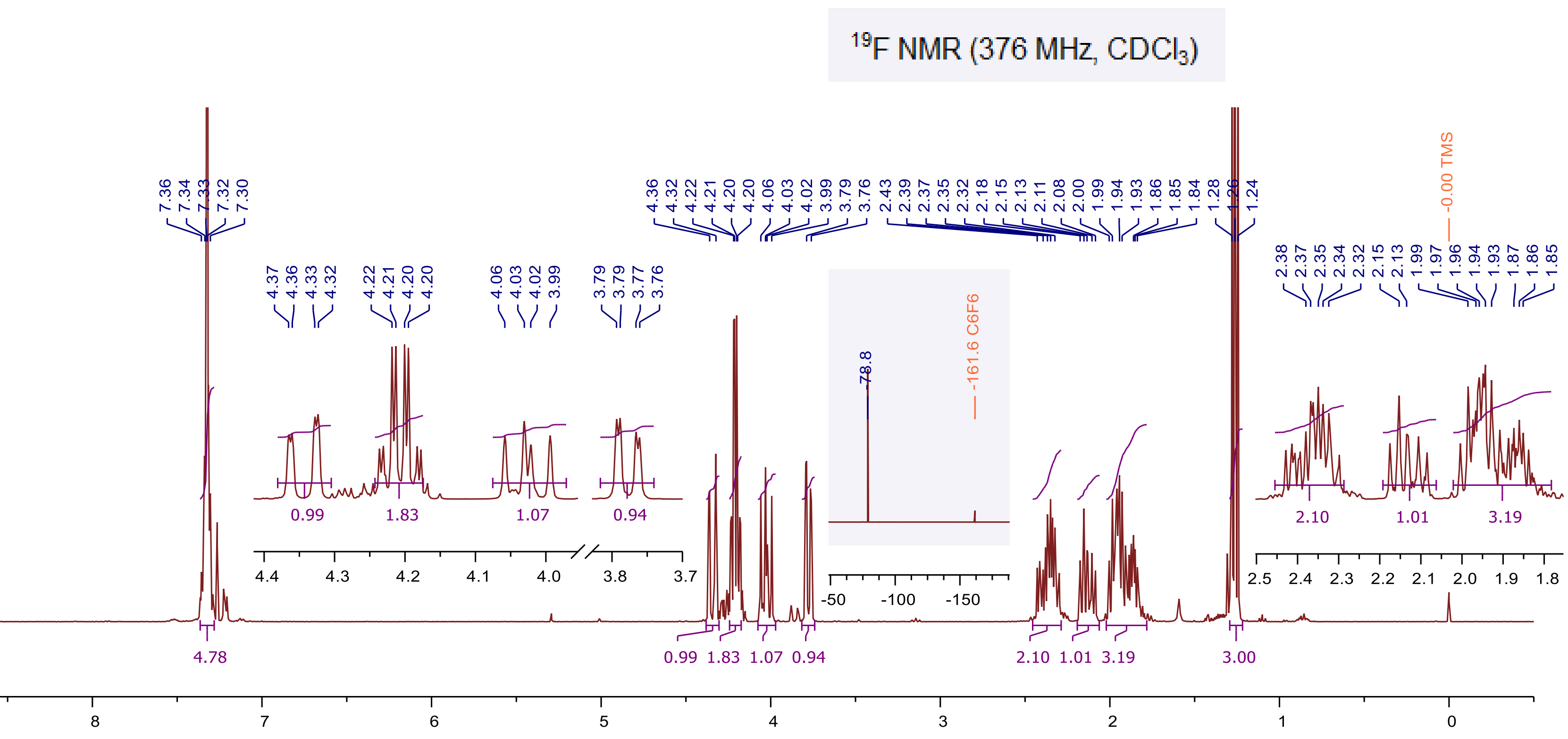

^{1}H NMR (400MHz, CDCl$_3$)
^{19}F NMR (376 MHz, CDCl$_3$)
-78.8
-161.6 C6F6
-0.00 TMS
4.78
0.99 1.83 1.07 0.94
2.10 1.01 3.19
3.00

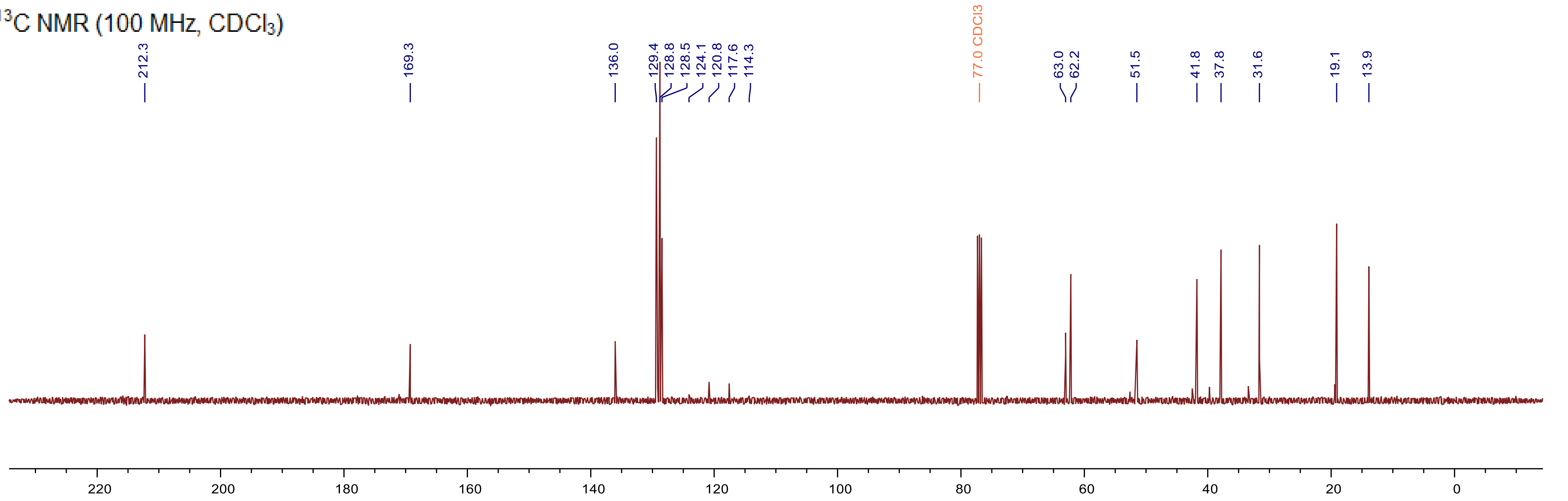

^{13}C NMR (100 MHz, CDCl$_3$)
212.3
169.3
136.0
129.4
128.8
128.5
124.1
120.8
117.6
114.3
77.0 CDCl3
63.0
62.2
51.5
41.8
37.8
31.6
19.1
13.9

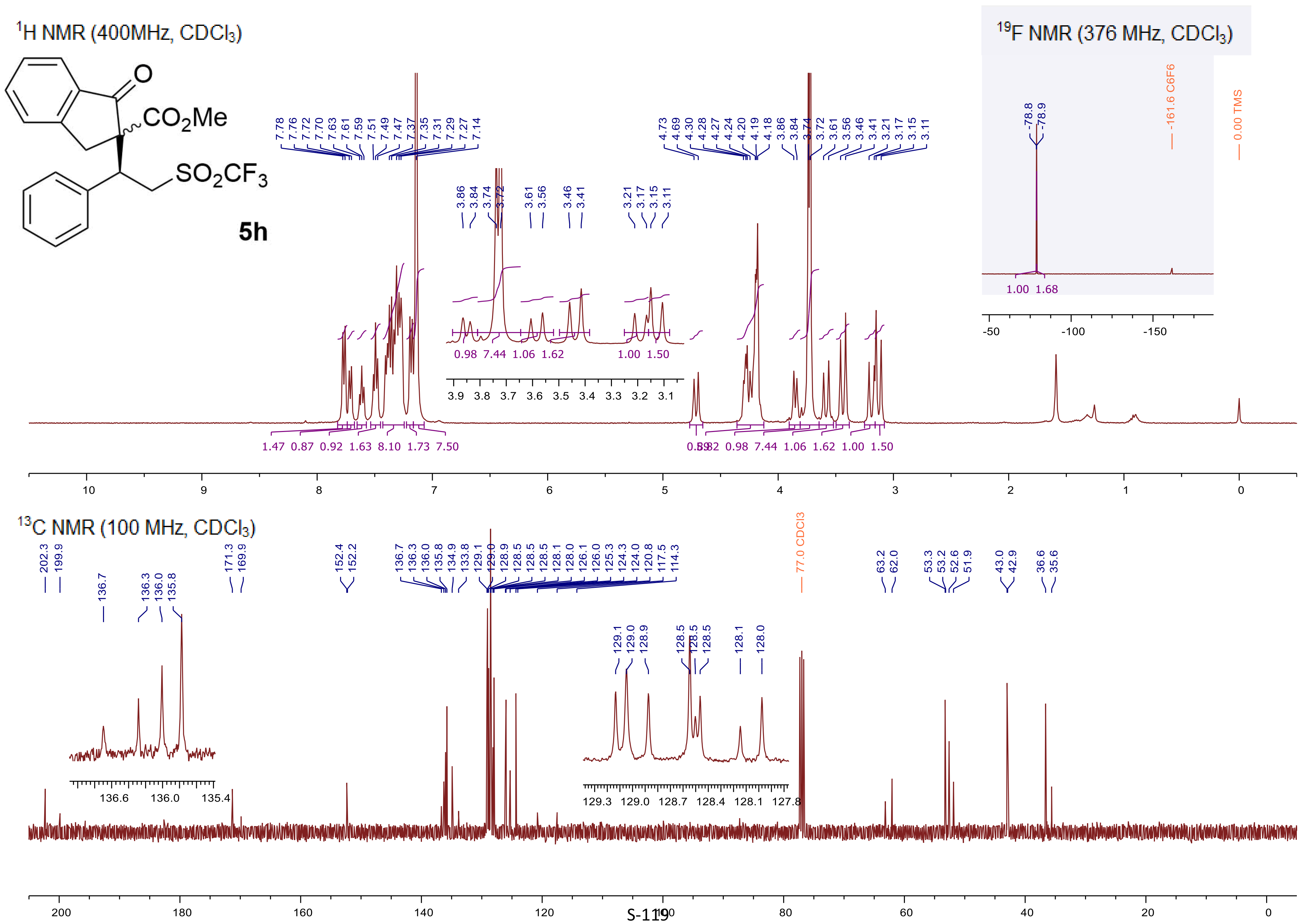
1H NMR (400MHz, CDCl3)
CO2Me
SO2CF3
5h
19F NMR (376 MHz, CDCl3)
-78.8
-78.9
-161.6 C6F6
0.00 TMS
1.00 1.68
7.78 7.76 7.72 7.70 7.63 7.61 7.59 7.51 7.49 7.47 7.37 7.35 7.31 7.29 7.27 7.14
4.73 4.69 4.30 4.28 4.27 4.24 4.20 4.19 4.18 3.86 3.84 3.74 3.72 3.61 3.56 3.46 3.41 3.21 3.17 3.15 3.11
1.47 0.87 0.92 1.63 8.10 1.73 7.50
0.98 7.44 1.06 1.62 1.00 1.50
13C NMR (100 MHz, CDCl3)
202.3 199.9 136.7 136.3 136.0 135.8 171.3 169.9 152.4 152.2
136.7 136.3 136.0 135.8 134.9 133.8 129.1 129.0 128.9 128.5 128.5 128.5 128.1 128.0 126.1 126.0 125.3 124.3 124.0 120.8 117.5 114.3
77.0 CDCl3
63.2 62.0 53.3 53.2 52.6 51.9 43.0 42.9 36.6 35.6

^{1}H NMR (400MHz, $CDCl_3$)

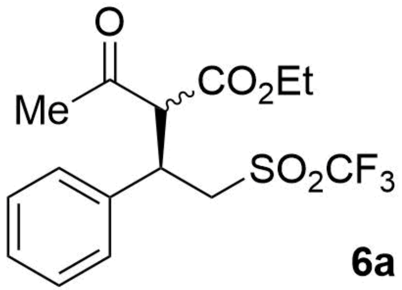

6a

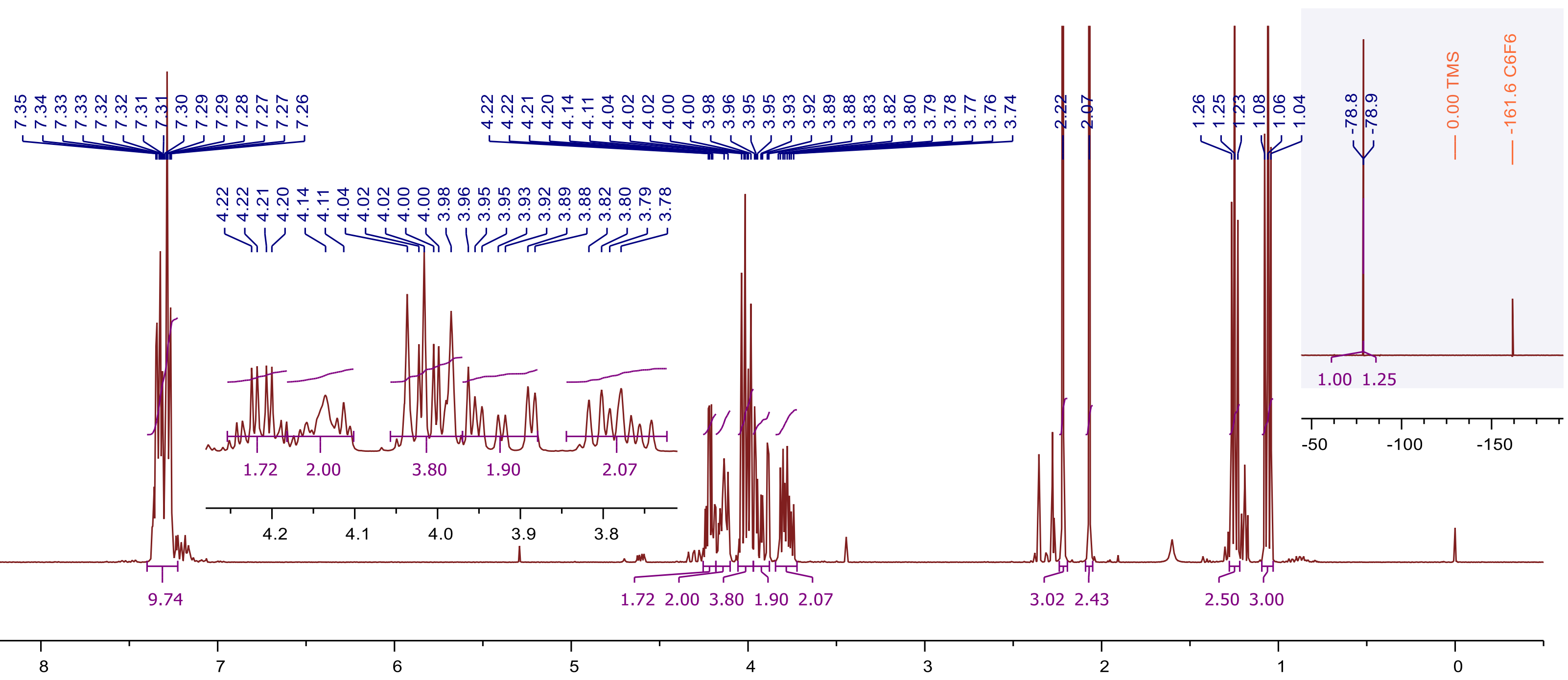

^{13}C NMR (100 MHz, $CDCl_3$)

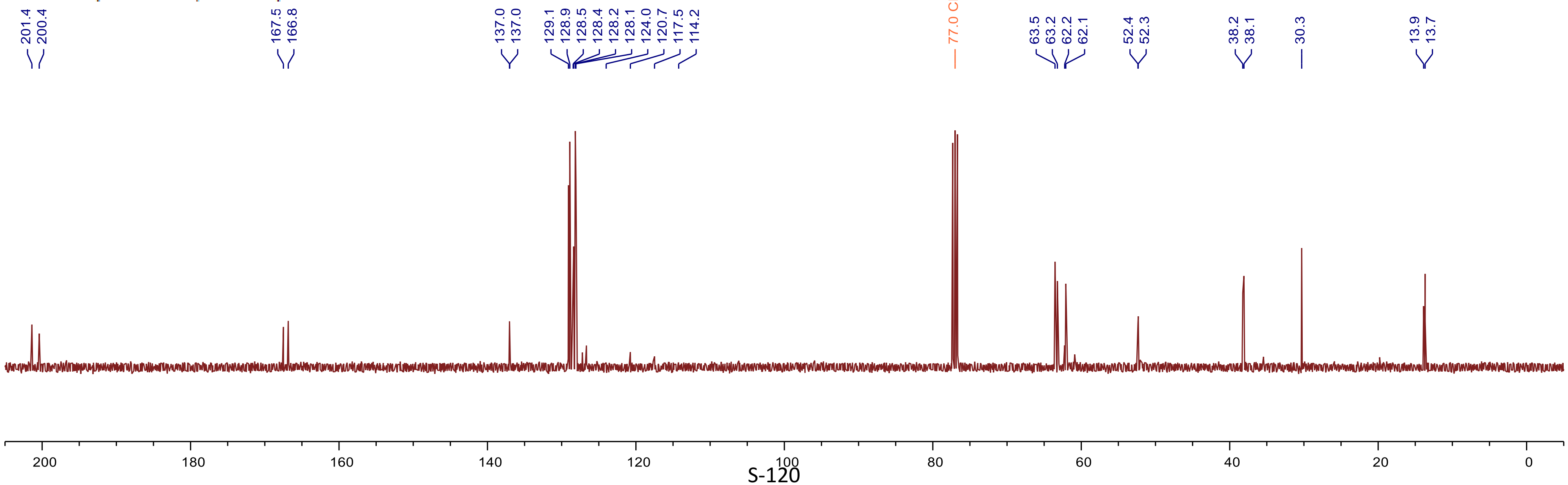

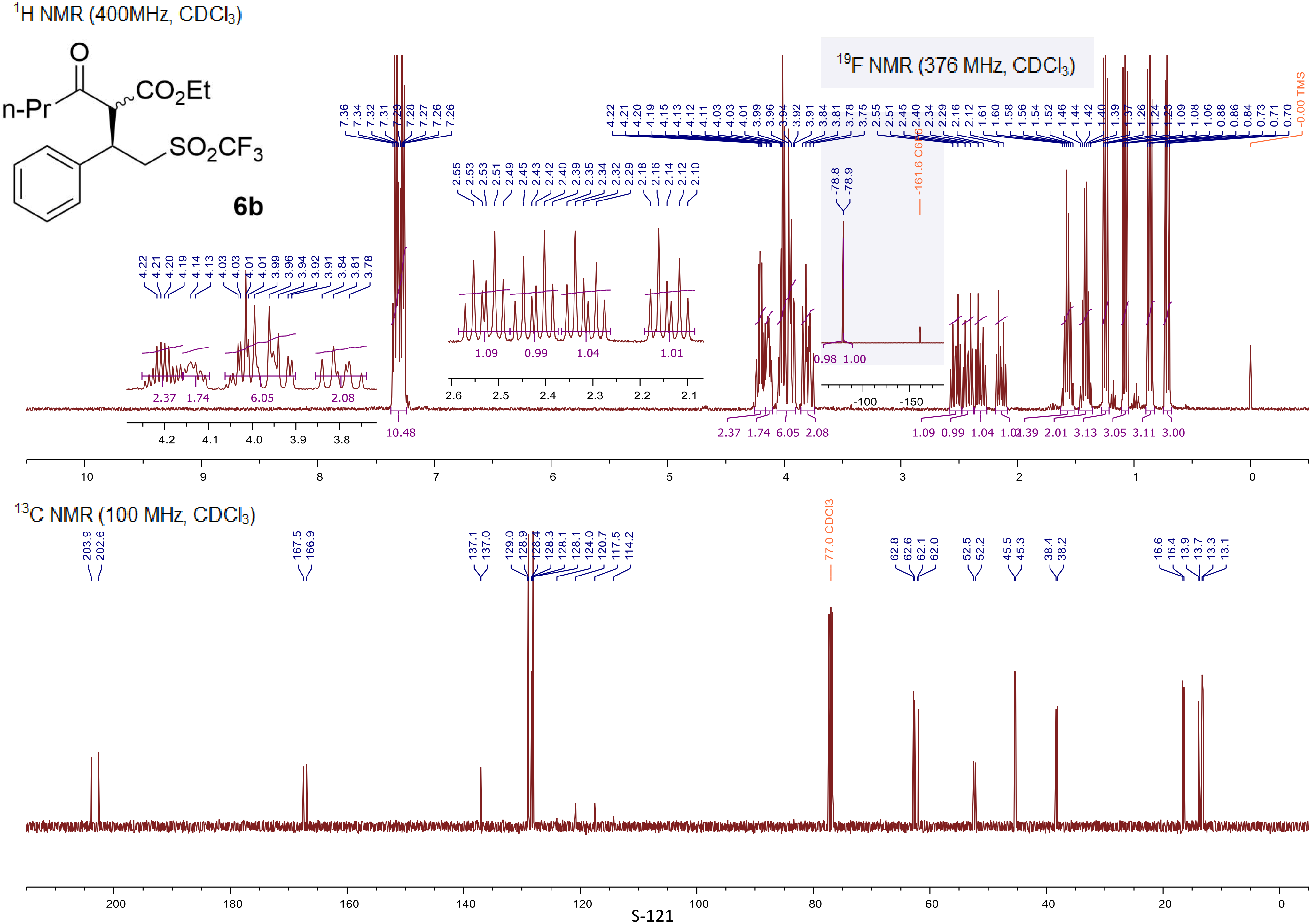

1H NMR (400MHz, CDCl3)
n-Pr
CO2Et
SO2CF3
6b
19F NMR (376 MHz, CDCl3)
13C NMR (100 MHz, CDCl3)

^{1}H NMR (400MHz, $CDCl_3$) ^{19}F NMR (376 MHz, $CDCl_3$)

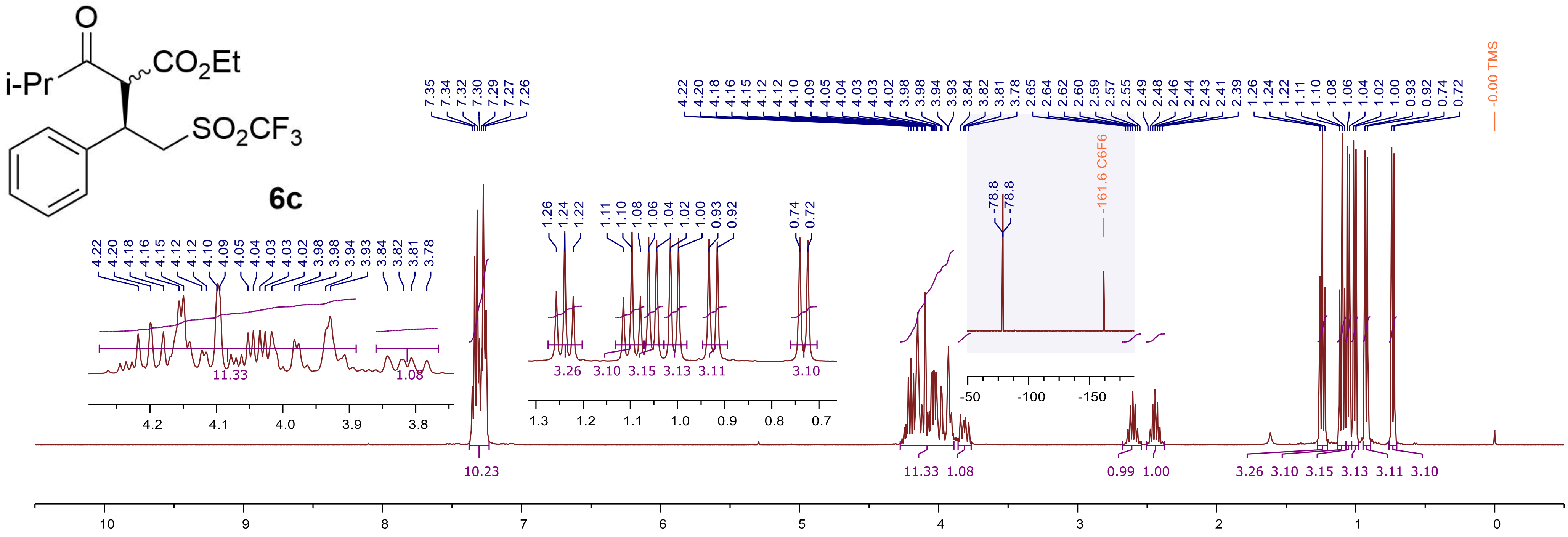

6c

^{13}C NMR (100 MHz, $CDCl_3$)

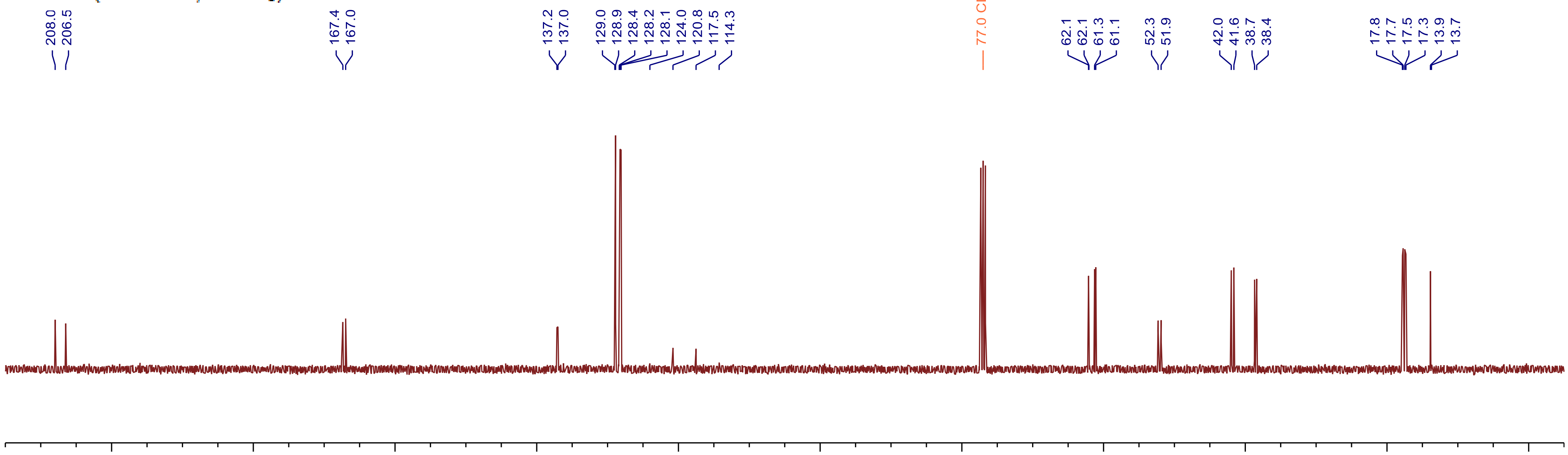

^{1}H NMR (400MHz, $CDCl_3$)

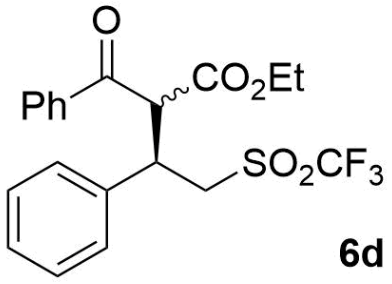

6d

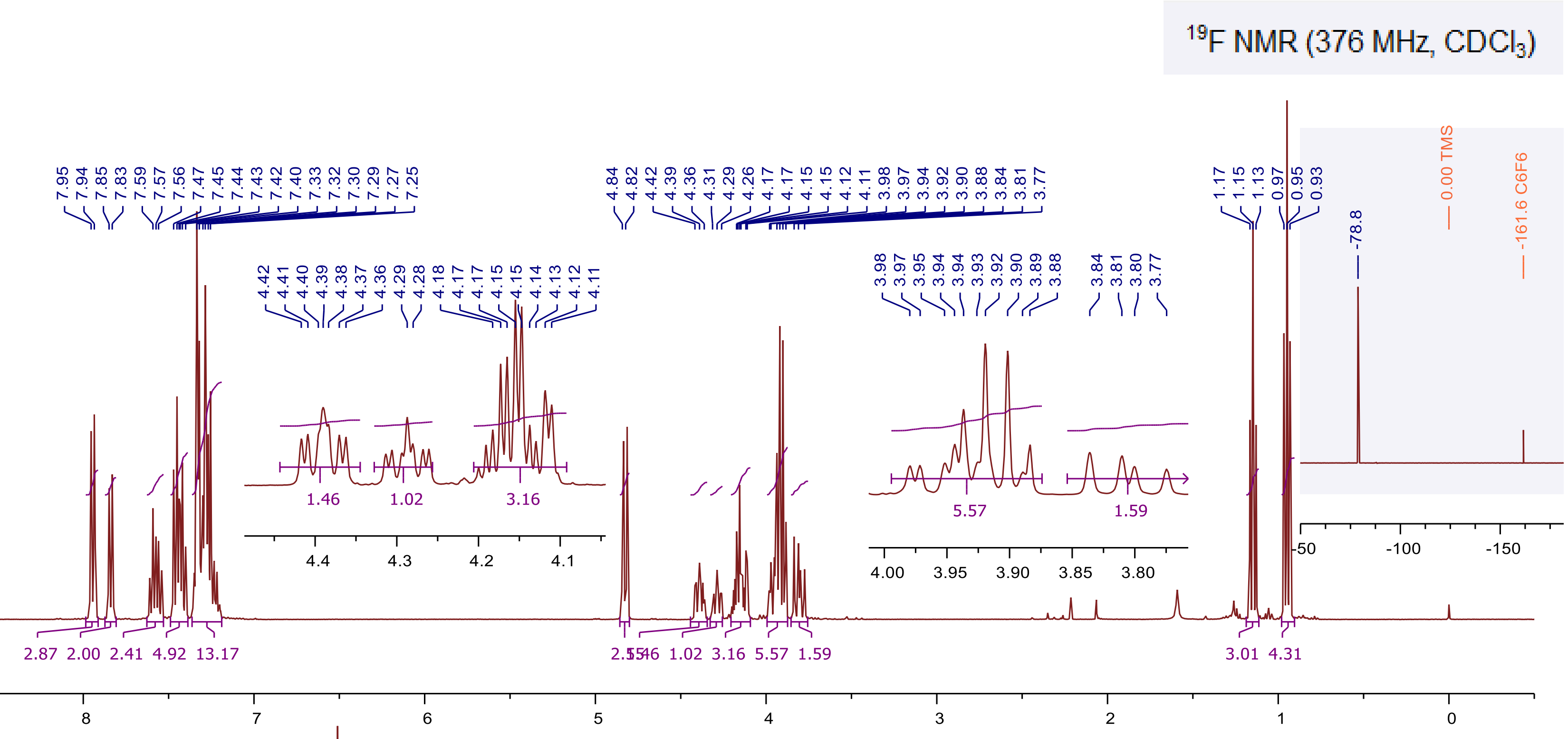

^{13}C NMR (100 MHz, $CDCl_3$)

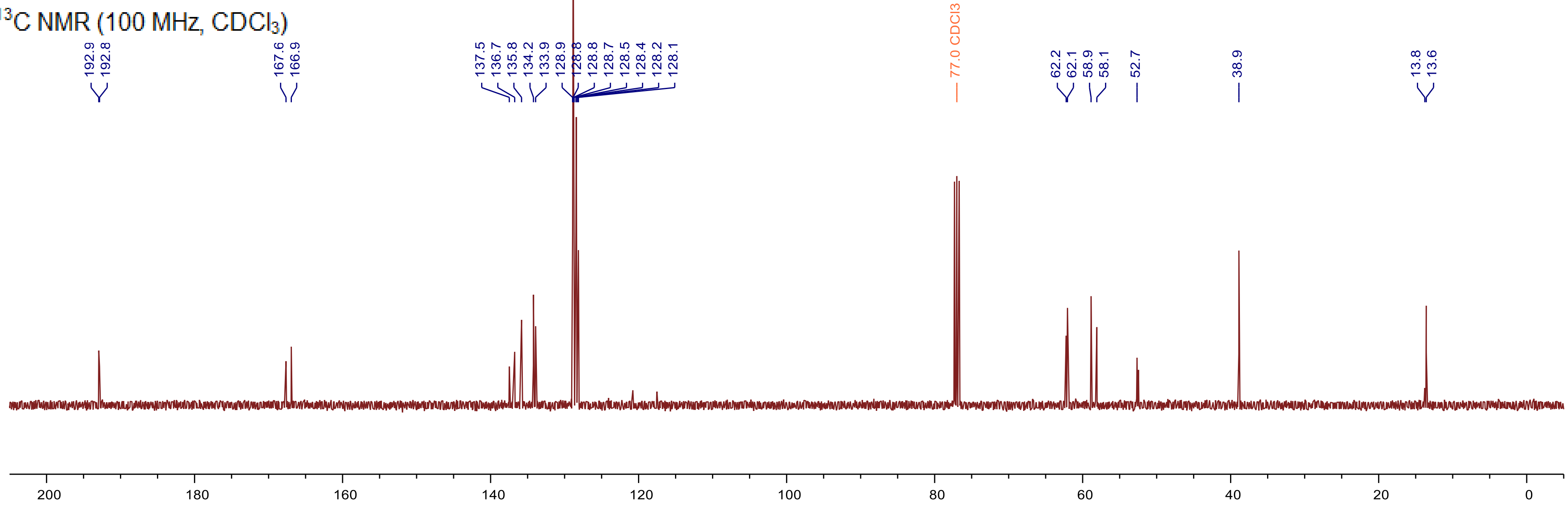

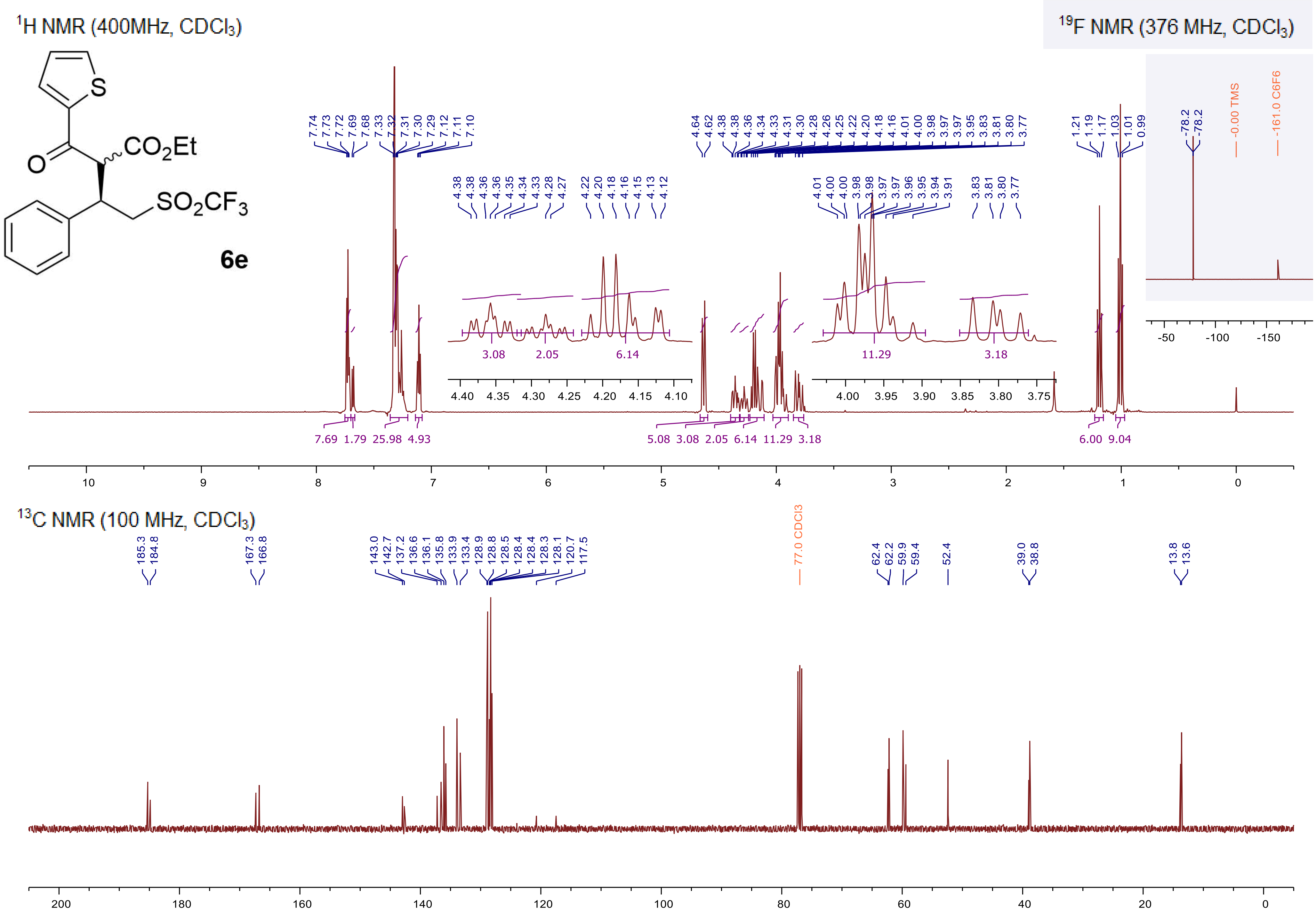

1H NMR (400MHz, CDCl3)
19F NMR (376 MHz, CDCl3)
6e
13C NMR (100 MHz, CDCl3)

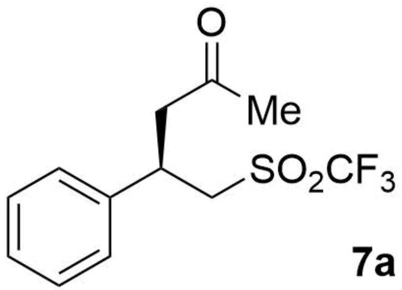

7a

^{1}H NMR (400MHz, $CDCl_3$)

^{19}F NMR (376 MHz, $CDCl_3$)

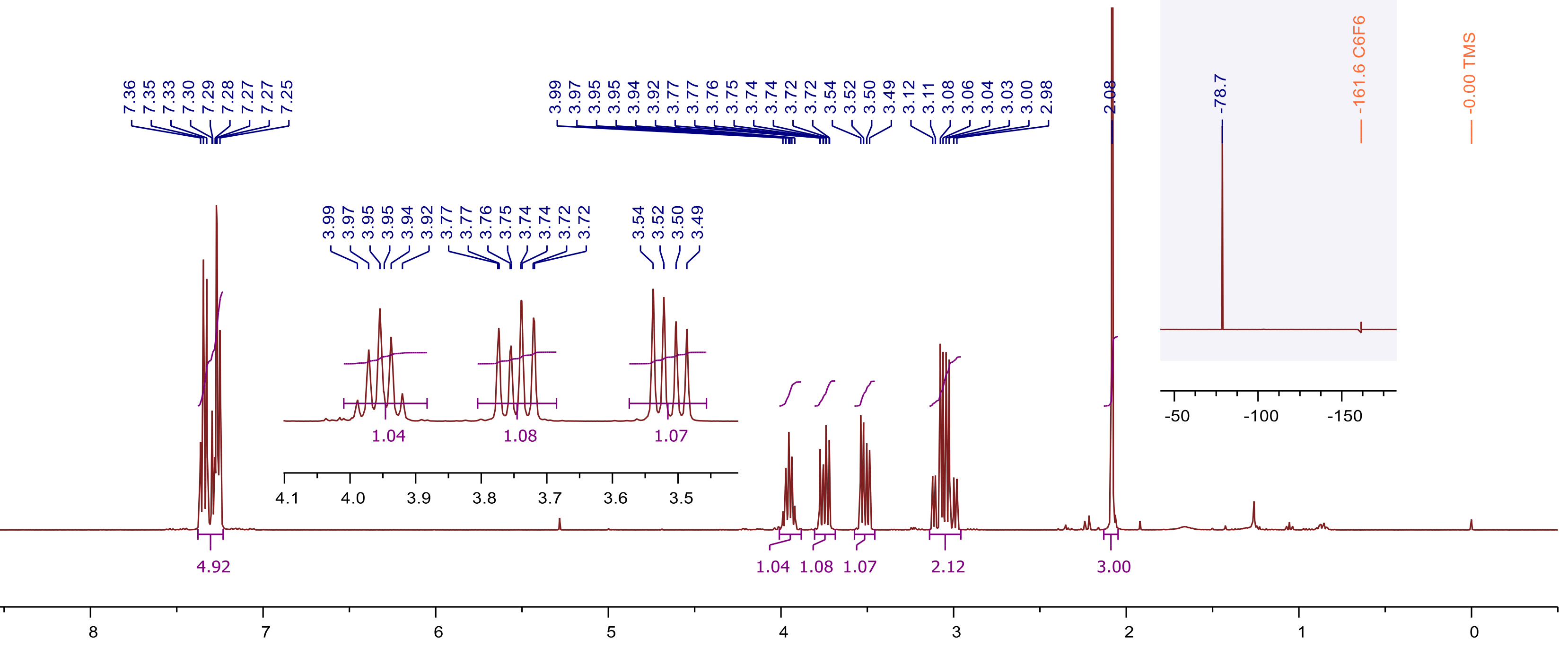

^{13}C NMR (100 MHz, $CDCl_3$)

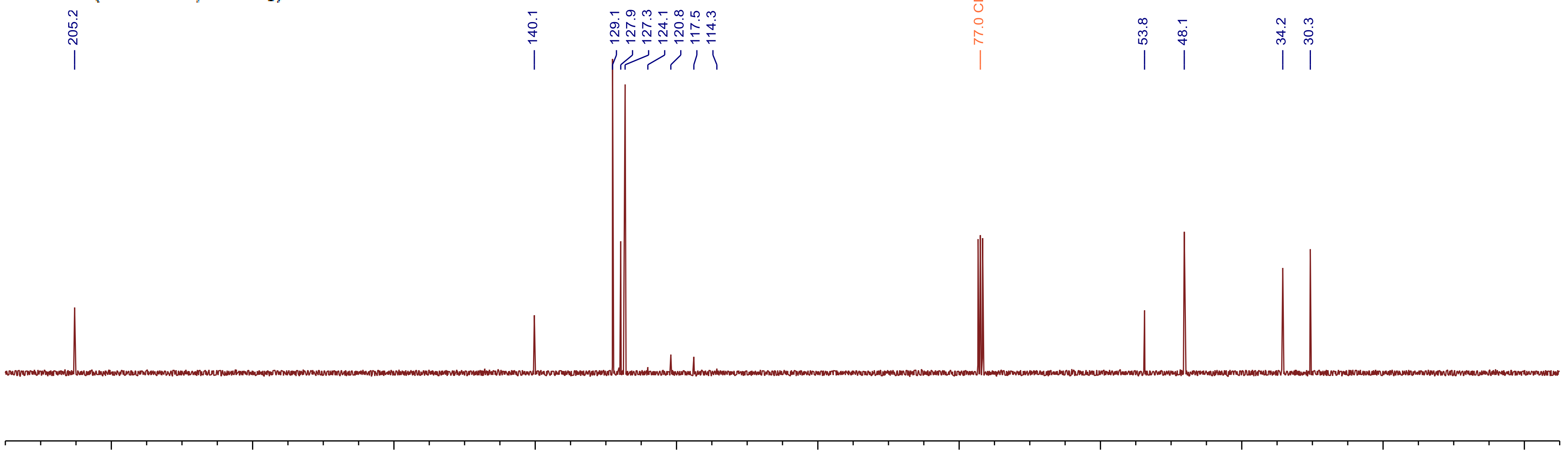

^{1}H NMR (400MHz, $CDCl_3$)

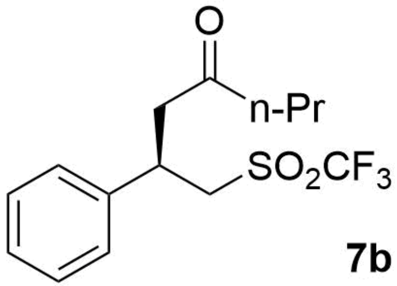

7b

^{19}F NMR (376 MHz, $CDCl_3$)

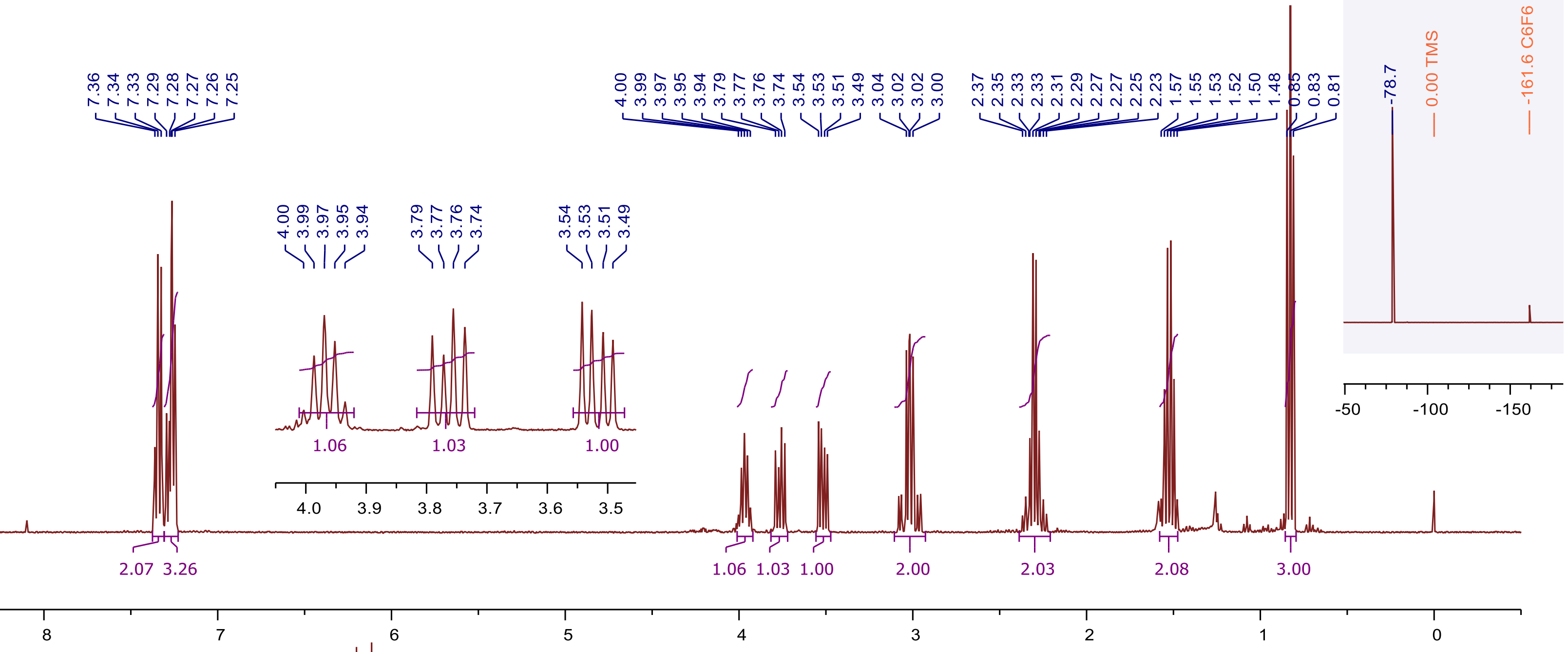

^{13}C NMR (100 MHz, $CDCl_3$)

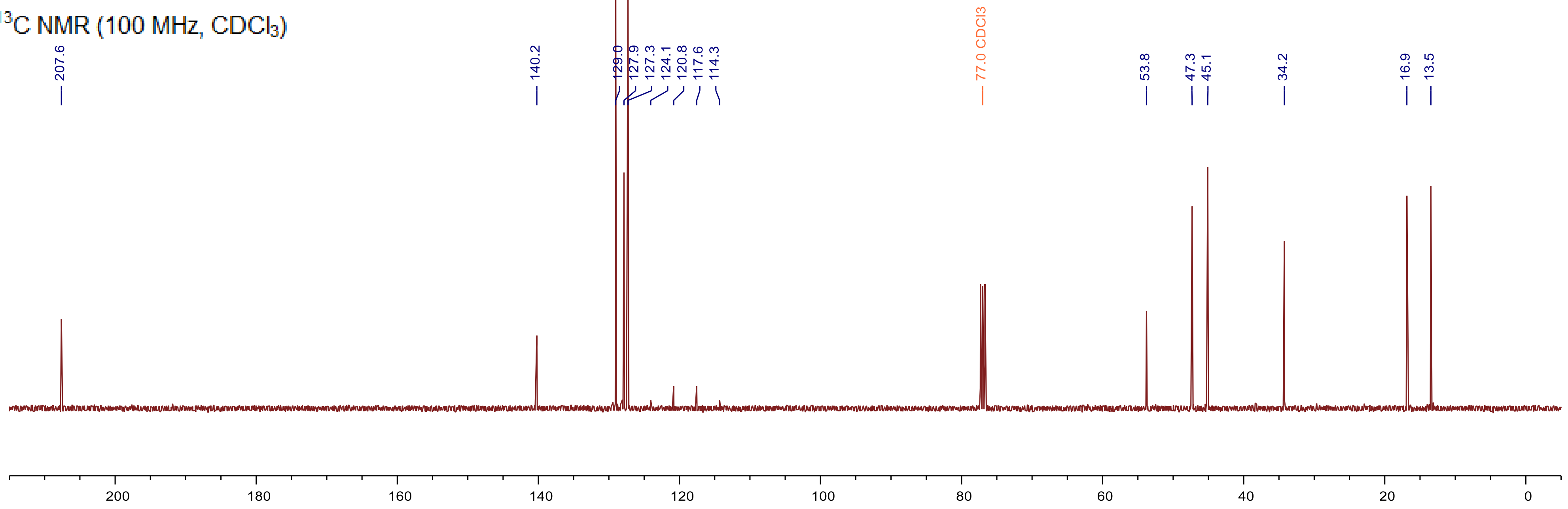

^1H NMR (400MHz, $CDCl_3$)

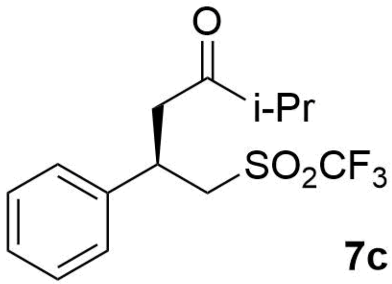

7c

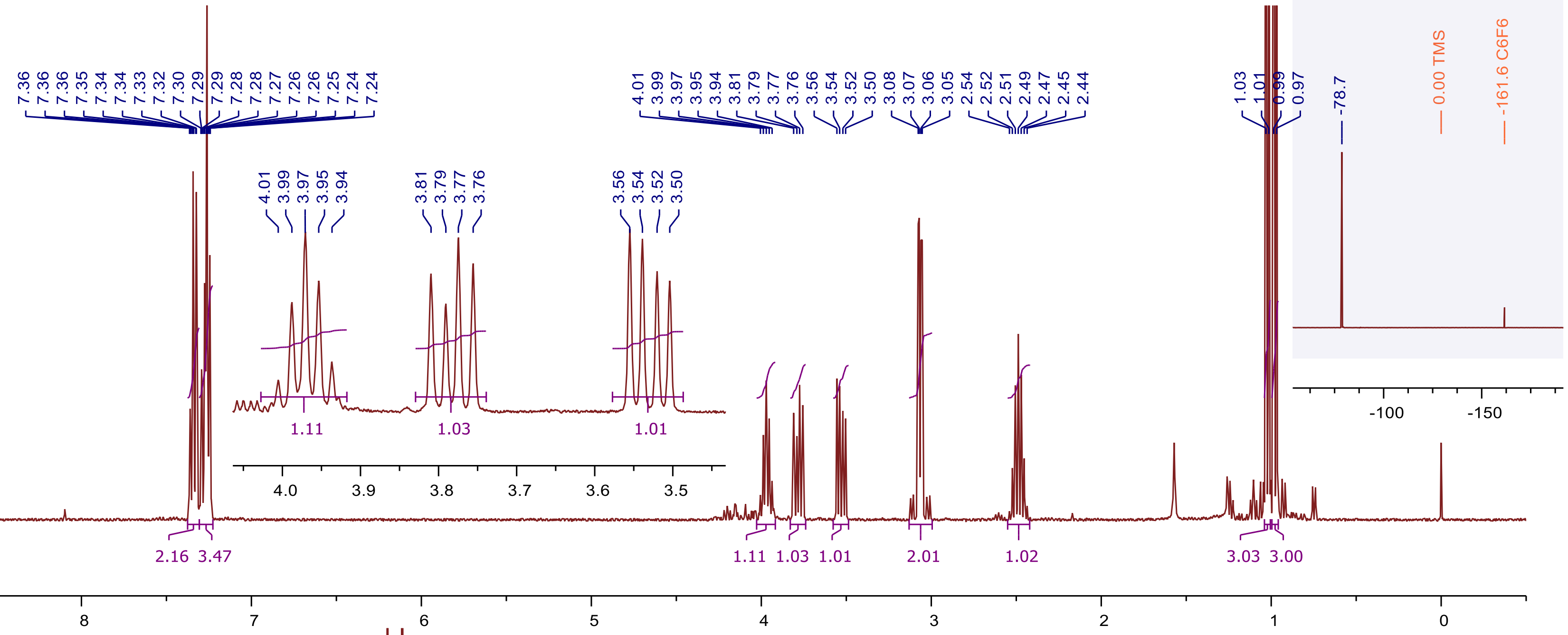

^{13}C NMR (100 MHz, $CDCl_3$)

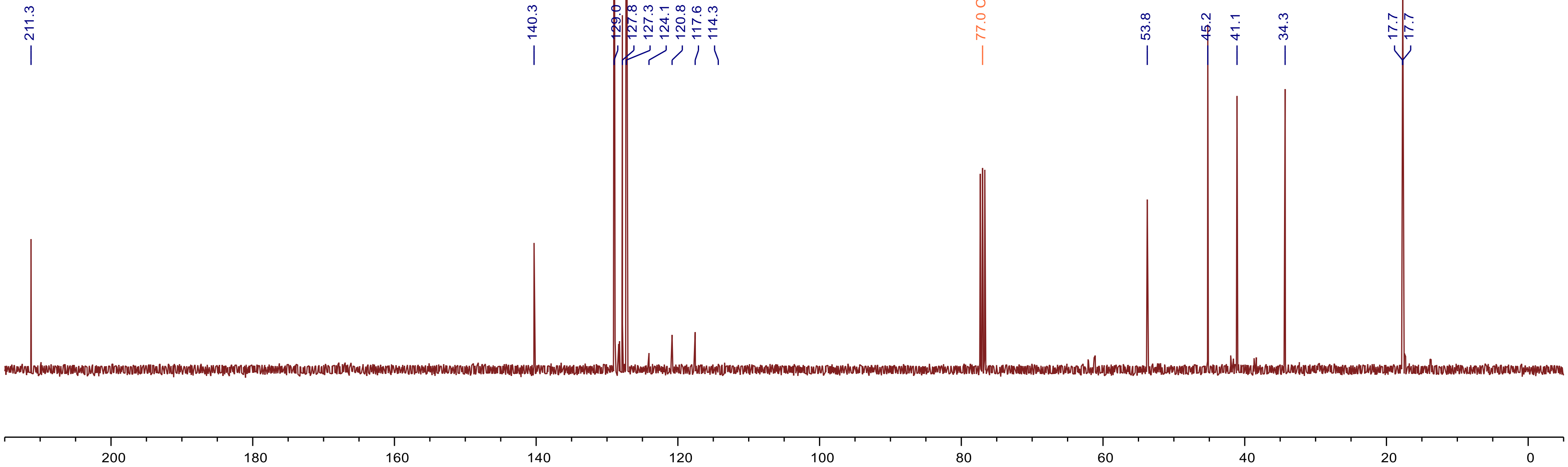

^{1}H NMR (400MHz, $CDCl_3$)

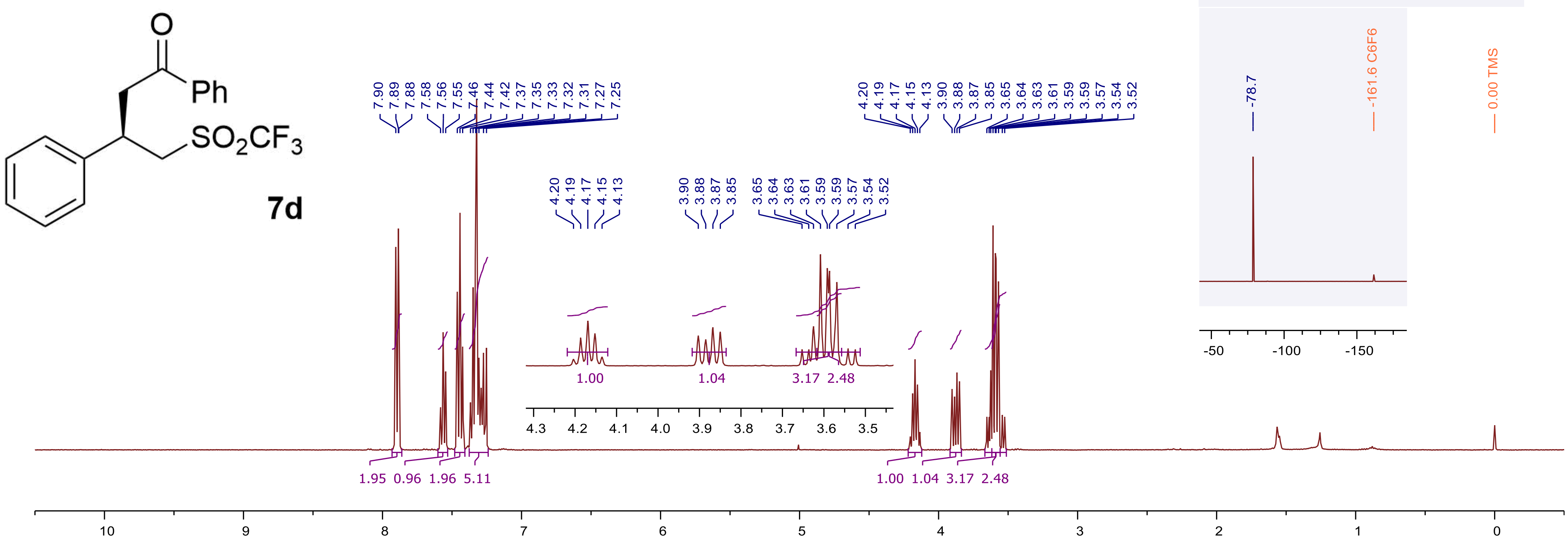

^{13}C NMR (100 MHz, $CDCl_3$)

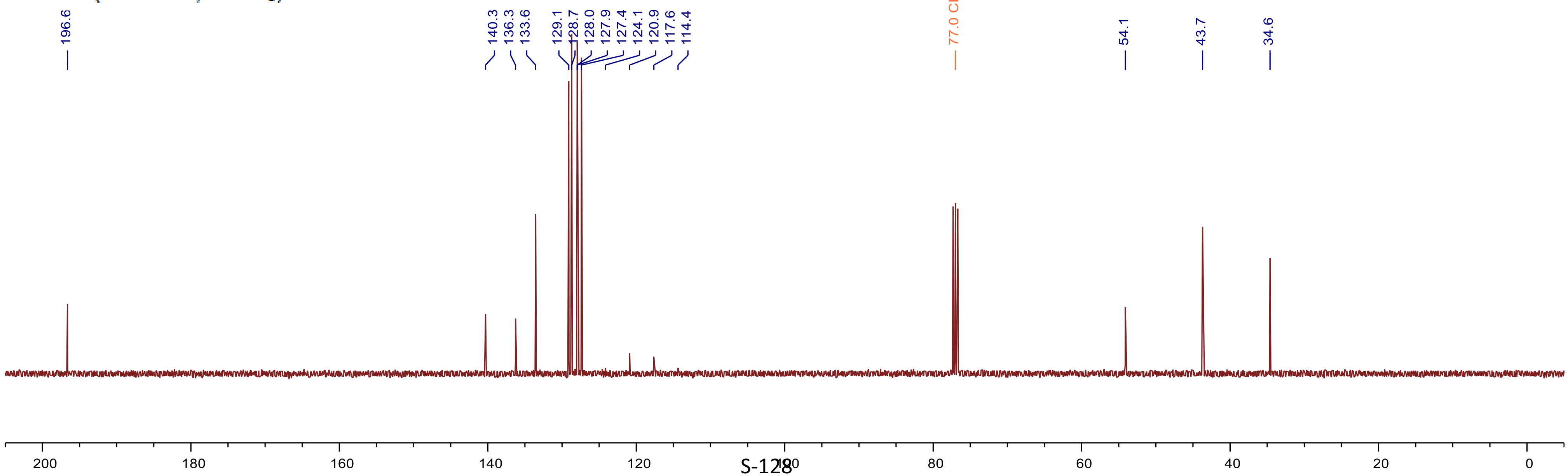

^{1}H NMR (400MHz, $CDCl_3$)

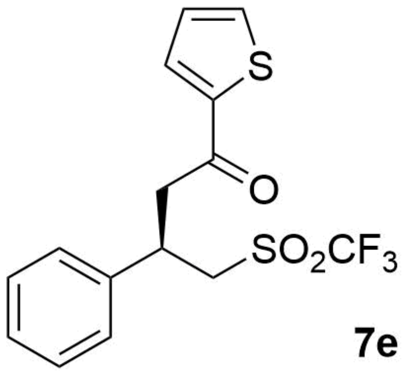

7e

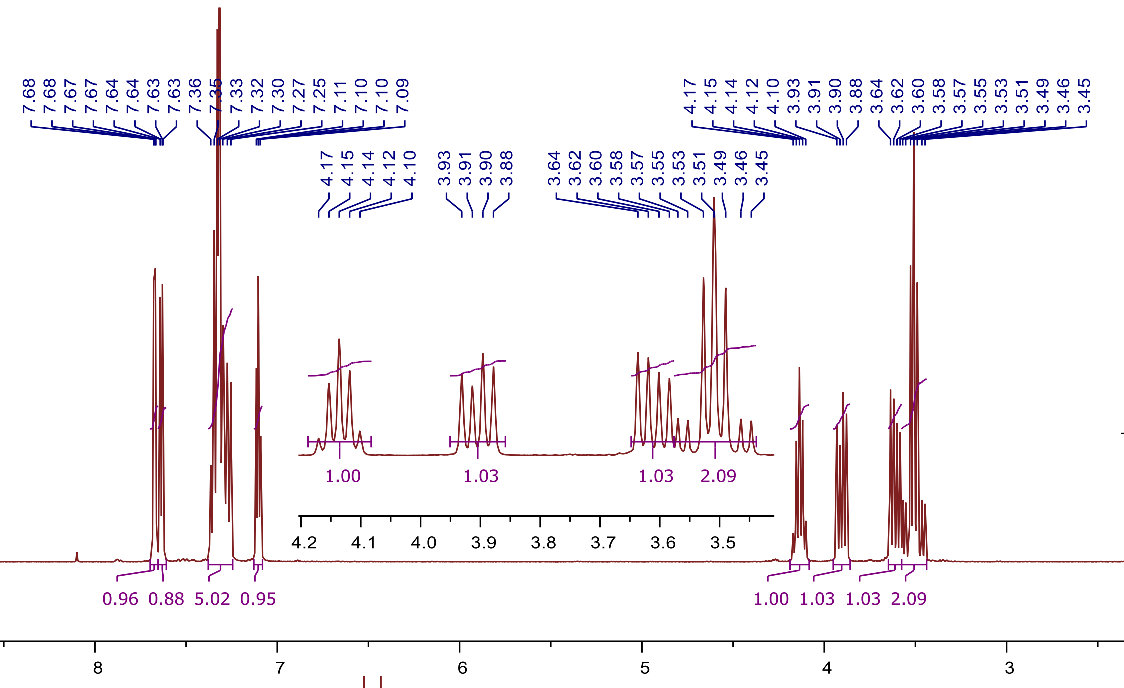

^{19}F NMR (376 MHz, $CDCl_3$)

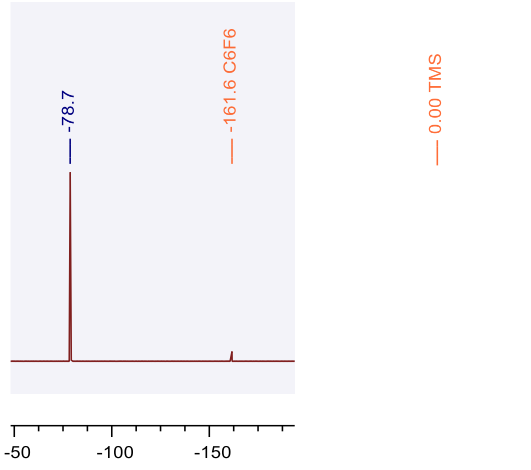

^{13}C NMR (100 MHz, $CDCl_3$)

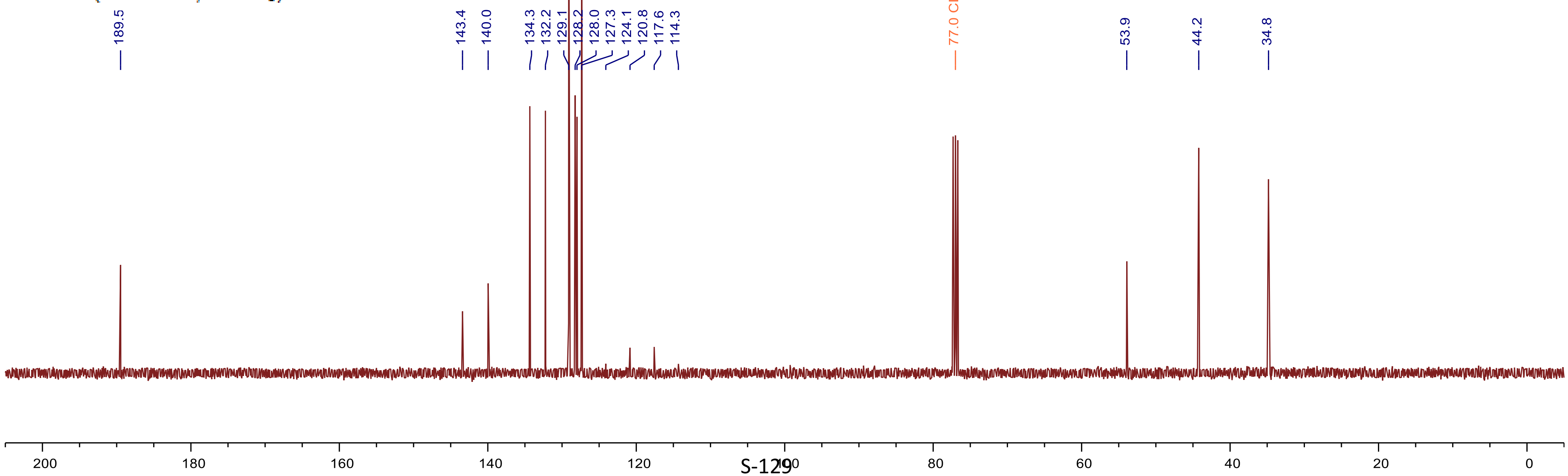

^{1}H NMR (400MHz, $CDCl_3$)

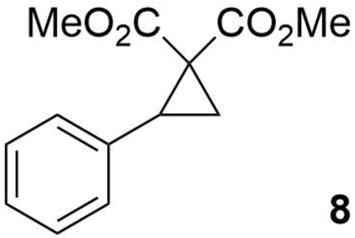

8

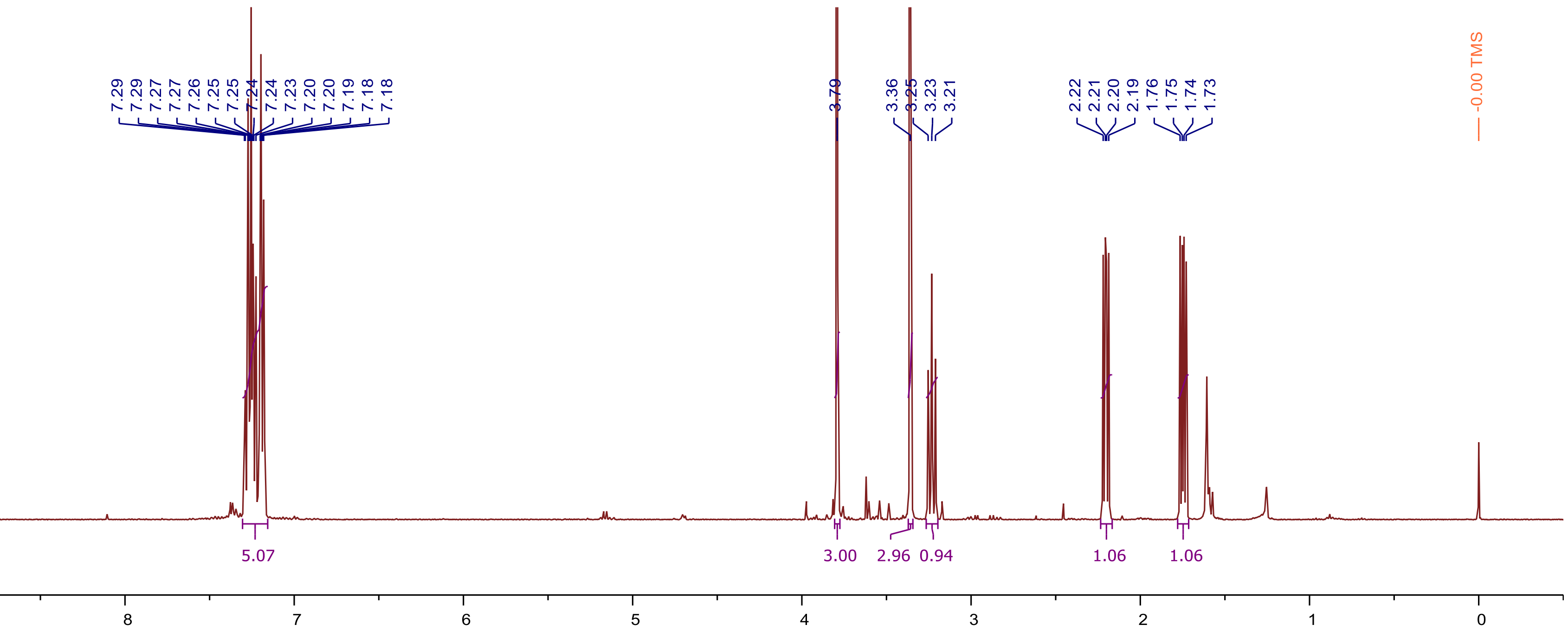

^{13}C NMR (100 MHz, $CDCl_3$)

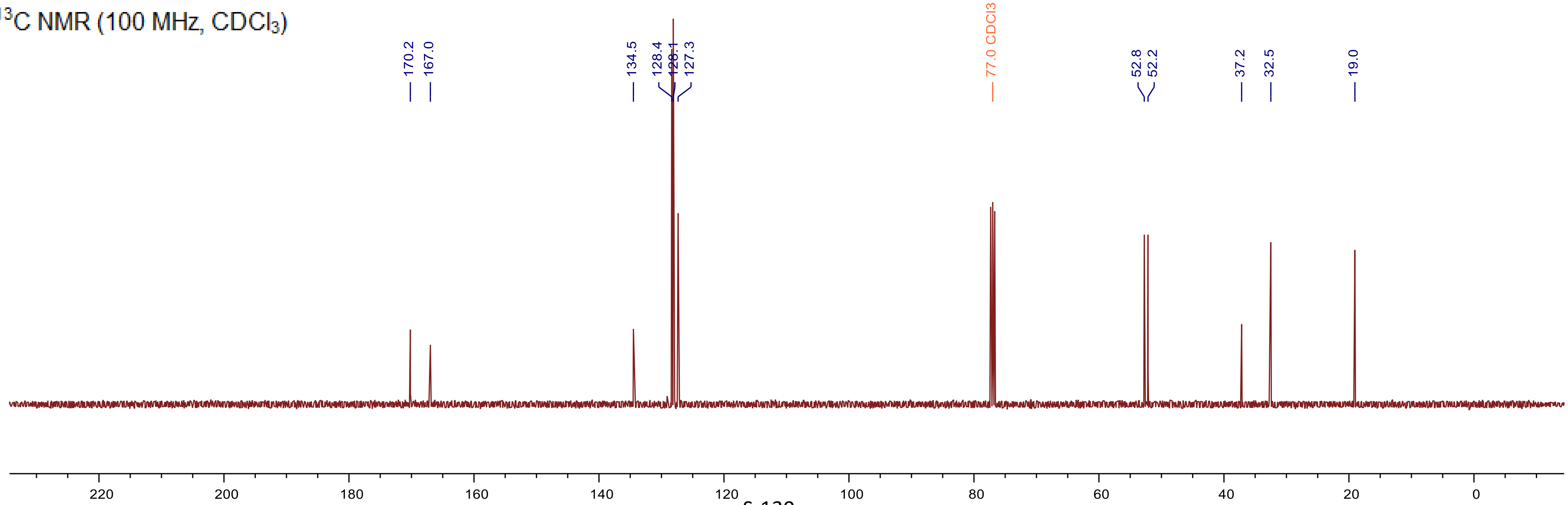

^1H NMR (400MHz, $CDCl_3$)

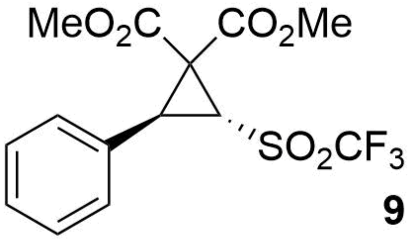

9

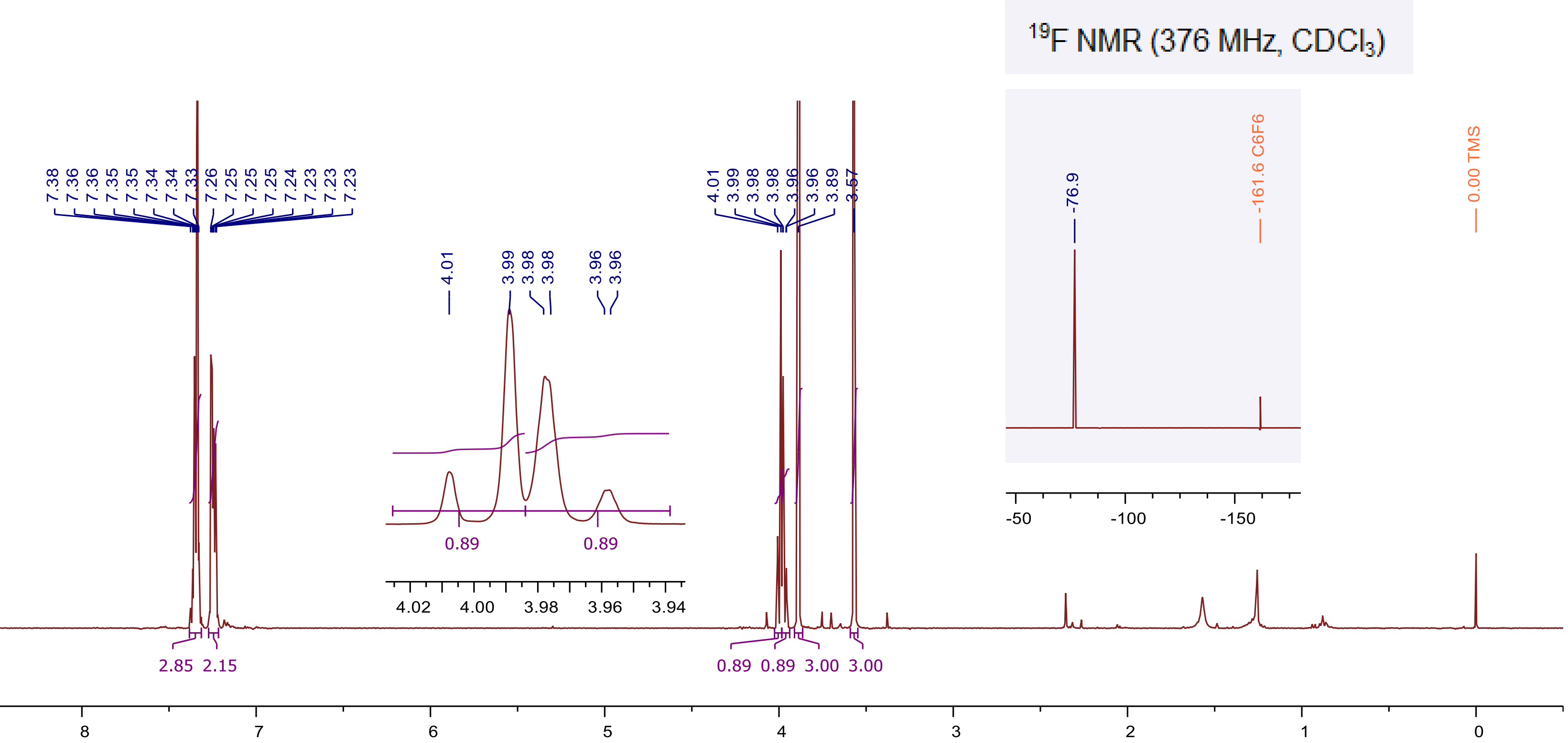

^{13}C NMR (100 MHz, $CDCl_3$)

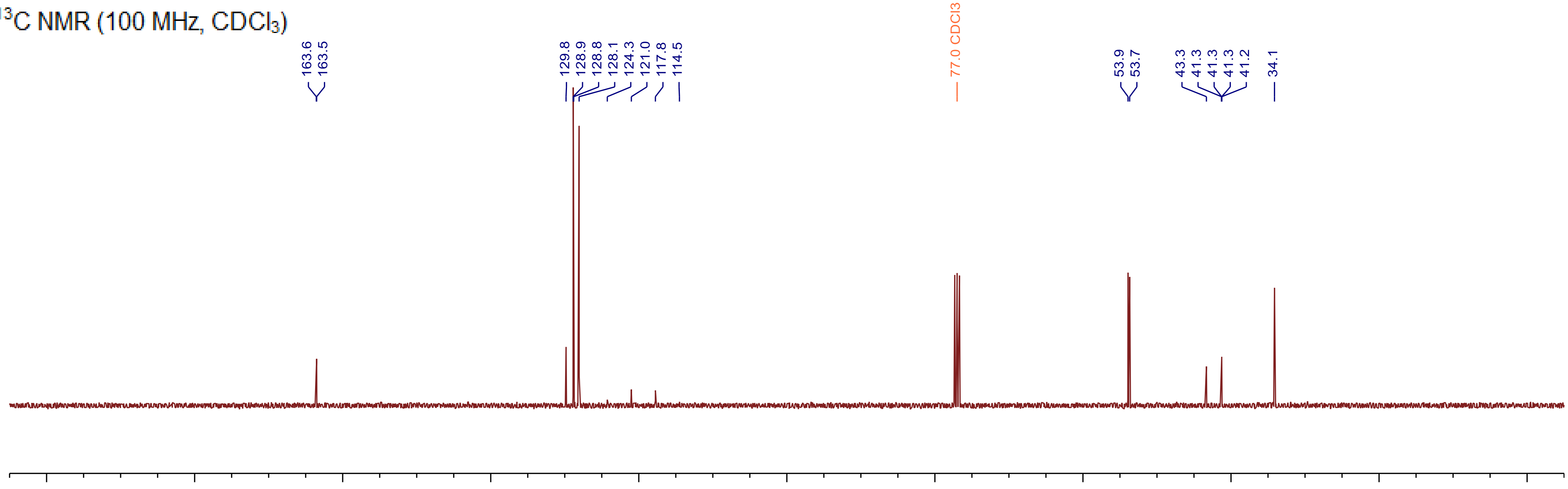

^{1}H NMR (400MHz, $CDCl_3$)

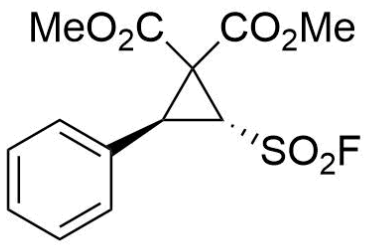

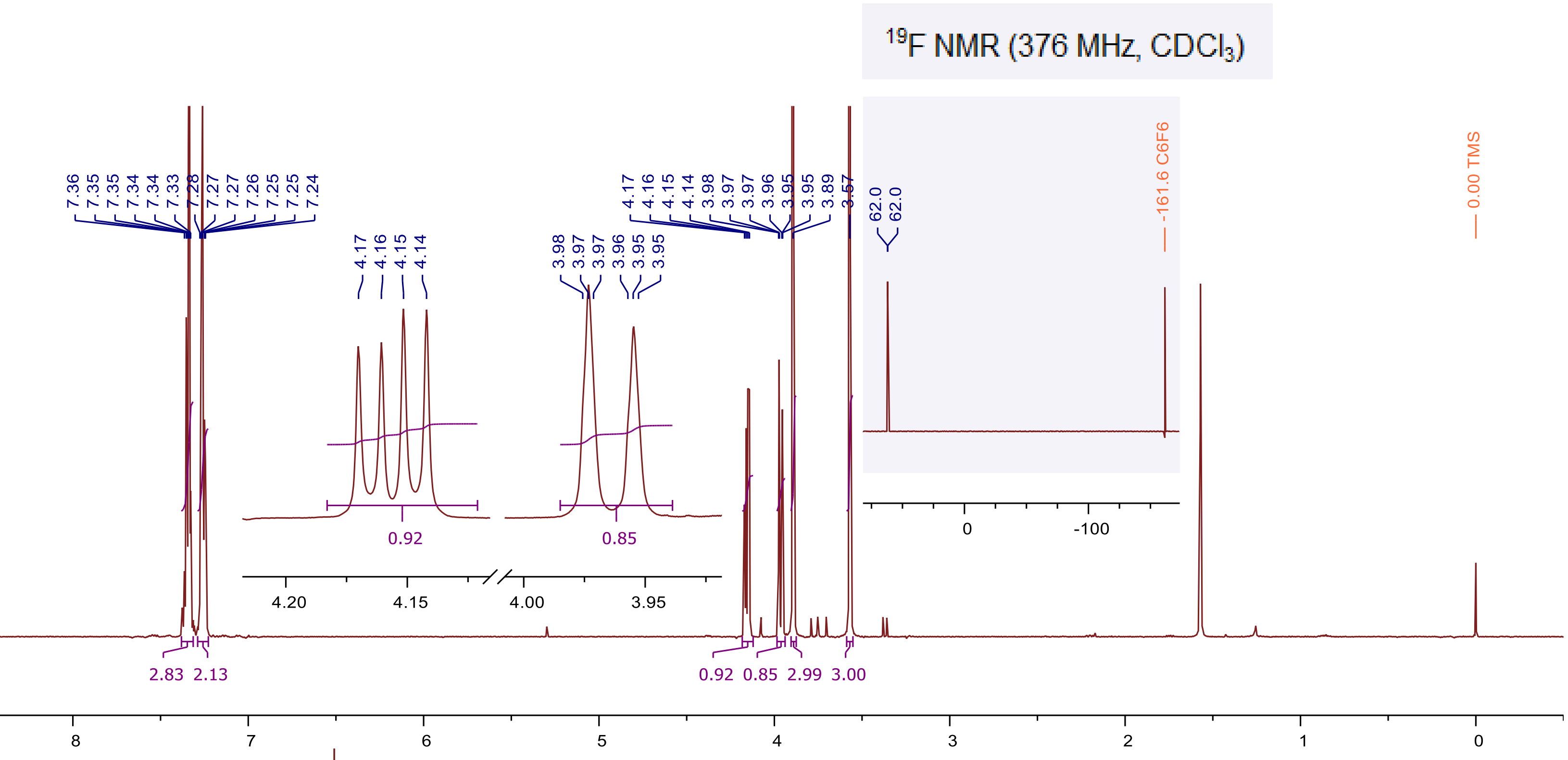

^{13}C NMR (100 MHz, $CDCl_3$)

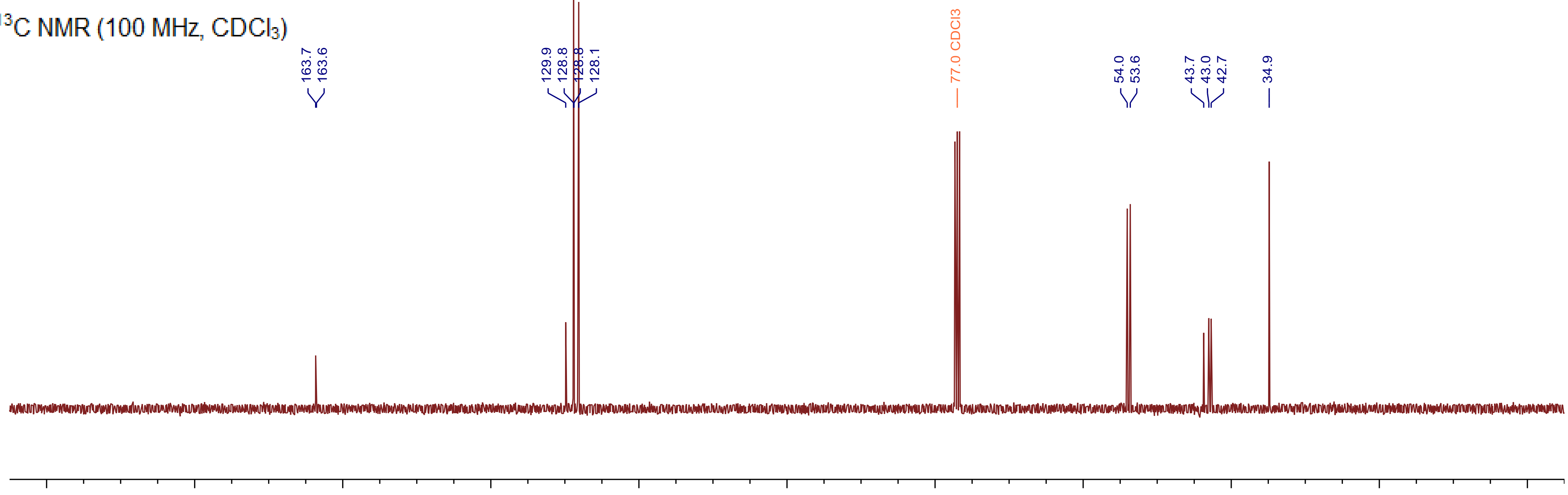

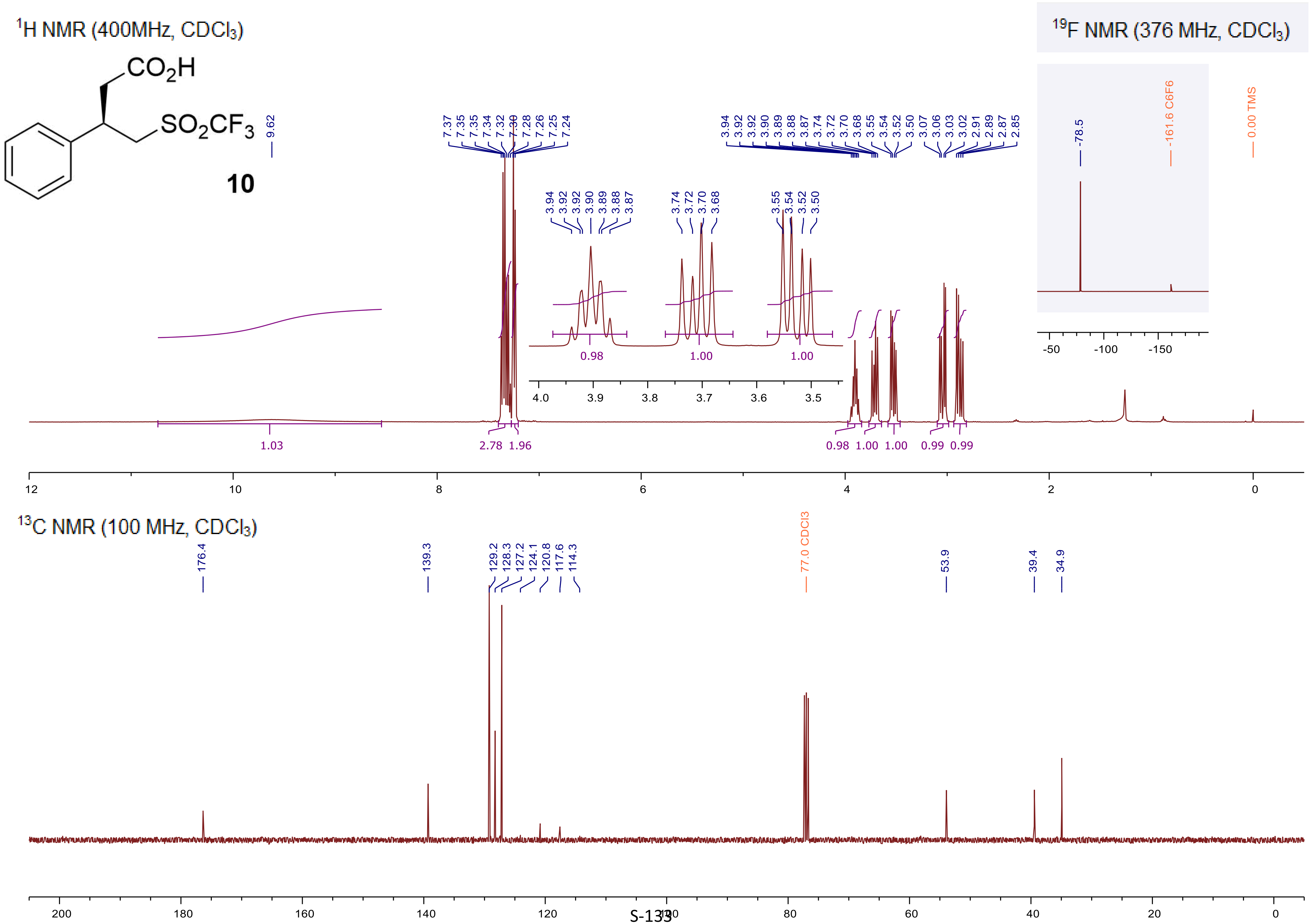

1H NMR (400MHz, CDCl3)
CO2H
SO2CF3
10
9.62
7.37 7.35 7.35 7.34 7.32 7.30 7.28 7.26 7.25 7.24
3.94 3.92 3.92 3.90 3.89 3.88 3.87 3.74 3.72 3.70 3.68 3.55 3.54 3.52 3.50 3.07 3.06 3.03 3.02 2.91 2.89 2.87 2.85
0.98 1.00 1.00
1.03 2.78 1.96 0.98 1.00 1.00 0.99 0.99
19F NMR (376 MHz, CDCl3)
-78.5
-161.6 C6F6
0.00 TMS
13C NMR (100 MHz, CDCl3)
176.4
139.3
129.2 128.3 127.2 124.1 120.8 117.6 114.3
77.0 CDCl3
53.9
39.4
34.9

^{1}H NMR (400MHz, $CDCl_3$)

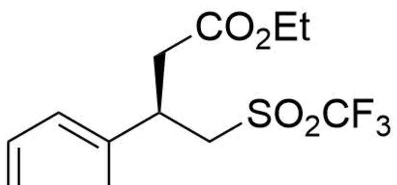

11

^{19}F NMR (376 MHz, $CDCl_3$)

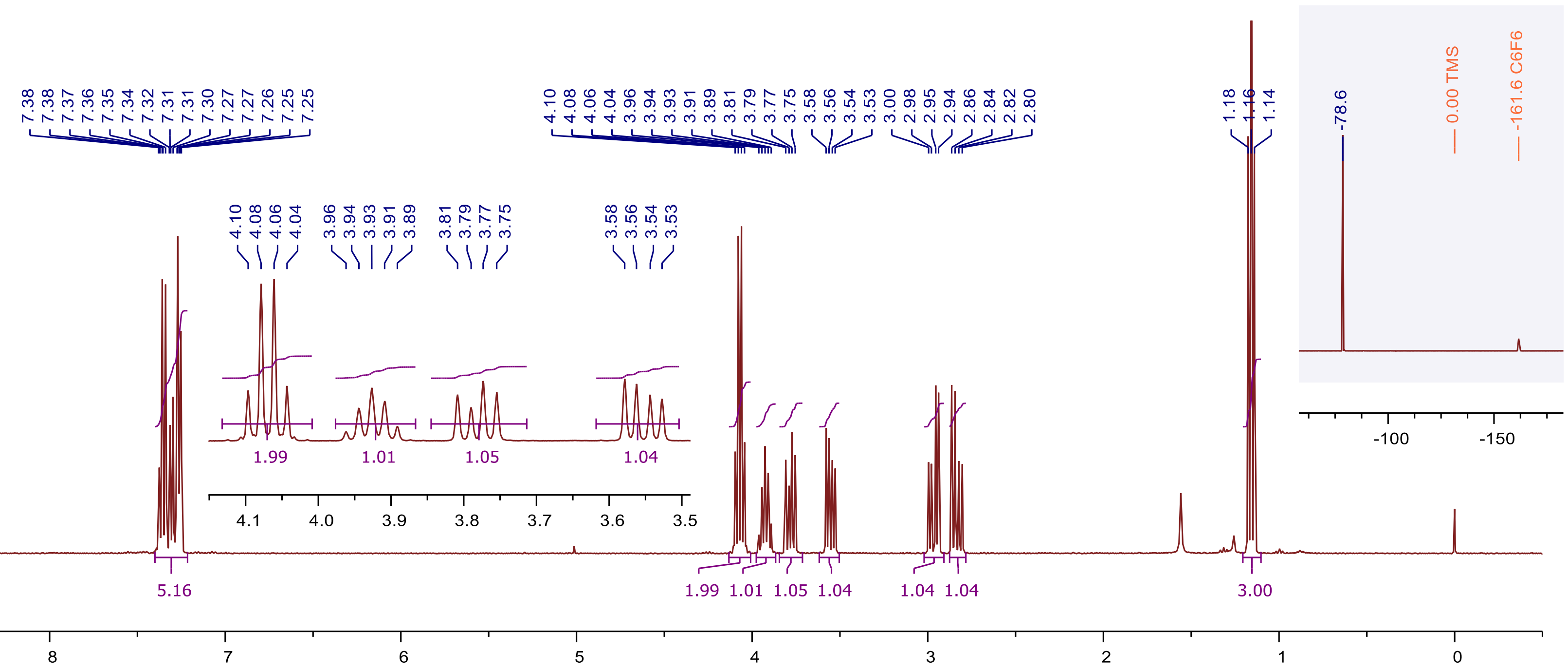

^{13}C NMR (100 MHz, $CDCl_3$)

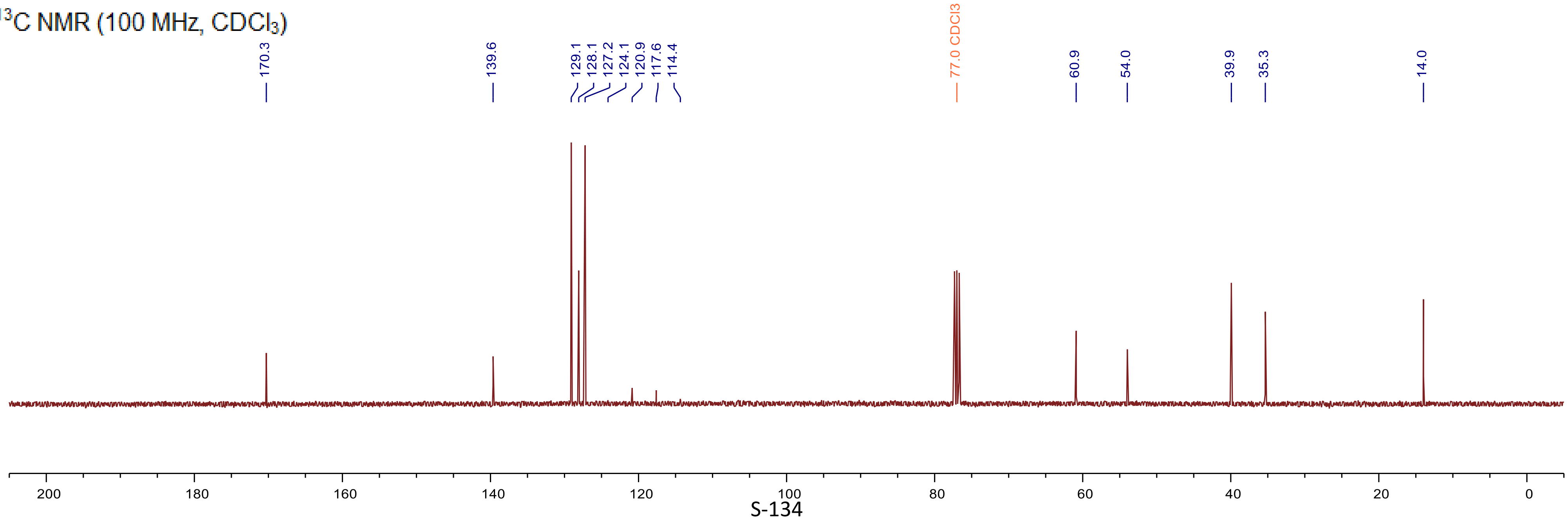

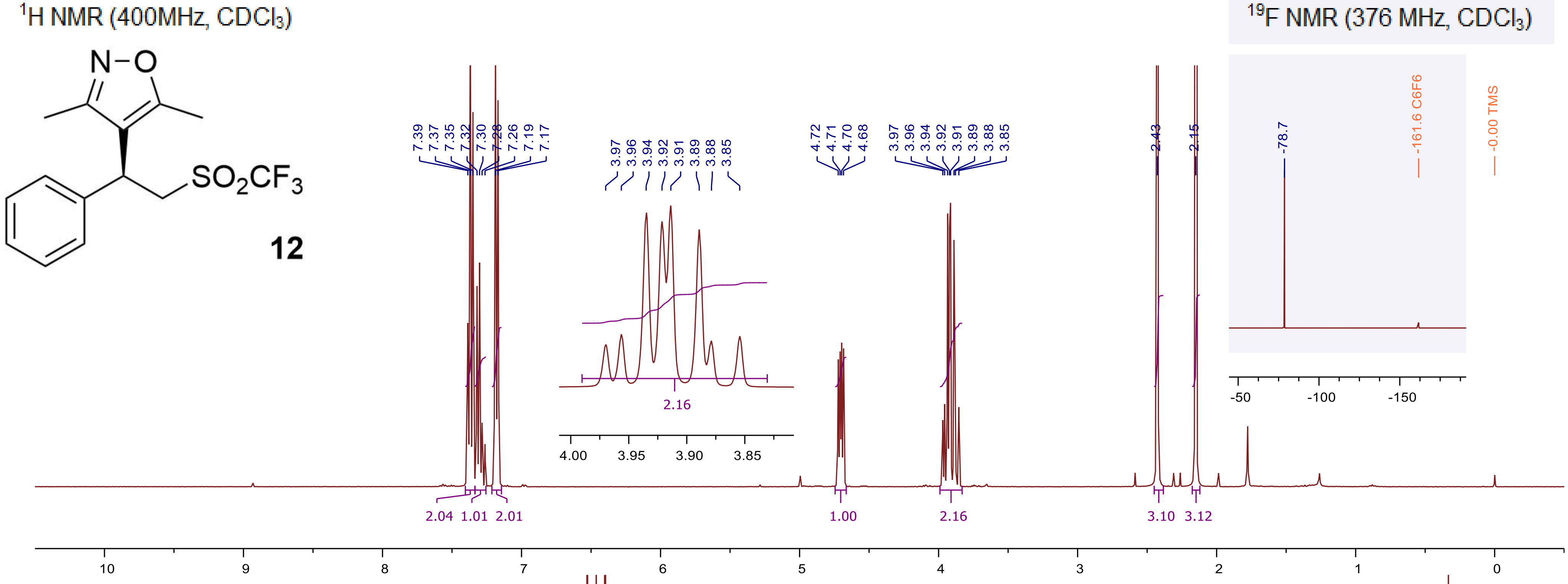

^{1}H NMR (400MHz, CDCl$_3$)
12
7.39 7.37 7.35 7.32 7.30 7.28 7.26 7.19 7.17
3.97 3.96 3.94 3.92 3.91 3.89 3.88 3.85
4.72 4.71 4.70 4.68
3.97 3.96 3.94 3.92 3.91 3.89 3.88 3.85
2.43
2.15
2.16
4.00 3.95 3.90 3.85
2.04 1.01 2.01
1.00
2.16
3.10 3.12
10 9 8 7 6 5 4 3 2 1 0
^{19}F NMR (376 MHz, CDCl$_3$)
-78.7
-161.6 C6F6
-0.00 TMS
-50 -100 -150

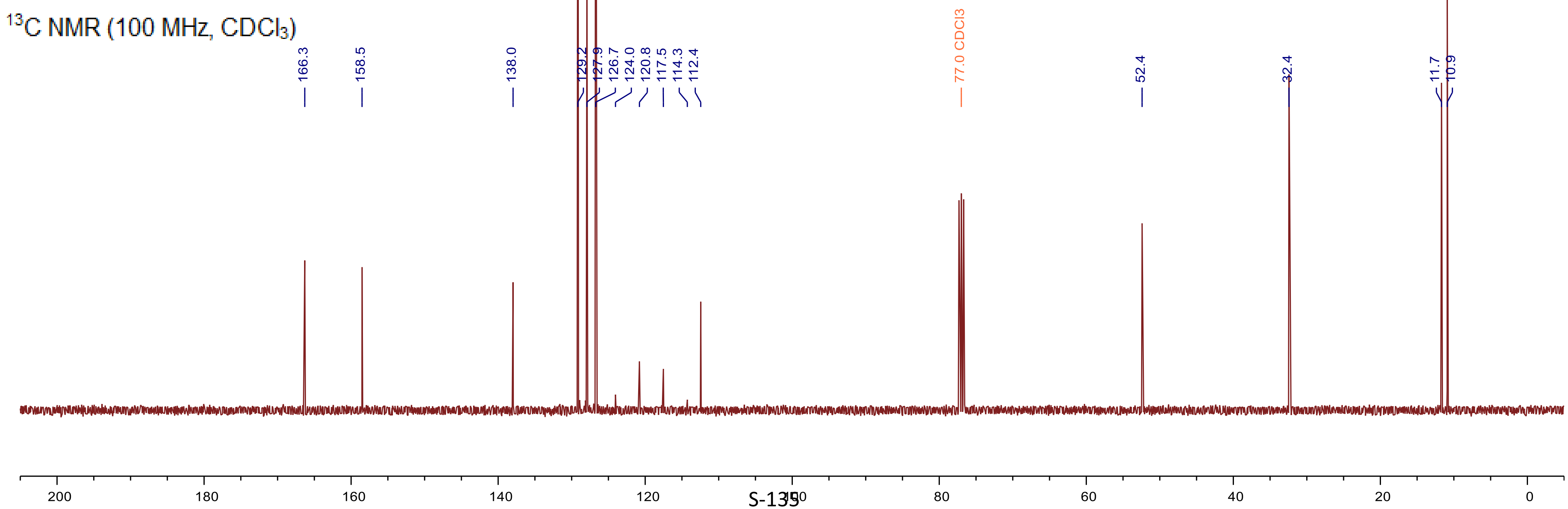

^{13}C NMR (100 MHz, CDCl$_3$)
166.3
158.5
138.0
129.2 127.9 126.7 124.0 120.8 117.5 114.3 112.4
77.0 CDCl3
52.4
32.4
11.7 10.9
200 180 160 140 120 100 80 60 40 20 0

^{1}H NMR (400MHz, $CDCl_3$)

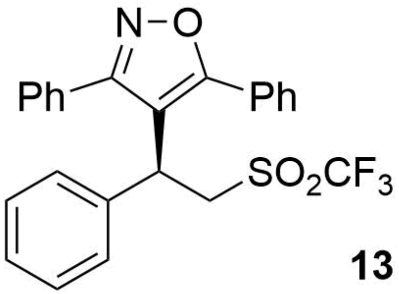

13

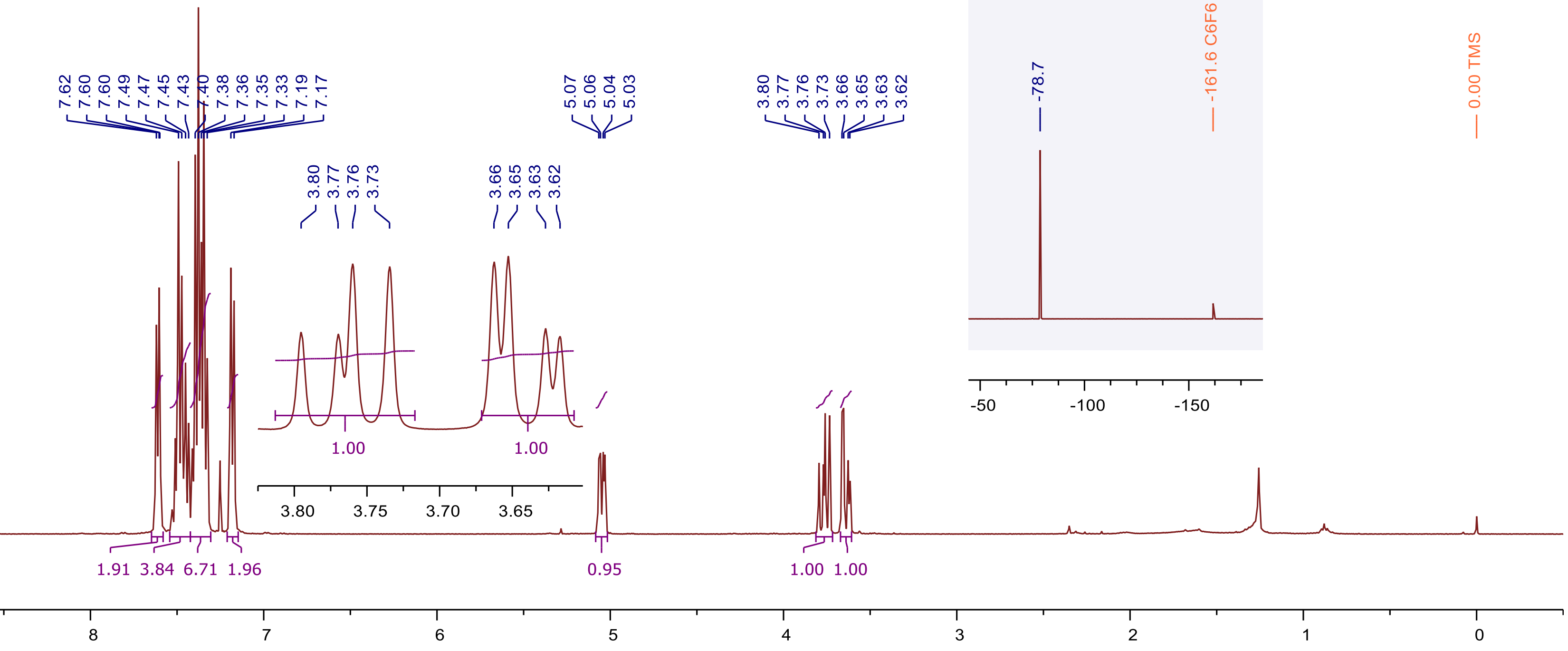

^{13}C NMR (100 MHz, $CDCl_3$)

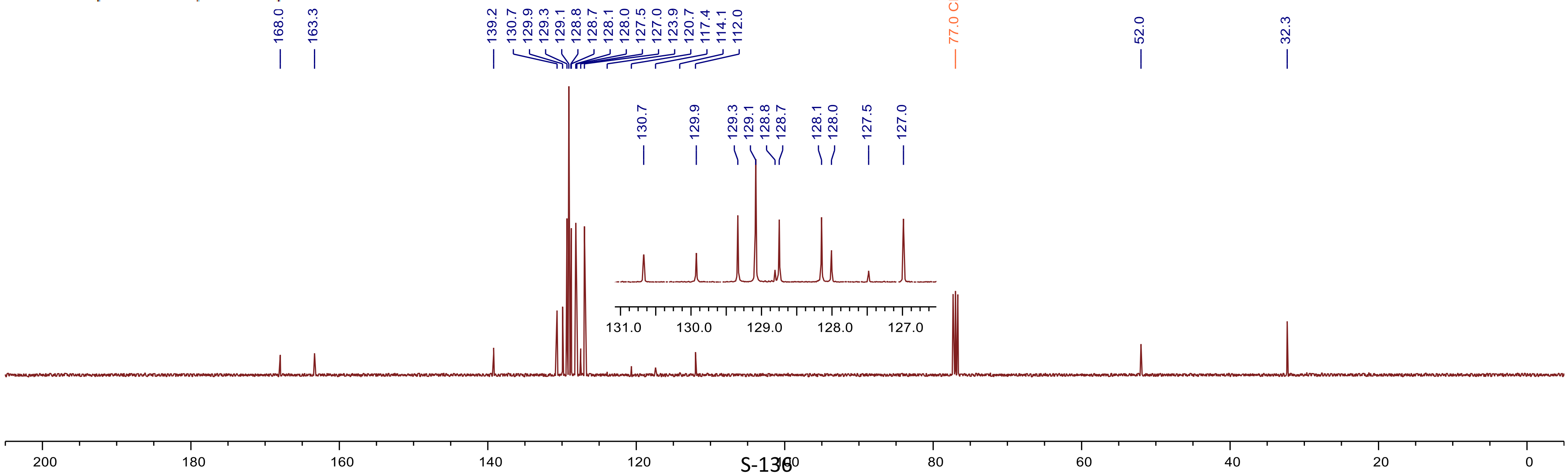

Supplement: Supplementary file 1 — ol5c00412_si_001.pdf [file ol5c00412_si_001.pdf]
